# Supplementary material for: Pd(OAc)2-catalyzed asymmetric hydrogenation of sterically hindered N-tosylimines
Source: Nat Commun. 2018 Nov 27;9:5000. doi: 10.1038/s41467-018-07462-w (PMC6258694; doi:10.1038/s41467-018-07462-w)
Supplement: Supplementary file 1 — Supplementary Information [file 41467_2018_7462_MOESM1_ESM.pdf]

Supplementary Information

$Pd(OAc)_2$-Catalyzed Asymmetric Hydrogenation of Sterically Hindered *N*-Tosylimines

Chen et al

Supplementary Methods

All reactions were performed in flame-dried glassware or in a glove box under an atmosphere of dry nitrogen, and the workup was carried out in air, unless otherwise noted. Solvents were dried and distilled before use by standard procedures. Commercially available reagents were used without further purification. (*R*)-DTBM-SegPhos, (*R*)-Seg SegPhos, (*R*)-BINAP and (*R*,*R*)-QuinozP* were purchased from Strem Chemicals Inc. and used without further purification.

^{1}H NMR (400 MHz) and ^{13}C NMR (100 MHz) spectra were recorded on a Varian MERCURY plus-400 spectrometer with TMS as an internal standard. HRMS was performed at the Analysis Center of Shanghai Jiao Tong University. Column chromatography was performed using 100-200 mesh silica gel. Melting points were measured with SGW X-4 micro melting point apparatus. Optical rotations were measured on a Rudolph Research Analytical Autopol VI automatic polarimeter using a 50 mm path-length cell at 589 nm. IR was measured with PerkinElmer Spectrum 100 FT-IR Spectrometer. Enantioselectivity was measured by high performance liquid chromatography (HPLC) using Daicel Chiralcel OD-H, AD-H, OJ-H, and IC-3 columns with hexane / 2-propanol as eluent.

Supplementary Note 1

Syntheses of *N*-tosylimines substrates

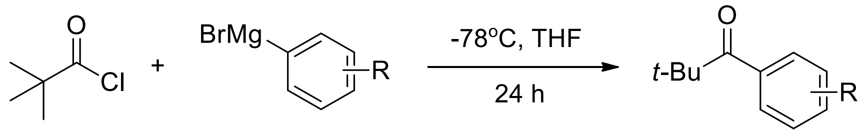

Procedure A: To a cooled (-78 °C) solution of pivaloyl chloride (3 mL) in THF (20 mL) was added a solution of freshly prepared arylmagnesium bromide (1.2 mmol in THF, 20 mL) under N_2 atmosphere. The solution was stirred for 24 h at the same temperature, then was allowed to warm to room temperature. The mixture was was diluted with saturated aqueous NH_4Cl solution (30 mL) and extracted with DCM (30 mL × 3). The combined organic phases were dried and concentrated. The residual oil was subjected to column chromatography to give colorless oil (yields, 97-88%).

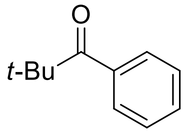

2,2-Dimethyl-1-phenylpropan-1-one (3a-e)[1]

Colorless oil. ^{1}HNMR (400 MHz, $CDCl_3$): δ 7.68 (d, J = 8.0 Hz, 2H), 7.45–7.39 (m, 3H), 1.35 (s, 9H).

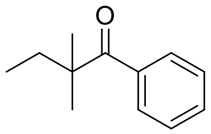

2,2-Dimethyl-1-phenylbutan-1-one (3f)[2]

Colorless oil. ^{1}HNMR (400 MHz, $CDCl_3$): δ 7.64 (d, J = 8.0 Hz, 2H), 7.43–7.35 (m, 3H), 1.78 (q, J = 8.0, 2H), 1.28 (s, 6H), 0.83 (t, J = 8.0, 3H).

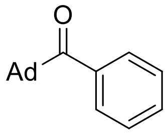

Adamantan-1-yl(phenyl)methanone (3g)[3]

White solid. ^{1}HNMR (400 MHz, $CDCl_3$): δ 7.52 (d, J = 8.0 Hz, 2H), 7.41–7.34 (m, 3H), 2.06 (s, 3H), 1.99 (s, 6H), 1.73 (s, 6H).

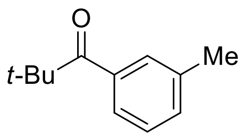

2,2-Dimethyl-1-(m-tolyl)propan-1-one (3h)[4]

Colorless oil. ^{1}HNMR (400 MHz, $CDCl_3$): δ 7.47–7.46 (m, 2H), 7.25–7.23 (m, 2H), 2.36 (s, 3H), 1.32 (s, 9H).

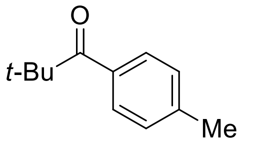

2,2-Dimethyl-1-(p-tolyl)propan-1-one (3i)[4]

Colorless oil. ^{1}HNMR (400 MHz, $CDCl_3$): δ 7.66 (d, J = 8.4 Hz, 2H), 7.19 (d, J = 8.0 Hz, 2H), 2.37 (s, 3H), 1.35 (s, 9H).

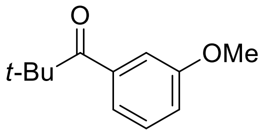

1-(3-Methoxyphenyl)-2,2-dimethylpropan-1-one (3j)[5]

Colorless oil. ^{1}HNMR (400 MHz, $CDCl_3$): δ 7.40–6.81 (m, 4H), 3.81 (s, 3H), 1.34 (s, 9H).

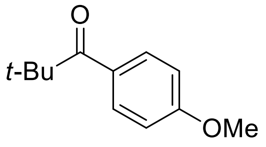

1-(4-Methoxyphenyl)-2,2-dimethylpropan-1-one (3k)[2]

Colorless oil. ^{1}HNMR (400 MHz, $CDCl_3$): δ 7.86 (d, J = 12.0 Hz, 2H), 6.89 (d, J = 12.0 Hz, 2H), 3.85 (s, 3H), 1.35 (s, 9H).

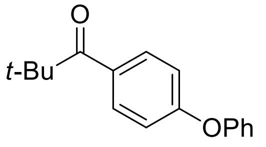

2,2-Dimethyl-1-(4-phenoxyphenyl)propan-1-one (3l)[6]

Colorless oil. ^{1}HNMR (400 MHz, $CDCl_3$): δ 7.81 (d, J = 8.0 Hz, 2H), 7.38 (t, J = 8.0 Hz, 2H), 7.18 (t, J = 8.0 Hz, 1H), 7.06 (d, J = 8.0 Hz, 2H), 6.97 (d, J = 8.0 Hz, 2H), 1.37 (s, 9H).

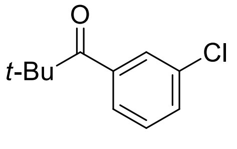

1-(3-Chlorophenyl)-2,2-dimethylpropan-1-one (3m)[4]

Colorless oil. ^{1}HNMR (400 MHz, $CDCl_3$): δ 7.63 (s, 1H), 7.54 (d, J = 8.0 Hz, 1H), 7.41 (d, J = 8.0 Hz, 1H), 7.35 (d, J = 8.0 Hz, 1H), 1.34 (s, 9H).

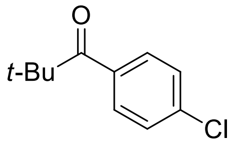

1-(4-Chlorophenyl)-2,2-dimethylpropan-1-one (3n)[2]

Colorless oil. ^{1}HNMR (400 MHz, $CDCl_3$): δ 7.65 (d, J = 8.0 Hz, 2H), 7.35 (d, J = 8.0 Hz, 2H), 1.32 (s, 9H).

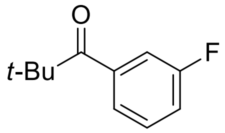

1-(3-Fluorophenyl)-2,2-dimethylpropan-1-one (3o)[4]

Colorless oil. ^{1}HNMR (400 MHz, $CDCl_3$): δ 7.46–7.36 (m, 3H), 7.14–7.11 (m, 1H), 1.35 (s, 9H).

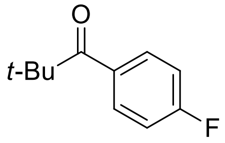

1-(4-Fluorophenyl)-2,2-dimethylpropan-1-one (3p)[4]

Colorless oil. ^{1}HNMR (400 MHz, $CDCl_3$): δ 7.77 (d, J = 8.4 Hz, 2H), 7.05 (d, J = 8.0 Hz, 2H), 1.33 (s, 9H).

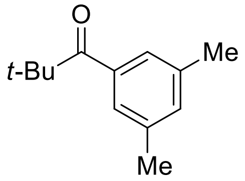

1-(3,5-Dimethylphenyl)-2,2-dimethylpropan-1-one (3q)[7]

Colorless oil. ^{1}HNMR (400 MHz, $CDCl_3$): δ 7.23 (s, 2H), 7.07 (s, 1H), 2.32 (s, 6H), 1.31 (s, 9H).

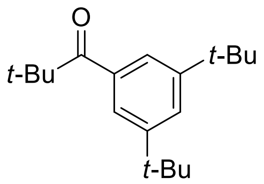

1-(3,5-Di-*tert*-butylphenyl)-2,2-dimethylpropan-1-one (3r)[8]

Colorless oil. ^{1}HNMR (400 MHz, $CDCl_3$): δ 7.44 (s, 1H), 7.23 (s, 2H), 1.37 (s, 9H), 1.33 (s, 18H).

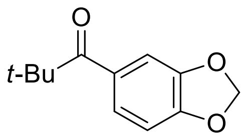

1-(Benzo[*d*][1,3]dioxol-5-yl)-2,2-dimethylpropan-1-one (3s)

Colorless oil. ^{1}HNMR (400 MHz, $CDCl_3$): δ 7.41 (d, J = 8.0 Hz, 2H), 7.29 (s, 1H), 6.79 (d, J = 8.0 Hz, 2H), 1.33 (s, 9H).

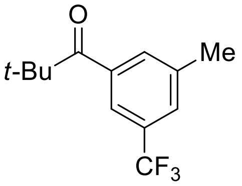

2,2-Dimethyl-1-(3-methyl-5-(trifluoromethyl)phenyl)propan-1-one (3t)

Colorless oil. ^{1}HNMR (400 MHz, $CDCl_3$): δ 7.69 (s, 1H), 7.61 (s, 1H), 7.51 (s, 1H), 2.45 (s, 3H), 1.34 (s, 9H); ^{13}C NMR (100 MHz, CDCl3): δ 207.8, 139.2, 138.9, 131.5, 130.3 (q, J = 32 Hz), 127.6 (q, J = 4 Hz), 123.8 (d, J = 136 Hz), 121.5 (q, J = 4 Hz), 44.1, 27.5, 21.0; ^{19}F NMR (376 MHz, $CDCl_3$): -62.7; IR (KBr) (v/cm^{-1}): 2973，1695，1368，1347，1156，1127，877，698； HRMS (ESI-MS) Calcd. For $C_{13}H_{15}F_3NaO$ $[M+Na]^+$ 267.0973, found: 267.0965.

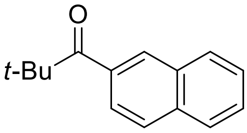

2,2-Dimethyl-1-(naphthalen-2-yl)propan-1-one (3u)[9]

Colorless oil. ^{1}HNMR (400 MHz, $CDCl_3$): δ 8.26 (s, 1H), 7.95–7.75 (m, 4H), 7.60–7.50 (m, 2H), 1.43 (s, 9H).

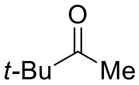

3,3-Dimethylbutan-2-one (3v)[10]

Yellow oil. ^{1}HNMR (400 MHz, $CDCl_3$): δ 2.14 (s, 3H), 1.15 (s, 9H).

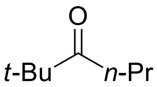

2,2-Dimethylhexan-3-one (3w)[11]

Colorless oil. ^{1}HNMR (400 MHz, $CDCl_3$): δ 2.46 (t, J = 7.2 Hz, 2H), 1.63–1.58 (m, 2H), 1.14 (s, 9H), 0.89 (t, J = 7.2, 3H).

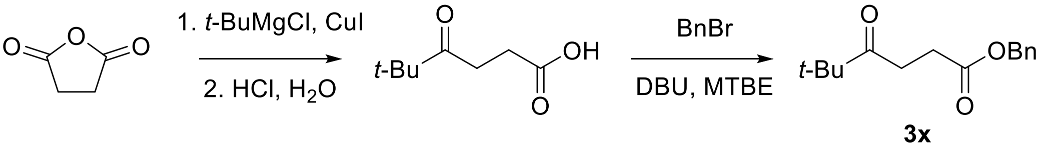

3x

Procedure B: To a stirred solution of anhydride (20 mmol) and CuI (2.0 mmol) in THF (80 mL) was added *tert*-butylmagnesium chloride (18 mmol) at −50 °C under N_2 atmosphere. The resulting mixture was allowed to lowly warm up to room temperature overnight and then quenched with HCl (50 mL, 0.1 M). The layers were separated and the aqueous layer was extracted with EtOAc (40 mL × 3). The combined organic layers were washed with brine, dried over anhydrous $MgSO_4$, and concentrated in vacuo to yield the crude product as light-yellow oil. ^{1}H NMR (400 MHz, CDCl3): δ 2.80 (t, J = 6.4 Hz, 2H), 2.60 (t, J = 6.4 Hz, 2H), 1.15 (s, 9H).

Procedure C: To a solution of ketonic acids (20 mmol) in MTBE (150 mL) at room temperature was added DBU (24 mmol) and benzyl bromide (24 mmol). The reaction mixture was stirred for 24 h. Then the resulting mixture was washed with 1 N HCl and 10 wt % aq NaCl, dried over Na_2SO_4, and concentrated in vacuo to afford corresponding crude product as light-yellow oil (63% yield). ^{1}H NMR (400 MHz, $CDCl_3$): δ 7.33–7.24 (m, 5H), 5.10 (s, 2H), 2.83–2.78 (m, 2H), 2.62–2.57 (m, 2H), 1.13 (s, 9H).

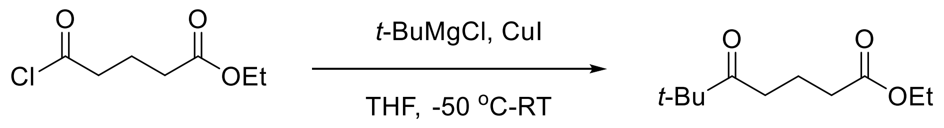

3y

Procedure B: To a stirred solution of acyl chlorides (20 mmol) and CuI (2.0 mmol) in THF (80 mL) was added tertiary butyl magnesium chloride (18 mmol) at −50 °C under N_2 atmosphere. The resulting mixture was allowed to slowly warm up to room temperature overnight and then quenched with saturated NH_4Cl (50 mL). The layers were separated and the aqueous layer was extracted with EtOAc (40 mL × 3). The combined organic layers were washed with brine, dried over anhydrous $MgSO_4$, and concentrated in vacuo to yield the desired ketoesters as a crude product that was used without purification (72% yield). ^{1}H NMR (400 MHz, $CDCl_3$): δ 4.09 (q, J = 7.2 Hz, 2H), 2.52 (q, J = 7.2 Hz, 2H), 2.28 (q, J = 7.2 Hz, 2H), 1.88–1.81 (m, 2H), 1.23–1.19 (m, 2H), 1.09 (s, 9H).

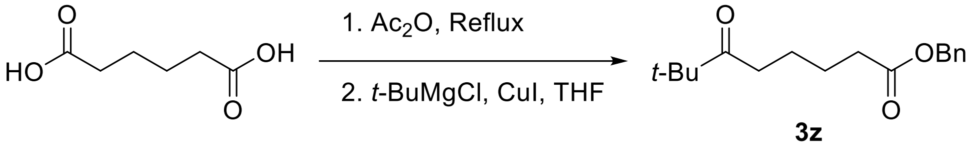

3z

Procedure D: Under argon atmosphere, a solution of hexanedioic acid (10 mmol) in acetic anhydride (2.5 mL/mmol) was stirred at reflux during 6 h to yield the corresponding anhydride. The solvent was evaporated under reduced pressure and the

resulting solid used in the next step 2 without further purification.

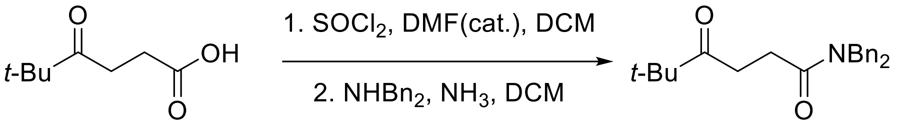

3aa

Procedure E: To a solution of ketonic acids (20 mmol) in DCM (50 mL) at room temperature was added DMF (0.1 mL) and $SOCl_2$ (30 mmol). The reaction mixture was stirred for 8 h. Then the resulting mixture was concentrated in vacuo. 100 mL of DCM, Et_3N (30 mmol) and $NHBn_2$ (30 mmol) were added and stirred overnight at room teperature. The reaction mixture was washed with 1 N HCl and saturated brines, dried over Na_2SO_4, and concentrated in vacuo to afford the corresponding crude product as light-yellow oil (68% yield). ^1H NMR (400 MHz, $CDCl_3$): δ 7.66–7.49 (m, 10H), 4.88 (s, 2H), 4.80 (s, 2H), 3.26–3.20 (m, 2H), 3.00–2.93 (m, 2H), 1.48 (s, 9H).

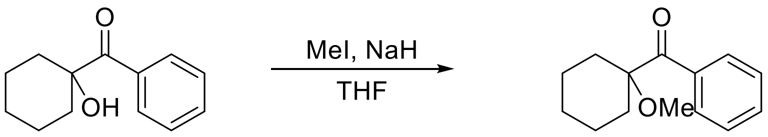

3ad

Procedure F: To a solution of (1-hydroxycyclohexyl)(phenyl)methanone (10 mmol) in THF (60 mL) at 0 ^oC was added methyl iodide (11 mmol) and NaH (11 mmol). The resulting mixture was allowed to slowly warm up to RT and stirred for 8 h, then quenched with saturated NH_4Cl (50 mL). The layers were separated and the aqueous layer was extracted with EtOAc (40 mL × 3). The combined organic layers were washed with brine, dried over anhydrous $MgSO_4$, and concentrated in vacuo to yield the crude product that was used after purification by flash chromatography (93% yield). ^1H NMR (400 MHz, $CDCl_3$): δ 8.24 (q, J = 8.0 Hz, 2H), 7.50 (t, J = 7.2 Hz, 1H), 7.41–7.37 (m, 2H), 3.12 (s, 3H), 2.04–2.00 (m, 2H), 1.79–1.51 (m, 8H).

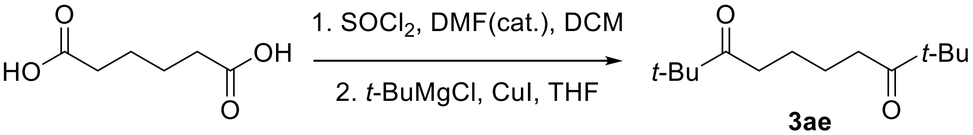

3ae

Procedure E and B, 43% yield. ^1H NMR (400 MHz, $CDCl_3$): δ 2.45 (s, 4H), 1.28 (m, 4H), 1.09 (s, 18H).

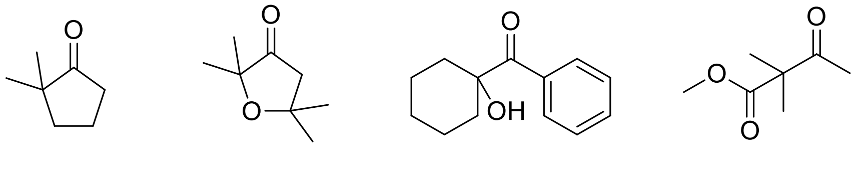

Those above compounds were purchased from reagent companies.

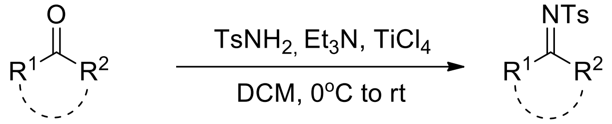

3 1

Procedure G: To a cooled solution of **3** (5.0 mmol), $TsNH_2$ (6.0 mmol), and Et_3N (25.0 mmol) in DCM (50 mL) was added $TiCl_4$ (5.0 mmol). After 12-24 h of stirring at room temperature, saturated aqueous $NaHCO_3$ (20 mL) was added. The mixture was filtered and the filtrate was extracted with DCM (20 mL × 2). The combined organic phases were dried, and concentrated. The residue was purified by flash chromatography and recrystallization to give **1** (yields, 71-35 %).

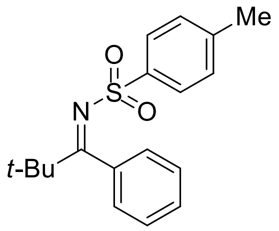

(*Z*)-*N*-(2,2-Dimethyl-1-phenylpropylidene)-4-methylbenzenesulfonamide (1a)[12]

White solid, ^{1}H NMR (400 MHz, $CDCl_3$): δ 7.68 (d, J = 8.0 Hz, 2H), 7.39–7.35 (m, 3H), 7.22 (d, J = 8.4 Hz, 2H), 7.11 (d, J = 8.0 Hz, 2H), 2.42 (s, 3H), 1.20 (s, 9H).

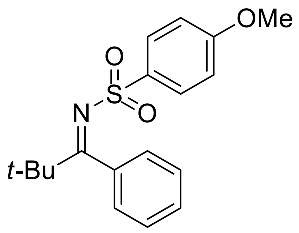

(*Z*)-*N*-(2, 2-Dimethyl-1-phenylpropylidene)-4-methoxybenzenesulfonamide (1b)

White solid, mp: 110−112 °C; ^{1}H NMR (400 MHz, $CDCl_3$): δ 7.70 (d, J = 8.0 Hz, 2H), 7.38–7.34 (m, 3H), 7.10-7.08 (m, 2H), 6.88 (d, J = 8.0 Hz, 2H), 3.85 (s, 3H), 1.18 (s, 9H); ^{13}C NMR (100 MHz, $CDCl_3$): δ 192.7, 162.7, 135.3, 132.8, 129.3, 128.8, 127.4, 126.2, 113.8, 55.6, 43.0, 27.8; IR (KBr) (v/cm^{-1}): 2974, 2934, 1595, 1499, 1326, 1153, 838, 807; HRMS (ESI-MS) Calcd. For $C_{18}H_{21}NNaO_3S$ [M+Na]$^+$ 342.0940, found: 342.0947.

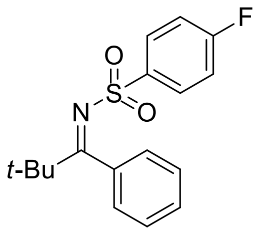

(*Z*)-*N*-(2, 2-Dimethyl-1-phenylpropylidene)-4-fluorobenzenesulfonamide (1c)

White solid, mp: 109−111 °C; ^{1}H NMR (400 MHz,CDCl$_3$) *δ* 7.82–7.79 (m, 2H), 7.41–7.36 (m, 3H), 7.12–7.08 (m, 4H), 1.19 (s, 9H); ^{13}C NMR (100 MHz, CDCl$_3$): δ 193.9, 166.2, 163.7, 137.2, 135.3, 130.0 (d, J = 36.0 Hz), 129.0, 127.5, 126.2, 116.0, 115.7, 43.1, 27.8; ^{19}F NMR (376 MHz, CDCl$_3$): -27.9; IR (KBr) (v/cm^{-1}): 3109, 2976, 2933, 1598, 1591, 1163, 1095, 841, 817, 737; HRMS (ESI-MS) Calcd. For $C_{17}H_{18}FNNaO_2S$ [M+Na]$^+$ 354.1140, found: 354.1138.

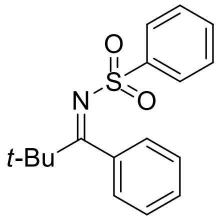

(*Z*)-*N*-(2,2-Dimethyl-1-phenylpropylidene)benzenesulfonamide (1d)

White solid, mp: 102−104 °C; ^{1}H NMR (400 MHz,CDCl$_3$) *δ* 7.80 (d, J = 8.0 Hz, 2H), 7.54–7.51 (m, 1H), 7.45–7.33 (m, 5H), 7.45–7.33 (m, 2H), 1.20 (s, 9H); ^{13}C NMR (100 MHz, CDCl$_3$): δ 193.8, 141.1, 135.3, 132.5, 128.9, 127.5, 127.1, 126.2, 43.1, 27.8; IR (KBr) (v/cm^{-1}): 3032, 2988, 2935, 1623, 1608, 1322, 1168, 839, 786, 732; HRMS (ESI-MS) Calcd. For $C_{17}H_{20}NO_2S$ [M+H]$^+$ 302.1215, found: 302.1214.

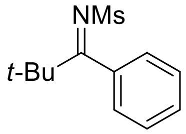

***N*-(2,2-Dimethyl-1-phenylpropylidene)methanesulfonamide (1e)**

White solid, mp: 85−87 °C; ^{1}H NMR (400 MHz, CDCl$_3$): δ 7.38 (s, 3H), 7.16 (s, 2H), 3.11 (s, 3H), 1.20 (s, 9H); ^{13}C NMR (100 MHz, CDCl$_3$): δ 193.9, 135.2, 129.0, 127.5, 126.3, 42.8, 42.7, 27.8; IR (KBr) (v/cm^{-1}): 3289, 2961, 2871, 1457,1319, 1155, 1089, 1062, 1023, 979, 793, 735,705,517; HRMS (ESI-MS) Calcd. For $C_{12}H_{18}NO_2S$ [M+H]$^+$ 240.1058, found: 240.1057.

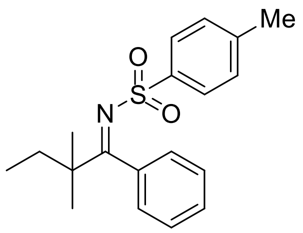

(Z)-*N*-(2,2-Dimethyl-1-phenylbutylidene)-4-methylbenzenesulfonamide (1f)

White solid, mp: 117−119 °C; ^{1}H NMR (400 MHz,CDCl$_3$) *δ* 7.67 (d, J = 8.0 Hz, 2H), 7.39–7.33 (m, 3H), 7.22 (d, J = 8.0 Hz, 2H), 7.09 (d, J = 8.0 Hz, 2H), 2.40 (s, 3H), 1.59 (q, J = 8.0 Hz, 2H), 1.11 (s, 6H), 0.90 (t, J = 8.0 Hz, 2H); ^{13}C NMR (100 MHz, CDCl$_3$): δ 192.8, 143.2, 138.4, 135.6, 129.2, 128.8, 127.4, 127.1, 126.0, 46.3, 32.9, 25.4, 21.6, 9.1; IR (KBr) (v/cm^{-1}): 2975, 1604, 1586, 1325, 1152, 1091, 981; HRMS (ESI-MS) Calcd. For $C_{19}H_{24}NO_2S$ [M+H]$^+$ 330.1528, found: 330.1532.

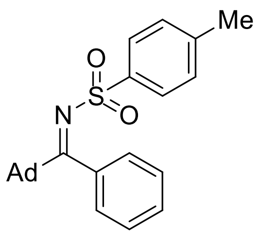

(Z)-*N*-(Adamantan-1-yl(phenyl)methylene)-4-methylbenzenesulfonamide (1g)

White solid, mp: 171−174 °C; ^{1}H NMR (400 MHz,CDCl$_3$) *δ* 7.68 (d, J = 8.0 Hz, 2H), 7.41–7.33 (m, 3H), 7.22 (d, J = 8.0 Hz, 2H), 7.07 (d, J = 8.0 Hz, 2H), 2.40 (s, 3H), 2.01 (s, 3H), 1.79 (s, 6H), 1.65 (dd, J = 12.0, 36.0 Hz, 6H); ^{13}C NMR (100 MHz, CDCl$_3$): δ 1.92.7, 143.1, 138.4, 134.8, 129.2, 128.7, 127.3, 127.2, 126.3, 44.7, 39.1, 36.2, 28.0, 21.6; IR (KBr) (v/cm^{-1}): 2926, 2900, 1603, 1583, 1326, 1301, 847, 818; HRMS (ESI-MS) Calcd. For $C_{24}H_{28}NO_2S$ [M+H]$^+$ 394.1841, found: 394.1847.

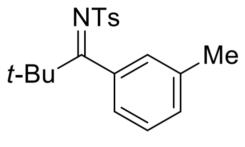

(Z)-*N*-(2, 2-Dimethyl-1-(m-tolyl)propylidene)-4-methylbenzenesulfonamide (1h)

White solid, mp: 106−108 °C; ^{1}H NMR (400 MHz, CDCl$_3$): δ 7.59 (d, J = 8.4 Hz, 2H), 7.20−7.12 (m, 4H), 6.88 (d, J = 7.6 Hz, 1H), 6.76 (s, 1H), 2.35 (s, 3H), 2.28 (s, 3H), 1.16 (s, 9H); ^{13}C NMR (100 MHz, CDCl$_3$): δ 190.9, 140.6, 135.6, 134.4, 132.6, 127.0, 126.6, 124.7, 124.6, 124.0, 120.9, 40.4, 25.3, 19.0, 18.9; IR (KBr) (v/cm^{-1}): 2975,

2930, 2869, 1613, 1596, 813, 802, 736; HRMS (ESI-MS) Calcd. For $C_{19}H_{24}NO_2S$ $[M+H]^+$330.1520, found: 330.1528.

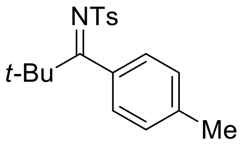

(Z)-*N*-(2, 2-Dimethyl-1-(p-tolyl) propylidene)-4-methylbenzenesulfonamide(1i)

White solid, mp: 132−134 °C; ^{1}H NMR (400 MHz, $CDCl_3$): δ 7.70 (d, J = 8.4 Hz, 2H), 7.25 (d, J = 8.0 Hz, 2H), 7.21−7.15 (m, 2H), 7.02 (d, J = 8.0 Hz, 2H), 2.40 (s, 3H), 2.38 (s, 3H), 1.18 (s, 9H); ^{13}C NMR (100 MHz, $CDCl_3$): δ 193.6, 143.1, 138.8, 138.5, 132.6, 129.2, 128.1, 127.2, 126.2, 43.0, 27.9, 21.5, 21.4; IR (KBr) (v/cm^{-1}): 2978, 2934, 2872, 1613, 1589, 842, 819,760; HRMS (ESI-MS) Calcd. For $C_{19}H_{24}NO_2S$ $[M+H]^+$ 330.1523, found: 330.1528.

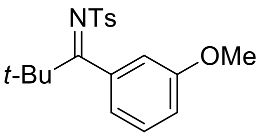

(Z)-*N*-(1-(3-Methoxyphenyl)-2, 2-dimethylpropylidene)-4-methylbenzenesulfonamide (1j)

White solid, mp: 118−120 °C; ^{1}H NMR (400 MHz, $CDCl_3$): δ 7.64 (d, J = 8.0 Hz, 2H), 7.28−7.24 (m, 1H), 7.20 (d, J = 8.0 Hz, 2H), 6.89 (d, J = 8.0 Hz, 1H), 6.68 (d, J = 7.6 Hz, 1H), 6.55 (s, 1H), 3.78 (s, 3H), 2.40 (s, 3H), 1.18 (s, 9H); ^{13}C NMR (100 MHz, $CDCl_3$): δ 192.7, 158.5, 143.2, 138.2, 136.5, 129.2, 128.6, 127.3, 118.8, 114.2, 112.3, 55.2, 43.0, 28.0, 21.5; IR (KBr) (v/cm^{-1}): 3356, 3261, 1607, 1540, 831, 812, 773; HRMS (ESI-MS) Calcd. For $C_{19}H_{23}NO_3SNa$ $[M+Na]^+$ 368.1293, found:368.1296.

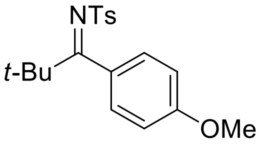

(Z)-*N*-(1-(4-Methoxyphenyl)-2, 2-dimethylpropylidene)-4-methylbenzenesulfonamide (1k)

White solid, mp: 115−117 °C; ^{1}H NMR (400 MHz, $CDCl_3$): δ 7.69 (d, J = 8.0 Hz, 2H), 7.26−7.22 (m, 2H), 7.07 (d, J = 8.4 Hz, 2H), 6.87 (d, J = 8.8 Hz, 2H), 3.83 (s, 3H), 2.40 (s, 3H), 1.18 (s, 9H); ^{13}C NMR (100 MHz, $CDCl_3$): δ 193.6, 159.9, 143.1, 138.5, 129.2, 127.9, 127.8, 127.2, 112.9, 55.2, 43.2, 28.0, 21.6; IR (KBr) (v/cm^{-1}): 3356, 3261, 1607, 1540, 831, 812, 773; HRMS (ESI-MS) Calcd. For $C_{19}H_{24}NO_3S$ $[M+H]^+$

346.1477, found:346.1477.

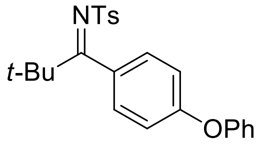

(*Z*)-*N*-(2, 2-Dimethyl-1-(4-phenoxyphenyl)propylidene)-4-methylbenzenesulfonamide (1l)

White solid, mp: 118−120 °C; ^{1}H NMR (400 MHz, $CDCl_3$): δ 7.70 (d, J = 8.4 Hz, 2H), 7.40−7.36 (m, 3H), 7.24 (s, 1H), 7.18−7.15 (m, 1H), 7.09−7.06 (m, 4H), 6.94(d, J = 8.8 Hz, 2H), 2.41 (s, 3H), 1.19 (s, 9H); ^{13}C NMR (100 MHz, $CDCl_3$): δ 193.2, 158.1, 156.0, 143.3, 138.4, 130.0, 129.9, 129.3, 128.1, 127.2, 124.1, 119.9, 116.9, 43.2, 27.9, 21.6; IR (KBr) (v/cm^{-1}): 2971, 1588, 1501, 878, 838, 813; HRMS (ESI-MS) Calcd. For $C_{24}H_{26}NO_3S$ $[M+H]^+$ 408.1633, found: 408.1634.

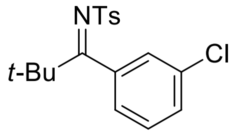

(*Z*)-*N*-(1-(3-Chlorophenyl)-2,2-dimethylpropylidene)-4-methylbenzenesulfonamide (1m)

White solid, mp: 119−121 °C; ^{1}H NMR (400 MHz, $CDCl_3$): δ 7.62 (d, J = 8.0 Hz, 2H), 7.35−7.28 (m, 2H), 7.22 (d, J = 8.0 Hz, 2H), 7.02 (d, J = 8.0 Hz, 1H), 6.94(s, 1H), 2.41 (s, 3H), 1.18 (s, 9H); ^{13}C NMR (100 MHz, $CDCl_3$): δ 191.0, 143.6, 137.7, 136.6, 133.7, 129.3, 129.0, 128.9, 127.2, 126.0, 124.8, 43.0, 27.8, 21.6; IR (KBr) (v/cm^{-1}): 2975, 2932, 1609, 1590, 1477, 1328, 880, 849, 768; HRMS (ESI-MS) Calcd. For $C_{18}H_{20}ClNNaO_2S$ $[M+Na]^+$ 372.0801, found: 372.0798.

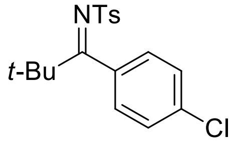

(*Z*)-*N*-(1-(4-Chlorophenyl)-2,2-dimethylpropylidene)-4-methylbenzenesulfonamide (1n)

White solid, mp: 176−178 °C; ^{1}H NMR (400 MHz, $CDCl_3$): δ 7.66 (d, J = 8.0 Hz, 2H), 7.31 (d, J = 8.4 Hz, 2H), 7.21 (d, J = 8.0 Hz, 2H), 7.03 (d, J = 8.4 Hz, 2H), 2.38 (s, 3H), 1.15 (s, 9H); ^{13}C NMR (100 MHz, $CDCl_3$): δ 191.9, 143.5, 138.0, 135.1, 133.7, 129.3, 127.8, 127.7, 127.2, 43.0, 27.7, 21.6; IR (KBr) (v/cm^{-1}): 2977, 1603, 1587, 1150, 1091, 983, 839, 669; HRMS (ESI-MS) Calcd. For $C_{18}H_{21}ClNO_2S$ $[M+H]^+$ 350.0982, found: 350.0990.

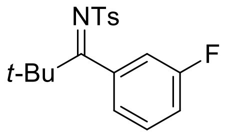

(Z)-N-(1-(3-Fluorophenyl)-2, 2-dimethylpropylidene)-4-methylbenzenesulfonamide (1o)

White solid, mp: 162−165 °C; ^{1}H NMR (400 MHz, $CDCl_3$): δ 7.67 (d, J = 8.0 Hz, 2H), 7.37−7.32 (m, 1H), 7.26−7.22 (m, 2H), 7.11−7.06 (m, 1H), 6.91 (d, J = 7.6 Hz, 1H), 6.78 (d, J = 8.8 Hz, 1H), 2.40 (s, 3H), 1.17 (s, 9H); ^{13}C NMR (100 MHz, $CDCl_3$): δ 191.1, 162.9, 160.5, 137.9, 137.1, 137.0 (d, J =7.0 Hz), 129.3 (t, J = 3.8 Hz), 127.3, 122.4 (d, J = 3.6 Hz), 116.0 (d, J = 3.6 Hz), 113.8 (d, J = 23.4 Hz), 43.0, 27.8, 21.6; ^{19}F NMR (376 MHz, $CDCl_3$): -112.4; IR (KBr) (v/cm^{-1}):2972, 1615, 1596, 815, 802, 786; HRMS (ESI-MS) Calcd. For $C_{18}H_{22}NO_2S$ $[M+H]^+$334.1266, found: 334.1270.

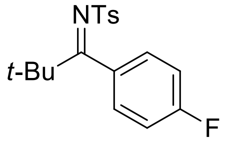

(Z)-N-(1-(4-Fluorophenyl)-2, 2-dimethylpropylidene)-4-methylbenzenesulfonamide (1p)

White solid, mp: 133−135 °C; ^{1}H NMR (400 MHz, $CDCl_3$): δ 7.68 (d, J = 8.4 Hz, 2H), 7.23 (d, J = 8.0 Hz, 2H), 7.10−7.02 (m, 4H), 2.39 (s, 3H), 1.16(s, 9H); ^{13}C NMR (100 MHz, $CDCl_3$): δ 192.4, 164.0, 161.5, 143.4, 138.1, 129.3, 128.4 (d, J = 8.2 Hz), 127.1, 114.8 (d, J = 21.7 Hz), 43.1, 27.8, 21.6; ^{19}F NMR(376 MHz, $CDCl_3$): -111.5; IR (KBr) (v/cm^{-1}): 2977, 2874, 1606, 1592, 847, 836, 818; HRMS (ESI-MS) Calcd. For $C_{18}H_{21}NO_2S$ $[M+H]^+$ 334.1287, found: 334.1290.

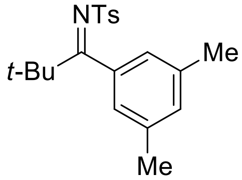

(Z)-N-(1-(3, 5-Dimethylphenyl)-2,2-dimethylpropylidene)-4-methylbenzenesulfonamide (1q)

White solid, mp: 134−136 °C; ^{1}H NMR (400 MHz, $CDCl_3$): δ 7.55 (d, J = 8.4 Hz, 2H), 7.15 (d, J = 8.0 Hz, 2H), 6.94(s, 1H), 6.58 (s, 2H), 2.37 (s, 3H), 2.26 (s, 6H), 1.17 (s, 9H); ^{13}C NMR (100 MHz, $CDCl_3$): δ 193.7, 143.0, 138.1, 136.8, 135.0, 130.3, 129.1, 127.3, 123.9, 43.0, 28.0, 21.5, 21.3; IR (KBr) (v/cm^{-1}): 3034, 2965, 2918, 1596, 1492, 848, 810; HRMS (ESI-MS) Calcd. For $C_{20}H_{25}NO_2SNa$ $[M+Na]^+$ 366.1489, found:

366.1504.

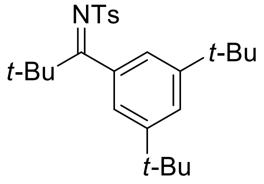

(*Z*)-*N*-(1-(3,5-Di-tert-butylphenyl)-2,2-dimethylpropylidene)-4-methylbenzenesulfonamide (1r)

White solid, mp: 112−114 °C; ^{1}H NMR (400 MHz, $CDCl_3$): δ 7.51 (d, J = 8.4 Hz, 2H), 7.33 (t, J = 3.6 Hz, 1H), 7.10 (d, J = 8.4 Hz, 2H), 6.80 (d, J = 2.0 Hz, 2H), 2.35 (s, 3H), 1.28 (s, 18H), 1.17 (s, 9H); ^{13}C NMR (100 MHz, $CDCl_3$): δ 194.5, 149.5, 142.8, 138.2, 134.2, 129.1, 127.3, 122.3, 120.9, 43.1, 34.8, 31.3, 28.0, 21.6; IR (KBr) (v/cm^{-1}): 2964, 2869, 1610, 1591, 884, 857; HRMS (ESI-MS) Calcd. For $C_{26}H_{38}NO_2S$ $[M+H]^+$428.2616, found: 428.2623.

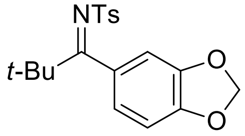

(*Z*)-*N*-(1-(Benzo[*d*][1,3]dioxol-5-yl)-2,2-dimethylpropylidene)-4-methylbenzenesulfonamide (1s)

White solid, mp: 145−147 °C; ^{1}H NMR (400 MHz, $CDCl_3$): δ 7.68 (d, J = 8.0 Hz, 2H), 7.23 (d, J = 8.0 Hz, 2H), 6.79 (d, J = 8.0 Hz, 1H), 6.60 (d, J = 8.0 Hz, 1H), 6.53 (s, 1H), 5.98 (s, 2H), 2.40 (s, 3H), 1.18 (s, 9H); ^{13}C NMR (100 MHz, $CDCl_3$): δ 192.6, 148.0, 146.8, 143.2, 138.3, 129.2, 128.8, 127.2, 120.5, 107.6, 107.1, 101.3, 43.1, 28.0, 21.5; IR (KBr) (v/cm^{-1}): 2967, 1611, 1489, 1314, 1149, 1091, 900, 831, 773; HRMS (ESI-MS) Calcd. For $C_{19}H_{22}NO_4S$ $[M+H]^+$ 360.1270, found: 360.1270.

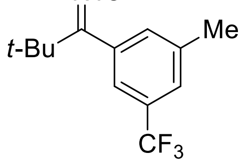

(*Z*)-*N*-(2,2-Dimethyl-1-(3-methyl-5-(trifluoromethyl)phenyl)propylidene)-4-methylbenzenesu lfonamide (1t)

White solid, mp: 124−126 °C; ^{1}H NMR (400 MHz, $CDCl_3$): δ 7.65 (d, J = 8.4 Hz, 2H),

7.22 (d, J = 8.0 Hz, 2H), 6.88 (d, J = 9.6 Hz, 1H), 6.64 (d, J = 9.6 Hz, 1H), 6.59 (d, J = 8.4 Hz, 1H), 2.41 (s, 3H), 2.34 (s, 3H), 1.17 (s, 9H); ^{13}C NMR (100 MHz, $CDCl_3$): δ 191.5 (d, J = 1.6 Hz), 162.9, 160.4, 143.5, 139.9 (d, J = 8.0 Hz), 138.0, 136.6 (d, J = 7.9 Hz), 129.3, 127.3, 122.8 (d, J = 2.9 Hz), 116.5 (d, J = 20.6 Hz), 110.8 (d, J = 23.6 Hz), 42.3, 27.9, 21.5;, 21.4 (d, J = 1.6 Hz) ^{19}F NMR (376 MHz, $CDCl_3$): -113.7 (d, J = 0.75 Hz); IR (KBr) (ν/cm^{-1}): 2971, 1617, 1603, 814, 797, 761; HRMS (ESI-MS) Calcd. For $C_{20}H_{22}F_3NO_2SNa$ $[M+Na]^+$ 420.1799, found: 420.1796.

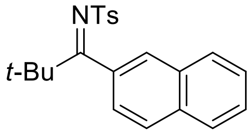

(*Z*)-*N*-(2, 2-Dimethyl-1-(naphthalen-2-yl)propylidene)-4-methylbenzenesulfonamide (1u)

White solid, mp: 136−138 °C; ^{1}H NMR (400 MHz, $CDCl_3$): δ 7.86−7.78 (m, 3H), 7.60 (d, J = 8.0 Hz, 2H), 7.53−7.50 (m, 3H), 7.22 (d, J = 9.6 Hz, 1H), 7.07 (d, J = 8.0 Hz, 2H), 2.29 (s, 3H), 1.26 (s, 9H); ^{13}C NMR (100 MHz, $CDCl_3$): δ 193.1, 143.3, 138.0, 132.9, 132.7, 131.9, 129.2, 128.4, 127.8, 127.3, 127.1, 127.0, 126.7, 125.7, 123.9, 43.3, 28.0, 21.5; IR (KBr) (ν/cm^{-1}): 3057, 2973, 2931, 1607, 1594, 857, 810; HRMS (ESI-MS) Calcd. For $C_{22}H_{24}NO_2S$ $[M+H]^+$ 366.1525, found: 366.1528.

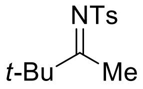

(*E*)-*N*-(3, 3-Dimethylbutan-2-ylidene)-4-methylbenzenesulfonamide (1v)[13]

White solid, ^{1}H NMR (400 MHz, $CDCl_3$): δ 7.83 (d, J = 8.0 Hz, 2H), 7.29 (d, J = 8.0 Hz, 2H), 2.52 (s, 3H), 2.41 (s, 3H), 1.13(s, 9H).

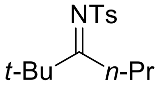

(*E*)-4-Methyl-N-(pentan-2-ylidene)benzenesulfonamide (1w)

White solid, mp: 74−75 °C; ^{1}H NMR (400 MHz, $CDCl_3$): δ 7.85 (d, J = 7.6 Hz, 2H), 7.30 (d, J = 7.8 Hz, 2H), 2.88–2.84 (m, 2H), 2.42 (s, 3H), 1.83–1.75 (m, 2H), 1.16 (s, 9H), 1.07 (q, J = 7.2 Hz, 3H); ^{13}C NMR (100 MHz, $CDCl_3$): δ 197.8, 143.1, 138.9, 129.3, 126.9, 43.5, 35.4, 27.5, 22.5, 21.6, 15.1; IR (KBr) (ν/cm^{-1}): 2969, 2932, 2873, 1602, 1478, 1317, 1155, 1093, 815, 754, 583; HRMS (ESI-MS) Calcd. For

$C_{15}H_{24}NO_2S$ [M+H]$^+$ 282.1528, found: 282.1539.

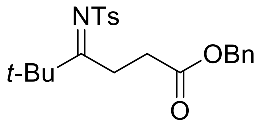

(*E*)-Benzyl 5,5-dimethyl-4-(tosylimino)hexanoate (1x)

White solid, mp: 90−91 °C; ^{1}H NMR (400 MHz, CDCl$_3$): δ 7.83 (d, J = 8.0 Hz, 2H), 7.35–7.31 (m, 5H), 7.29 (d, J = 8.4 Hz, 2H), 5.14 (s, 3H), 3.23 (t, J = 8.4 Hz, 2H), 2.86 (t, J = 8.4 Hz, 2H), 2.41 (s, 3H), 1.15 (s, 9H); ^{13}C NMR (100 MHz, CDCl$_3$): δ 195.3, 171.5, 143.4, 138.5, 135.7, 129.3, 128.5, 128.3, 128.2, 127.0, 66.7, 43.8, 32.1, 27.6, 27.3, 21.5; IR (KBr) (v/cm^{-1}): 2922, 2359, 1737, 1608, 1315, 1303, 1153, 748, 661; HRMS (ESI-MS) Calcd. For $C_{22}H_{28}NO_4S$ [M+H]$^+$ 402.1739, found: 402.1744.

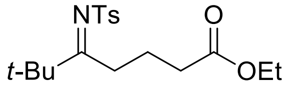

(*E*)-Ethyl 6,6-dimethyl-5-(tosylimino)heptanoate (1y)

White solid, mp: 41−42 °C; ^{1}H NMR (400 MHz, CDCl$_3$): δ 7.83 (d, J = 8.0 Hz, 2H), 7.29 (d, J = 8.4 Hz, 2H), 4.14 (q, J = 7.2 Hz, 2H), 2.93 (t, J = 8.0 Hz, 2H), 2.47 (t, J = 6.8 Hz, 2H), 2.41 (s, 3H), 2.11–2.03 (m, 2H), 1.26 (t, J = 7.2 Hz, 3H), 1.16 (s, 9H); ^{13}C NMR (100 MHz, CDCl$_3$): δ 196.9, 172.9, 143.2, 138.7, 129.3, 126.9, 60.5, 43.6, 34.4, 32.5, 27.5, 23.8, 21.6, 14.2; IR (KBr) (v/cm^{-1}): 2973, 2359, 1732, 1608, 1315, 1154, 1092, 814, 749, 582; HRMS (ESI-MS) Calcd. For $C_{18}H_{28}NO_4S$ [M+H]$^+$ 354.1739, found: 354.1744.

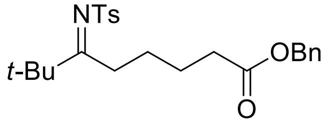

(*E*)-Benzyl 7,7-dimethyl-6-(tosylimino)octanoate (1z)

Light yellow liquid; ^{1}H NMR (400 MHz, CDCl$_3$): δ 7.82 (d, J = 8.0 Hz, 2H), 7.33–7.30 (m, 5H), 7.28 (d, J = 8.0 Hz, 2H), 5.10 (s, 2H), 2.87 (t, J = 7.6 Hz, 2H), 2.44–2.37 (m, 5H), 1.82–1.76 (m, 4H) 1.11 (s, 9H); ^{13}C NMR (100 MHz, CDCl$_3$): δ 197.2, 173.1, 143.1, 138.9, 136.0, 129.3, 128.5, 128.2, 128.1, 126.9, 66.2, 43.5, 33.6, 32.9, 28.0, 27.5, 25.5, 21.5; IR (KBr) (v/cm^{-1}): 2969, 1735, 1607, 1314, 1153, 1092, 750, 582, 553; HRMS (ESI-MS) Calcd. For $C_{24}H_{32}NO_4S$ [M+H]$^+$ 430.2052, found: 430.2049.

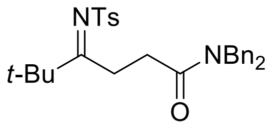

(*E*)-*N,N*-Dibenzyl-5,5-dimethyl-4-(tosylimino)hexanamide (1aa)

White solid, mp: 112−113 °C; ^{1}H NMR (400 MHz, $CDCl_3$): δ 7.79 (d, J = 8.0 Hz, 2H), 7.36–7.21 (m, 10H), 7.17 (d, J = 8.4 Hz, 2H), 4.61 (s, 2H), 4.51 (s, 2H), 3.33 (t, J = 8.0 Hz, 2H), 2.91 (t, J = 8.0 Hz, 2H), 2.39 (s, 3H), 1.13 (s, 9H); ^{13}C NMR (100 MHz, $CDCl_3$): δ 196.7, 171.3, 143.3, 138.5, 137.1, 136.4, 129.3, 128.9, 128.6, 128.2, 127.6, 127.4, 126.9, 126.6, 49.9, 48.6, 43.9, 31.5, 28.5, 27.3, 21.5; IR (KBr) (v/cm^{-1}): 2922, 2852, 2360, 1651, 1607, 1452, 1384, 1152, 750, 698, 582; HRMS (ESI-MS) Calcd. For $C_{29}H_{35}N_2O_3S$ $[M+H]^+$ 491.2368, found: 491.2371.

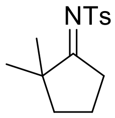

(*E*)-*N*-(2,2-Dimethylcyclopentylidene)-4-methylbenzenesulfonamide (1ab)

White solid, mp: 68−69 °C; ^{1}H NMR (400 MHz, $CDCl_3$): δ 7.82 (d, J = 8.0 Hz, 2H), 7.29 (d, J = 8.0 Hz, 2H), 2.99 (t, J = 7.6 Hz, 2H), 2.40 (s, 3H), 1.91–1.83 (m, 2H), 1.67 (t, J = 6.8 Hz, 2H), 1.07 (s, 6H); ^{13}C NMR (100 MHz, $CDCl_3$): δ 204.3, 143.5, 137.9, 129.4, 127.1, 47.3, 38.4, 34.1, 25.5, 21.6, 21.1; IR (KBr) (v/cm^{-1}): 2963, 2868, 1629, 1316, 1304, 1178, 1093, 854, 771, 666, 577; HRMS (ESI-MS) Calcd. For $C_{14}H_{20}NO_2S$ $[M+H]^+$ 266.1215, found: 266.1213.

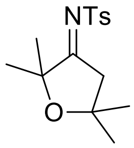

(*E*)-4-Methyl-N-(2,2,5,5-tetramethyldihydrofuran-3(2H)-ylidene)benzenesulfonamide (1ac)

White solid, mp: 134−135 °C; ^{1}H NMR (400 MHz, $CDCl_3$): δ 7.81 (d, J = 8.0 Hz, 2H), 7.31 (d, J = 8.0 Hz, 2H), 3.20 (s, 2H), 2.42 (s, 3H), 1.33 (t, J = 7.2 Hz, 12H); ^{13}C NMR (100 MHz, $CDCl_3$): δ 198.2, 144.1, 137.0, 129.6, 127.3, 83.7, 79.3, 45.4, 29.9, 28.4, 21.6; IR (KBr) (v/cm^{-1}): 2927, 2359, 1640, 1318, 1159, 1091, 996, 913, 828, 669, 512; HRMS (ESI-MS) Calcd. For $C_{15}H_{22}NO_3S$ $[M+H]^+$ 296.1320, found: 296.1332.

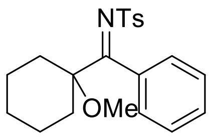

(*E*)-*N*-((1-Methoxycyclohexyl)(phenyl)methylene)-4-methylbenzenesulfonamide (1ad)

White solid, mp: 96−98 °C; ^{1}H NMR (400 MHz, $CDCl_3$): δ 7.67 (d, J = 8.0 Hz, 2H), 7.42–7.28 (m, 5H), 7.21 (d, J = 8.0 Hz, 2H), 3.16 (s, 3H), 2.38 (s, 3H), 1.78–1.23 (m, 10H); ^{13}C NMR (100 MHz, $CDCl_3$): δ 186.3, 143.4, 138.2, 134.7, 129.7, 129.3, 127.5, 127.2, 127.0, 81.4, 50.6, 32.2, 25.1, 21.7, 21.5; IR (KBr) (v/cm^{-1}): 2929, 2855, 2359, 1595, 1443, 1327, 1158, 1090, 814, 784, 709, 674, 572, 557; HRMS (ESI-MS) Calcd. For $C_{21}H_{26}NO_3S$ $[M+H]^+$ 372.1633, found: 372.1645.

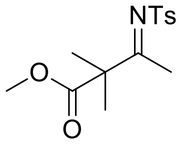

(*E*)-Methyl 2,2-dimethyl-3-(tosylimino)butanoate (1ae)

White solid, mp: 58−59 °C; ^{1}H NMR (400 MHz, $CDCl_3$): δ 7.82 (d, J = 8.0 Hz, 2H), 7.30 (d, J = 8.0 Hz, 2H), 3.67 (s, 3H), 2.48 (s, 3H), 2.41 (s, 3H), 1.34 (s, 6H); ^{13}C NMR (100 MHz, $CDCl_3$): δ 187.7, 173.9, 143.6, 138.1, 129.4, 127.0, 54.6, 52.7, 22.9, 21.6, 20.9; IR (KBr) (v/cm^{-1}): 2986, 2952, 1741, 1622, 1318, 1091, 815, 718, 694, 553; HRMS (ESI-MS) Calcd. For $C_{14}H_{20}NO_4S$ $[M+H]^+$ 298.1113, found: 298.1118.

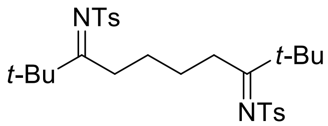

(*E*)-*N,N'*-(2,2,9,9-Tetramethyldecane-3,8-diylidene)bis(4-methylbenzenesulfonamide) (1af)

White solid, mp: 192−193 °C; ^{1}H NMR (400 MHz, $CDCl_3$): δ 7.83 (d, J = 8.0 Hz, 4H), 7.28 (d, J = 8.0 Hz, 4H), 3.00–2.91 (m, 4H), 2.40 (s, 6H), 1.95–1.87 (m, 4H), 1.15 (s, 18H); ^{13}C NMR (100 MHz, $CDCl_3$): δ 197.5, 143.1, 138.9, 129.3, 126.9, 43.56, 32.6, 29.2, 27.5, 21.5; IR (KBr) (v/cm^{-1}): 3033, 2969, 2872, 1735, 1608, 1478, 1456, 1315, 1154, 1092, 668, 814, 752, 671, 583, 553; HRMS (ESI-MS) Calcd. For $C_{28}H_{41}N_2O_4S_2$ $[M+H]^+$ 533.2508, found: 533.2519.

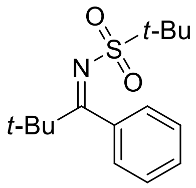

(Z)-*N*-(2,2-Dimethyl-1-phenylpropylidene)-2-methylpropane-2-sulfonamide (1ag)

White solid, mp: 118−120 °C; ^{1}H NMR (400 MHz, $CDCl_3$): δ 7.41–7.34 (m, 3H), 7.20–7.14 (m, 2H), 1.43 (s, 9H), 1.22 (s, 9H); ^{13}C NMR (100 MHz, $CDCl_3$): δ 194.5, 135.7, 128.9, 127.3, 126.3, 59.0, 43.2, 27.9, 23.9; IR (KBr) (ν/cm^{-1}): 3055, 2982, 2933, 2871, 1614, 1479, 1440, 1363, 1305, 1197, 1124, 983, 911, 841, 810, 791, 706, 694,519; HRMS (ESI-MS) Calcd. For $C_{15}H_{24}NO_2S$ $[M+H]^+$ 282.1528, found: 282.1526.

Asymmetric hydrogenation of *N*-tosylimines

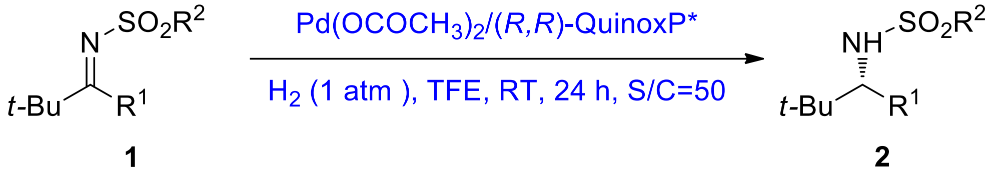

Procedure H :(*R*, *R)*-QuinoxP* (1.4 mg, 2.1 mol%) and $Pd(OAc)_2$ (0.89 mg, 2.0 mol%) were placed in a dried Schlenk tube under nitrogen atmosphere, and degassed anhydrous acetone (1.0 mL) was added. The mixture was stirred at room temperature for 5 min, then the solvent was removed under vacuum to give the dry catalyst. In a glovebox, substrate **1** (0.2 mmol) was stirred in a solvent (0.5 mL) at room temperature for 10 min. Subsequently, the above catalyst together with a mixed solvent (1.5 mL) was added to the reaction mixture. The hydrogenation was performed at room temperature under H_2 (1 bar) in a stainless steel autoclave for 24 h. After carefully releasing the hydrogen, the conversion of the product **2** was determined by ^{1}H NMR spectroscopic analysis of the crude reaction mixture. The enantiomeric excesses of the products were determined by HPLC with chiral columns (OD-H, OJ-H, AD-H or IC-3).

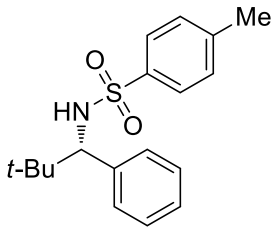

(*S*)-*N*-(2,2-Dimethyl-1-phenylpropyl)-4-methylbenzenesulfonamide (2a)[14]

White solid, 99% yield, 99.9% ee; ^{1}H NMR (400 MHz, $CDCl_3$): δ 7.40 (d, J = 8.4 Hz, 2H), 7.06−7.04 (m, 3H), 6.96 (d, J = 8.0 Hz, 2H), 6.88−6.86 (m, 2H), 5.69 (d, J = 8.0 Hz, 1H), 4.02 (d, J = 8.0 Hz, 1H), 2.27 (s, 3H), 0.89 (s, 9H), HPLC conditions: DAICEL Chiralpak OJ column, Hexane/*i*-PrOH = 70/30, 220 nm, 0.7 mL/min, t_{major} =7.8 min, t_{minor} = 15.2 min.

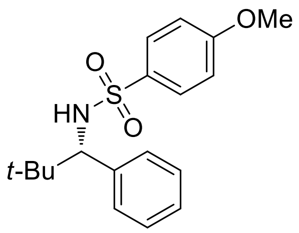

(*S*)-*N*-(2, 2-Dimethyl-1-phenylpropyl)-4-methoxybenzenesulfonamide (2b)

White solid, 96% yield, 99.0% ee; ^{1}H NMR (400 MHz, $CDCl_3$): δ 7.43 (d, J = 7.2 Hz, 2H), 7.07−7.05(m, 3H), 6.89−6.87 (m, 2H), 6.63 (d, J = 8.8 Hz, 2H), 5.16 (d, J = 8.4 Hz, 1H), 4.01 (d, J = 8.8 Hz, 1H), 3.75 (s, 3H), 0.89 (s, 9H); HPLC conditions: DAICEL Chiralpak OJ column, Hexane/*i*-PrOH = 70/30, 230 nm, 1.0 mL/min, t_{major} = 6.7 min, t_{minor}= 15.0 min.

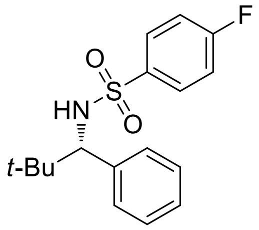

(*S*)-*N*-(2, 2-Dimethyl-1-phenylpropyl)-4-fluorobenzenesulfonamide (2c)

White solid, 96.0% yield, 96% ee; ^{1}H NMR (400 MHz, $CDCl_3$): δ 7.52−7.49 (m, 2H), 7.07−7.05 (m, 3H), 6.87−6.79 (m, 4H), 5.41(d, J = 8.4 Hz, 1H), 4.06 (d, J = 8.4 Hz, 1H), 0.92 (s, 9H); HPLC conditions: DAICEL Chiralpak OJ column, Hexane/*i*-PrOH = 70/30, 230 nm, 1.0 mL/min, t_{major}= 5.0 min, t_{minor}= 13.0 min.

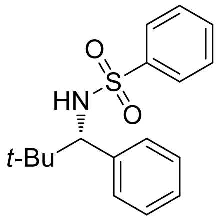

(*S*)-*N*-(2,2-Dimethyl-1-phenylpropyl)benzenesulfonamide (2d)

White solid, 99% yield, 99.9% ee, mp: 153−155 °C; ^{1}H NMR (400 MHz, $CDCl_3$): δ 7.55 (d, J = 7.6 Hz, 2H), 7.30 (t, J = 7.6 Hz, 1H), 7.17 (t, J = 8.0 Hz, 2H), 7.04–7.01 (m, 3H), 6.89–6.87 (m, 2H), 5.64(d, J = 9.6 Hz, 1H), 3.06 (d, J = 9.6 Hz, 1H), 0.91 (s, 9H); ^{13}C NMR (100 MHz, $CDCl_3$): δ 140.1, 138.0, 131.9, 128.4, 128.0, 127.5, 126.9, 126.8, 67.0, 35.3, 26.6; HPLC conditions: DAICEL Chiralpak OJ column, Hexane/*i*-PrOH = 95/5, 220 nm, 1.0 mL/min, t_{major}=19.5 min, t_{minor}=44.1 min; $[\alpha]_D^{20}$ =

–43.7 (c 0.11, CH_2Cl_2); IR (KBr) (v/cm^{-1}): 3327, 2970, 1327, 1313, 1158, 756, 717, 591; HRMS (ESI-MS) Calcd. For $C_{17}H_{22}NO_2S$ $[M+H]^+$ 304.1371, found: 304.1373.

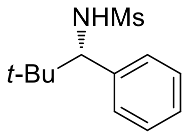

(*S*)-*N*-(2,2-Dimethyl-1-phenylpropyl)methanesulfonamide (2e)

White solid, mp: 133−135 °C; 98% yield, 99.1% ee; ^{1}H NMR (400 MHz, $CDCl_3$): δ 7.34−7.22 (m, 5H), 5.72−5.65 (m, 1H), 4.17 (d, J = 9.6 Hz, 1H), 2.46 (s, 3H), 0.94 (m, 9H); ^{13}C NMR (100 MHz, $CDCl_3$): δ 139.1, 128.2, 128.1, 127.6, 66.8, 41.4, 35.2, 26.7; HPLC conditions: DAICEL Chiralpak IC-3 column, Hexane/*i*-PrOH = 80/20, 210 nm, 0.6 mL/min, t_{minor}= 16.8 min, t_{major} = 28.4 min; $[\alpha]_D^{20}$ = –24.9 (c 0.28, CH_2Cl_2); IR (KBr) (v/cm^{-1}): 2971, 2933, 2870, 1616, 1597, 1319, 1200, 1147, 984, 967, 843, 803, 703, 546, 521; HRMS (ESI-MS) Calcd. For $C_{12}H_{19}NNaO_2S$ $[M+Na]^+$ 264.1034, found: 264.1047.

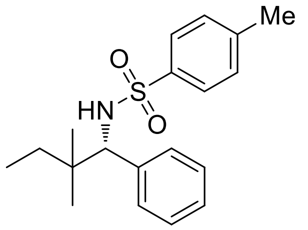

(*S*)-*N*-(2,2-Dimethyl-1-phenylbutyl)-4-methylbenzenesulfonamide (2f)

White solid, 96% yield, 99.9% ee, mp: 123−125 °C; ^{1}H NMR (400 MHz, $CDCl_3$): δ 7.42 (d, J = 8.0 Hz, 2H), 7.04–6.99 (m, 3H), 6.94–6.88 (m, 4H), 5.76(d, J = 9.6 Hz, 1H), 4.11 (d, J = 9.6 Hz, 1H), 2.26 (s, 3H), 1.34–1.31 (m, 2H) 0.89 (s, 3H), 0.85 (t, J = 7.2 Hz, 3H), 0.77 (s, 3H); ^{13}C NMR (100 MHz, $CDCl_3$): δ 142.5, 138.1, 137.2, 128.9, 128.3, 127.4, 127.0, 126.5, 65.5, 37.9, 31.6, 23.0, 22.8, 21.4, 8.1; HPLC conditions: DAICEL Chiralpak OJ column, Hexane/*i*-PrOH = 90/10, 220 nm, 0.5 mL/min, t_{major}= 16.1 min, t_{minor}= 29.6 min; $[\alpha]_D^{20}$ = –27.3 (c 0.14, CH_2Cl_2); IR (KBr) (v/cm^{-1}): 3284, 2967, 1472, 1319, 1161, 1088, 810, 705, 670; HRMS (ESI-MS) Calcd. For $C_{19}H_{26}NO_2S$ $[M+H]^+$ 332.1684, found: 332.1689.

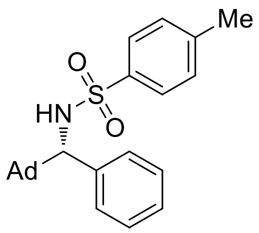

(*S*)-*N*-(Adamantan-1-yl(phenyl)methyl)-4-methylbenzenesulfonamide (2g)

White solid, 96% yield, 99.9% ee, mp: 206−208 °C; ^{1}H NMR (400 MHz, $CDCl_3$): δ 7.38 (d, J = 8.0 Hz, 2H), 7.08–7.02 (m, 3H), 6.95 (d, J = 8.4 Hz, 2H), 6.82 (d, J = 7.6 Hz, 2H), 5.24 (d, J = 9.2 Hz, 1H), 3.85 (d, J = 9.6 Hz, 1H), 2.27 (s, 3H), 1.95 (s, 3H), 1.66–1.61 (m, 6H), 1.54–1.50 (m, 3H), 1.34 (d, J = 12.0 Hz, 3H); ^{13}C NMR (100 MHz, $CDCl_3$): δ 142.5, 137.3, 137.2, 128.9, 128.2, 127.4, 126.9, 126.7, 67.6, 38.7, 36.6, 36.5, 28.2, 21.4; HPLC conditions: DAICEL Chiralpak OD column, Hexane/*i*-PrOH = 90/10, 220 nm, 0.8 mL/min, t_{minor}=7.1 min, t_{major}=9.8 min; $[\alpha]_D^{20}$ = –15.70 (*c* 0.53, CH_2Cl_2); IR (KBr) (v/cm^{-1}): 3276, 2903, 2848, 1455, 1320, 1159, 811, 703, 673; HRMS (ESI-MS) Calcd. For $C_{24}H_{30}NO_2S$ $[M+H]^+$ 396.1997, found: 396.2000.

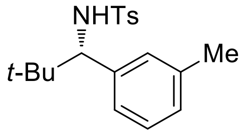

(*S*)-*N*-(2, 2-Dimethyl-1-(m-tolyl)propyl)-4-methylbenzenesulfonamide (2h)

White solid, 96% yield, 99.9% ee, mp: 131−133 °C; ^{1}H NMR (400 MHz, $CDCl_3$): δ 7.40 (d, J = 8.4 Hz, 2H), 6.98–6.95 (m, 3H), 6.85 (d, J = 7.2 Hz, 1H), 6.72 (d, J = 7.6 Hz, 1H), 6.56 (s, 1H), 5.33(d, J = 9.6 Hz, 1H), 3.97 (d, J = 9.6 Hz, 1H), 2.27 (s, 3H), 2.12 (s, 3H), 0.89 (s, 9H); ^{13}C NMR (100 MHz, $CDCl_3$): δ 142.4, 138.1, 137.3, 136.8, 128.9, 128.8, 127.4, 127.3, 127.0, 125.3, 67.1, 35.2, 26.7, 21.3, 21.2; HPLC conditions: DAICEL Chiralpak OD column, Hexane/*i*-PrOH = 93/7, 220 nm, 1.0 mL/min, t_{major} =7.2 min, t_{minor} = 8.2 min; $[\alpha]_D^{20}$ = –21.5 (*c* 0.37, CH_2Cl_2); IR (KBr) (v/cm^{-1}): 3279, 2971, 1791, 1717, 1749, 810, 699, 690; HRMS (ESI-MS) Calcd. For $C_{19}H_{25}NO_2SNa$ $[M+Na]^+$ 354.1504, found: 354.1504.

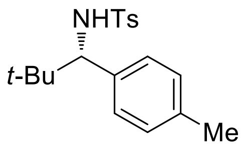

(*S*)-*N*-(2, 2-Dimethyl-1-(p-tolyl) propyl)-4-methylbenzenesulfonamide (2i)

White solid, 96% yield, 99.9% ee, mp: 172−174 °C; ^{1}H NMR (400 MHz, $CDCl_3$): δ 7.39 (d, J = 6.8 Hz, 2H), 6.97 (d, J = 7.2 Hz, 2H), 6.85 (d, J = 7.6 Hz, 2H), 6.74 (d, J = 7.8 Hz, 2H), 4.94 (d, J = 9.2 Hz, 1H), 3.97 (d, J = 9.2 Hz, 1H), 2.29 (s, 3H), 2.24 (s, 3H), 0.87 (s, 9H); ^{13}C NMR (100 MHz, $CDCl_3$): δ 142.5, 137.3, 136.4, 135.3, 128.9, 128.1, 128.0, 127.1, 66.8, 35.2, 26.7, 21.4, 20.9; HPLC conditions: DAICEL Chiralpak OD column, Hexane/*i*-PrOH = 93/7, 220 nm, 1.0 mL/min, t_{major}=8.0 min, t_{minor} = 8.7 min; $[\alpha]_D^{20}$ = –34.0 (*c* 0.36, CH_2Cl_2); IR (KBr) (v/cm^{-1}): 3272, 2965, 2920, 1520, 1470, 1456, 808, 778, 718; HRMS (ESI-MS) Calcd. For $C_{19}H_{25}NO_2SNa$ $[M+Na]^+$ 354.1504, found: 354.1504.

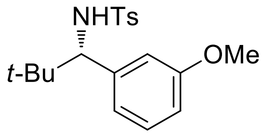

(*S*)-*N*-(1-(3-Methoxyphenyl)-2, 2-dimethylpropylidene)-4-methylbenzenesulfonamide (2g)

White solid, 98% yield, 99.9% ee, mp: 133−135 °C; ^{1}H NMR (400 MHz, $CDCl_3$): δ 7.42 (d, J = 7.6 Hz, 2H), 7.01–7.05 (m, 3H), 6.59 (d, J = 8.4 Hz, 1H), 6.51 (d, J = 7.6 Hz, 1H), 6.35 (s, 1H), 5.50 (d, J = 9.2 Hz, 1H), 3.97 (d, J = 9.2 Hz, 1H), 3.65 (s, 3H), 2.28 (s, 3H), 0.91 (s, 9H); ^{13}C NMR (100 MHz, $CDCl_3$): δ 158.7, 142.6, 139.9, 137.2, 128.9, 128.5, 127.1, 120.7, 113.9, 112.1, 67.0, 55.0, 35.3, 26.8, 21.4; HPLC conditions: DAICEL Chiralpak OJ column, Hexane/*i*-PrOH = 93/7, 220 nm, 1.0 mL/min, t_{major}=11.5 min, t_{minor} = 16.2 min; $[\alpha]_D^{20}$ = –46.1 (*c* 0.42, CH_2Cl_2); IR (KBr) (v/cm^{-1}): 2965, 1558, 1515, 840, 809; HRMS (ESI-MS) Calcd. For $C_{19}H_{25}NO_3SNa$ $[M+Na]^+$ 370.1287, found: 370.1289.

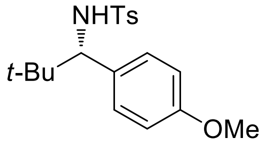

(*S*)-*N*-(1-(4-Methoxyphenyl)-2, 2-dimethylpropyl)-4-methylbenzenesulfonamide(2k)

White solid, 97% yield, 99.9% ee, mp: 117−119 °C; ^{1}H NMR (400 MHz, $CDCl_3$): δ

7.42 (d, J = 8.4 Hz, 2H), 6.99 (d, J = 7.6 Hz, 2H), 6.80 (d, J = 8.8 Hz, 2H), 6.57 (d, J = 8.8 Hz, 2H), 5.31 (d, J = 9.2 Hz, 1H), 3.97 (d, J = 9.2 Hz, 1H), 3.72 (s, 3H), 2.29 (s, 3H), 0.88 (s, 9H); ^{13}C NMR (100 MHz, $CDCl_3$) *δ* 158.3, 142.5, 137.2, 130.5, 129.0, 128.9, 127.0, 112.8, 66.4, 55.2, 35.3, 26.6, 21.4; HPLC conditions: DAICEL Chiralpak OD column, Hexane/*i*-PrOH = 93/7, 220 nm, 1.0 mL/min, t_{major} =10.2 min, t_{minor} = 12.6 min; $[\alpha]_D^{20}$ = –43.5 (*c* 0.42, CH_2Cl_2); IR (KBr) (v/cm^{-1}): 2964, 1868, 1844, 1791, 813, 668; HRMS (ESI-MS) Calcd. For $C_{19}H_{25}NO_3SNa$ $[M+Na]^+$ 370.1442, found: 370.1453.

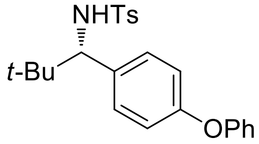

(*S*)-*N*-(2, 2-Dimethyl-1-(4-phenoxyphenyl)propyl)-4-methylbenzenesulfonamide (2l)

White solid, 99% yield, 99.9% ee, mp: 121−123 °C; ^{1}H NMR (400 MHz, $CDCl_3$): δ 7.47(d, J = 8.4 Hz, 2H), 7.34−7.31 (m, 2H), 7.12−7.08(m, 1H), 7.04 (d, J = 8.0 Hz, 2H), 6.91 (dd, J = 1.2 Hz, 8.8 Hz, 2H), 6.85 (d, J = 8.4 Hz, 2H), 6.69 (d, J = 8.4 Hz, 2H), 5.38 (d, J = 8.8 Hz, 1H), 4.0 (d, J = 9.2 Hz, 1H), 2.33 (s, 3H), 0.90 (s, 9H); ^{13}C NMR (100 MHz, $CDCl_3$): δ 157.1, 155.9, 142.6, 137.5, 133.4, 129.7, 129.5, 129.0, 127.2, 123.3, 118.7, 117.8, 114.3 (d, J = 2.1Hz), 66.5, 35.4, 26.7, 21.5; HPLC conditions: DAICEL Chiralpak OJ column, Hexane/*i*-PrOH = 96/4, 220 nm, 1.0 mL/min, t_{major} =21.9 min, t_{minor} = 26.4 min; $[\alpha]_D^{20}$ = –25.6 (*c* 0.34, CH_2Cl_2), IR (KBr) (v/cm^{-1}): 3281, 2964, 2871, 1791, 1749, 1683, 848, 812,749; HRMS (ESI-MS) Calcd. For $C_{24}H_{28}NO_3S$ $[M+H]^+$ 410.1796, found: 410.1790.

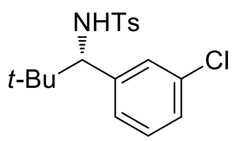

(*S*)-*N*-(1-(3-Chlorophenyl)-2,2-dimethylpropyl)-4-methylbenzenesulfonamide (2m)

White solid, 96% yield, 99.8% ee, mp: 120−122 °C; ^{1}H NMR (400 MHz, $CDCl_3$): δ 7.46 (d, J = 8.8 Hz, 2H), 7.03−6.97 (m, 4H), 6.89 (d, J = 6.8 Hz, 1H), 6.75 (s, 1H), 5.96 (d, J = 9.2 Hz, 1H), 3.98 (d, J = 9.2 Hz, 1H), 2.30 (s, 3H), 0.90 (s, 9H); ^{13}C NMR (100 MHz, $CDCl_3$): δ 143.0, 140.4, 136.8, 133.3, 129.1, 128.8, 128.4, 127.0, 126.8,

126.3, 66.6, 35.2, 26.6, 21.4; HPLC conditions: DAICEL Chiralpak OJ column, Hexane/*i*-PrOH = 97/3, 220 nm, 0.5 mL/min, t_{major}=25.9 min, t_{minor}=35.0 min; $[\alpha]_D^{20}$ = –47.8 (*c* 0.20, CH_2Cl_2); IR (KBr) (v/cm^{-1}): 3289, 2966, 1597, 1438, 1331, 1160, 794, 698, 668; HRMS (ESI-MS) Calcd. For $C_{18}H_{22}ClNNaO_2S$ $[M+Na]^+$ 374.0958, found: 374.0952.

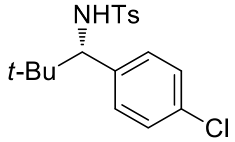

(S)-*N*-(1-(4-Chlorophenyl)-2,2-dimethylpropyl)-4-methylbenzenesulfonamide (2n)

White solid, 97% yield, 99.9% ee, mp: 206−208 °C; ^1H NMR (400 MHz, $CDCl_3$): δ 7.47 (d, J = 6.8 Hz, 2H), 7.01−6.97 (m, 4H), 6.84 (d, J = 7.2 Hz, 2H), 6.15 (d, J = 9.6 Hz, 1H), 4.00 (d, J = 9.2 Hz, 1H), 2.32 (s, 3H), 0.87 (s, 9H); ^{13}C NMR (100 MHz, $CDCl_3$): δ 143.0, 137.0, 136.8, 132.5, 129.5, 129.1, 127.5, 127.0, 66.5, 35.2, 26.5, 21.4; HPLC conditions: DAICEL Chiralpak OJ column, Hexane/*i*-PrOH = 95/5, 220 nm, 1.0 mL/min, t_{major}= 16.1 min, t_{minor}= 22.5 min; $[\alpha]_D^{20}$ = –54.4 (*c* 0.09, CH_2Cl_2);IR (KBr) (v/cm^{-1}): 3272, 2987, 1507, 1334, 1163, 802, 704; HRMS (ESI-MS) Calcd. For $C_{18}H_{22}ClNNaO_2S$ $[M+Na]^+$ 374.0958, found: 374.0966.

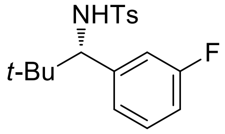

(*S*)-*N*-(1-(3-Fluorophenyl)-2, 2-dimethylpropyl)-4-methylbenzenesulfonamide (2o)

White solid, 99% yield, 99.9% ee, mp: 164−166 °C; ^1H NMR (400 MHz, $CDCl_3$): δ 7.45 (d, J = 8.4 Hz, 2H), 7.04−6.70 (m, 3H), 6.77−6.72 (m, 2H), 6.57−6.54 (m, 1H), 5.50 (d, J = 8.8 Hz, 1H), 4.0 (d, J = 9.2 Hz, 1H), 2.30 (s, 3H), 0.89 (s, 9H); ^{13}C NMR (100 MHz, $CDCl_3$): δ 163.2, 160.8, 143.0, 141.0 (d, J = 6.6 Hz), 137.1, 129.0 (t, J = 2.9 Hz), 127.0, 123.9 (d, J = 2.8 Hz), 115.2 (d, J = 21.7 Hz), 113.6 (d, J =21.0 Hz), 66.5, 35.2, 26.6, 21.4; ^{19}F NMR(376 MHz, $CDCl_3$): -114.1; HPLC conditions: DAICEL ChiralpakOJ column, Hexane/*i*-PrOH = 70/30, 220 nm, 1.0 mL/min, t_{major} =4.7 min, t_{minor} = 5.5 min; $[\alpha]_D^{20}$ = –21.5 (*c* 0.37, CH_2Cl_2); IR (KBr) (v/cm^{-1}): 3273, 2971, 1791, 1771, 1749, 810, 699, 680; HRMS (ESI-MS) Calcd. For $C_{18}H_{22}NO_2SNa$

$[M+Na]^+$ 358.1262, found: 358.1263.

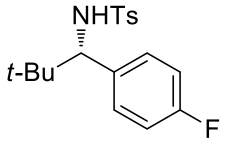

(*S*)-*N*-(2, 2-Dimethyl-1-(p-tolyl)propyl)-4-methylbenzenesulfonamide (2p)

White solid, 96% yield, 99.9% ee, mp: 204−206 °C; ^{1}H NMR (400 MHz, $CDCl_3$): δ 7.44 (d, J = 8.4 Hz, 2H), 7.01 (d, J = 8.0 Hz, 2H), 6.89−6.86 (m, 2H), 6.74 (d, J = 8.4 Hz, 2H), 5.62 (d, J = 9.2 Hz, 1H), 4.01 (d, J =8.8 Hz, 1H), 2.31 (s, 3H), 0.88 (s, 9H); ^{13}C NMR (100 MHz, $CDCl_3$): δ 162.9, 160.5, 142.9, 137.2, 134.1(d, J = 3.3 Hz), 129.6 (d, J = 8.1 Hz), 129.1, 127.0, 66.3, 35.2, 26.6, 21.4; ^{19}F NMR(376 MHz, $CDCl_3$): -115.9; HPLC conditions: DAICEL Chiralpak OJ column, Hexane/*i*-PrOH = 70/30, 220 nm, 1.0 mL/min, t_{major} =5.1 min, t_{minor} = 6.2 min; $[\alpha]_D^{20}$ = –28.8 (*c* 0.36, CH_2Cl_2); IR (KBr) (v/cm^{-1}): 3272, 2971, 1558, 1515, 1540, 840, 809, 699; HRMS (ESI-MS) Calcd. For $C_{18}H_{22}NO_2SNa$ $[M+Na]^+$ 358.1265, found: 358.1267.

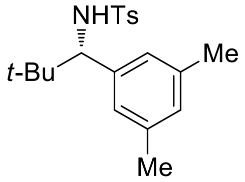

(*S*)-*N*-(1-(3, 5-Dimethylphenyl)-2,2-dimethylpropyl)-4-methylbenzenesulfonamide (2q)

White solid, 98% yield, 99.9% ee, mp: 167−169 °C; ^{1}H NMR (400 MHz, $CDCl_3$): δ 7.38 (d, J = 8.4 Hz, 2H), 6.95 (d, J = 8.4 Hz, 2H), 6.66 (s, 1H), 6.41 (s, 2H), 5.15 (d, J = 9.6 Hz, 1H), 3.93 (d, J = 9.6 Hz, 1H), 2.28 (s, 3H), 2.10 (s, 6H), 0.89 (s, 9H); ^{13}C NMR (100 MHz, $CDCl_3$): δ 142.3, 138.0, 137.3, 136.7, 128.1, 127.0, 126.1, 67.1, 35.1, 26.8, 21.3, 21.1; HPLC conditions: DAICEL Chiralpak OD column, Hexane/*i*-PrOH = 93/7, 220 nm, 1.0 mL/min, t_{major} = 5.6 min, t_{minor} = 6.1 min; $[\alpha]_D^{20}$ = –28.8 (*c* 0.36, CH_2Cl_2); IR (KBr) (v/cm^{-1}): 2952, 2871, 1558, 1521, 1540, 848, 808; HRMS (ESI-MS) Calcd. For $C_{20}H_{27}NO_2S$ $[M+Na]^+$ 368.1660, found: 368.1660.

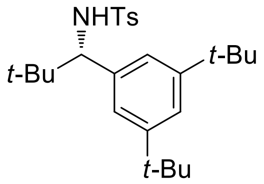

(*S*)-*N*-(1-(3, 5-Di-tert-butylphenyl)-2,2-dimethylpropyl)-4-methylbenzenesulfonamide (2r)

White solid, 99% yield, 99.9% ee, mp: 199−201 °C; ^{1}H NMR (400 MHz, $CDCl_3$): δ 7.34 (d, J = 8.0 Hz, 2H), 7.05 (t, J = 1.2 Hz, 1H), 6.84 (d, J = 8.0 Hz, 2H), 6.64 (d, J = 1.2 Hz, 2H), 5.37 (d, J = 10.0 Hz, 1H), 4.07 (d, J = 10.0 Hz, 1H), 2.19 (s, 3H), 1.17 (s, 18H), 0.93 (s, 9H); ^{13}C NMR (100 MHz, $CDCl_3$): δ 149.5, 142.1, 137.4, 136.9, 128.8, 127.0, 122.2, 120.4, 67.4, 35.3, 34.5, 31.3, 26.7, 21.3; HPLC conditions: DAICEL Chiralpak IC column, Hexane/*i*-PrOH = 95/5, 220 nm, 0.8 mL/min, t_{major}=15.4 min, t_{minor} =16.0 min; $[\alpha]_D^{20}$ = –7.21 (*c* 0.36, CH_2Cl_2); IR (KBr) (v/cm^{-1}): 2961, 1791, 1698, 1683, 808, 719; HRMS (ESI-MS) Calcd. For $C_{26}H_{39}NO_2SNa$ $[M+Na]^+$ 452.2603, found: 452.2599.

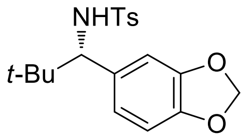

(S)-*N*-(1-(Benzo[d][1,3]dioxol-5-yl)-2,2-dimethylpropyl)-4-methylbenzenesulfonamide (2s)

White solid, 98% yield, 99.9% ee, mp: 161−163 °C; ^{1}H NMR (400 MHz, $CDCl_3$): δ 7.46 (d, J = 8.4 Hz, 2H), 7.04 (d, J = 8.0 Hz, 2H), 6.52 (d, J = 8.0 Hz, 1H), 6.41 (dd, J = 1.6, 8.0 Hz, 1H), 6.33 (d, J = 1.6 Hz, 1H), 5.84 (dd, J = 1.2, 7.2 Hz, 2H), 5.44 (d, J = 9.2 Hz, 1H), 3.94 (d, J = 8.8 Hz, 1H), 2.32 (s, 3H), 0.88 (s, 9H); ^{13}C NMR (100 MHz, $CDCl_3$): δ 146.7, 146.2, 142.6, 137.3, 132.4, 128.9, 127.1, 121.7, 108.4, 107.3, 100.8, 66.8, 35.3, 26.7, 21.4; HPLC conditions: DAICEL Chiralpak OJ column, Hexane/*i*-PrOH = 95/5, 220 nm, 1.0 mL/min, t_{major}=28.1 min, t_{minor} =38.2 min; $[\alpha]_D^{20}$ = –65.9 (*c* 0.20, CH_2Cl_2); IR (KBr) (v/cm^{-1}): 3289, 2965, 1456, 1326, 1159, 929, 810, 669, 561; HRMS (ESI-MS) Calcd. For $C_{19}H_{24}NO_4S$ $[M+Na]^+$ 362.1426, found: 362.1434.

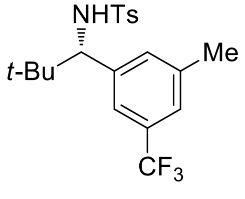

(*S*)-*N*-(2,2-Dimethyl-1-(3-methyl-5-(trifluoromethyl)phenyl)propyl)-4-methylbenzenesulfonamide (2t)

White solid, 99% yield, 99.9% ee, mp: 154−156 °C; ^{1}H NMR (400 MHz, $CDCl_3$): δ 7.41 (d, J = 8.0 Hz, 2H), 7.01 (d, J = 8.8 Hz, 2H), 6.58 (d, J = 9.2 Hz, 1H), 6.38 (d, J = 14.0 Hz, 2H), 4.96 (d, J = 16.8 Hz, 1H), 3.96 (d, J = 9.6 Hz, 1H), 2.30 (s, 3H), 2.15 (s, 3H), 0.88 (s, 9H); ^{13}C NMR (100 MHz, $CDCl_3$): δ 163.2, 160.8, 142.8, 140.5(d, J = 7.1 Hz), 139.2 (d, J = 8.0 Hz), 137.1, 128.9, 127.0, 124.7, 114.1 (d, J = 20.9 Hz), 112.3,(d, J = 21.9 Hz), 66.6, 35.2, 26.6, 21.3, 21.1; ^{19}F NMR(376 MHz, $CDCl_3$): -115.2; HPLC conditions: DAICEL Chiralpak IC column, Hexane/*i*-PrOH = 95/5, 220 nm, 0.8 mL/min, t_{major}=28.1 min, t_{minor} =30.8 min; $[\alpha]_D^{20}$ = –19.9 (*c* 0.4, CH_2Cl_2); IR (KBr) (v/cm^{-1}): 2969, 2872, 1595, 1540, 1521, 808, 719; HRMS (ESI-MS) Calcd. For $C_{20}H_{24}F_3NO_2SNa$ $[M+Na]^+$ 422.1875, found: 422.1875.

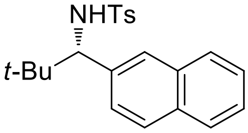

(*S*)-*N*-(2, 2-Dimethyl-1-(naphthalen-2-yl)propyl)-4-methylbenzenesulfonamide (2u)

White solid, 99% yield, 99.4% ee, mp: 174−176 °C; ^{1}H NMR (400 MHz, $CDCl_3$): δ 7.71−7.69 (m, 1H), 7.57−7.55 (m, 1H), 7.51 (d, J = 8.4 Hz, 1H), 7.41−7.35 (m, 4H), 7.21 (s, 1H), 7.07 (d, J = 8.4 Hz, 1H), 6.70 (d, J = 7.6 Hz, 2H), 5.58 (d, J = 9.2 Hz, 1H), 4.20 (d, J = 9.2 Hz, 1H), 1.95 (s, 3H), 0.97 (s, 9H); ^{13}C NMR (100 MHz, $CDCl_3$): δ 142.7, 137.0, 135.5, 132.5, 132.2, 128.8, 127.7, 127.5, 127.3, 127.2, 126.9, 126.0, 125.9, 125.7, 67.2, 35.5, 26.8, 21.0; HPLC conditions: DAICEL Chiralpak OD column, Hexane/*i*-PrOH = 96/4, 220 nm, 1.0 mL/min, t_{major}=16.9 min, t_{minor} =22.1 min; $[\alpha]_D^{20}$ = –23.1 (*c* 0.48, CH_2Cl_2); IR (KBr) (v/cm^{-1}): 3272, 2966, 1558, 1540, 1507, 848, 810, 741; HRMS (ESI-MS) Calcd. For $C_{22}H_{25}NO_2SNa$ $[M+Na]^+$ 390.1526, found: 390.1504.

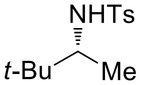

(*R*)-*N*-(3, 3-Dimethylbutan-2-yl)-4-methylbenzenesulfonamide (2v)[13]

White solid, 99% yield, 99.9% ee; ^{1}H NMR (400 MHz, $CDCl_3$): δ 7.76 (d, J = 8.4 Hz, 2H), 7.28 (d, J = 7.6 Hz, 2H), 4.20 (d, J = 9.2 Hz, 1H), 3.07−3.02 (m, 1H), 2.42 (s, 3H), 0.87(d, J = 7.6 Hz, 3H), 0.82 (s, 9H); HPLC conditions: DAICEL Chiralpak OJ

column, Hexane/*i*-PrOH = 99/1, 220 nm, 1.0 mL/min, t_{minor}= 13.0 min t_{major}= 13.9 min.

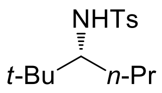

(*R*)-*N*-(2, 2-Dimethylhexan-3-yl)-4-methylbenzenesulfonamide (2w)[15]

White solid, 97% yield, 99.9% ee; ^{1}H NMR (400 MHz, $CDCl_3$): δ 7.76 (d, J = 8.4 Hz, 2H), 7.29 (d, J = 4.4 Hz, 2H), 4.20 (d, J = 9.2 Hz, 1H), 3.09−3.06 (m, 1H), 2.43 (s, 3H), 1.56−1.49 (m, 1H), 1.29−1.02 (m, 3H), 0.82−0.79 (m, 12H); HPLC conditions: DAICEL Chiralpak OD column, Hexane/*i*-PrOH = 93/7, 220 nm, 1.0 mL/min, t_{minor} = 6.5 min, t_{major} = 6.8 min.

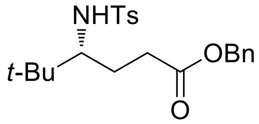

(*R*)-Benzyl 5,5-dimethyl-4-(4-methylphenylsulfonamido)hexanoate (2x)

White solid, 98% yield, 99.7% ee, mp: 92−95 °C; ^{1}H NMR (400 MHz, $CDCl_3$): δ 7.69 (d, J = 8.4 Hz, 2H), 7.38−7.29 (m, 5H), 7.21 (d, J = 8.4 Hz, 2H), 5.08 (s, 2H), 4.30 (d, J = 9.6 Hz, 1H), 3.15−3.09 (m, 1H), 2.39−2.31 (m, 5H), 1.99−1.92 (m, 1H), 1.52−1.36 (m, 1H), 0.76 (s, 9H); ^{13}C NMR (100 MHz, $CDCl_3$): δ 173.6, 143.0, 139.0, 136.0, 129.5, 128.5, 128.2, 128.1, 126.9, 66.3, 62.8, 35.1, 31.1, 26.6, 26.2, 21.4; HPLC conditions: DAICEL Chiralpak IE column, Hexane/*i*-PrOH = 90/10, 220 nm, 0.5 mL/min, t_{major}= 58.9 min, t_{minor} = 64.6 min; $[\alpha]_D^{20}$ = –13.8 (*c* 0.50, CH_2Cl_2); IR (KBr) (ν/cm^{-1}): 3293, 2963, 1735, 1324, 1156, 1079, 1025, 814, 665, 579, 550; HRMS (ESI-MS) Calcd. For $C_{22}H_{30}NO_4S$ $[M+H]^+$ 404.1896, found: 404.1919.

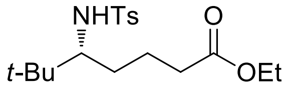

(*R*)-Ethyl 6,6-dimethyl-5-(4-methylphenylsulfonamido)heptanoate (2y)

Colourless thick liquid, 96% yield, 99.4% ee; ^{1}H NMR (400 MHz, $CDCl_3$): δ 7.70 (d, J = 8.0 Hz, 2H), 7.19 (d, J = 8.0 Hz, 2H), 5.05 (d, J = 9.2 Hz, 1H), 4.01 (q, J = 7.2 Hz, 2H), 2.98−2.93 (m, 1H), 2.33 (s, 3H), 2.08 (t, J = 7.2 Hz, 2H), 1.52−1.26 (m, 4H), 1.16 (t, J = 7.2 Hz, 3H), 0.73 (s, 9H); ^{13}C NMR (100 MHz, $CDCl_3$): δ 173.2, 142.9, 139.1, 129.4, 126.9, 63.1, 60.2, 35.0, 33.9, 31.0, 26.7, 22.2, 21.4, 14.2; HPLC

conditions: DAICEL Chiralpak OJ column, Hexane/*i*-PrOH = 92/8, 220 nm, 0.5 mL/min, t_{major}= 18.2 min, t_{minor} = 21.2 min; $[\alpha]_D^{20}$ = 1.2 (*c* 0.44, CH_2Cl_2); IR (KBr) (v/cm^{-1}): 2963, 1734, 1322, 1156, 1090, 1024, 815, 666, 578, 549; HRMS (ESI-MS) Calcd. For $C_{18}H_{30}NO_4S$ $[M+H]^+$ 356.1896, found: 356.1906.

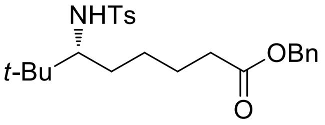

(*R*)-Benzyl 7,7-dimethyl-6-(4-methylphenylsulfonamido)octanoate (2z)

Pale yellow solid, 98% yield, 99.1% ee, mp: 93−94 °C; ^{1}H NMR (400 MHz, $CDCl_3$): δ 7.71 (d, J = 8.0 Hz, 2H), 7.38−7.32 (m, 5H), 7.23 (d, J = 8.0 Hz, 2H), 5.10 (s, 2H), 4.14 (d, J = 10.0 Hz, 1H), 3.02 (dt, J = 2.8, 9.6 Hz, 1H), 2.38 (s, 3H), 2.15 (t, J = 7.6 Hz, 2H), 1.55−1.39 (m, 4H), 1.13−1.06 (m, 2H), 0.80 (s, 9H); ^{13}C NMR (100 MHz, $CDCl_3$): δ 173.5, 143.0, 139.6, 136.3, 129.6, 128.8, 128.4, 127.1, 66.3, 63.6, 35.3, 34.3, 31.5, 27.0, 26.8, 25.0, 21.6; HPLC conditions: DAICEL Chiralpak IC-3 column, Hexane/*i*-PrOH = 90/10, 220 nm, 0.8 mL/min, t_{minor}= 41.3 min$_{major}$, t = 43.6 min; $[\alpha]_D^{20}$ = 6.6 (*c* 0.22, CH_2Cl_2); IR (KBr) (v/cm^{-1}): 3278, 2927, 1726, 1598, 1434, 1328, 1161, 1092, 815, 669, 552; HRMS (ESI-MS) Calcd. For $C_{24}H_{34}NO_4S$ $[M+H]^+$ 432.2209, found: 432.2216.

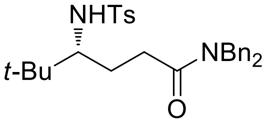

(*R*)-*N*,*N*-Dibenzyl-5,5-dimethyl-4-(4-methylphenylsulfonamido)hexanamide (2aa)

Pale yellow thick liquid, 97% yield, 99.4% ee; ^{1}H NMR (400 MHz, $CDCl_3$): δ 7.66 (d, J = 8.0 Hz, 2H), 7.36−7.22 (m, 8H), 7.11 (d, J = 7.6 Hz, 4H), 4.81 (d, J = 9.2 Hz, 1H), 4.57 (s, 2H), 4.27 (dd, J = 17.2, 22.4 Hz, 2H), 3.14 (t, J = 10.0 Hz, 1H), 2.45−2.36 (m, 2H), 2.30 (s, 3H), 2.04−1.97 (m, 1H), 1.66−1.56 (m, 1H), 0.77 (s, 9H); ^{13}C NMR (100 MHz, $CDCl_3$): δ 173.6, 142.8, 139.2, 137.2, 136.4, 129.4, 128.9, 128.6, 128.3, 127.6, 127.3, 126.9, 126.7, 63.0, 49.7, 48.3, 35.2, 30.1, 26.7, 26.0, 21.4; HPLC conditions: DAICEL Chiralpak IE column, Hexane/*i*-PrOH = 85/15, 230 nm, 0.8 mL/min, t_{major}= 49.2 min, t_{minor} = 59.9 min; $[\alpha]_D^{20}$ = –16.7 (*c* 0.64, CH_2Cl_2); IR (KBr) (v/cm^{-1}): 2962, 2360, 2342, 1629, 1452, 1327, 1155, 1077, 668, 580, 549; HRMS (ESI-MS) Calcd. For $C_{29}H_{37}N_2O_3S$ $[M+H]^+$ 493.2525, found: 493.2532.

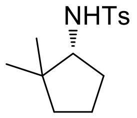

(*R*)-*N*-(2,2-Dimethylcyclopentyl)-4-methylbenzenesulfonamide (2ab)

White solid, 96% yield, 99.3% ee, mp: 76−77 °C; ^{1}H NMR (400 MHz, $CDCl_3$): δ 7.75 (d, J = 8.8 Hz, 2H), 7.26 (d, J = 8.8 Hz, 2H), 4.71 (d, J = 9.2 Hz, 1H), 3.10 (dd, J = 8.8, 18.0 Hz, 1H), 2.40 (s, 3H), 1.69−1.51 (m, 2H), 1.43−1.23 (m, 4H), 0.88 (s, 3H), 0.80 (s, 3H); ^{13}C NMR (100 MHz, $CDCl_3$): δ 143.1, 138.2, 129.5, 127.1, 62.8, 40.7, 38.3, 30.7, 26.6, 21.5, 21.2, 19.3; HPLC conditions: DAICEL Chiralpak AS column, Hexane/*i*-PrOH = 80/20, 220 nm, 0.6 mL/min, t_{minor}= 15.9 min, t_{major} = 22.9 min; $[\alpha]_D^{20}$ = –1.5 (*c* 0.13, CH_2Cl_2); IR (KBr) (v/cm^{-1}): 3276, 2958, 2872, 1598, 1452, 1322, 1158, 1093, 912, 814, 666, 571, 549; HRMS (ESI-MS) Calcd. For $C_{14}H_{21}NO_2SNa$ $[M+Na]^+$ 290.1191, found: 290.1206.

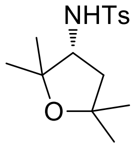

(*R*)-4-Methyl-*N*-(2,2,5,5-tetramethyltetrahydrofuran-3-yl)benzenesulfonamide (2ac)

Colourless thick liquid, 98% yield, 99.8% ee; ^{1}H NMR (400 MHz, $CDCl_3$): δ 7.76 (d, J = 8.0 Hz, 2H), 7.29 (d, J = 8.0 Hz, 2H), 5.11 (d, J = 9.6 Hz, 1H), 3.58 (dd, J = 10.0, 18.0 Hz, 1H), 2.41 (s, 3H), 1.79 (d, J = 10.0 Hz, 1H), 1.60 (d, J = 11.6 Hz, 1H), 1.17 (s, 3H), 1.11 (s, 3H), 1.07 (s, 3H), 1.04 (s, 3H); ^{13}C NMR (100 MHz, $CDCl_3$): δ 143.6, 137.6, 129.7, 127.0, 80.6, 77.4, 61.0, 44.1, 30.5, 30.1, 28.0, 23.5, 21.5; HPLC conditions: DAICEL Chiralpak AS column, Hexane/*i*-PrOH = 80/20, 220 nm, 0.7 mL/min, t_{minor}= 9.8 min, t_{major} = 13.0 min; $[\alpha]_D^{20}$ = –2.9 (*c* 0.36, CH_2Cl_2); IR (KBr) (v/cm^{-1}): 3271, 2972, 2928, 1458, 1325, 1162, 1093, 994, 664, 569; HRMS (ESI-MS) Calcd. For $C_{115}H_{23}NO_3SNa$ $[M+Na]^+$ 320.1296, found: 320.1308.

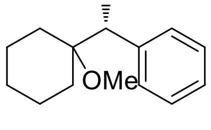

(*R*)-*N*-((1-Methoxycyclohexyl)(phenyl)methyl)-4-methylbenzenesulfonamide (2ad)

Colourless thick liquid, 99% yield, 99.4% ee; ^{1}H NMR (400 MHz, $CDCl_3$): δ 7.39 (d,

J = 8.0 Hz, 2H), 7.10−7.04 (m, 5H), 7.01 (d, J = 8.0 Hz, 2H), 5.36 (d, J = 3.2 Hz, 1H), 4.38 (d, J = 3.6 Hz, 1H), 3.06 (s, 3H), 2.29 (s, 3H), 1.67−1.04 (m, 10H); ^{13}C NMR (100 MHz, $CDCl_3$): δ 142.7, 137.2, 137.0, 129.0, 128.9, 127.5, 127.2, 127.1, 77.8, 61.2, 48.0, 29.9, 28.7, 25.2, 21.7, 21.4; HPLC conditions: DAICEL Chiralpak AS column, Hexane/*i*-PrOH = 85/15, 220 nm, 0.8 mL/min, t_{major}= 18.9 min, t_{minor} = 32.4 min; $[\alpha]_D^{20}$ = –63.8 (*c* 0.48, CH_2Cl_2); IR (KBr) (v/cm^{-1}): 2934, 2857, 2360, 2343, 1453, 1325, 1160, 1088, 812, 703, 669, 568; HRMS (ESI-MS) Calcd. For $C_{21}H_{27}NO_3SNa$ $[M+Na]^+$ 396.1609, found: 396.1617.

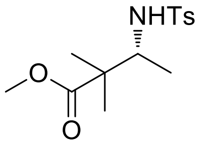

(*R*)-Methyl 2,2-dimethyl-3-(4-methylphenylsulfonamido)butanoate (2ae)

Colourless thick liquid, 96% yield, 96.9% ee; ^{1}H NMR (400 MHz, $CDCl_3$): δ 7.41 (d, J = 8.0 Hz, 2H), 7.25 (d, J = 8.0 Hz, 2H), 5.29 (d, J = 9.6 Hz, 1H), 3.56 (s, 3H), 3.40−3.33 (m, 1H), 2.37 (s, 3H), 1.09 (d, J = 11.2 Hz, 6H), 7.25 (d, J = 6.8 Hz, 3H); ^{13}C NMR (100 MHz, $CDCl_3$): δ 176.6, 143.1, 138.4, 129.6, 127.0, 56.1, 51.9, 46.6, 23.0, 22.8, 21.5, 17.4; HPLC conditions: DAICEL Chiralpak OJ column, Hexane/*i*-PrOH = 85/15, 220 nm, 0.6 mL/min, t_{major}= 20.2 min, t_{minor} = 25.4 min; $[\alpha]_D^{20}$ = 5.1 (*c* 0.62, CH_2Cl_2); IR (KBr) (v/cm^{-1}): 3274, 2981, 1733, 1457, 1436, 1327, 1161, 1092, 816, 669, 552; HRMS (ESI-MS) Calcd. For $C_{14}H_{21}NO_4SNa$ $[M+Na]^+$ 322.1089, found: 322.1092.

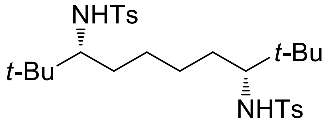

(*R*,*R*)-*N*,*N'*-((3R,8R)-2,2,9,9-tetramethyldecane-3,8-diyl)bis(4-methylbenzenesulfonamide) (2af)

White solid, mp: 208-210 °C; 98% yield, 99% de, 99.9% ee; ^{1}H NMR (400 MHz, DMSO) *δ* 7.61 (d, J = 8.0 Hz, 4H), 7.29 (d, J = 8.4 Hz, 4H), 7.15 (d, J = 8.8 Hz, 2H), 3.32 (s, 6H), 2.70 (d, J = 8.4 Hz, 2H), 2.32 (s, 6H), 0.97−0.80 (m, 4H), 0.73 (s, 18H), 0.51−0.39 (m, 10H); ^{13}C NMR (100 MHz, DMSO) *δ* 142.3, 141.2, 129.8, 126.9, 63.5, 35.4, 30.8, 27.5, 27.4, 21.6; HPLC conditions: DAICEL Chiralpak IC-3 column,

Hexane/*i*-PrOH = 90/10, 220 nm, 1.0 mL/min, t_{minor}= 72.9 min, t_{major} = 76.8 min; $[\alpha]_D^{20}$ = –5.6 (*c* 0.48, CH_2Cl_2); IR (KBr) (v/cm^{-1}): 3298, 2947, 1329, 1319, 1152, 1093, 1082, 1026, 805, 665, 582; HRMS (ESI-MS) Calcd. For $C_{28}H_{45}N_2O_4S_2$ $[M+H]^+$ 537.2821, found: 537.2837.

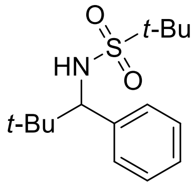

(*S*)-*N*-(2,2-Dimethyl-1-phenylpropyl)-2-methylpropane-2-sulfonamide (2ag)

White solid, mp: 169-172 °C; ^{1}H NMR (400 MHz, $CDCl_3$): δ 7.32−7.24 (m, 3H), 7.13 (d, J = 7.2 Hz, 2H), 4.67 (d, J = 9.6 Hz, 1H), 4.22 (d, J = 9.6 Hz, 1H), 1.13 (s, 9H), 0.95 (s, 9H); ^{13}C NMR (100 MHz, $CDCl_3$): δ 140.0, 128.2, 127.8, 127.2, 67.1, 59.7, 35.7, 26.8, 24.1; IR (KBr) (v/cm^{-1}): 3226, 3293, 2970, 2950, 1454, 1294, 1088, 1062, 709, 667, 556, 511; HRMS (ESI-MS) Calcd. For $C_{15}H_{29}N_2O_2S$ $[M+NH_4]^+$ 301.1950, found: 301.1950.

Supplementary Note 2

Transformations of compound 2a to compounds 5 and 6

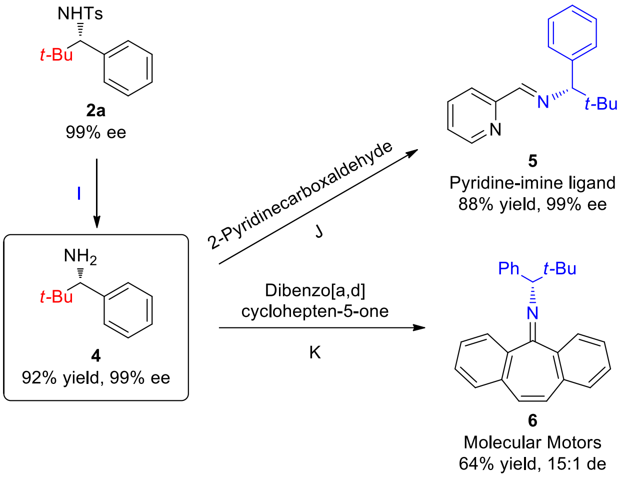

Procedure I: Sodium metal (0.46 g, 20.0 mmol) was weighed and added to a solution of naphthalene (2.82 g, 22.0 mmol) in dry DME (40 mL) under argon atmosphere. It was stirred for 4 h at RT during which the metal slowly dissolved to give a 0.5 M solution of sodium naphthalide.

To a solution of sulfamide **2a** (0.64 g, 2.0 mmol, 1.0 equiv) in DME (20 mL) at -70 ^{o}C under argon atmosphere was added dropwise Sodium naphthalide (0.5 M in DME, 40 mL, 10 equiv) for 1 minute. The resulting mixture was stirred at -70 - 0 ^{o}C for 2 h. After the material disappeared, the reaction was quenched with 2 drops of water and extracted with EtOAc (60 mL × 3). The mixture was diluted with saturated aqueous NH_4Cl solution (60 mL) and saturated aqueous $NaHCO_3$ solution (60 mL) respectively, dried with $MgSO_4$. After filtration, the solvents were evaporated and the residue was purified by column chromatography (PE/EtOAc=8/1) to give product **4** with 91% yield (296.7 mg), 99% ee (The ee was determined by HPLC after transformation of (*S*)-**4** to (*S*)-**5**). ^{1}H NMR (400 MHz, $CDCl_3$): δ 7.28−7.21 (m, 5H), 3.68 (s, 1H), 1.68 (s, 2H), 0.89 (s, 9H); ^{13}C NMR (100 MHz, CDCl3): δ 143.6, 128.2, 127.5, 126.7, 65.3, 35.0, 26.5 (*Angew. Chem. Int. Ed.* **2007**, *46*, 8484 –8487).

Procedure J: Mixtures (1:1.1) of chiral amines **4** (81.5 mg, 0.5 mmol) and 2-pyridinecarboxaldehyde (58.8 mg, 0.55 mmol) were dissolved in dry Et_2O (10 mL) and the mixture stirred at room temperature overnight until the material disappeared. The reaction mixture was quenched with saturated NaCl solution (10 mL), extracted

with EtOAc (40 mL × 3), dried over Na_2SO_4. After filtration, the solvents were evaporated and the residue was purified by column chromatography (PE/EtOAc=20/1) to give product **5**. White solid, 110.9 mg, 88% yield, 99.3% ee, ^{1}H NMR (400 MHz, $CDCl_3$): δ 8.60 (d, J = 4.8 Hz, 1H), 8.37 (s, 1H), 8.19 (d, J = 8.0 Hz, 1H), 7.72 (t, J = 8.0 Hz, 1H), 7.43 (d, J = 7.6 Hz, 2H), 7.31−7.22 (m, 4H), 4.05 (s, 1H), 0.95 (s, 9H); ^{13}C NMR (100 MHz, $CDCl_3$): δ 160.6, 155.0, 149.2, 141.9, 136.4, 128.8, 127.5, 126.7, 124.5, 121.2, 84.8, 35.7, 26.9; HPLC conditions: DAICEL Chiralpak OD column, Hexane/*i*-PrOH = 95/5, 230 nm, 0.5 mL/min, t_{minor}= 8.4 min, t_{major} = 9.6 min (*Angew. Chem. Int. Ed*. **2007**, *46*, 2082 –2085).

Procedure K: To a solution of the dibenzo[*a*,*d*]cyclohepten-5-one (0.5 mmol), the chiral amines **4** (81.5 mg, 0.5 mmol) and Et_3N (5.0 mmol) in dry toluene was added dropwise as solution of $TiCl_4$ at room temperature. The mixture was stirred for 16 h at room temperature, quenched by addition of saturated Na_2CO_3 solution and extracted with CH_2Cl_2 (30 mL × 3). The combined organic phases were evaporated. The residue was purified by column chromatography (PE/EtOAc=20/1) to afford the imines **6** as solids. White solid, 126.4 mg, 69% yield, Resolution of the racemate was not been successful by HPLC. ^{1}H NMR (400 MHz, $CDCl_3$): δ 7.55 (d, J = 7.2 Hz, 1H), 7.47−7.22 (m, 11H), 6.96 (d, J = 5.6 Hz, 2H), 6.81 (d, J = 7.6 Hz, 1H), 4.12 (s, 1H), 0.66 (s, 9H) (J. *Am. Chem. Soc.*, **2014**, *136*, 13114–13117).

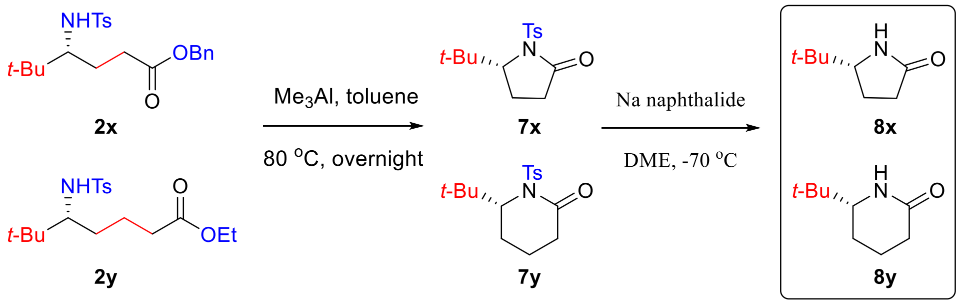

Procedure L: To a solution of **2x**, **y** (1.0 mmol in 20 mL toluene) in tube sealing was added 1.5 mL of trimethylaluminum (1.5 mmol, 1M in hexanes). The reaction mixture was stirred at different temperature and time. After cooling the reaction mixture to 0 °C, 1M aqueous hydrochloric acid (10 mL) was added cautiously over 15 min. The resulting biphasic mixture was stirred for 10 min at 0 °C and then transferred to a

separatory funnel. The mixture was extracted with ethyl acetate (3×20 mL). The combined organic layers were dried over magnesium sulfate, filtered and solvents were removed *in vacuo* to give a yellow solid. The residue was purified by flash chromatography (PE/EtOAc=10/1) and recrystallization to give **7x**, **y**.

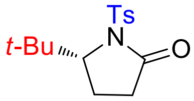

(*R*)-5-(Tert-butyl)-1-tosylpyrrolidin-2-one (7x)

The reaction mixture was stirred at 60 °C for overnight. White solid, mp: 185-186 °C; 274.3 mg, 93% yield, 99.9% ee; ^{1}H NMR (400 MHz, $CDCl_3$): δ 7.86 (d, J = 7.6 Hz, 2H), 7.28 (d, J = 8.0 Hz, 2H), 4.26 (d, J = 3.6 Hz, 1H), 2.57−2.47 (m, 1H), 2.40 (s, 3H), 2.24−2.02 (m, 3H), 1.01 (s, 9H); ^{13}C NMR (100 MHz, $CDCl_3$): δ 175.6, 144.7, 136.2, 129.3, 128.5, 69.1, 36.7, 31.9, 26.7, 22.7, 21.6; HPLC conditions: DAICEL Chiralpak AS column, Hexane/*i*-PrOH = 85/15, 220 nm, 1.0 mL/min, t_{major}= 11.2 min, t_{minor} = 14.6 min; $[\alpha]_D^{20}$ = 76.8 (*c* 0.18, CH_2Cl_2); IR (KBr) (v/cm^{-1}): 2961, 2918, 1718, 1359, 1172, 1088, 944, 666, 603, 551; HRMS (ESI-MS) Calcd. For $C_{15}H_{22}NO_3S$ $[M+H]^+$ 296.1320, found: 296.1335.

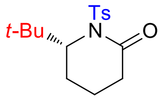

(*R*)-6-(Tert-butyl)-1-tosylpiperidin-2-one (7y)

The reaction mixture was stirred at 80 °C for 24 h. White solid, mp: 134-135 °C; 278.1 mg, 90% yield, 99.9% ee; ^{1}H NMR (400 MHz, $CDCl_3$): δ 7.78 (d, J = 8.0 Hz, 2H), 7.25 (d, J = 8.0 Hz, 2H), 4.55 (t, J = 6.0 Hz, 1H), 2.39 (s, 3H), 2.29−2.17 (m, 2H), 1.97−1.92 (m, 2H), 1.89−1.84 (m, 1H), 1.43−1.35 (m, 1H), 1.05 (s, 9H); ^{13}C NMR (100 MHz, $CDCl_3$): δ 172.7, 144.3, 136.4, 129.1, 128.8, 63.1, 37.3, 33.6, 27.5, 24.6, 21.6, 18.4; HPLC conditions: DAICEL Chiralpak OJ column, Hexane/*i*-PrOH = 80/20, 210 nm, 1.0 mL/min, t_{major}= 10.5min, t_{minor} = 12.5 min; $[\alpha]_D^{20}$ = 51.0 (*c* 0.73, CH_2Cl_2); IR (KBr) (v/cm^{-1}): 2966, 2920, 2875, 2360, 1699, 1348, 1163, 598; HRMS (ESI-MS) Calcd. For $C_{16}H_{24}NO_3S$ $[M+H]^+$ 310.1477, found: 310.1464.

Procedure I: **7x**, **y** (0.5 mmol), naphthalene (5.0 mmol), sodium (5.0 mmol), DME (30 mL) -70 °C, 1 min. The residue was purified by column chromatography (EtOAc)

to give product **8x**, **y**.

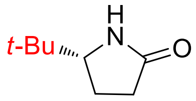

(*R*)-5-(Tert-butyl)pyrrolidin-2-one (8x)

White solid, mp: 149-151 °C; 87% yield, 97% ee, ^{1}H NMR (400 MHz, $CDCl_3$): δ 6.42 (bs, 1H), 3.37 (t, J = 6.8 Hz, 1H), 2.29 (t, J = 8.0 Hz, 2H), 2.08−1.99 (m, 1H), 1.87−1.78 (m, 1H), 0.87 (s, 9H); ^{13}C NMR (100 MHz, $CDCl_3$): δ 178.6, 63.7, 33.7, 30.5, 25.3, 22.1; HPLC conditions: DAICEL Chiralpak AS column, Hexane/*i*-PrOH = 85/15, 220 nm, 1.0 mL/min, t_{major}= 11.2 min, t_{minor} = 14.6 min; $[\alpha]_D^{20}$ = 11.9 (*c* 0.17, CH_2Cl_2); IR (KBr) (v/cm^{-1}): 3197, 2956, 2871, 1702, 1663, 1358, 1309, 1272; HRMS (ESI-MS) Calcd. For $C_8H_{16}NO$ $[M+H]^+$ 142.1232, found: 142.1229.

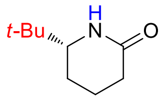

(*R*)-6-(tert-butyl)piperidin-2-one (8y)

White solid, mp: 71-73 °C; 82% yield, 98% ee, ^{1}H NMR (400 MHz, $CDCl_3$): δ 5.76 (bs, 1H), 3.04 (d, J = 11.2 Hz, 1H), 2.37−2.33 (m, 1H), 2.21−2.12 (m, 1H), 1.87 (t, J = 14.0 Hz, 2H), 1.65−1.54 (m, 1H), 1.34−1.28 (m, 1H), 0.87 (s, 9H); ^{13}C NMR (100 MHz, $CDCl_3$): δ 173.0, 62.4, 33.6, 31.3, 25.5, 23.5, 20.3; HPLC conditions: DAICEL Chiralpak AS column, Hexane/*i*-PrOH = 85/15, 210 nm, 1.0 mL/min, t_{major}= 18.4 min, t_{minor} = 27.7 min; $[\alpha]_D^{20}$ = 30.7 (*c* 0.68, CH_2Cl_2); IR (KBr) (v/cm^{-1}): 3234, 2958, 2870, 1664, 1652, 1507, 1473, 1405, 1329, 1307; HRMS (ESI-MS) Calcd. For $C_9H_{18}NO$ $[M+H]^+$ 156.1388, found: 156.1390.

Supplementary Note 3

Weak attractive catalyst-substrate interactions

Supplementary Table 1. Close intramolecular interactions found in **TS(*S*)** and **TS(*R*)**

entry	Type of interaction		Interatomic distance, Å	
			**TS(*S*)**	**TS(*R*)**
1	Trasnferred hydride	*o*-Ph-H	1.90	2.42
2		*t*-Bu (imine)	2.21, 2.76	2.37, 2.44
3		*t*-Bu1 (cat.)	2.65	2.68
4		Me^1 (cat.)	2.85	-
5	*t*-Bu^1(cat.)	*o*-Ph-H	2.34, 2.36	2.97, 2.98
6		S=O	2.78	2.63
7	*t*-Bu^2(cat.)	*t*-Bu (imine)	2.11, 2.08	2.22, 2.54
8		Tosyl (CH···π)	2.81	-
9		Tosyl (CH···HC)	2.76, 2.82	-
10	Me^1(cat.)	Tosyl (CH···π)	2.73, 2.92	2.42, 2.50
11	Me^2(cat.)	*o*-Ph-H	-	2.42
12	C=N···Pd		2.25	-
13	O=S=O···Pd		-	2.21

The computed transition states, **TS(*S*)** and **TS(*R*)**, for the hydrogen transfer are shown for the substrate **1a**. Both structures are stabilized by the numerous weak intermolecular interactions (Supplementary Table 1). Of interest is the extremely short interatomic distance between the migrating hydride and the *o*-hydrogen atom of the phenyl ring in **TS(*S*)** (1.90 Å vs 2.42 Å in the **TS(*R*)**). In addition, binding of the C=N group available only in **TS(*S*)** leads to the formation of a four-membered ring transition state stabilized by many CH···π and CH···HC interactions between the Ts group and the Me or *t*-Bu substituents of the catalyst (trans to the migrating hydride), respectively. Due to the fixed geometry of the imine, in **TS(*R*)** the lone pair of the nitrogen atom is not available for making a coordination bond with Pd. Instead, the O=S=O···Pd interaction leads to formation of a six-membered transition state stabilized by CH···π interactions between the Ts group and the *t*-Bu group of the catalyst (*cis* to the migrating hydride).

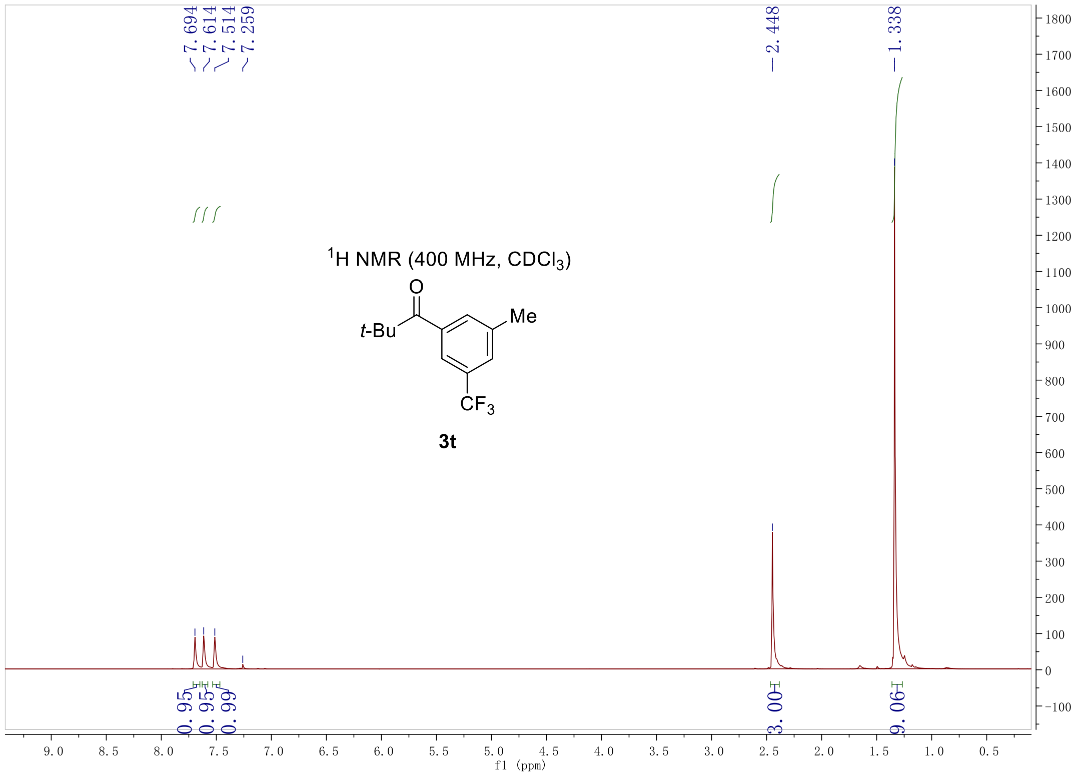

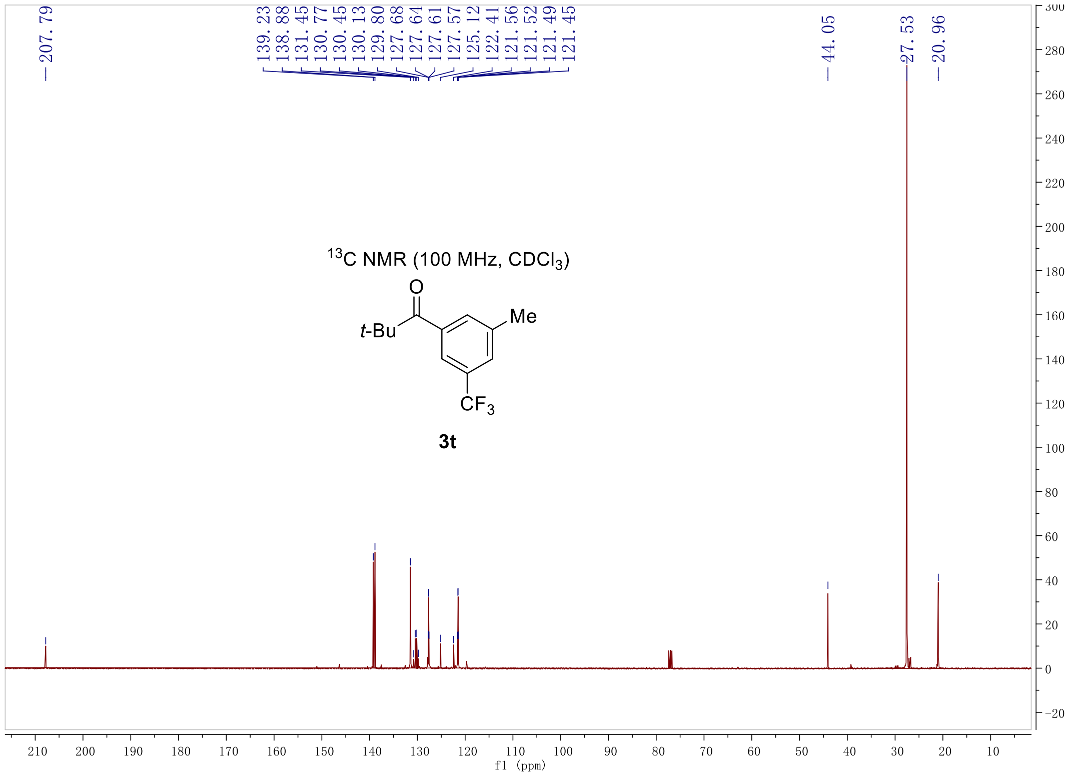

Supplementary Figure 1 ^{1}H and ^{13}C NMR spectrum of compound **3t** in CDCl3

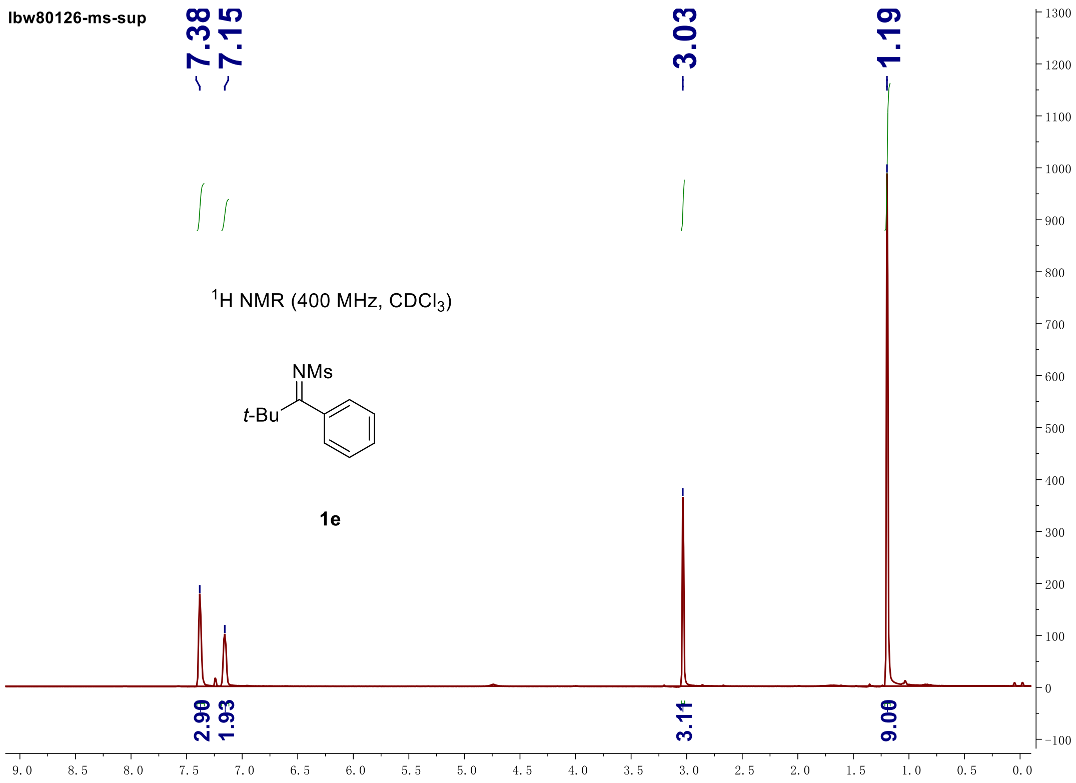

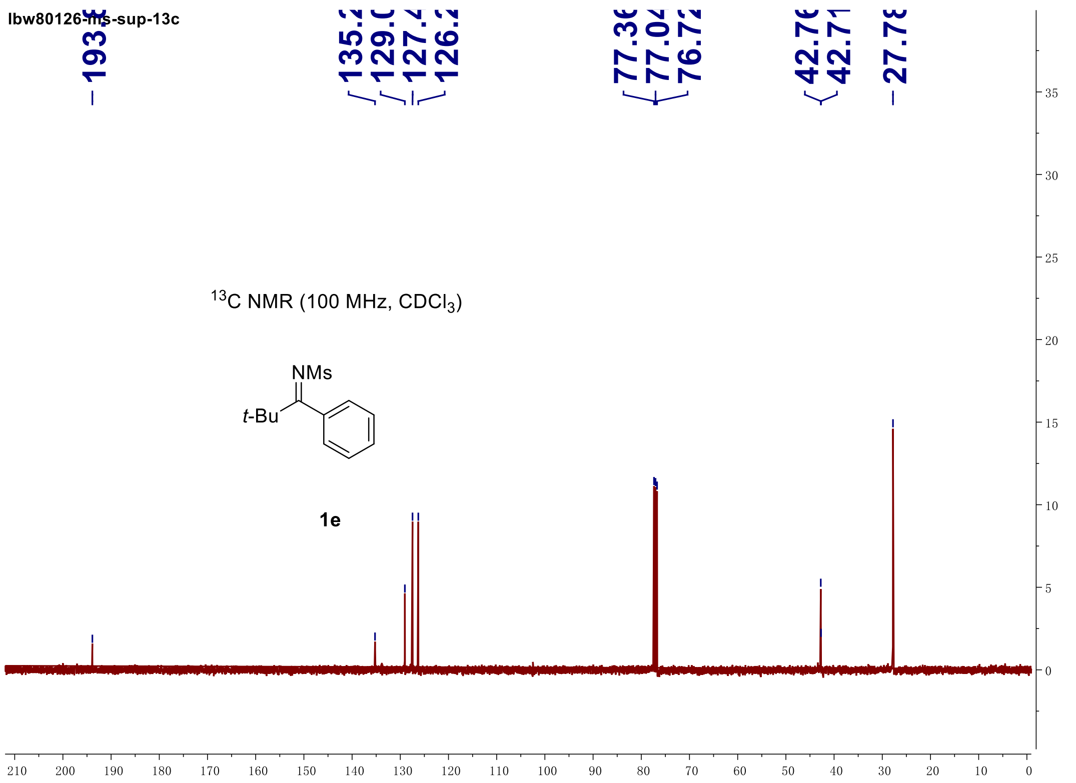

Supplementary Figure 2 ^{1}H and ^{13}C NMR spectrum of compound **1e** in $CDCl_3$

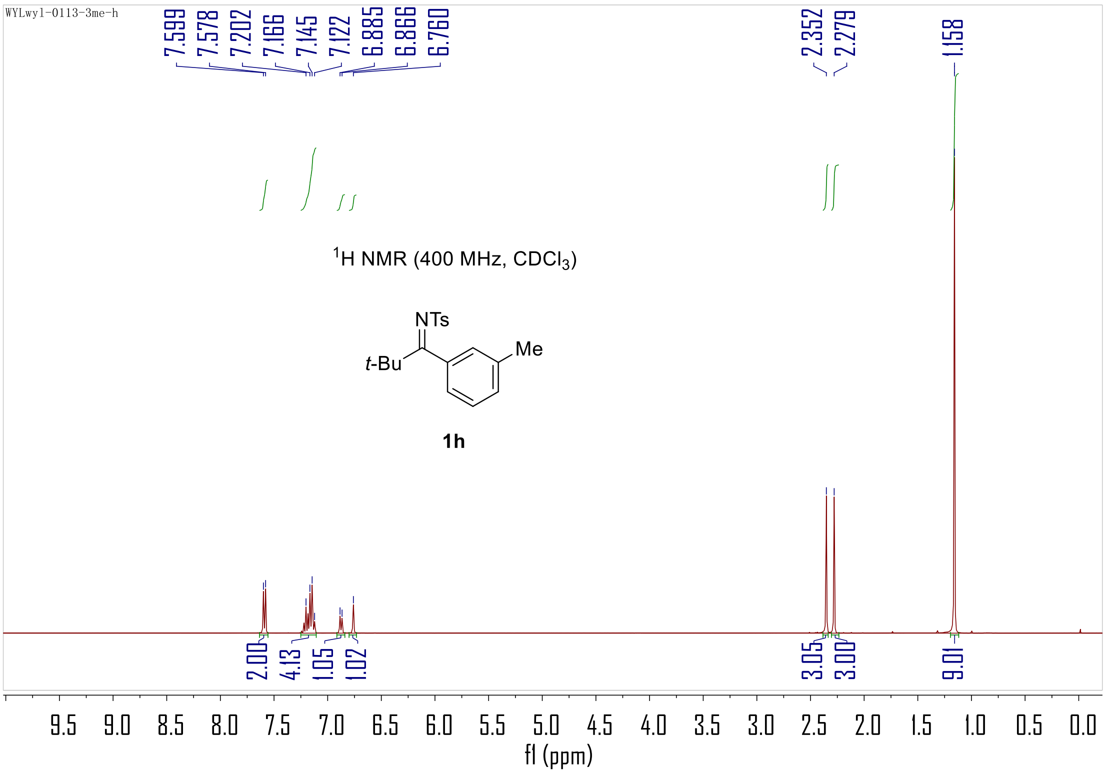

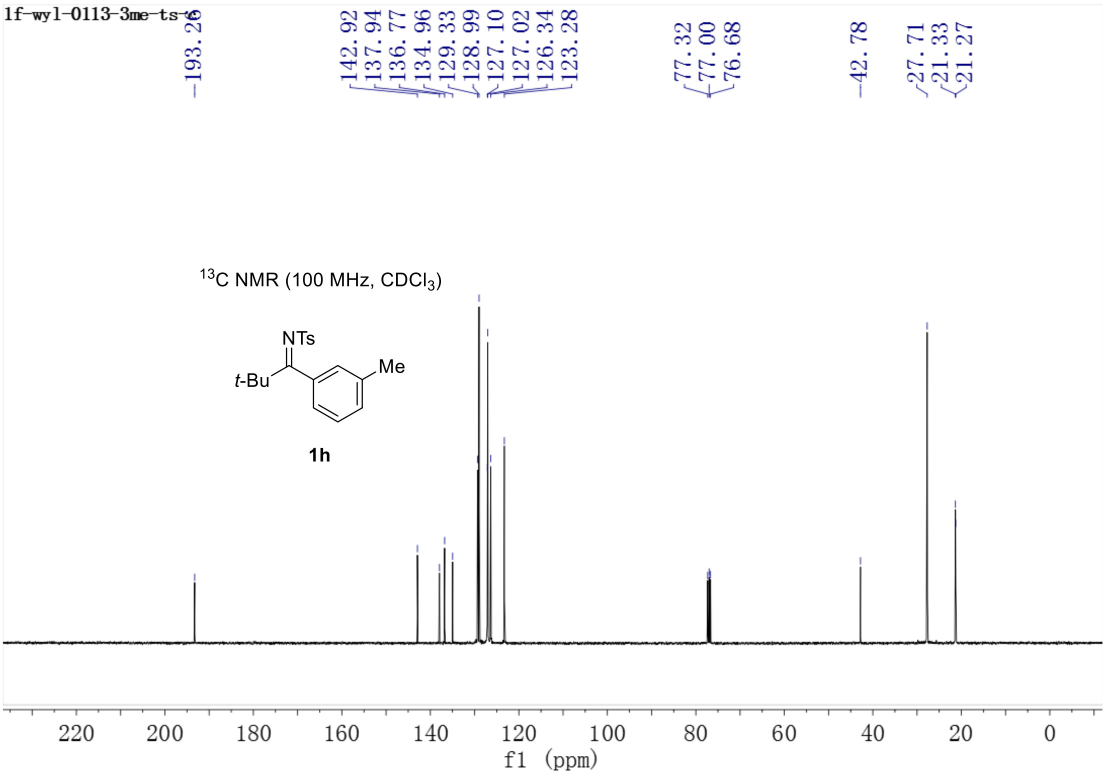

Supplementary Figure 3 ^{1}H and ^{13}C NMR spectrum of compound **1h** in CDCl3

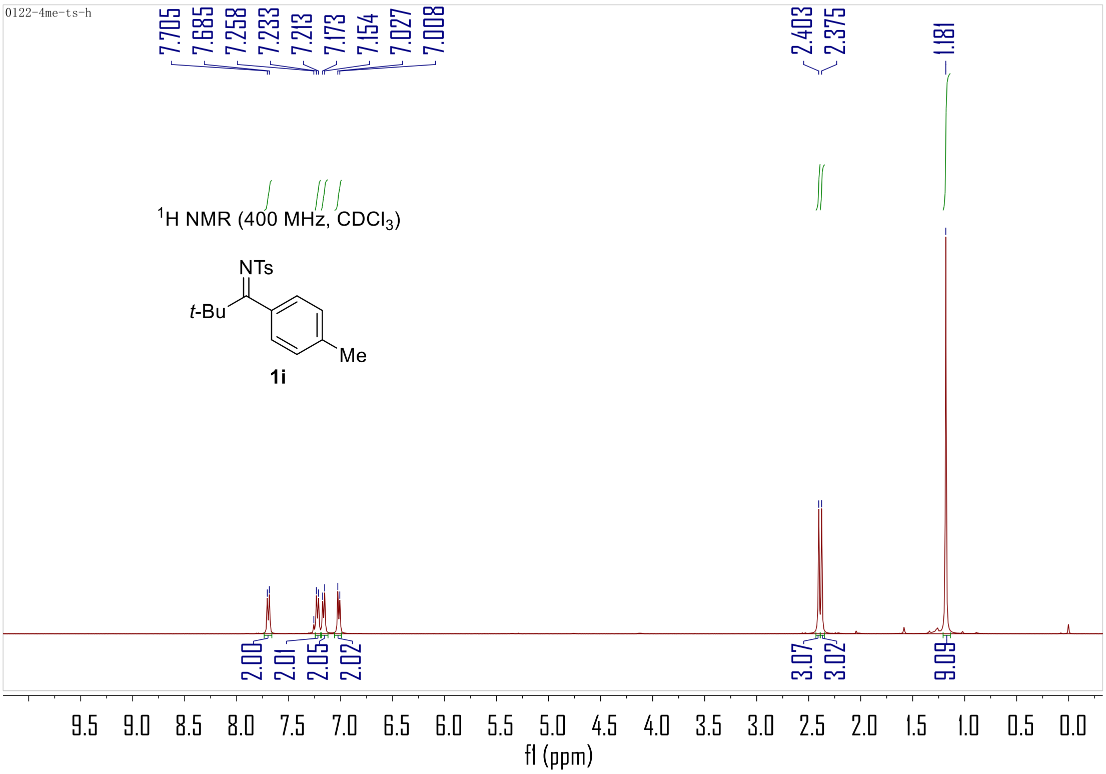

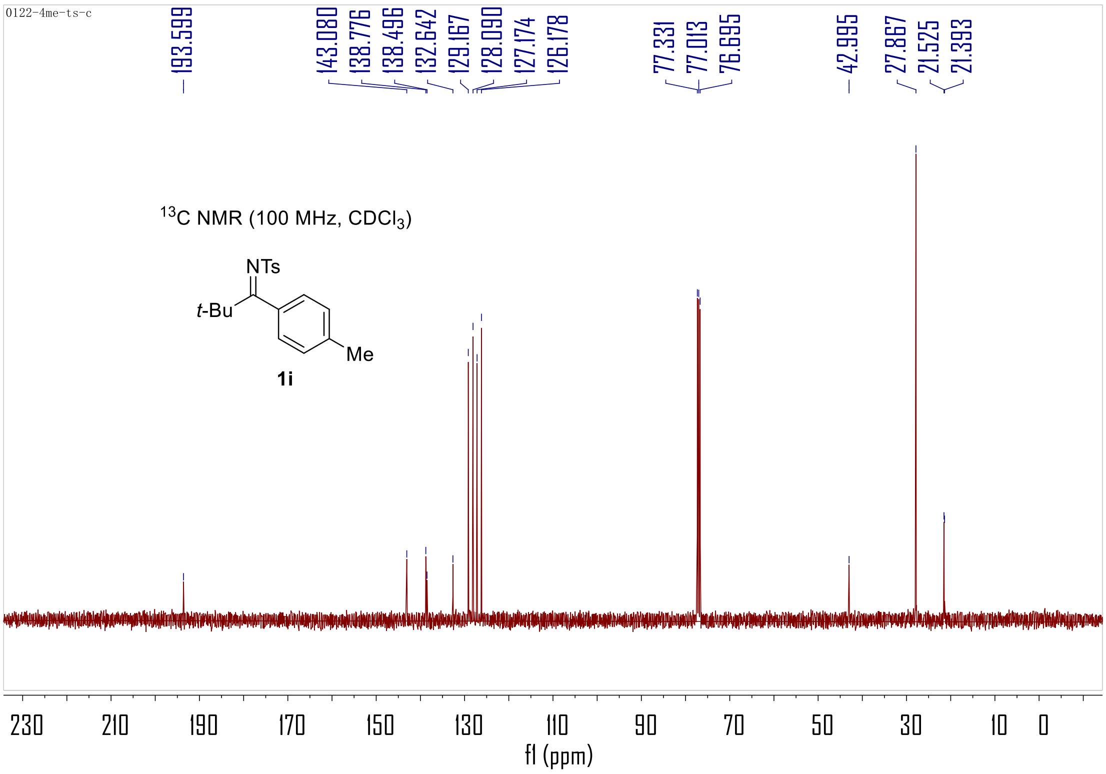

Supplementary Figure 4 ^{1}H and ^{13}C NMR spectrum of compound **1i** in $CDCl_3$

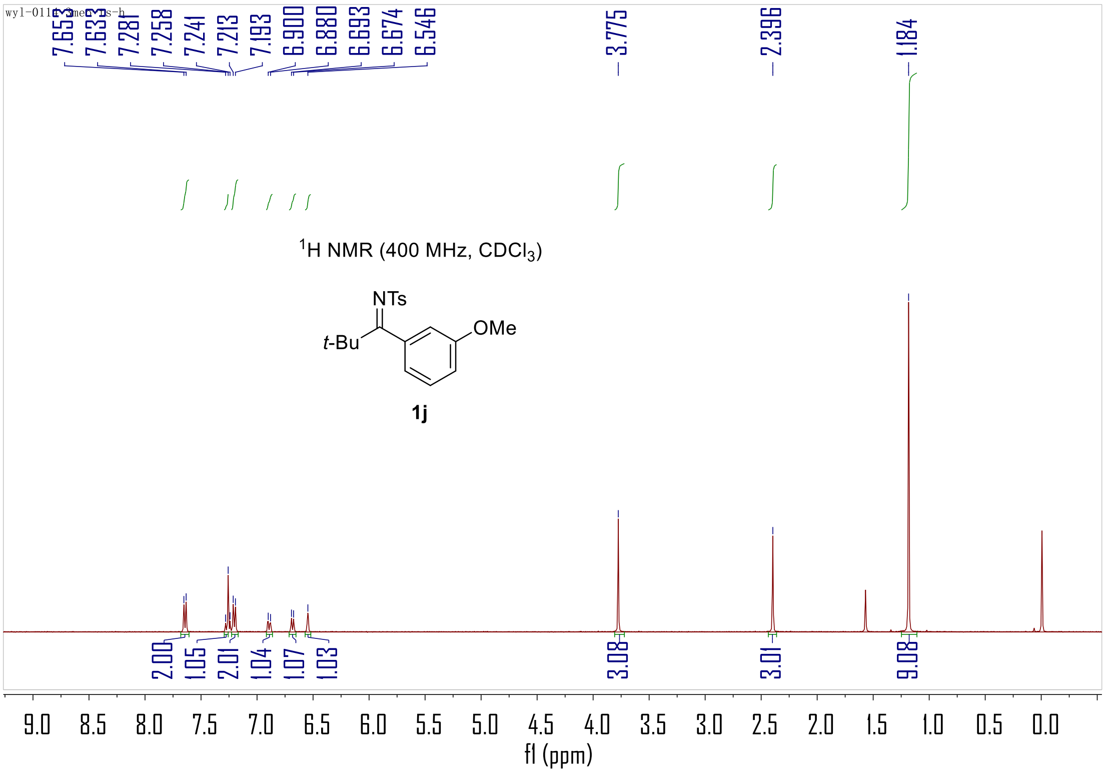

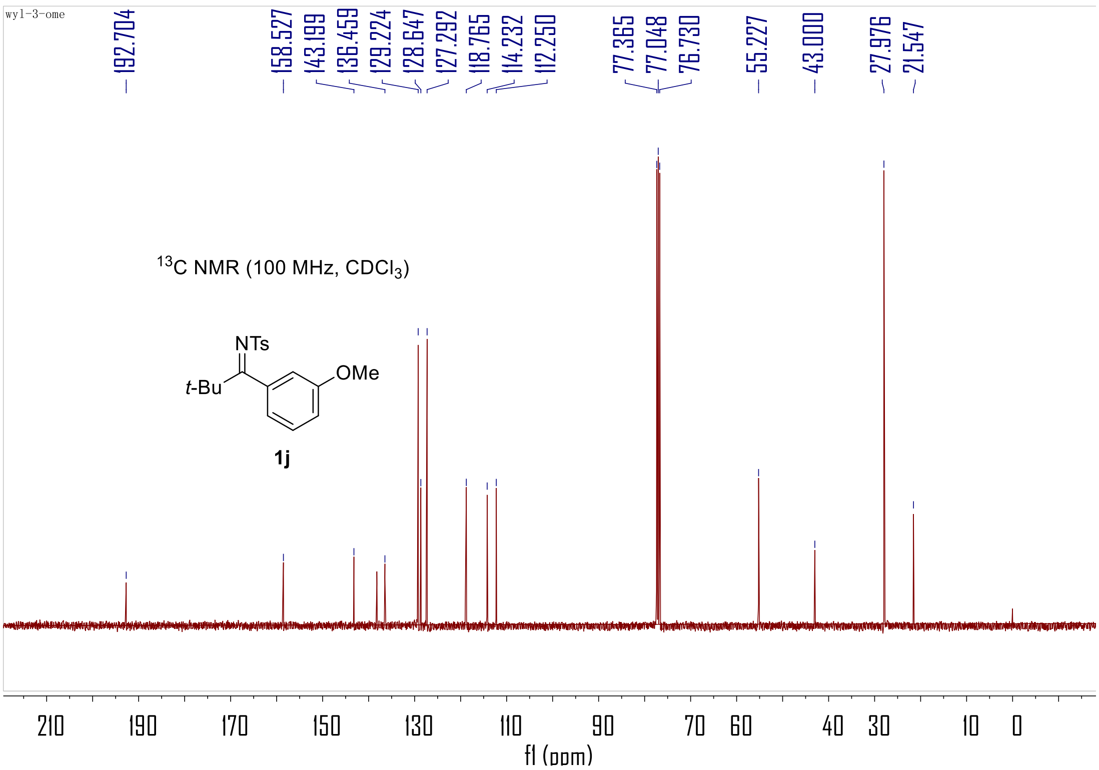

Supplementary Figure 5 ^{1}H and ^{13}C NMR spectrum of compound **1j** in CDCl3

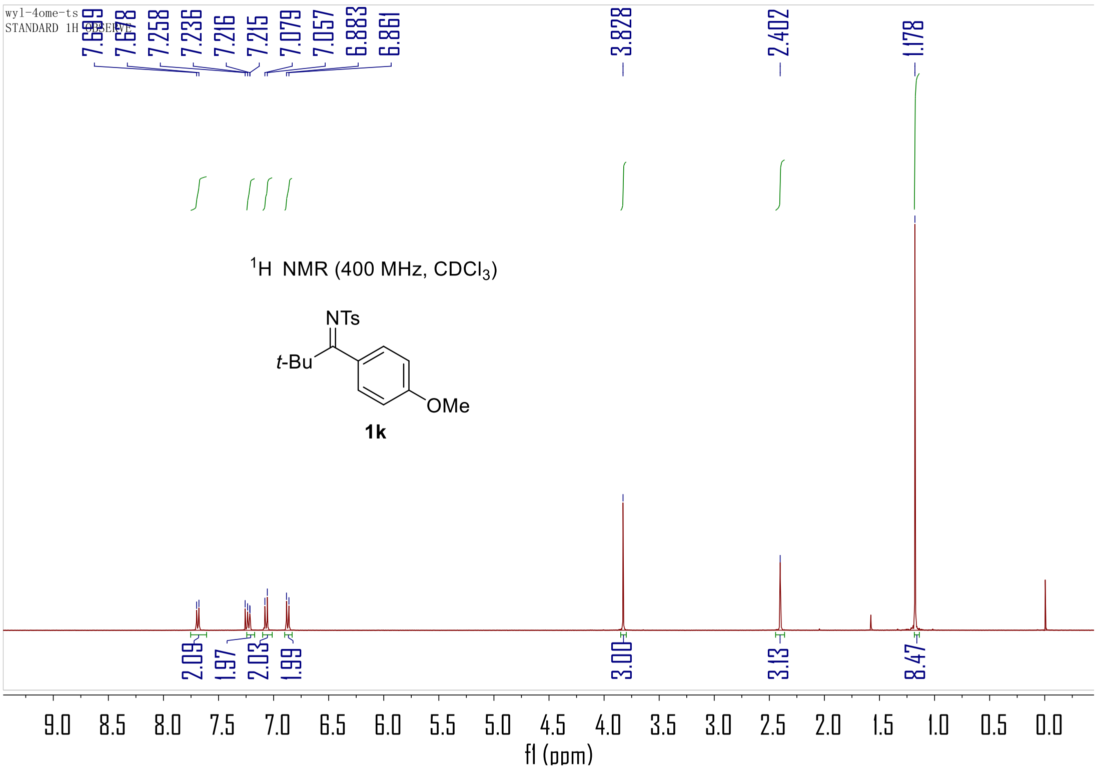

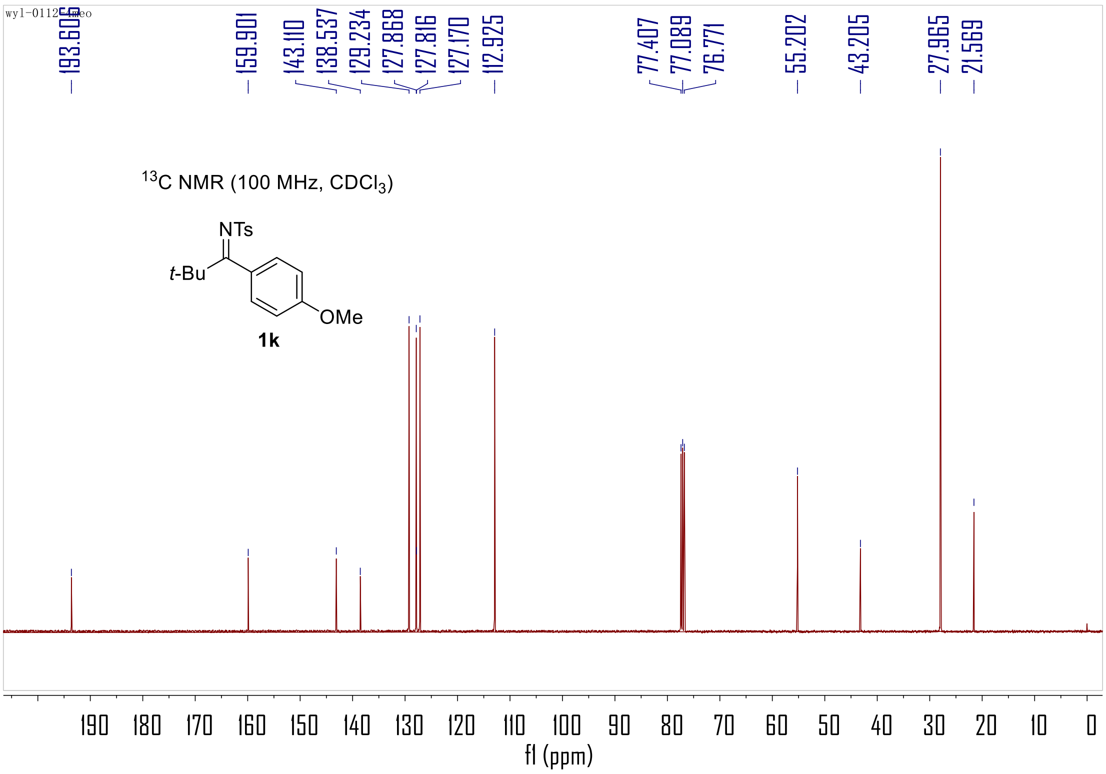

Supplementary Figure 6 ^{1}H and ^{13}C NMR spectrum of compound **1k** in $CDCl_3$

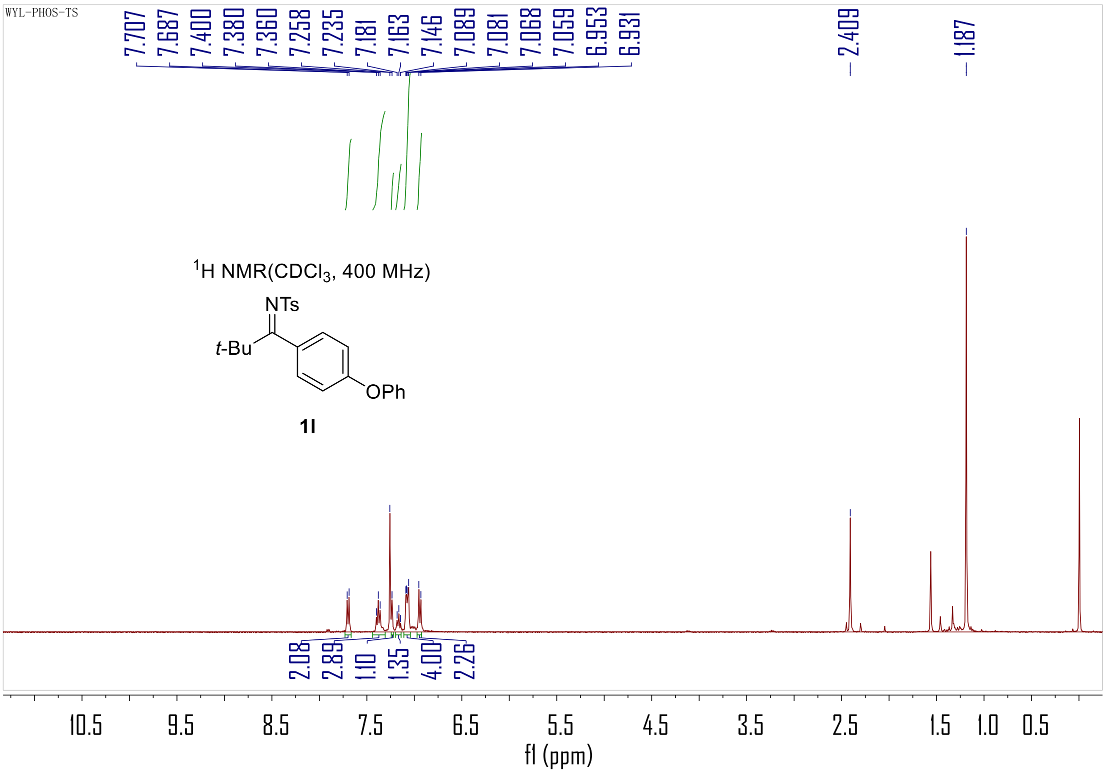

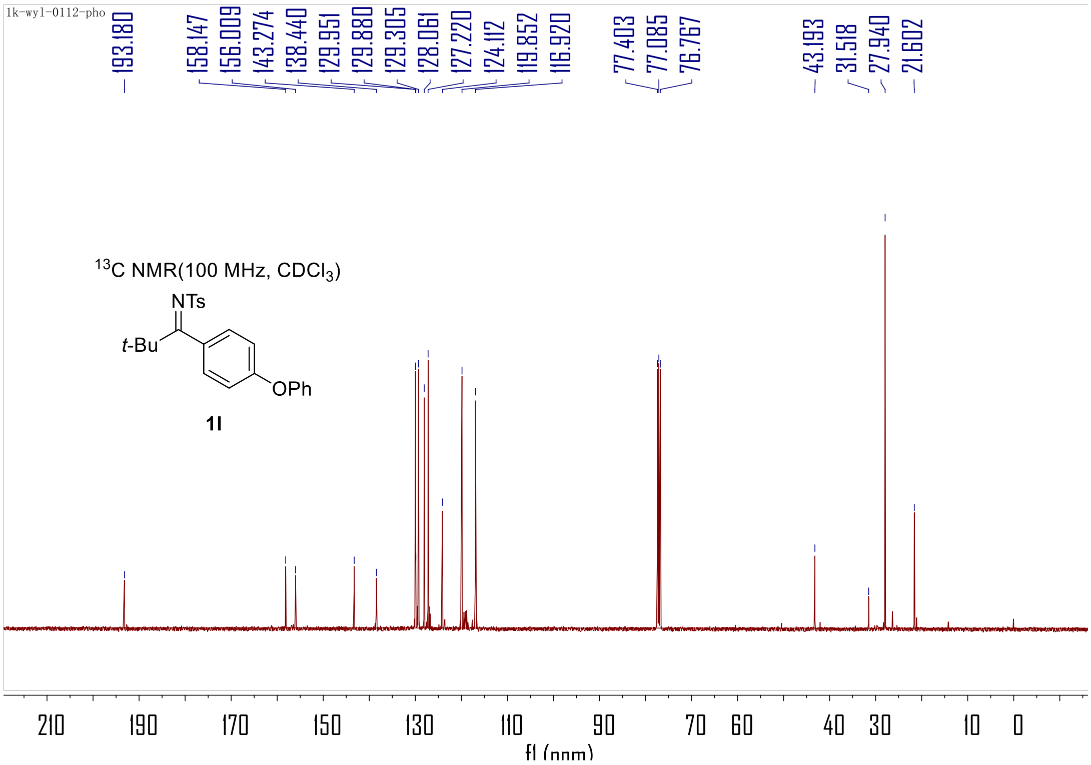

Supplementary Figure 7 ^{1}H and ^{13}C NMR spectrum of compound **1l** in CDCl3

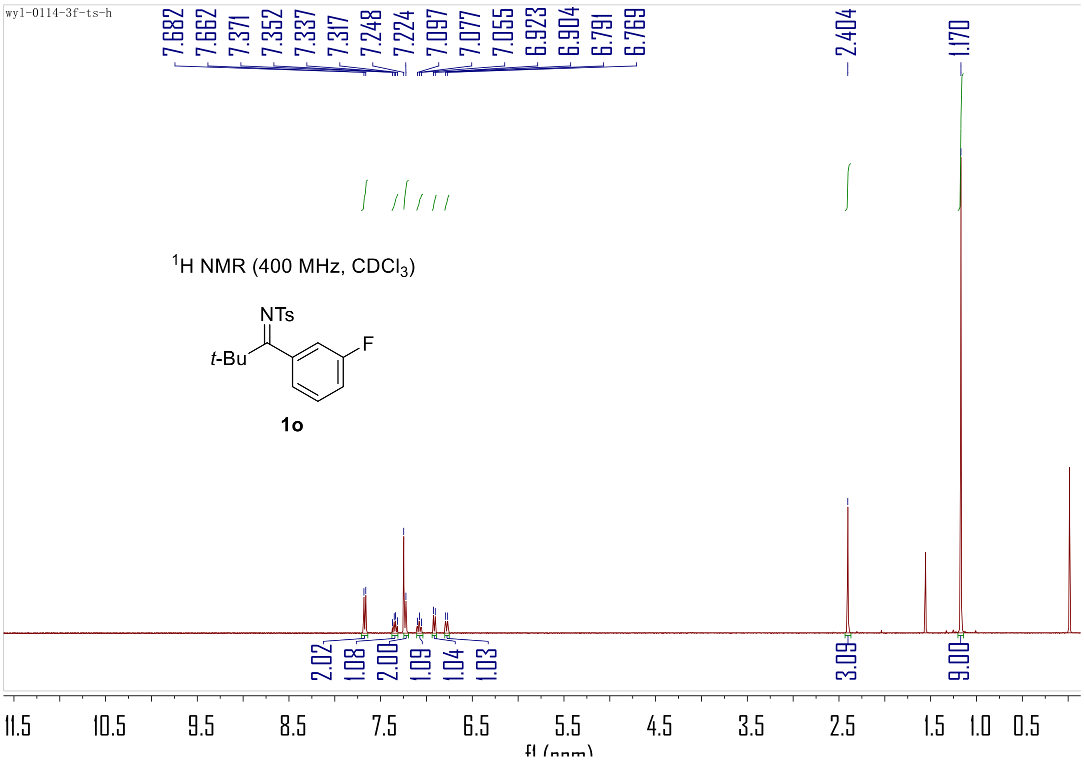

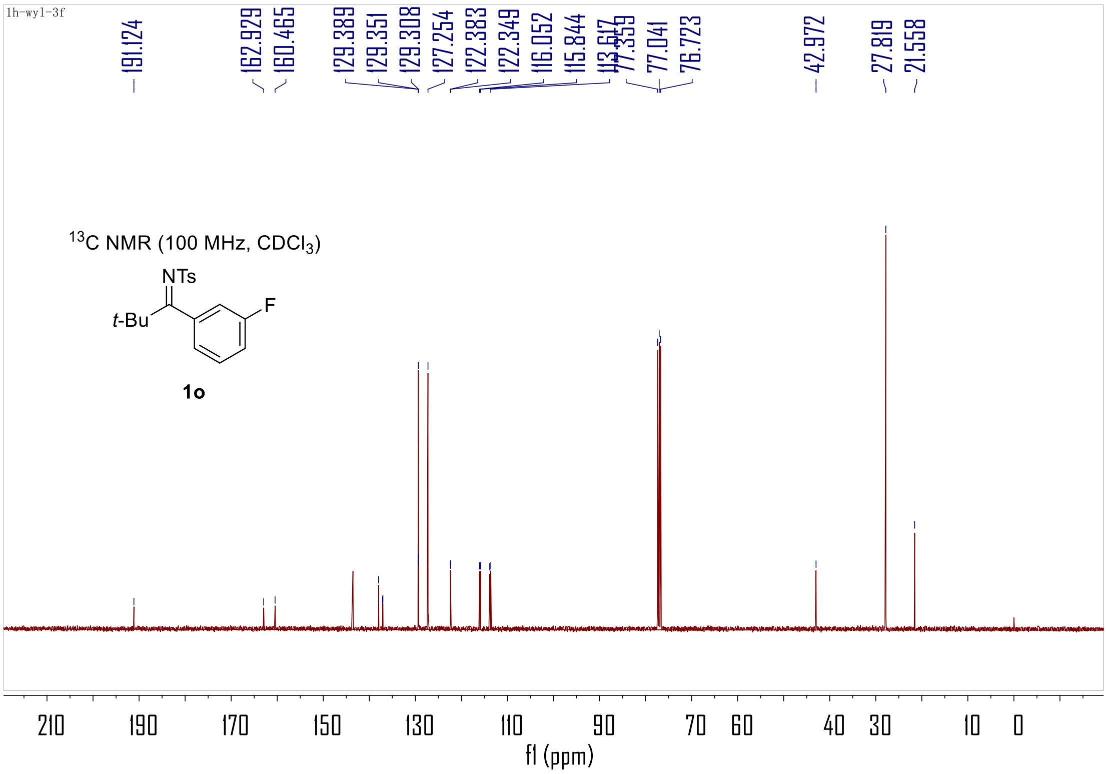

Supplementary Figure 8 ^{1}H and ^{13}C NMR spectrum of compound **1o** in CDCl3

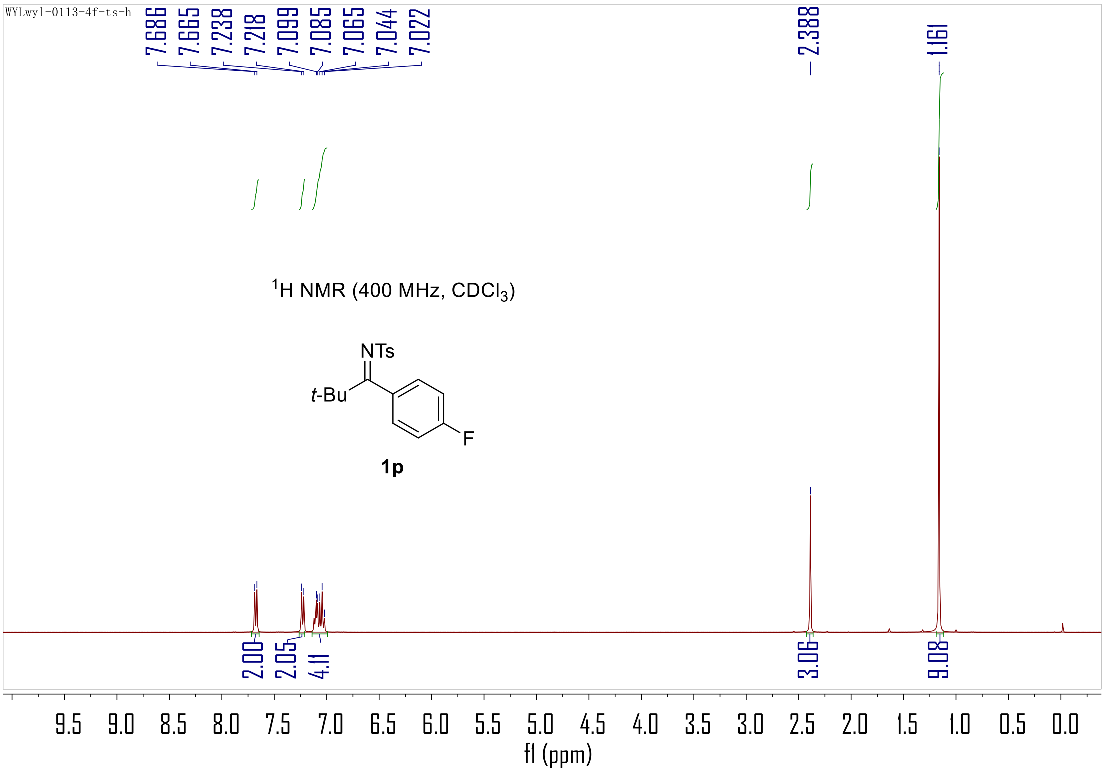

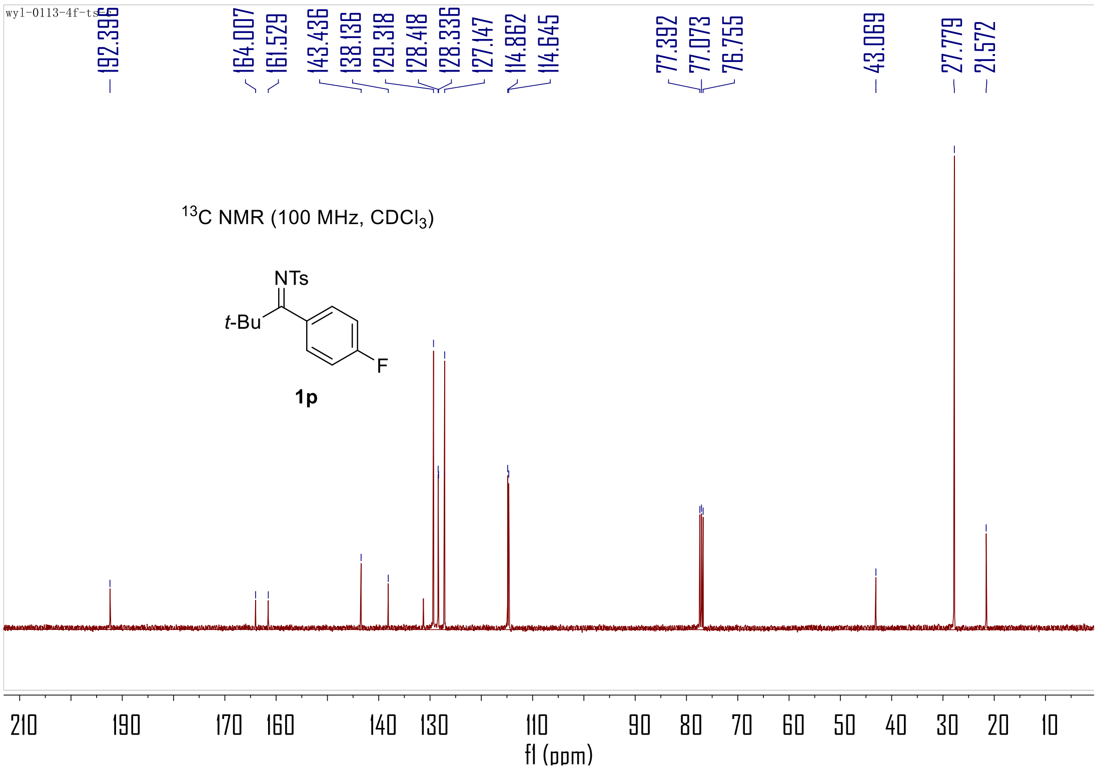

Supplementary Figure 9 ^{1}H and ^{13}C NMR spectrum of compound **1p** in $CDCl_3$

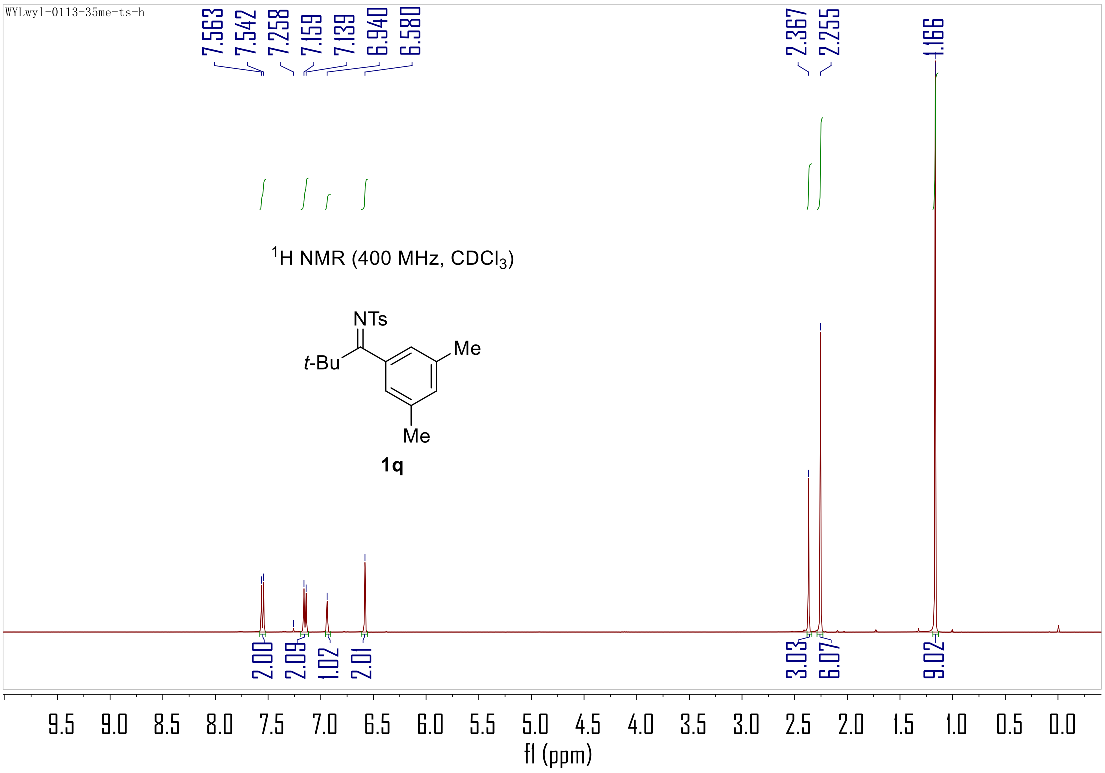

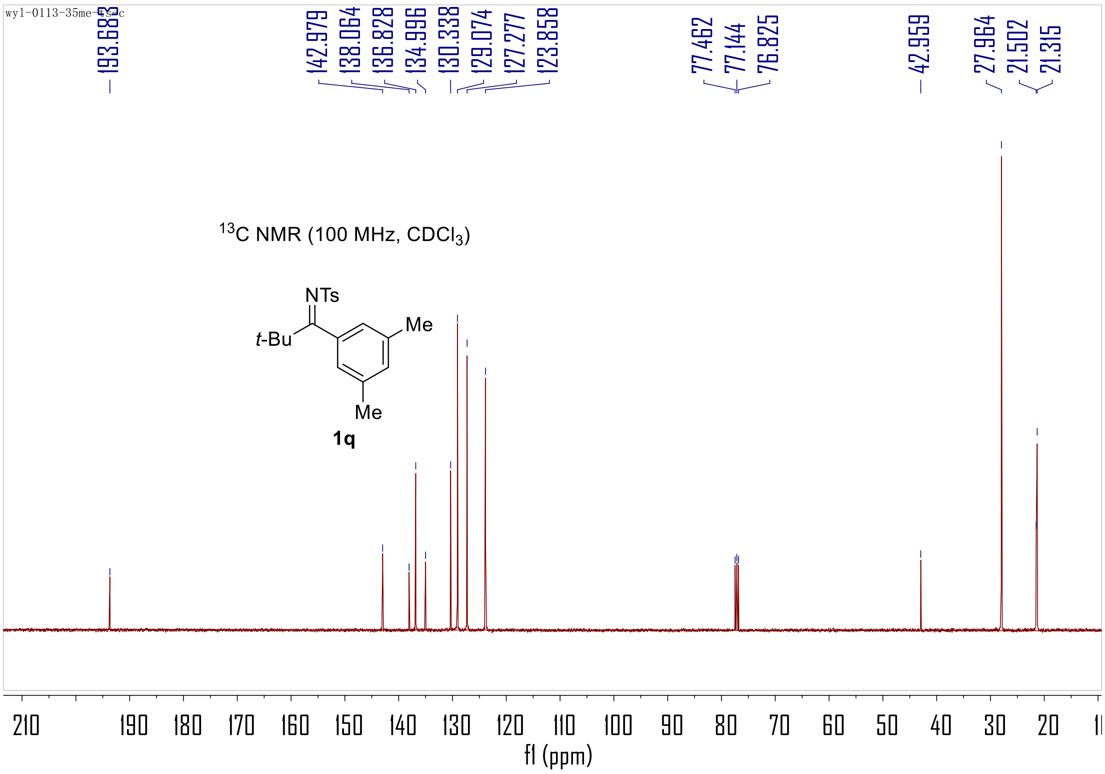

Supplementary Figure 10 ^{1}H and ^{13}C NMR spectrum of compound **1q**in $CDCl_3$

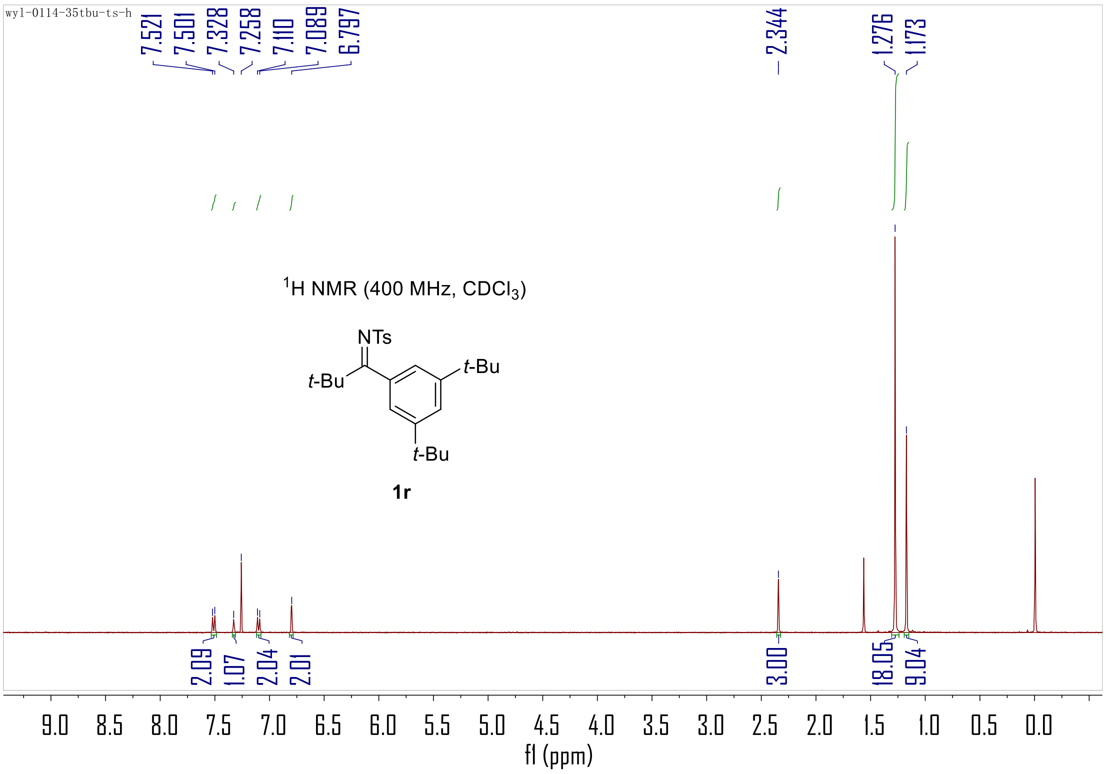

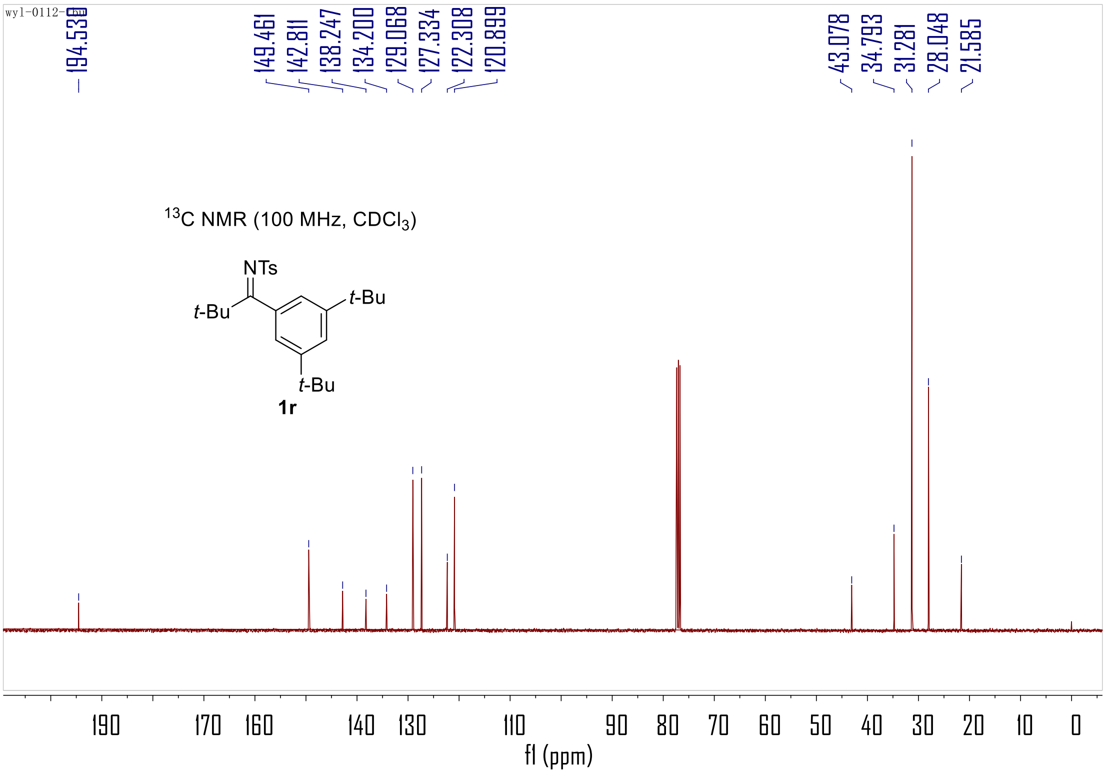

Supplementary Figure 11 ^{1}H and ^{13}C NMR spectrum of compound **1r** in $CDCl_3$

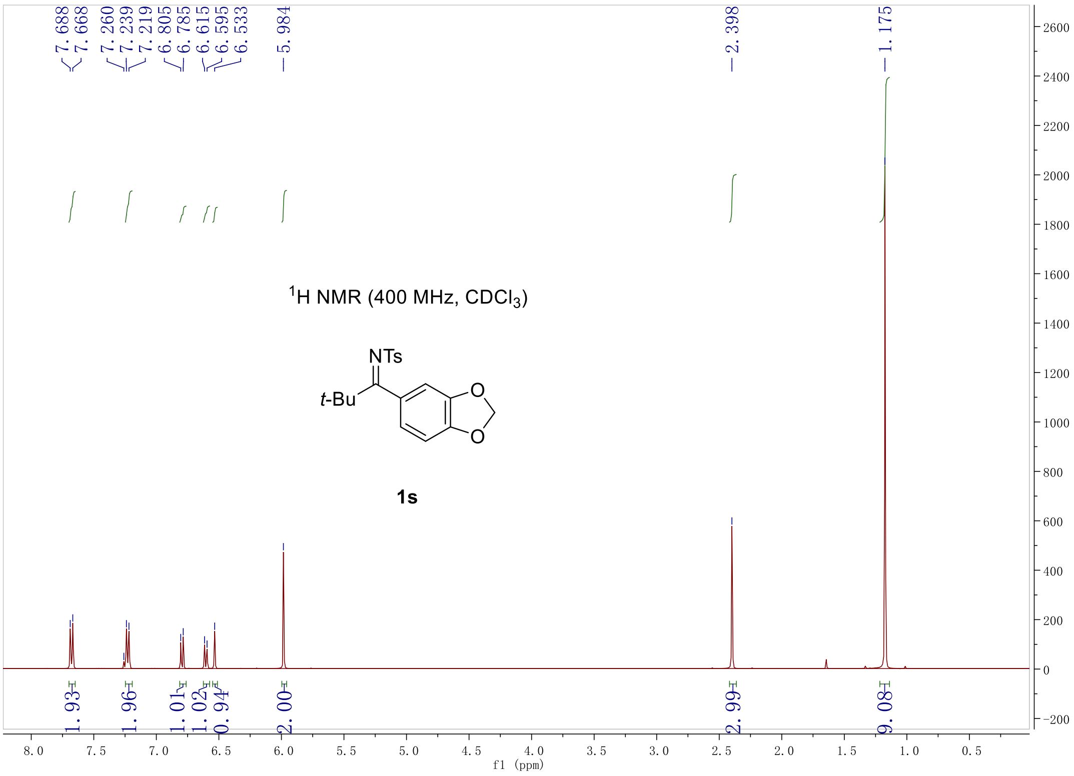

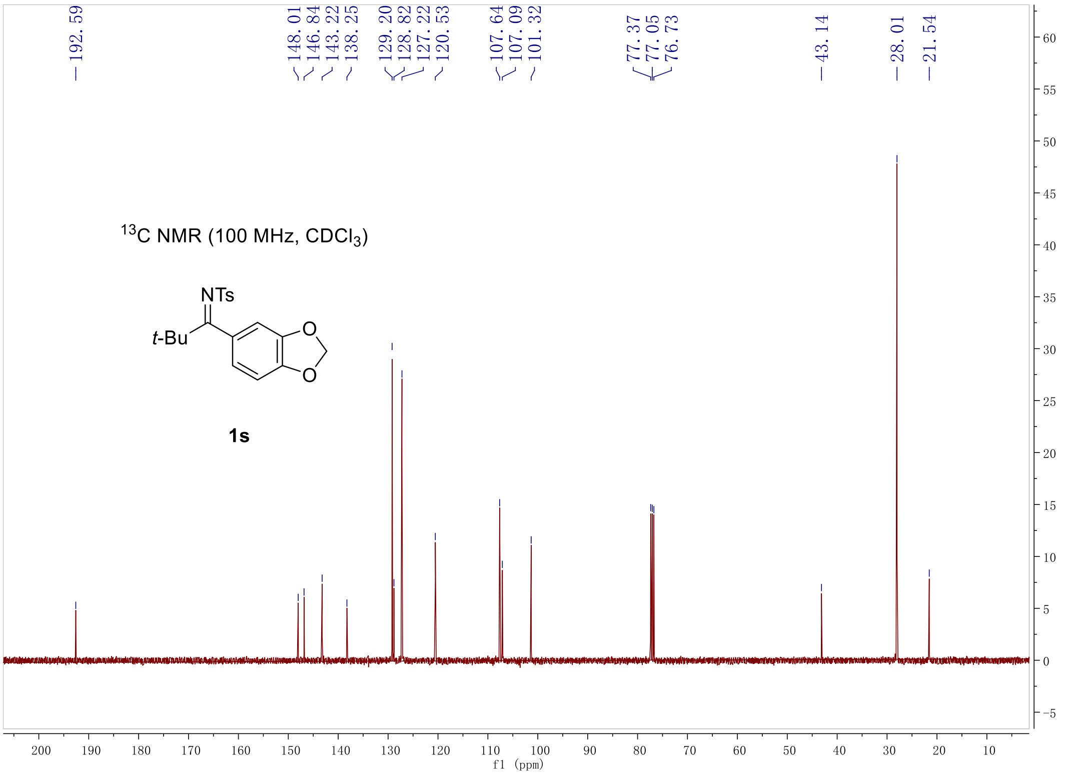

Supplementary Figure 12 ^{1}H and ^{13}C NMR spectrum of compound **1s** in $CDCl_3$

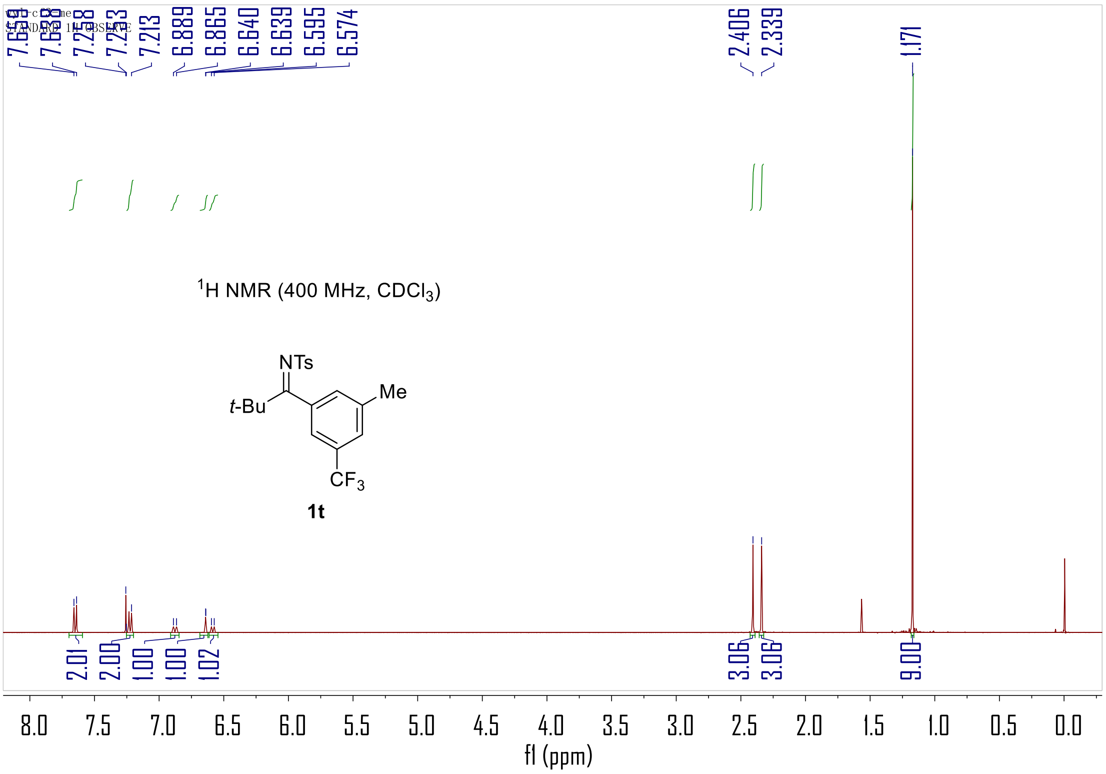

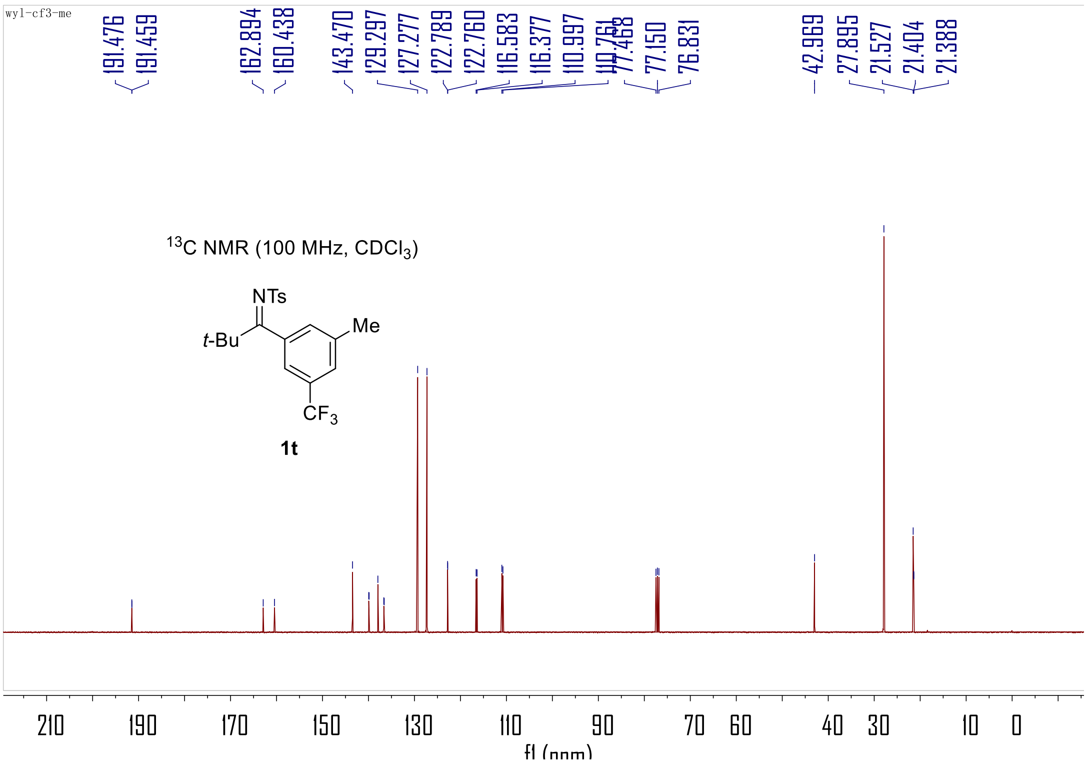

Supplementary Figure 13 ^{1}H and ^{13}C NMR spectrum of compound **1t** in $CDCl_3$

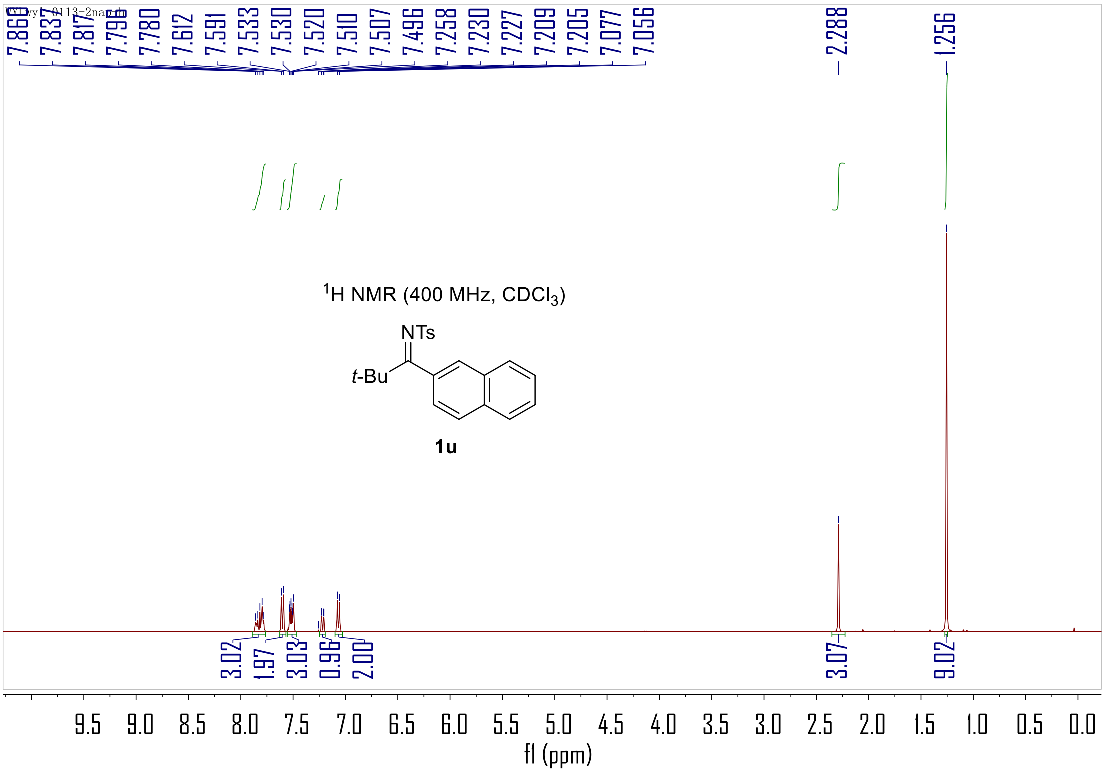

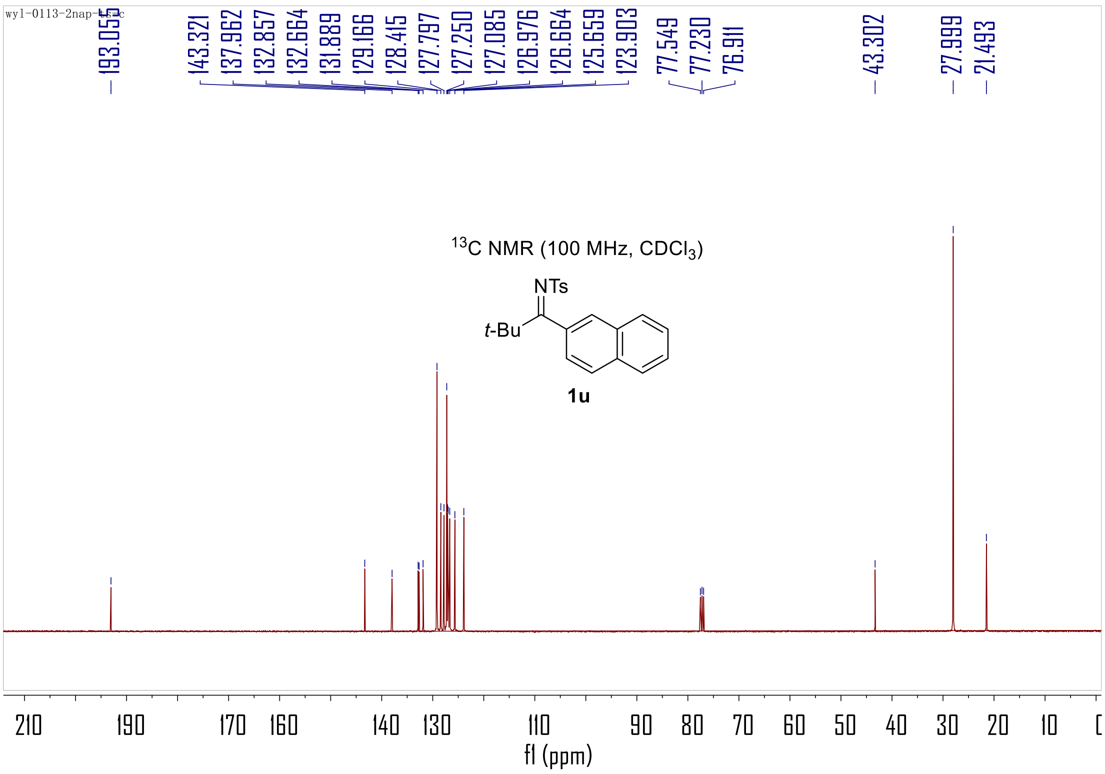

Supplementary Figure 14 ^{1}H and ^{13}C NMR spectrum of compound **1u** in CDCl3

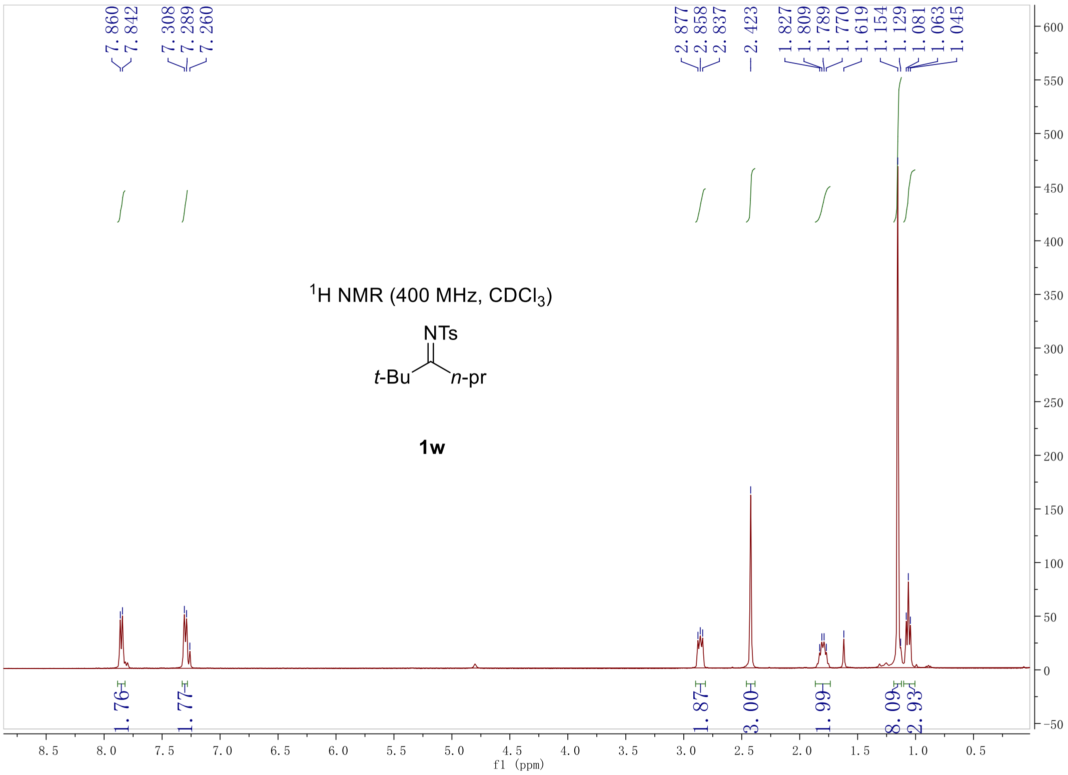

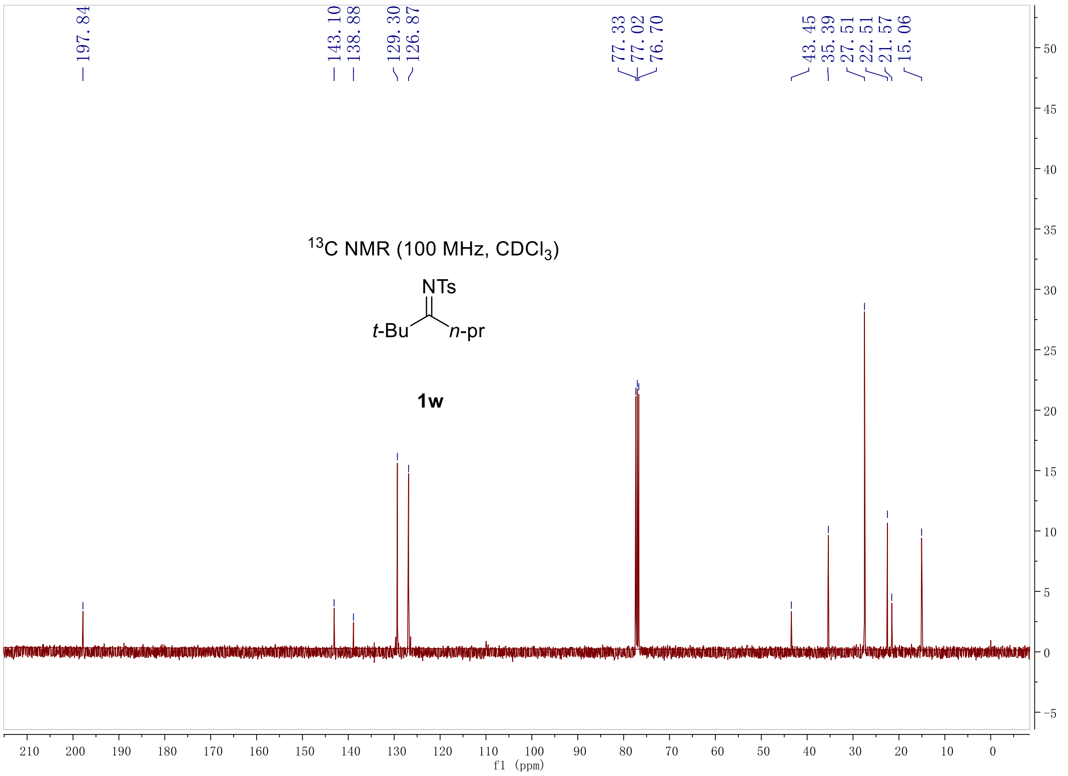

Supplementary Figure 15 ^{1}H and ^{13}C NMR spectrum of compound **1w** in CDCl3

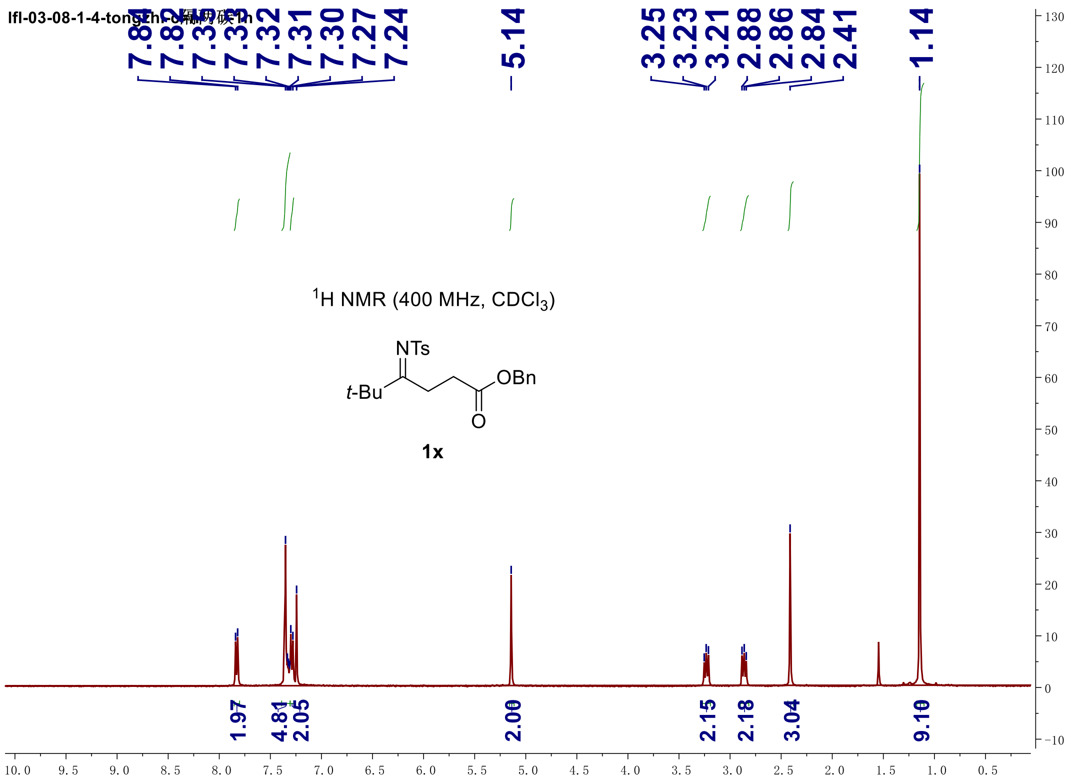

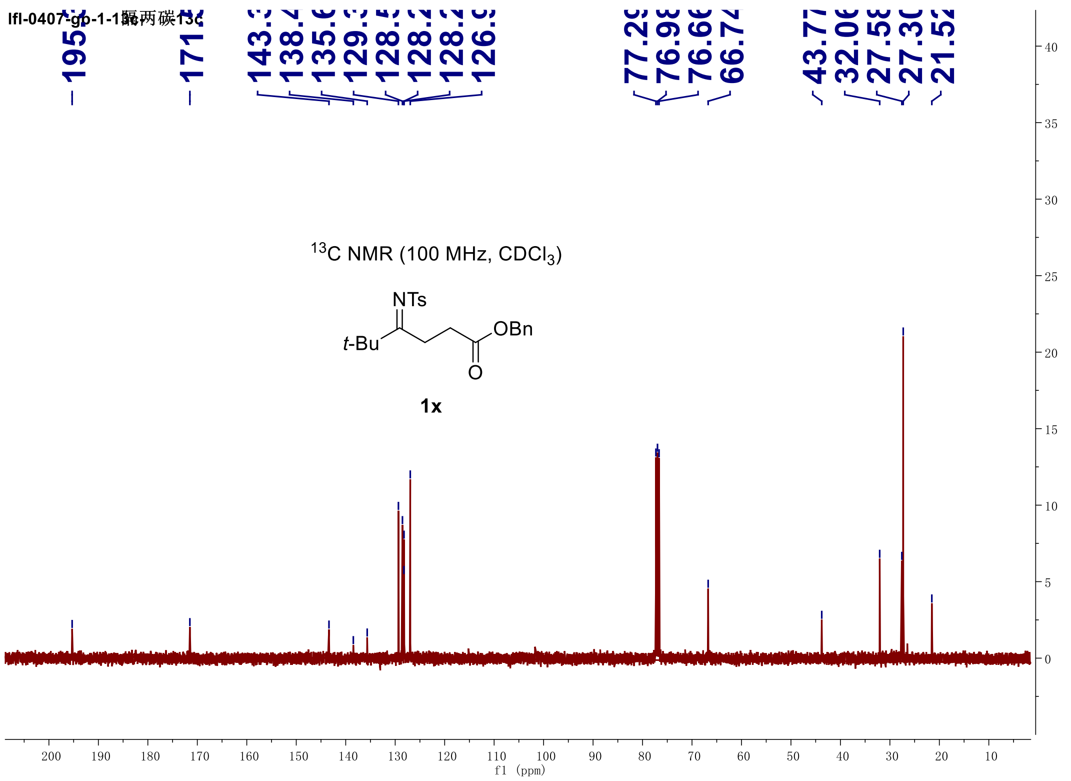

Supplementary Figure 16 ^{1}H and ^{13}C NMR spectrum of compound **1x** in CDCl3

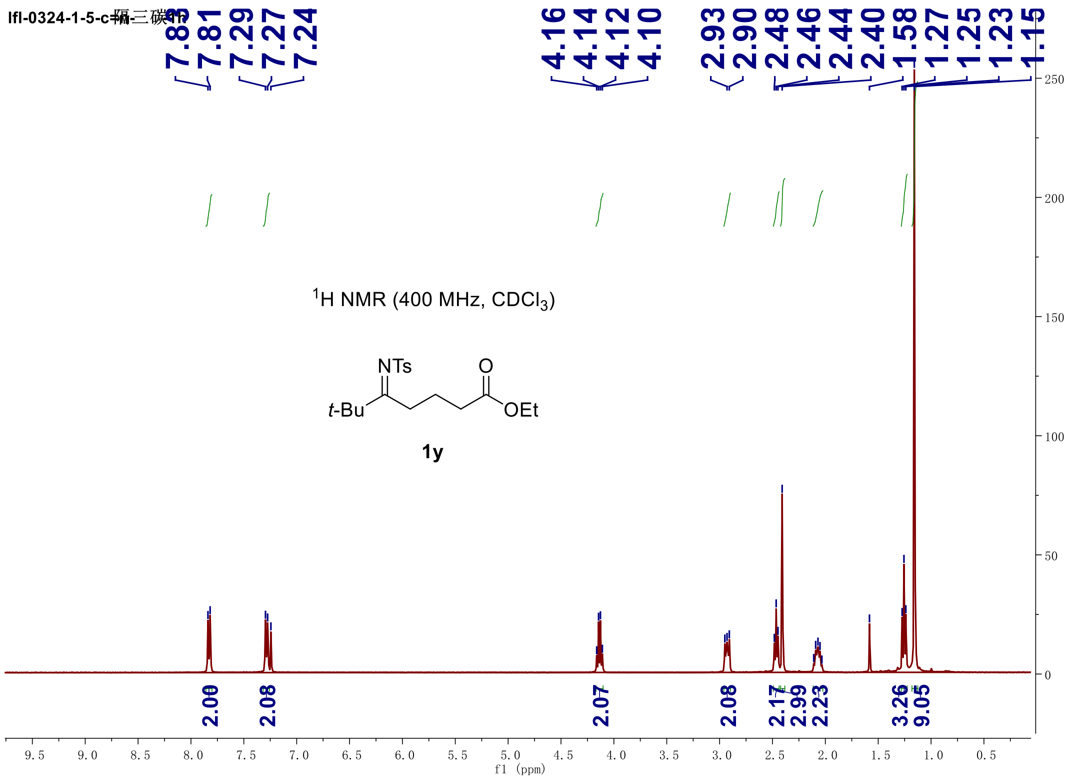

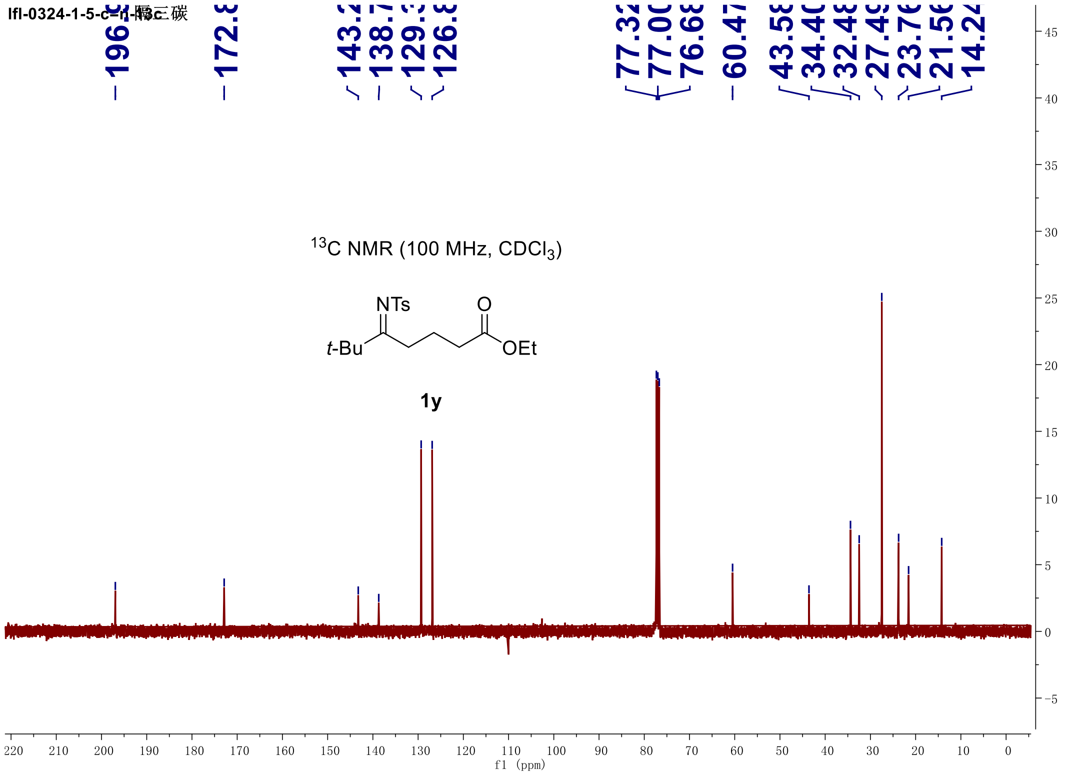

Supplementary Figure 17 ^{1}H and ^{13}C NMR spectrum of compound **1y** in $CDCl_3$

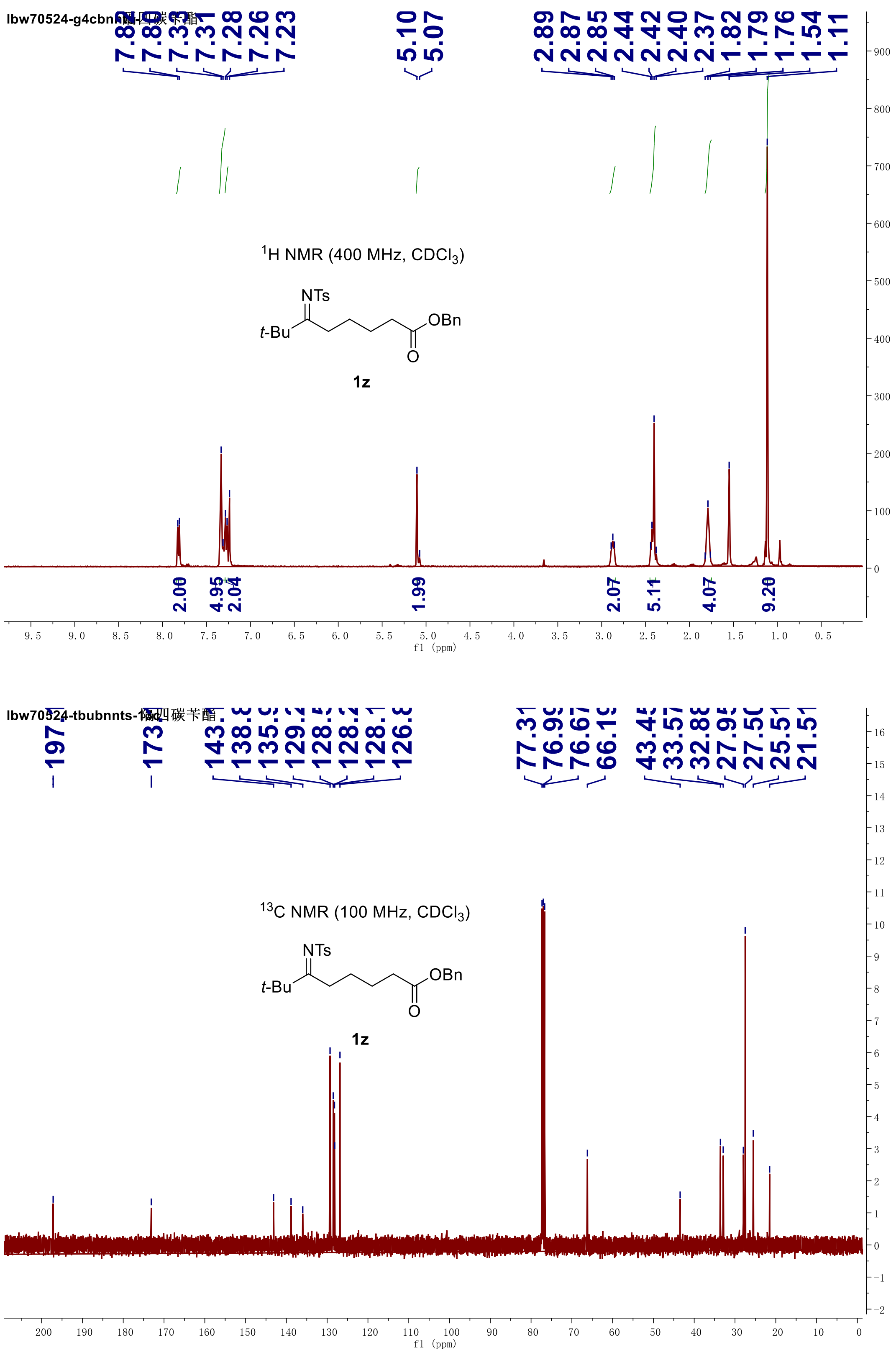

Supplementary Figure 18 ^{1}H and ^{13}C NMR spectrum of compound **1z** in $CDCl_3$

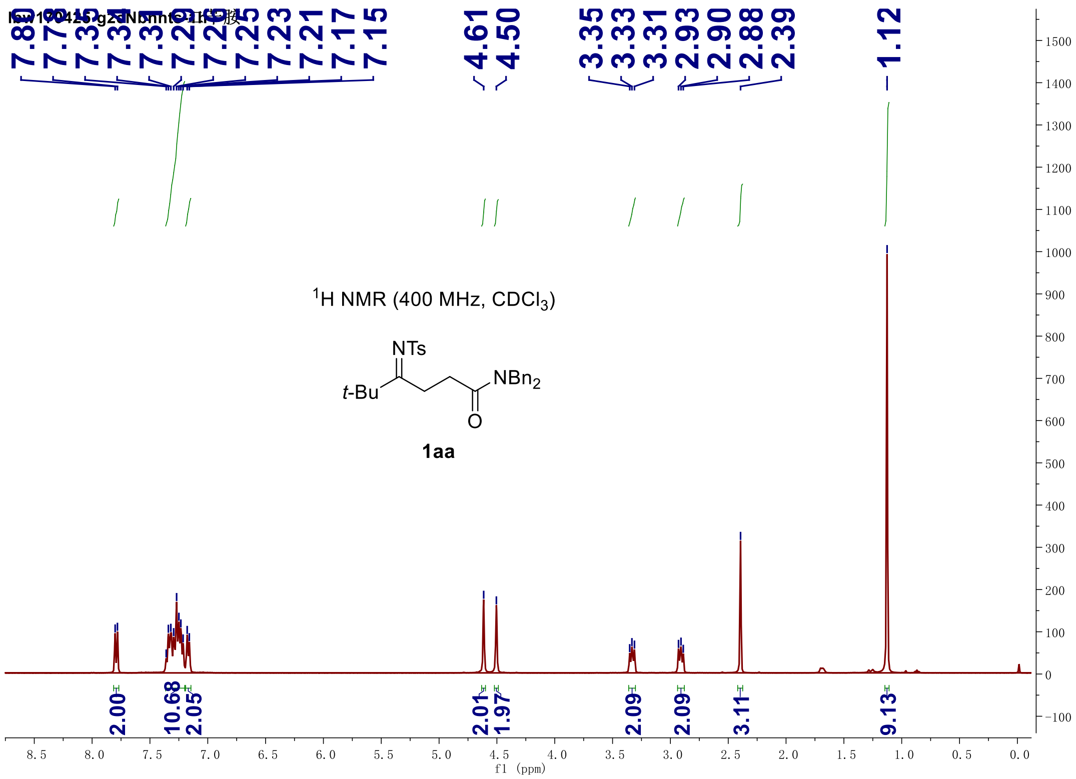

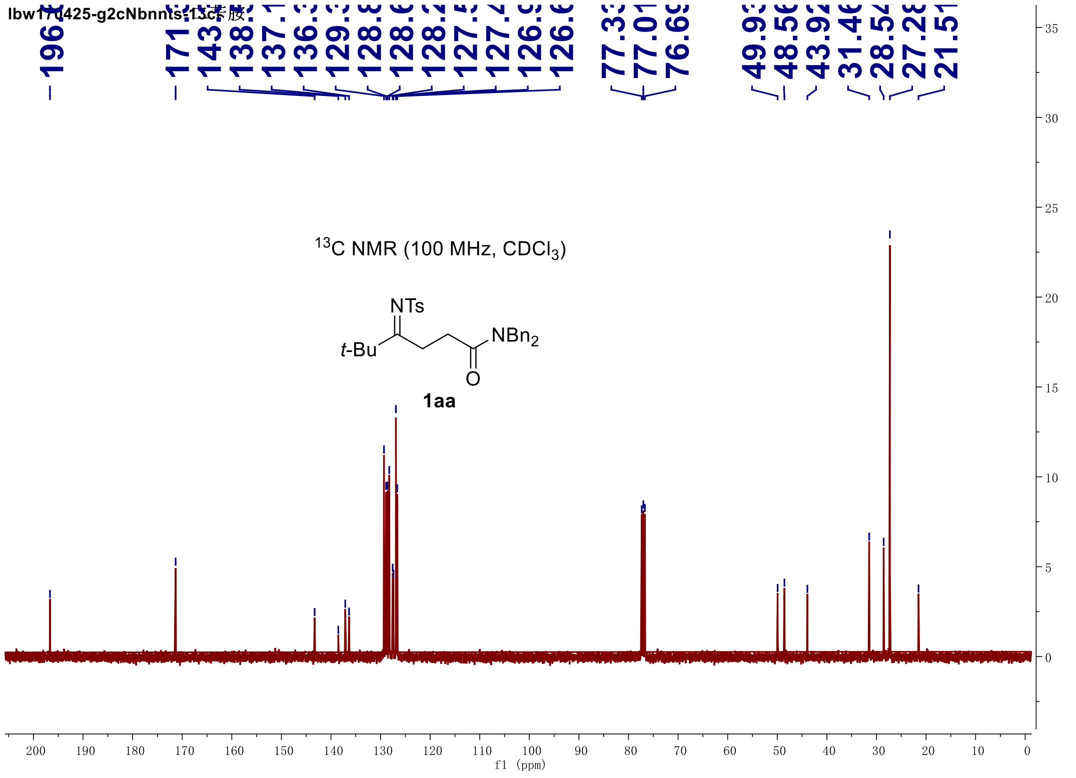

Supplementary Figure 19 ^{1}H and ^{13}C NMR spectrum of compound **1aa** in CDCl3

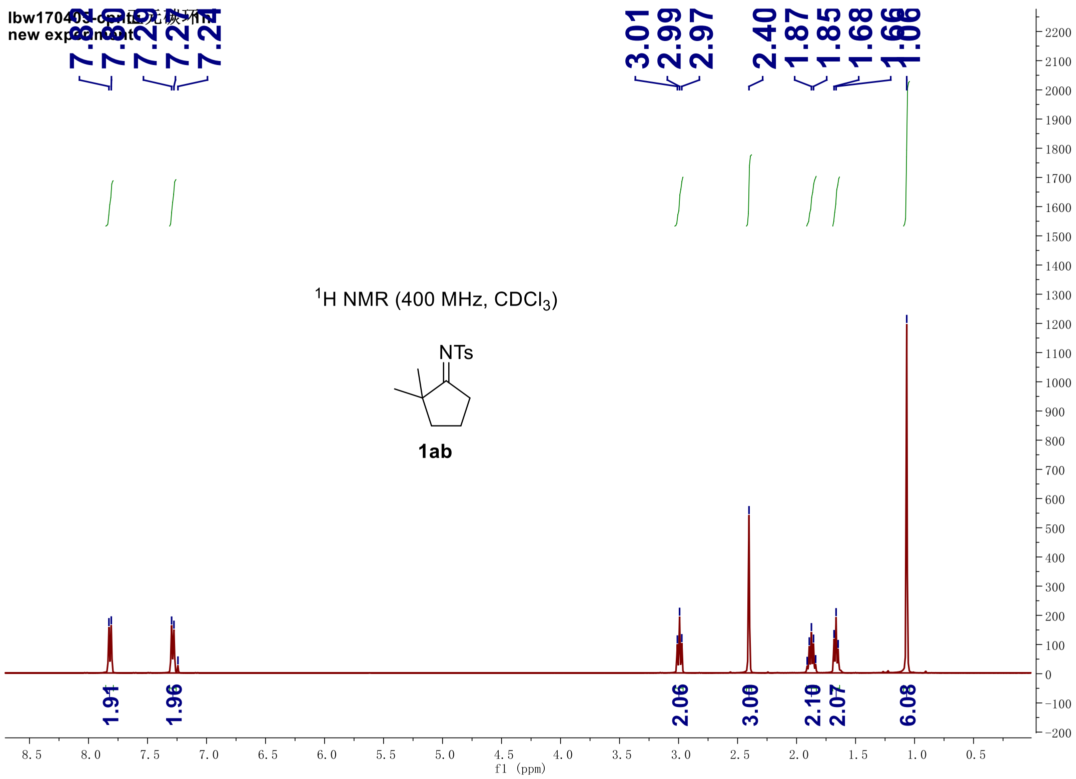

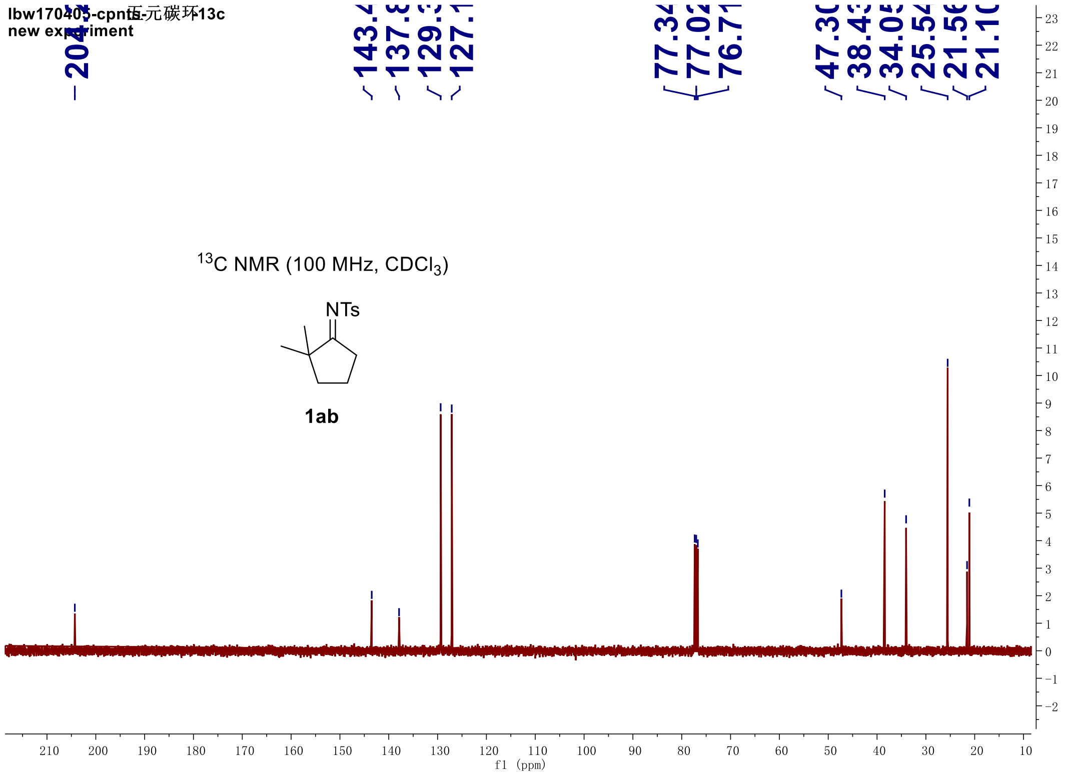

Supplementary Figure 20 ^{1}H and ^{13}C NMR spectrum of compound **1ab** in $CDCl_3$

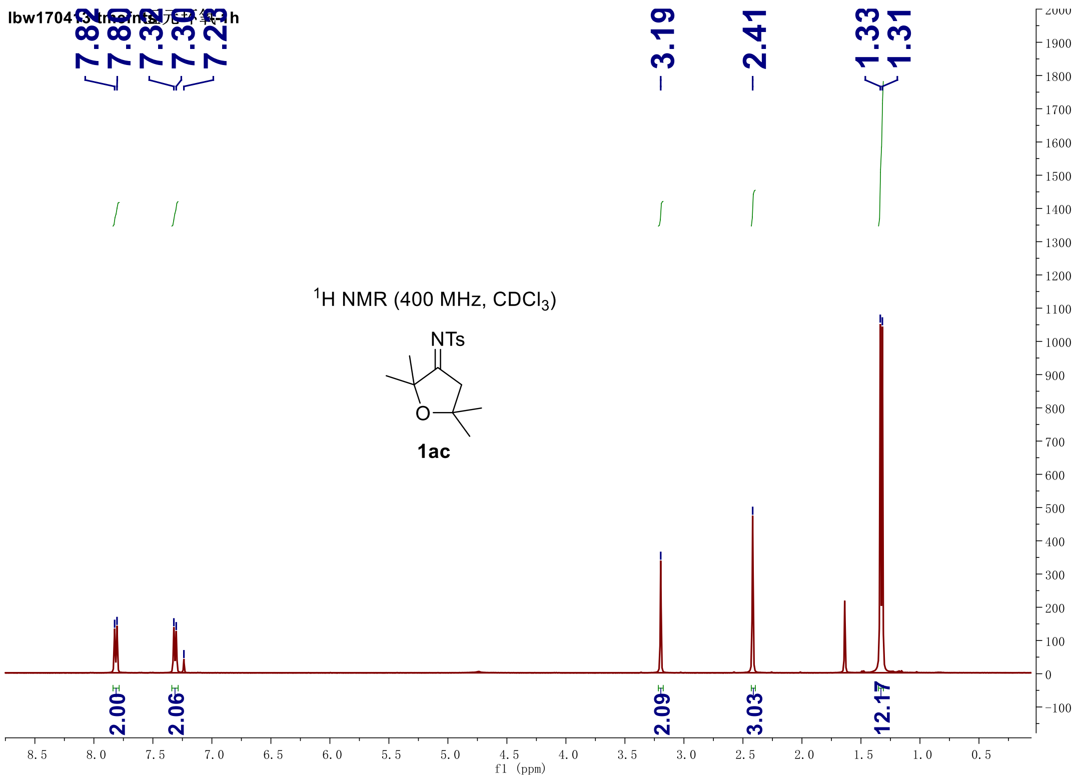

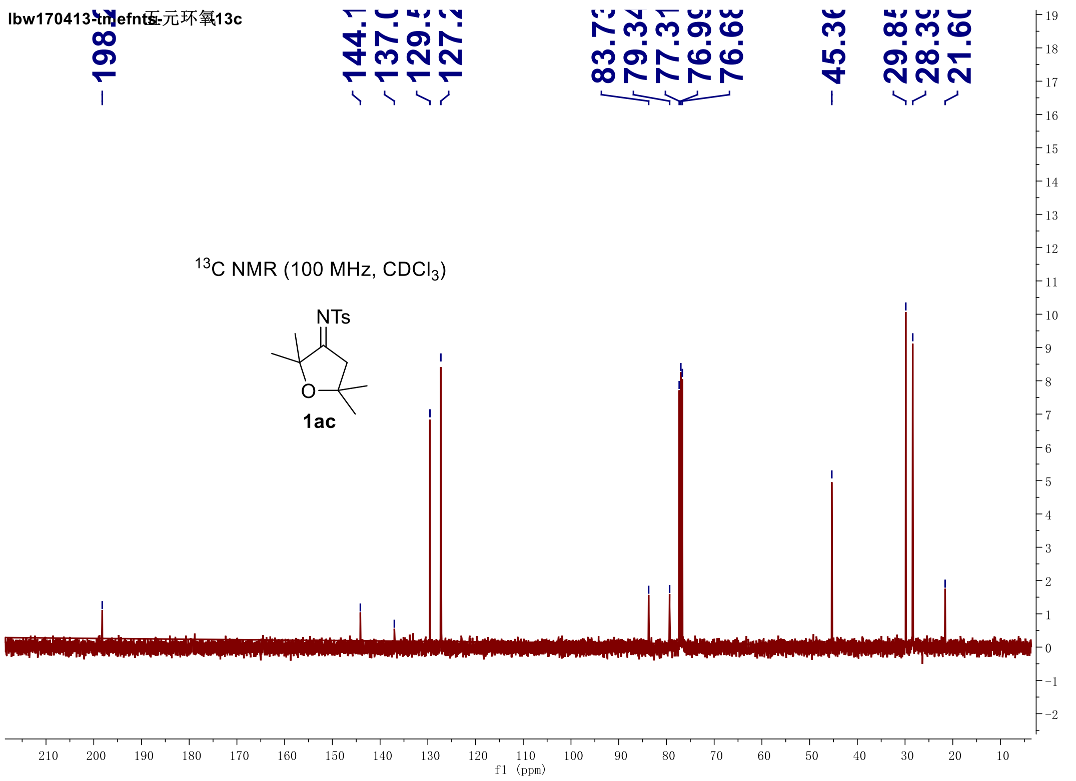

Supplementary Figure 21 ^{1}H and ^{13}C NMR spectrum of compound **1ac** in CDCl3

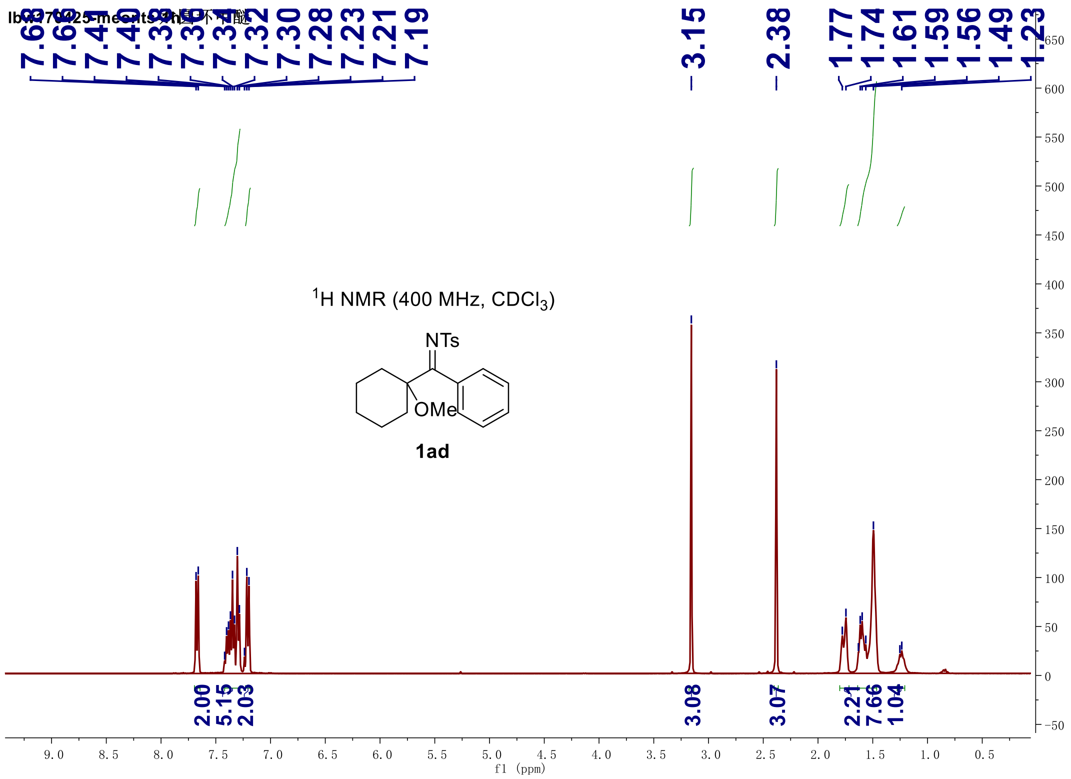

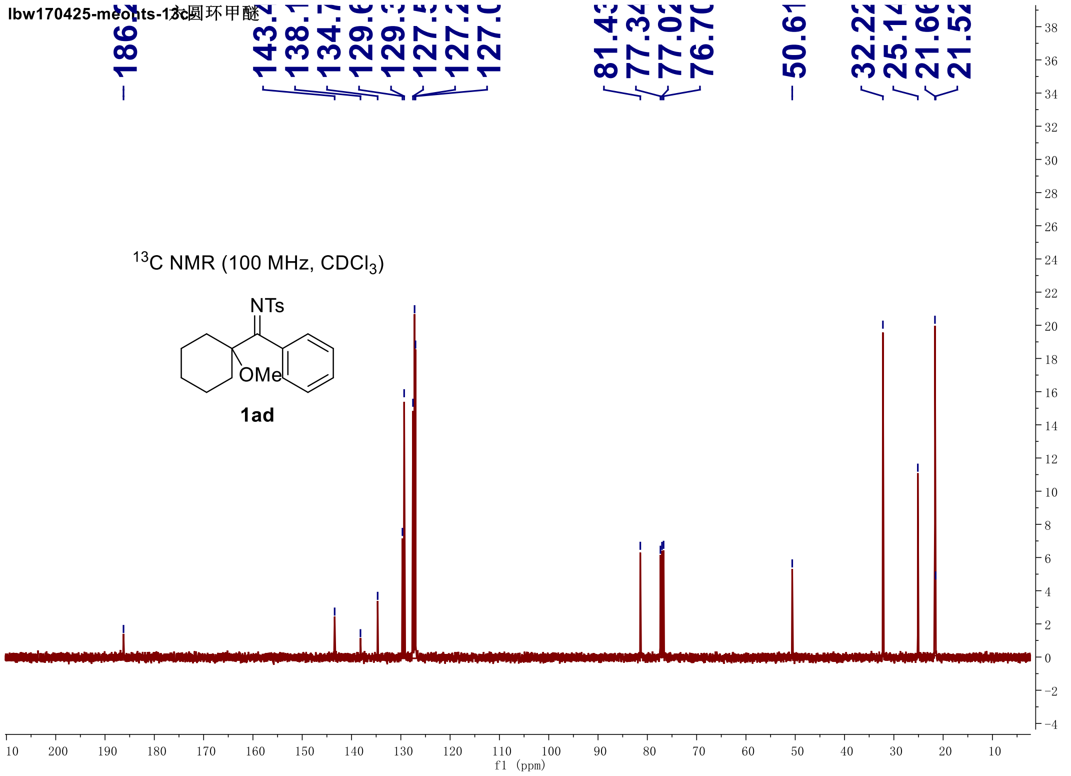

Supplementary Figure 22 ^{1}H and ^{13}C NMR spectrum of compound **1ad** in $CDCl_3$

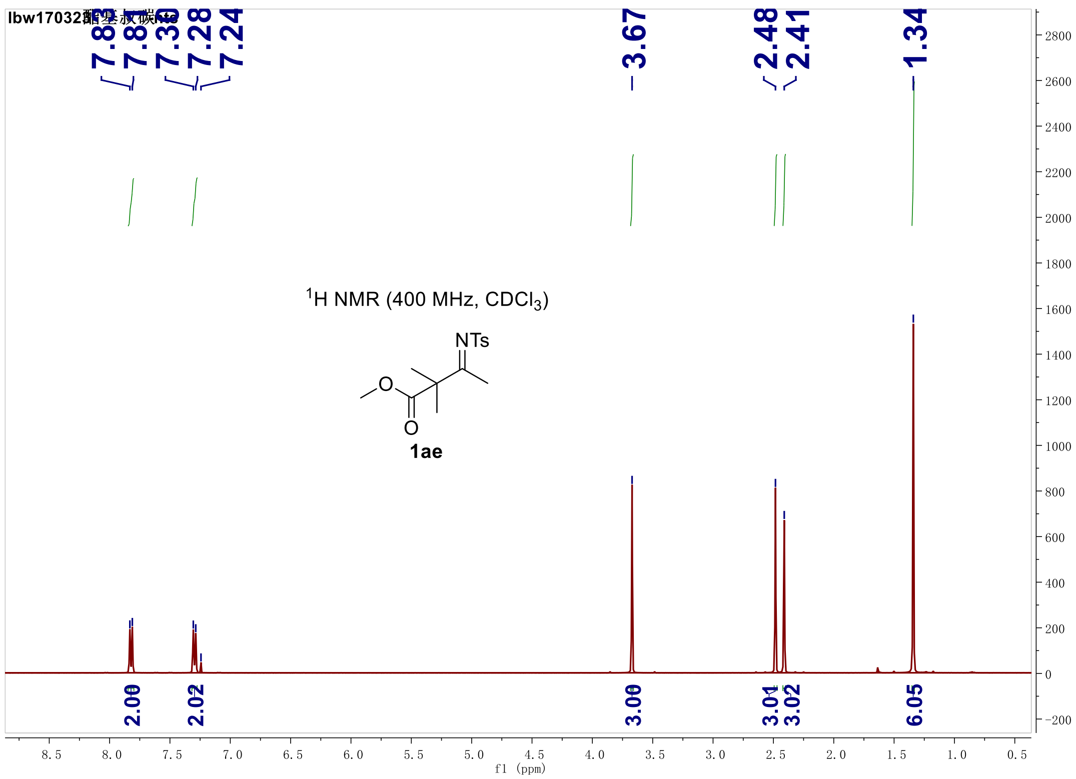

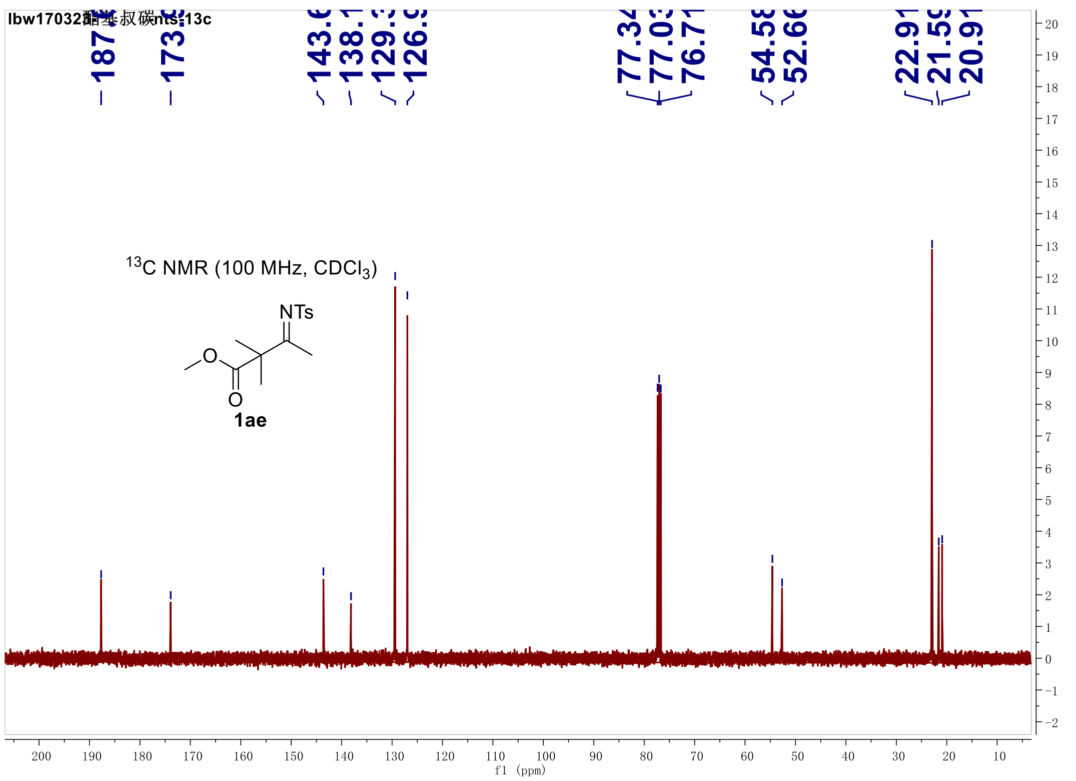

Supplementary Figure 23 ^{1}H and ^{13}C NMR spectrum of compound **1ae** in CDCl3

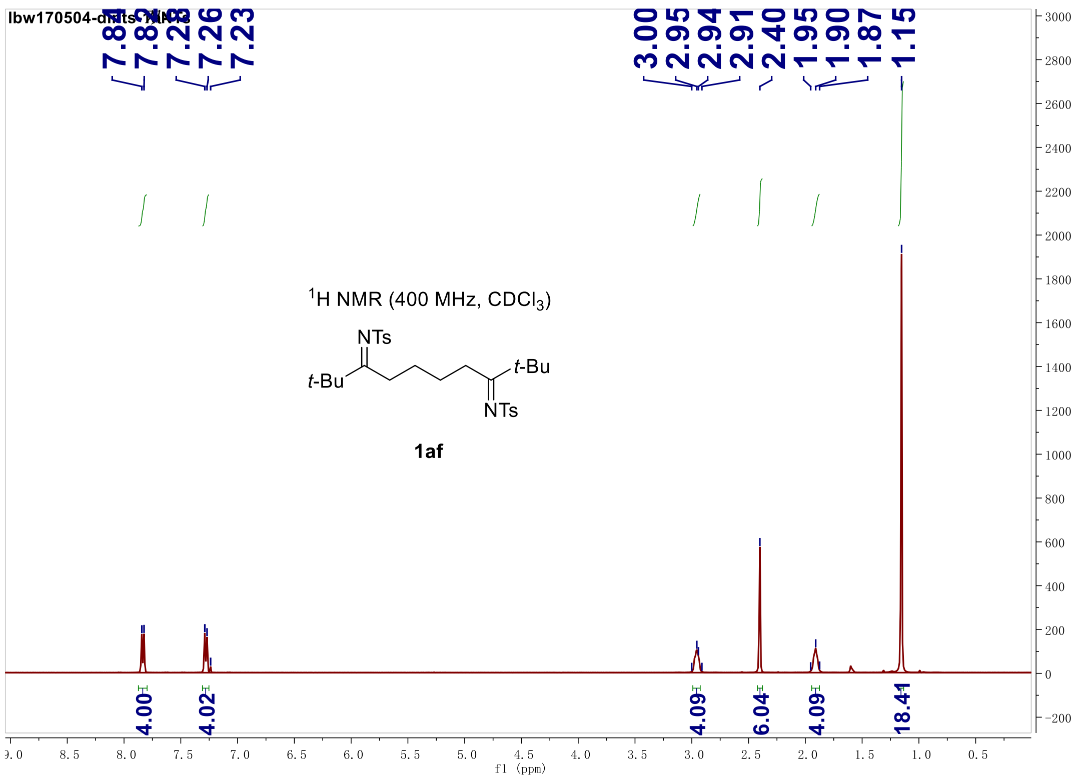

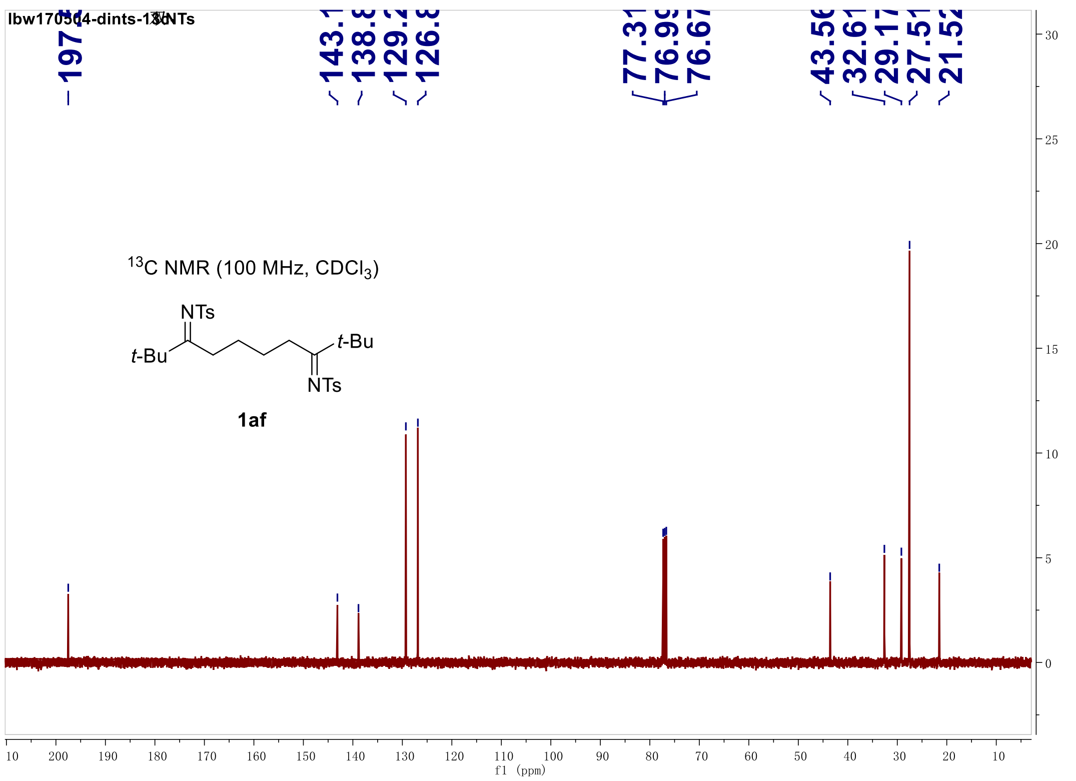

Supplementary Figure 24 ^{1}H and ^{13}C NMR spectrum of compound **1af** in $CDCl_3$

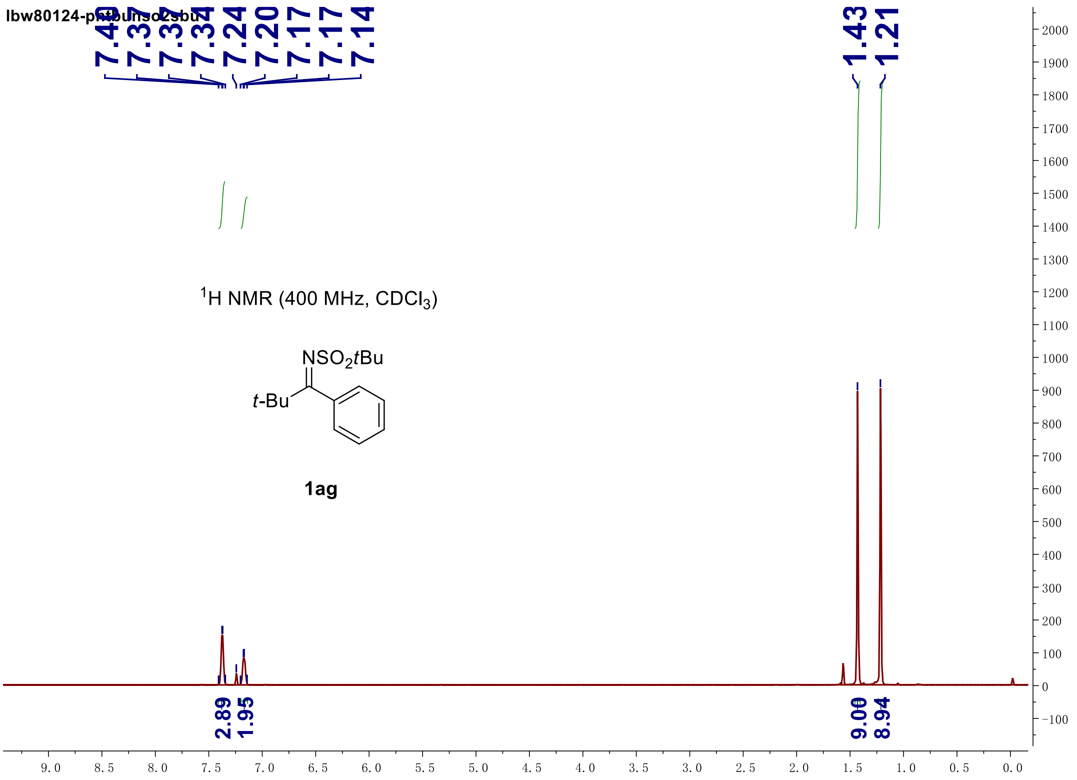

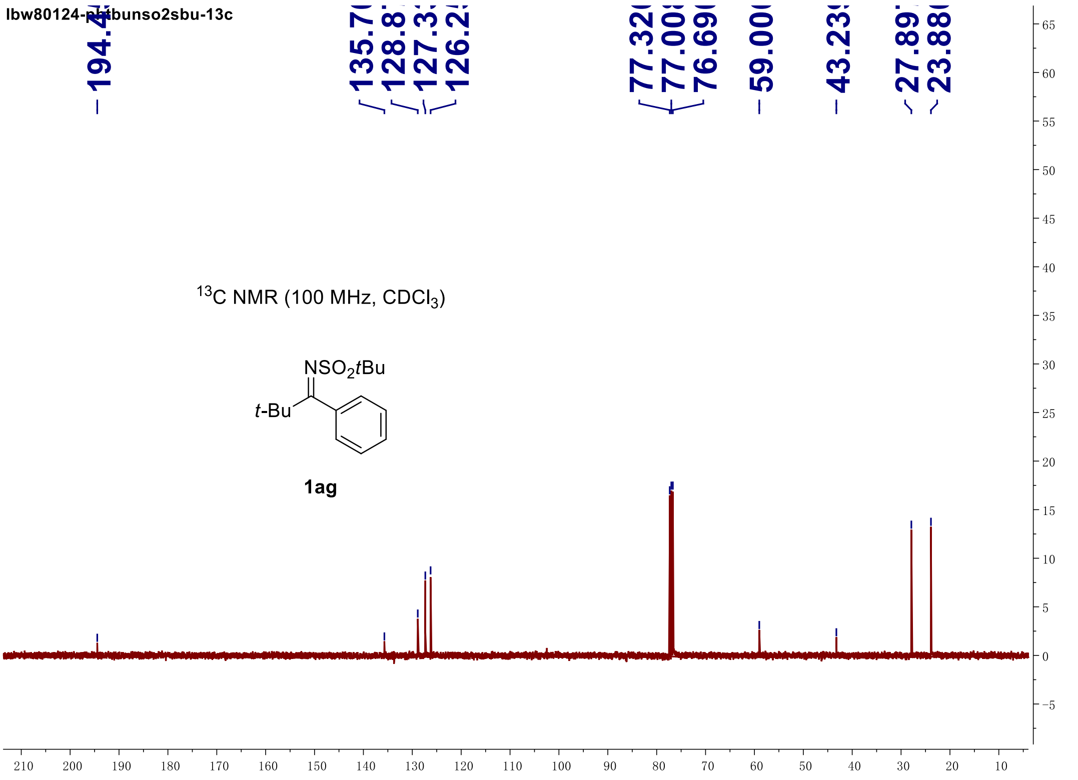

Supplementary Figure 25 ^{1}H and ^{13}C NMR spectrum of compound **1ag** in $CDCl_3$

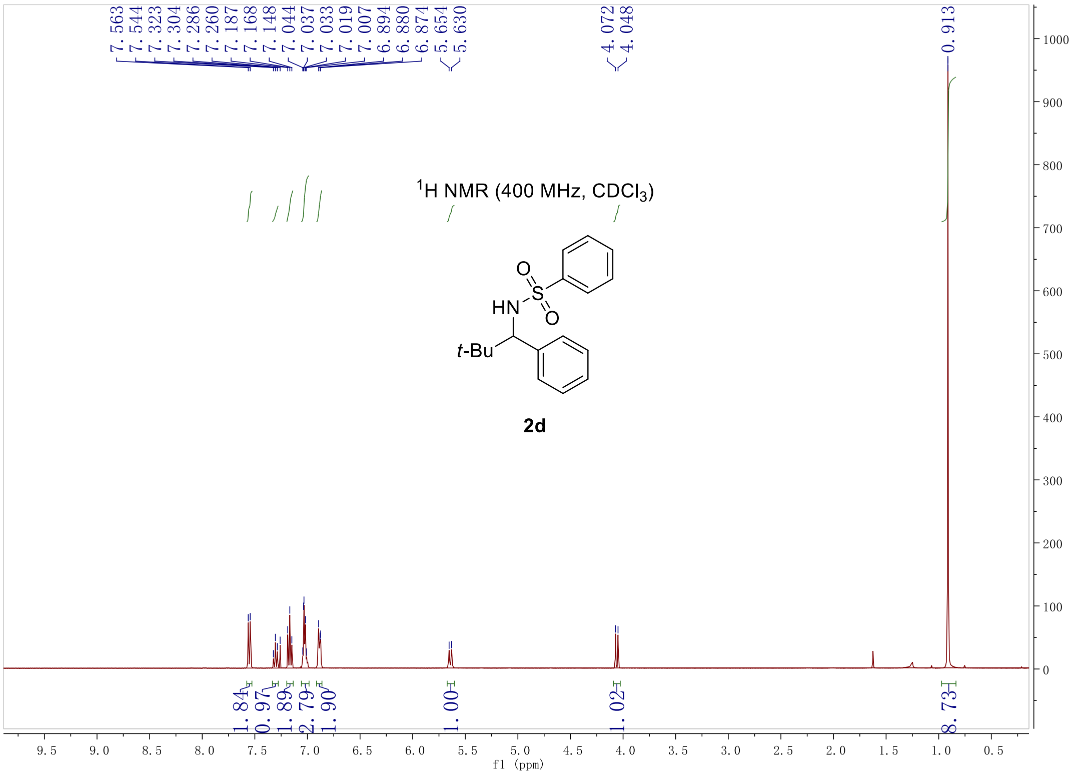

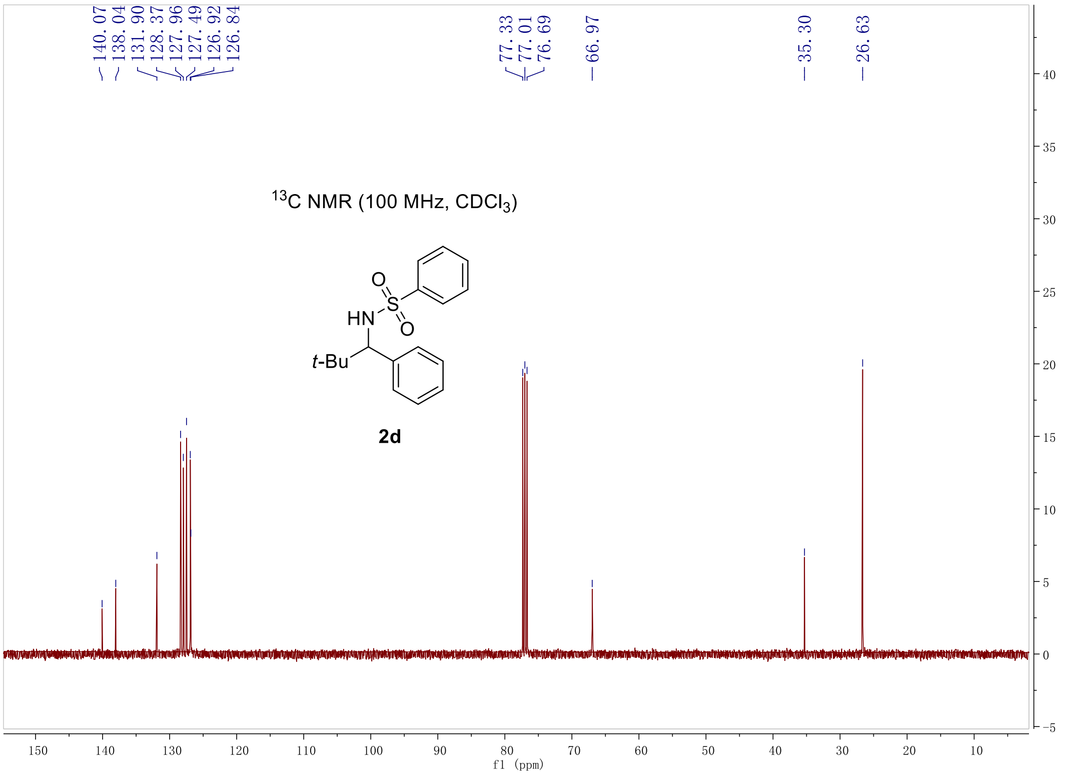

Supplementary Figure 26 ^{1}H and ^{13}C NMR spectrum of compound **2d** in $CDCl_3$

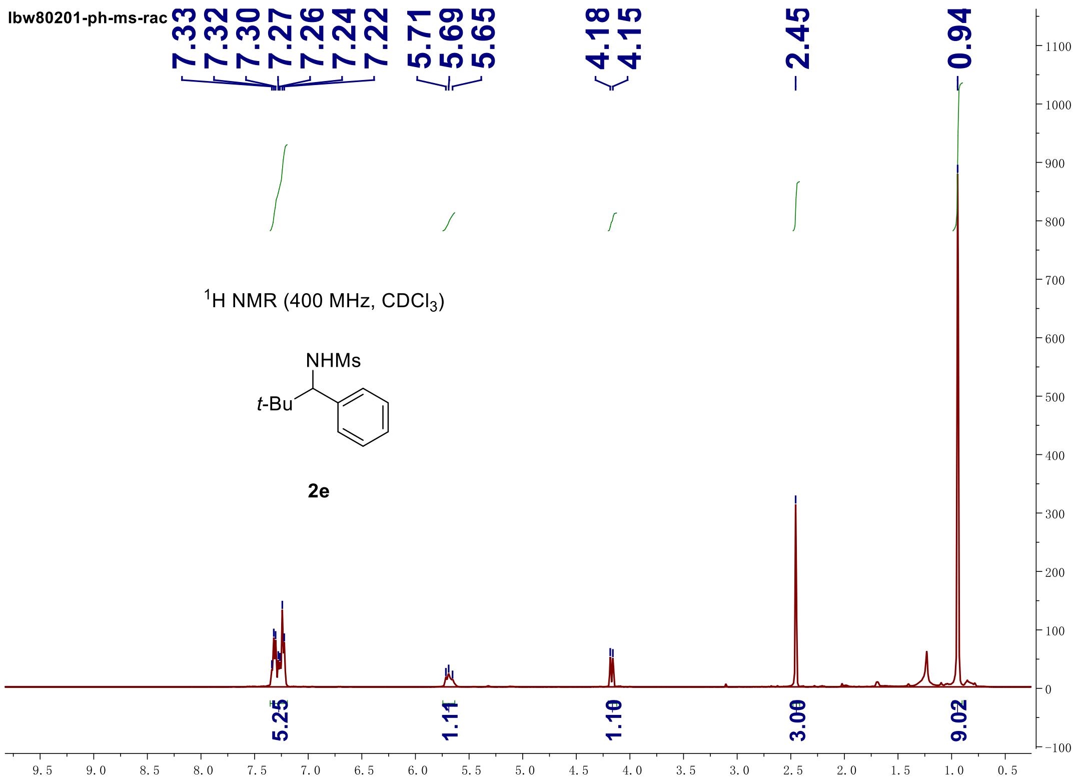

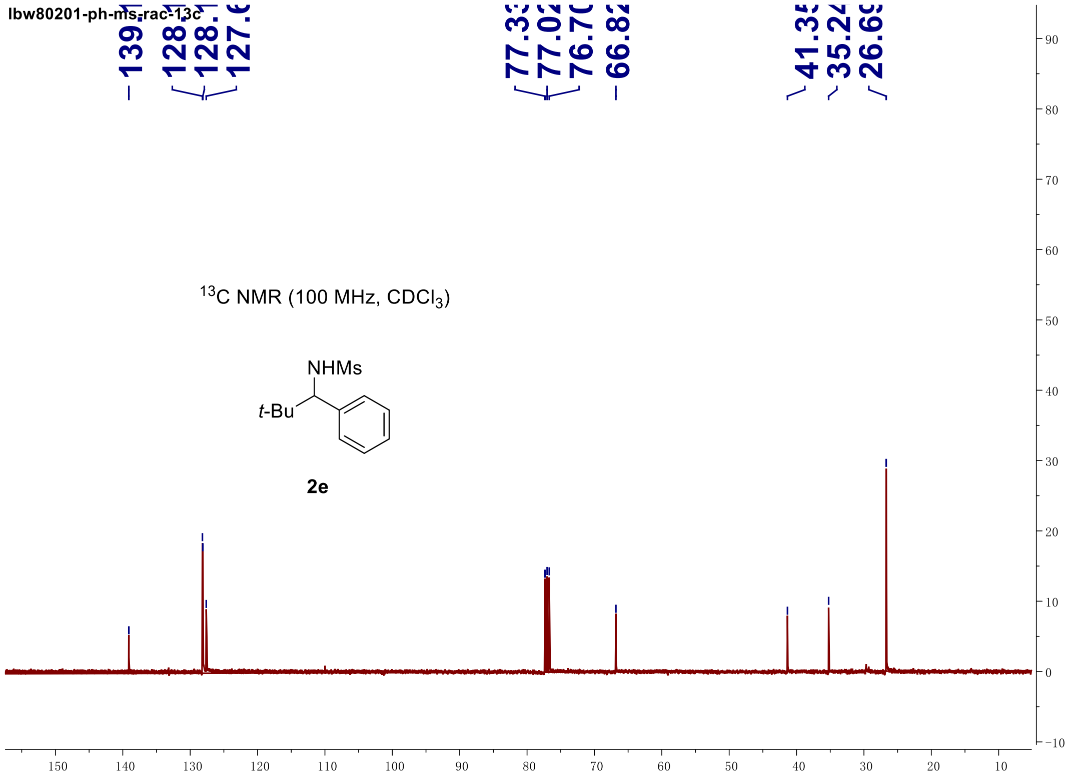

Supplementary Figure 27 ^{1}H and ^{13}C NMR spectrum of compound **2e** in $CDCl_3$

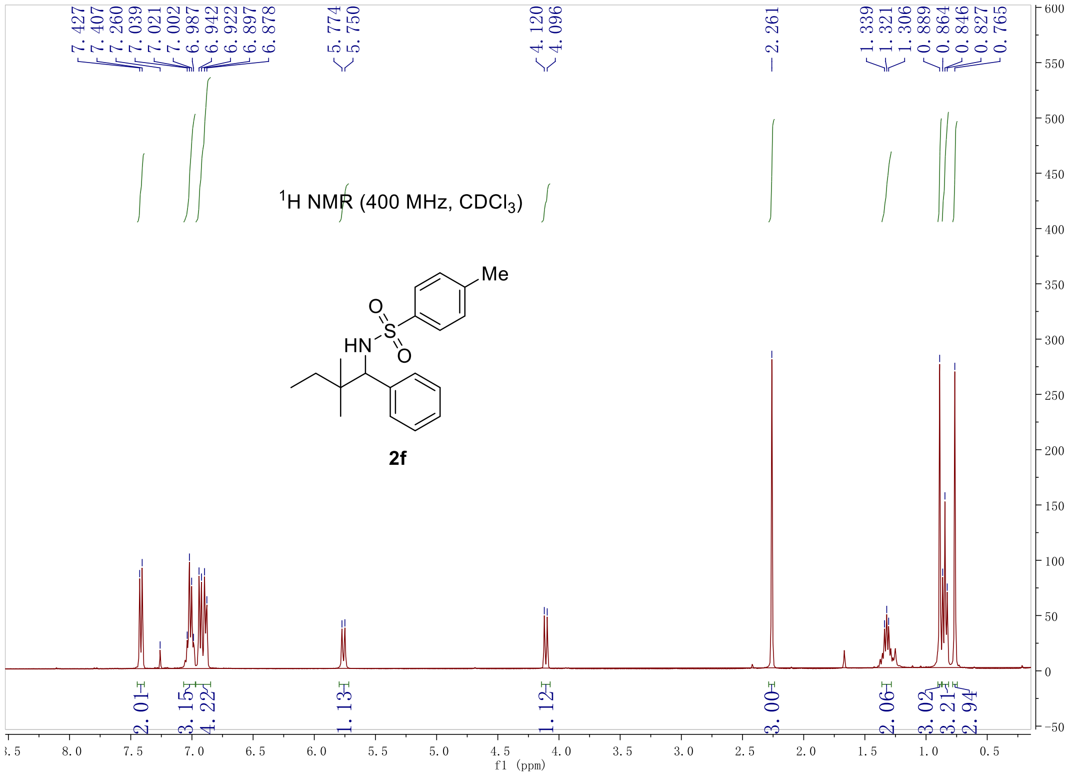

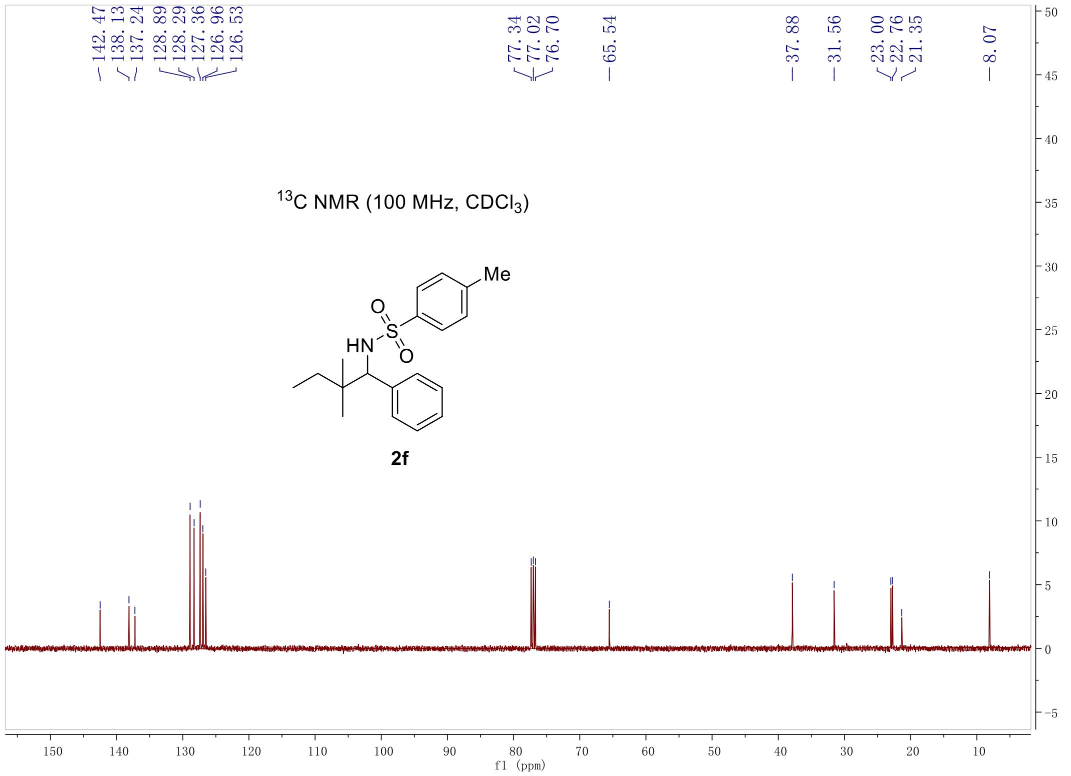

Supplementary Figure 28 ^{1}H and ^{13}C NMR spectrum of compound **2f** in CDCl3

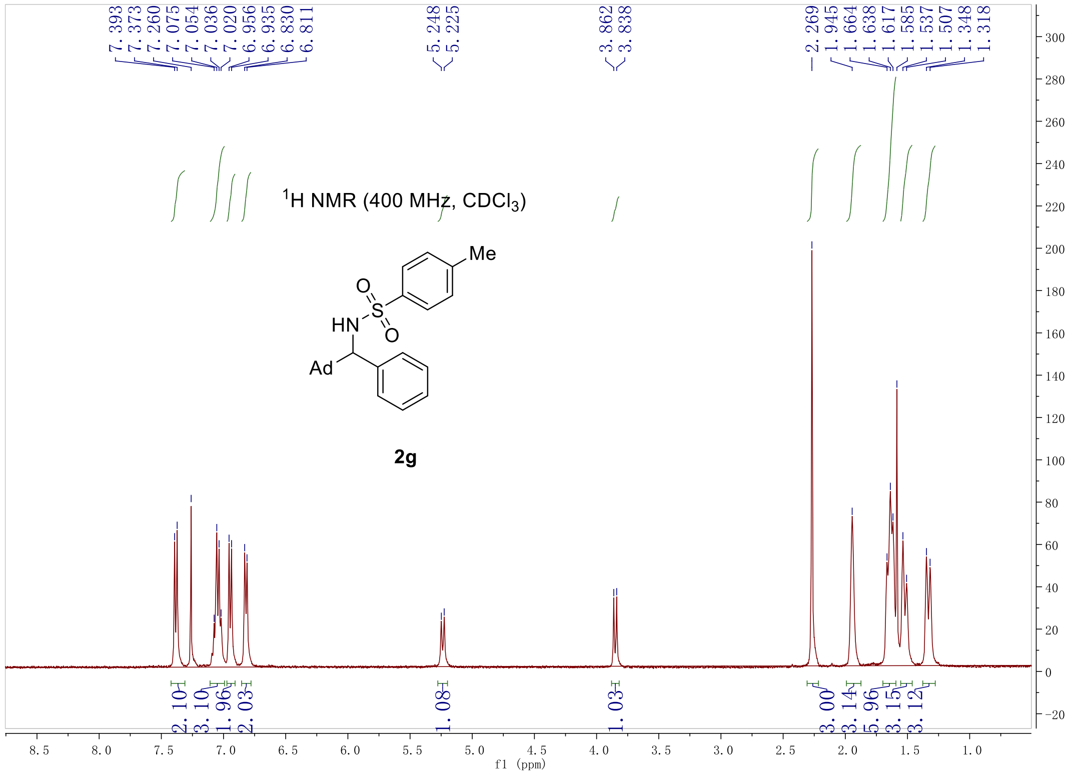

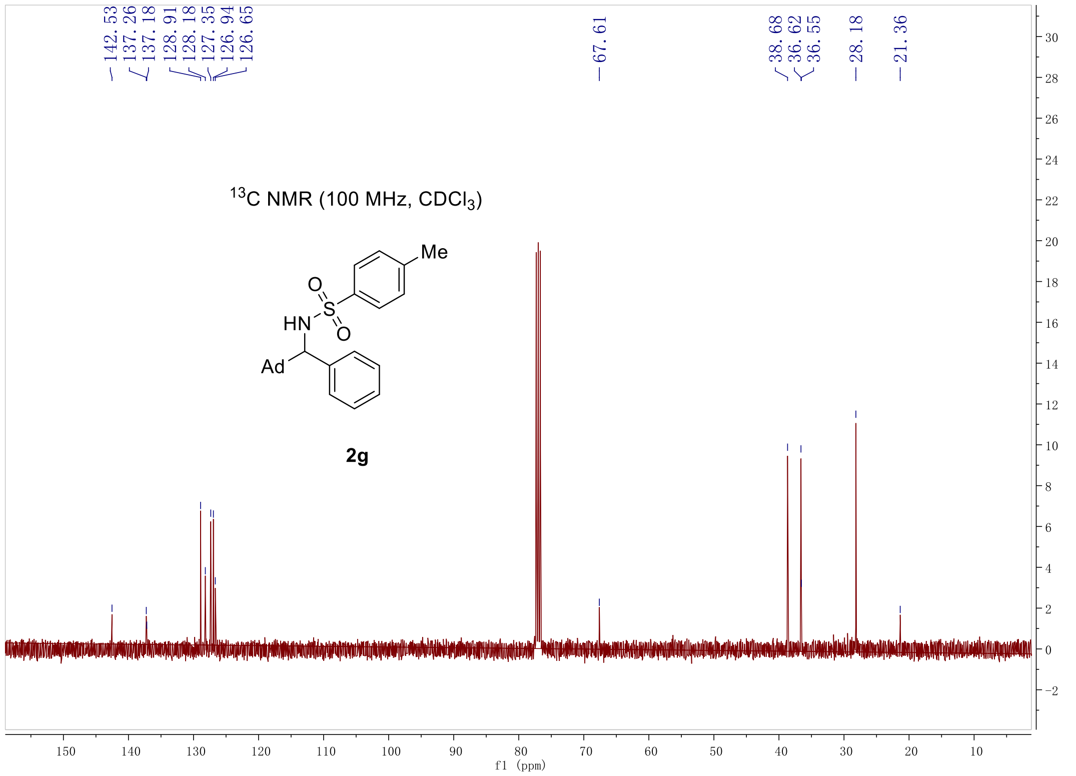

Supplementary Figure 29 ^{1}H and ^{13}C NMR spectrum of compound **2g** in $CDCl_3$

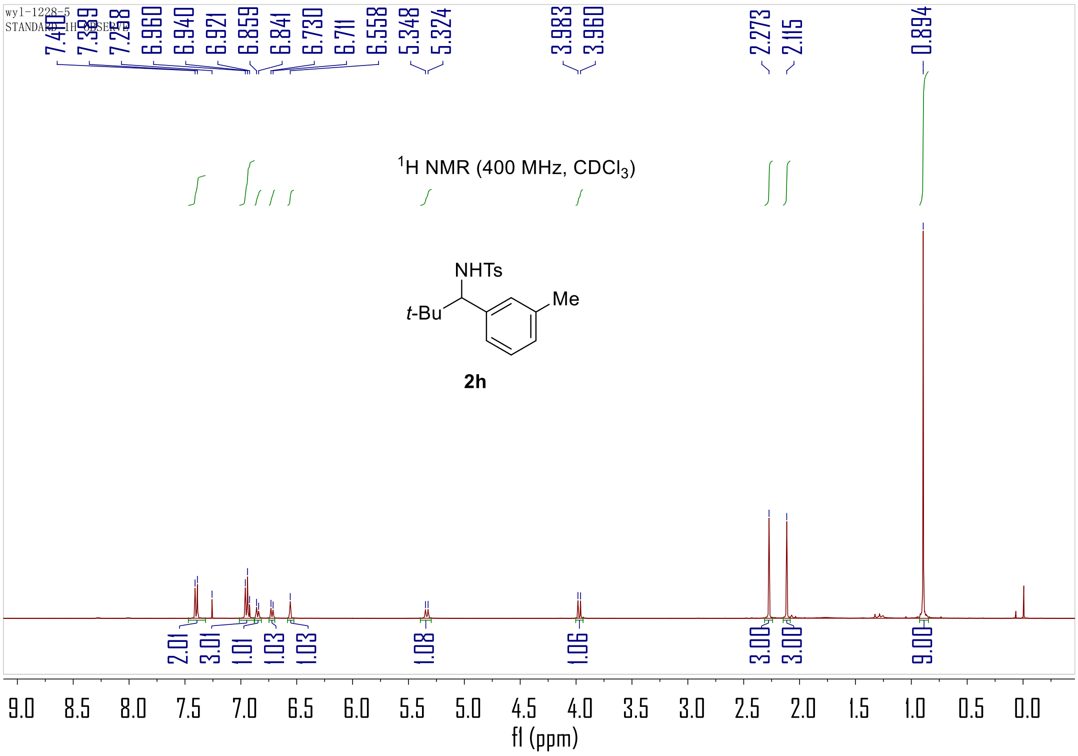

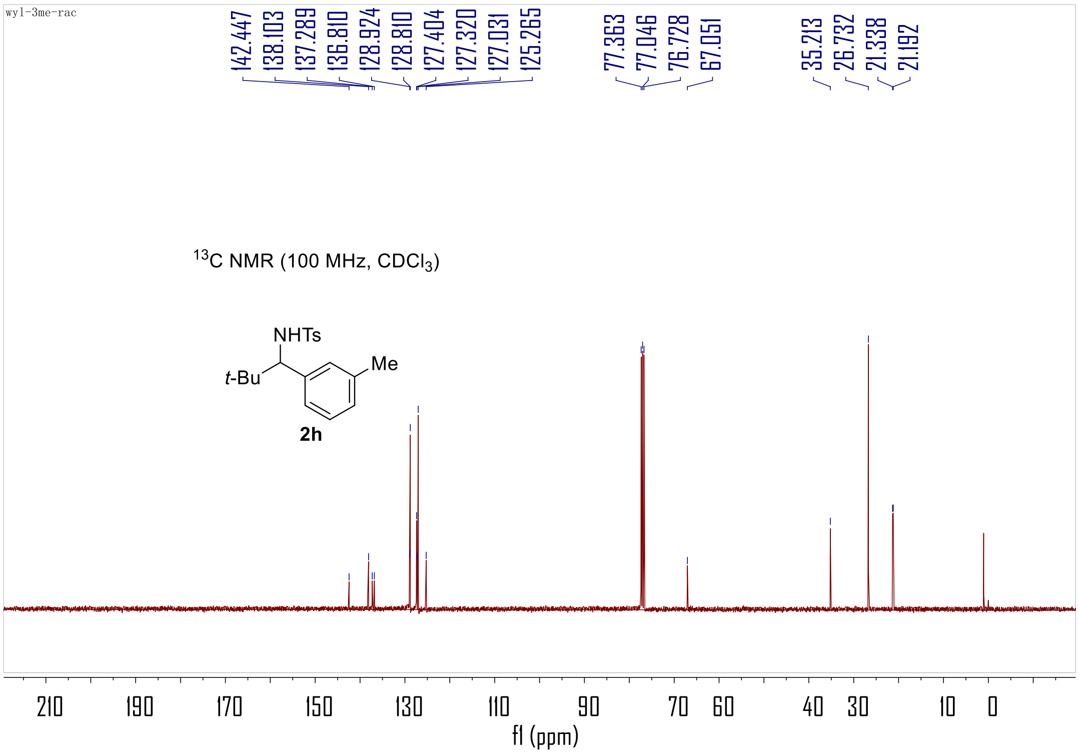

Supplementary Figure 30 ^{1}H and ^{13}C NMR spectrum of compound **2h** in $CDCl_3$

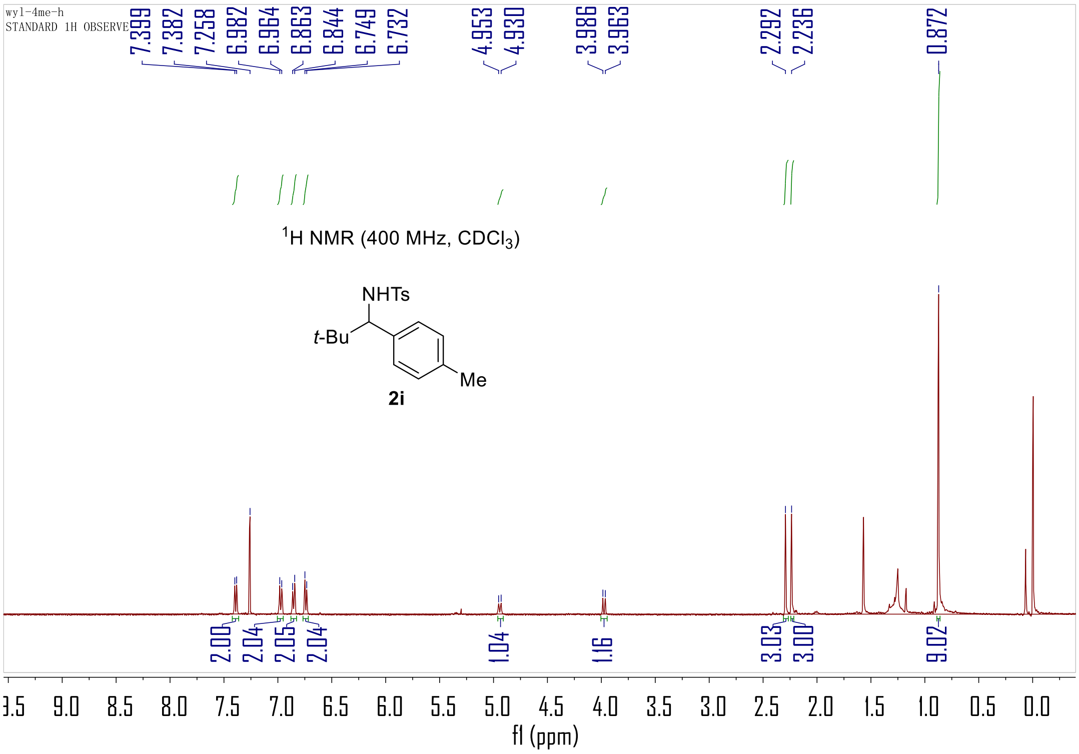

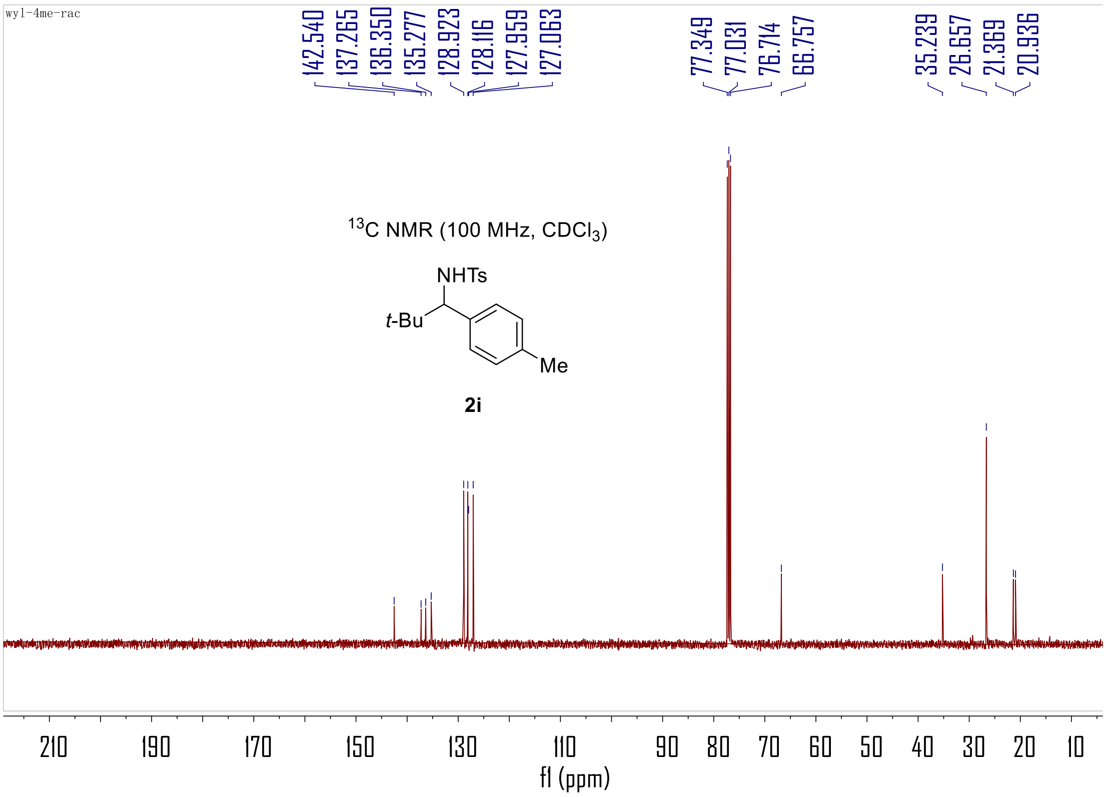

Supplementary Figure 31 ^{1}H and ^{13}C NMR spectrum of compound **2i** in $CDCl_3$

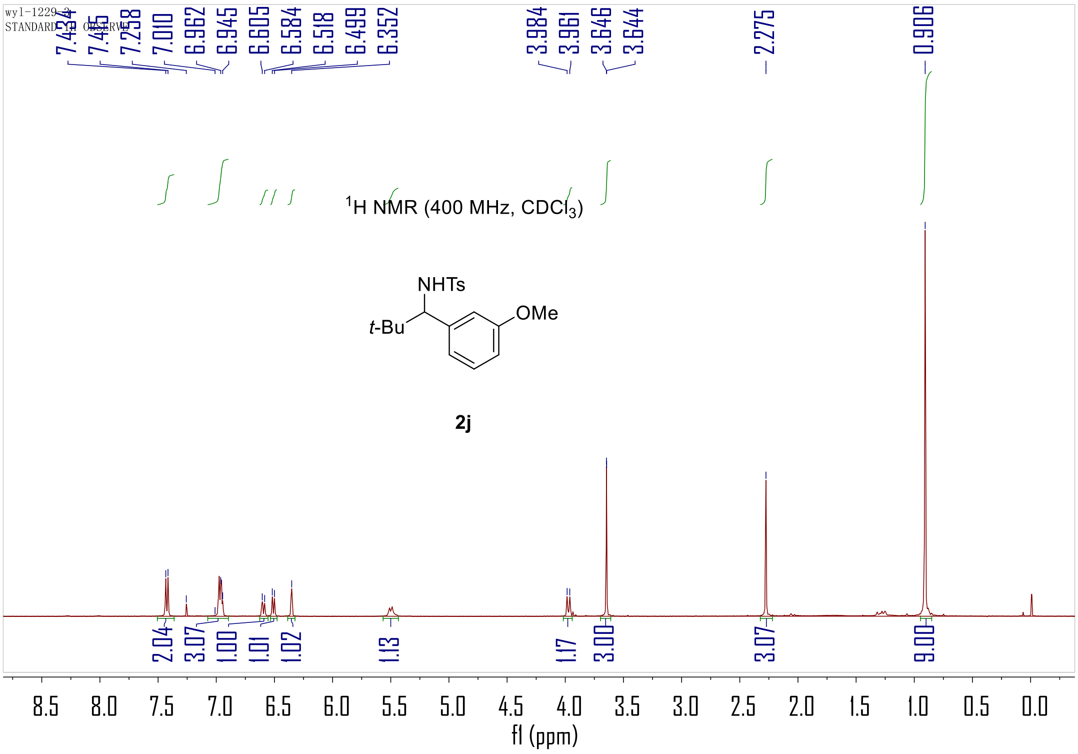

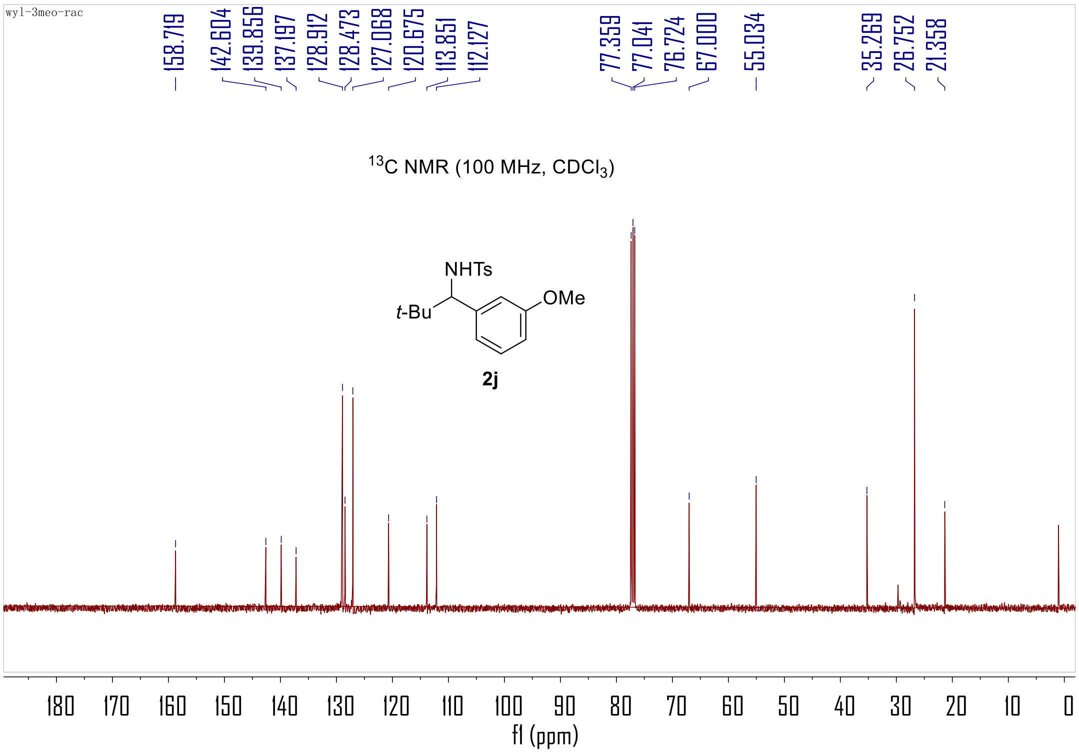

Supplementary Figure 32 ^{1}H and ^{13}C NMR spectrum of compound **2j** in $CDCl_3$

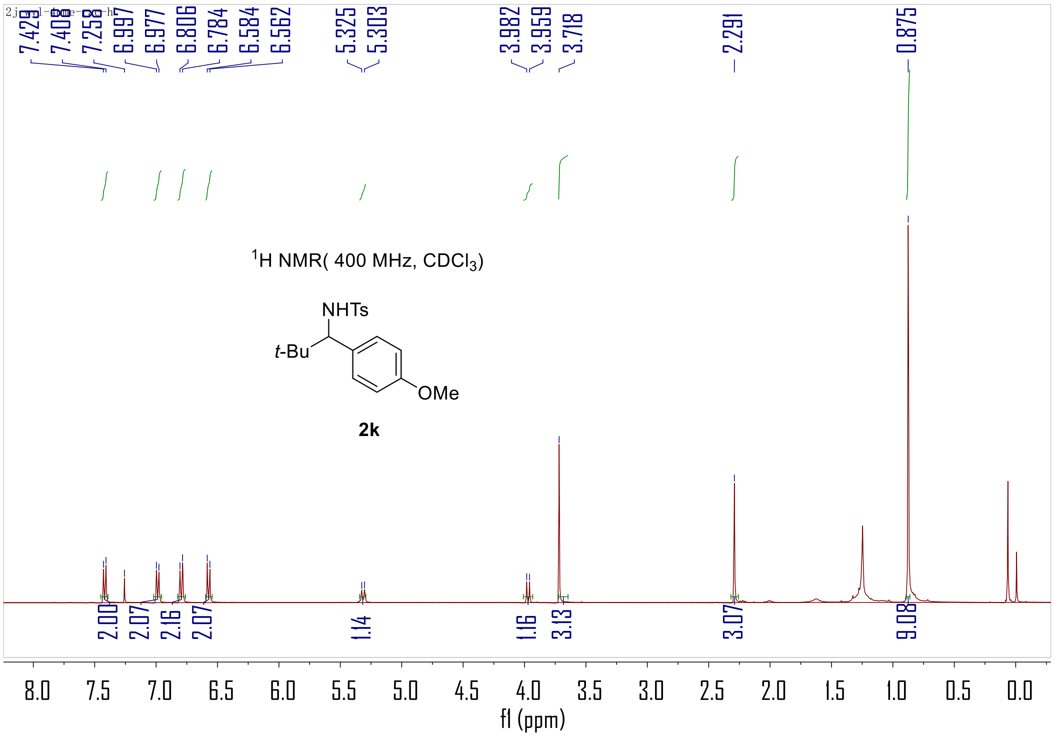

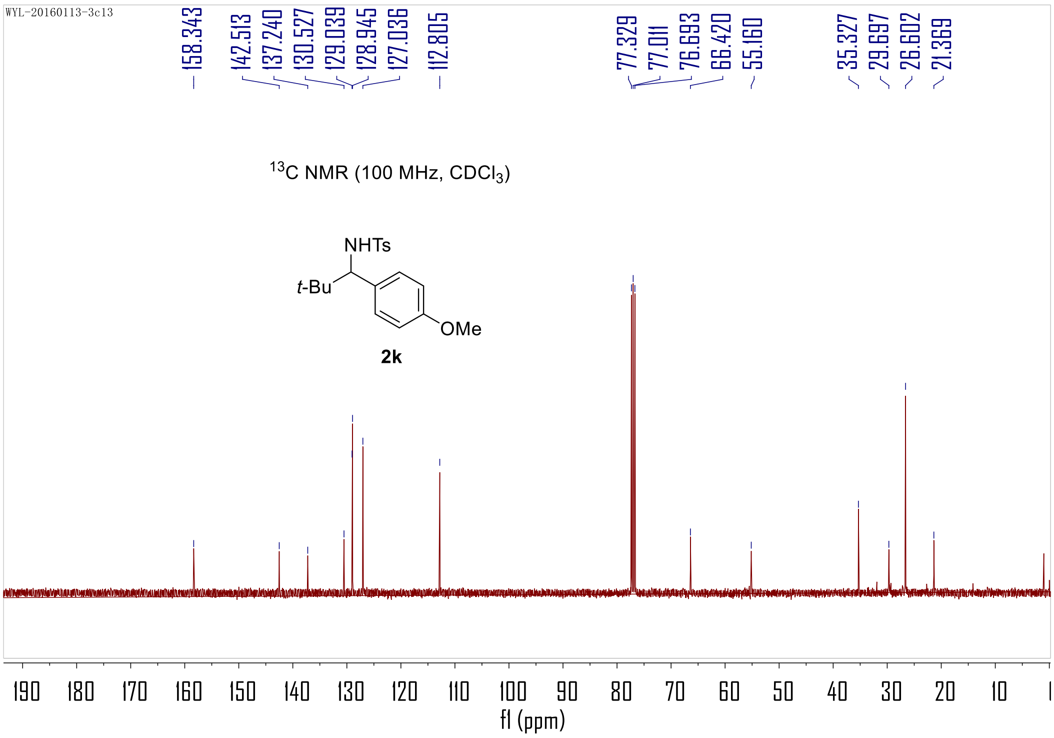

Supplementary Figure 33 ^{1}H and ^{13}C NMR spectrum of compound **2k** in $CDCl_3$

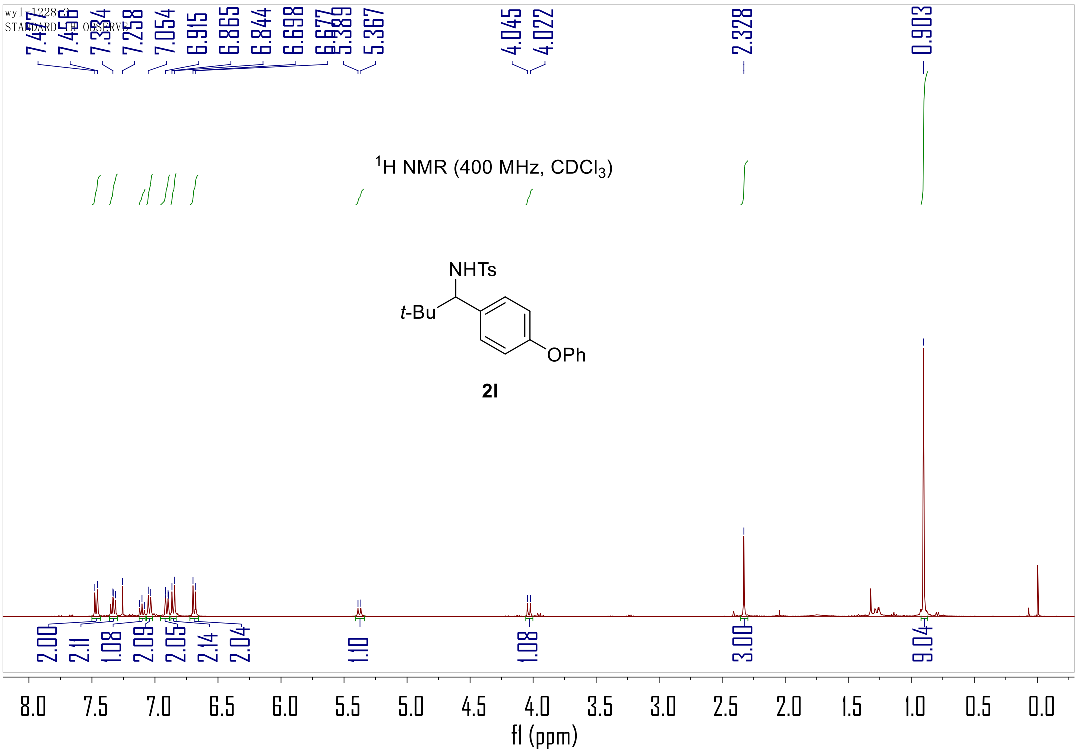

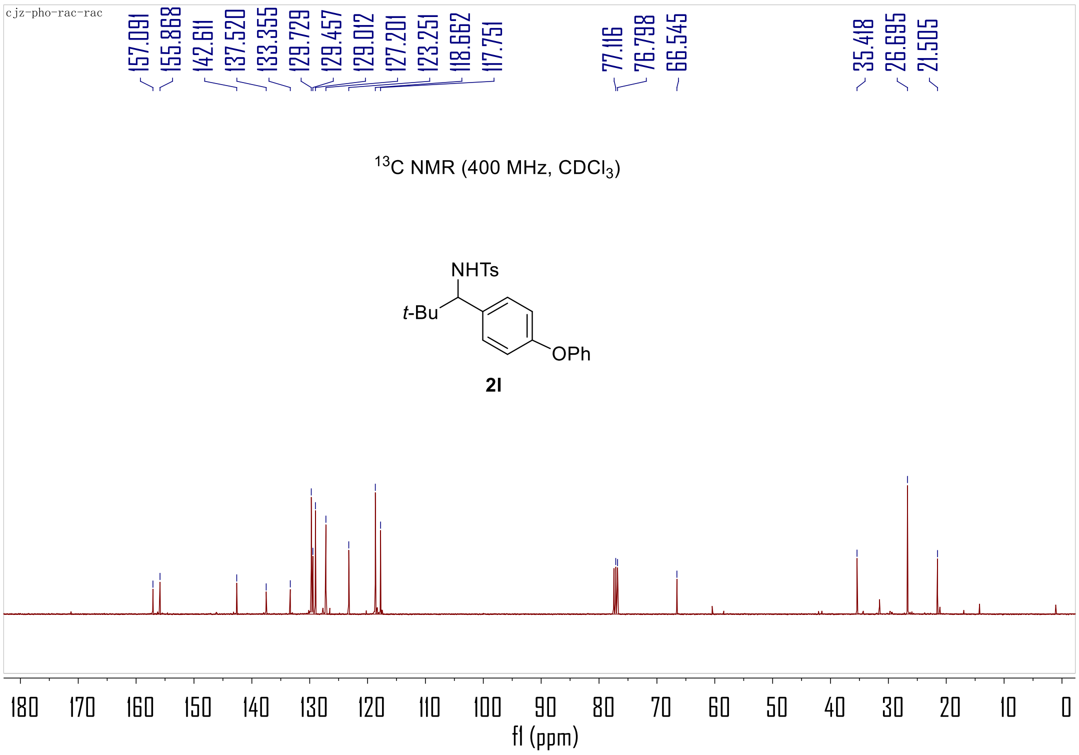

Supplementary Figure 34 ^{1}H and ^{13}C NMR spectrum of compound **2l** in $CDCl_3$

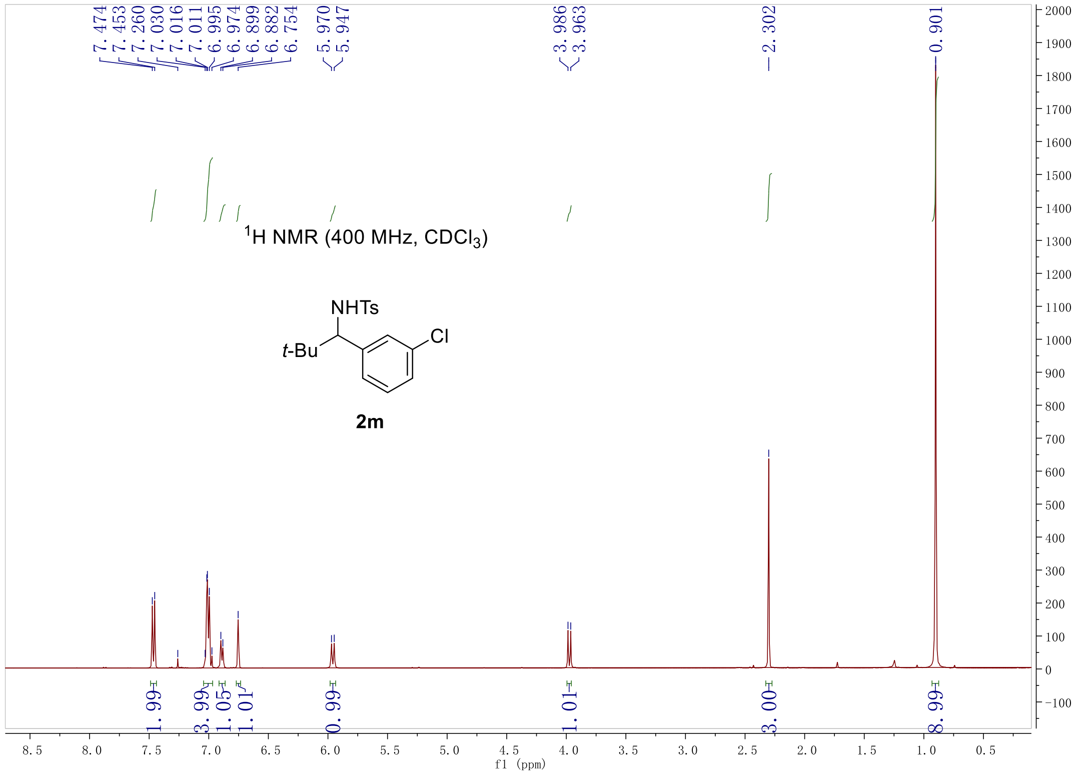

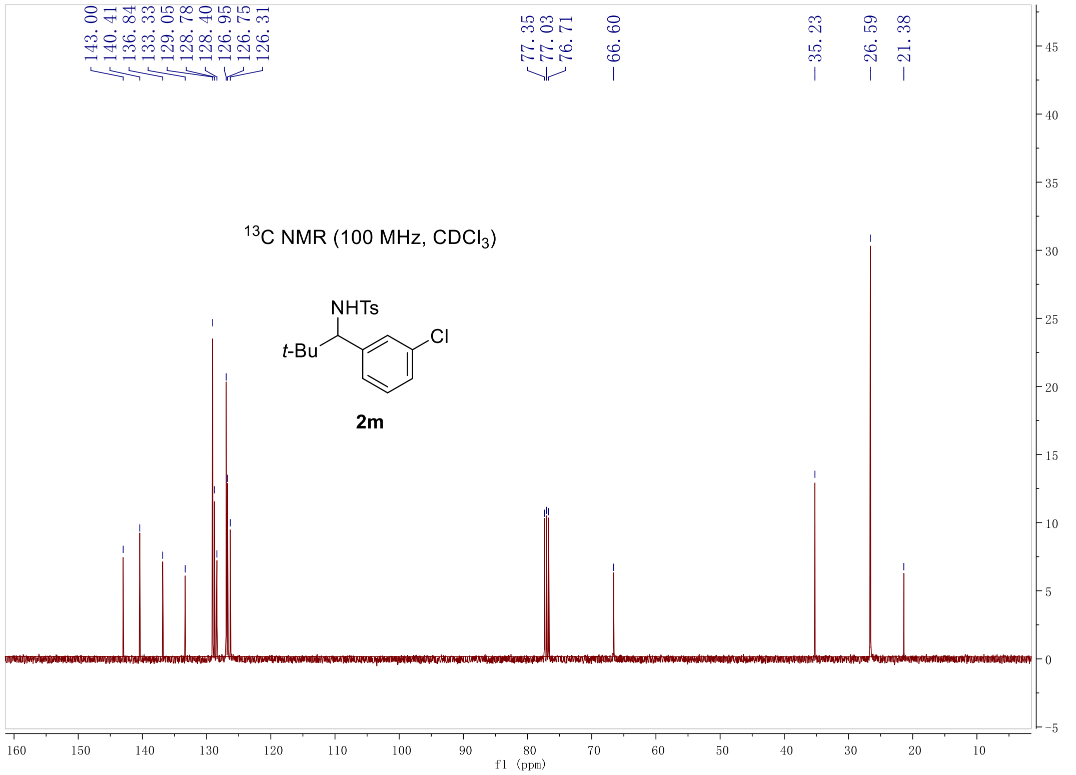

Supplementary Figure 35 ^{1}H and ^{13}C NMR spectrum of compound **2m** in $CDCl_3$

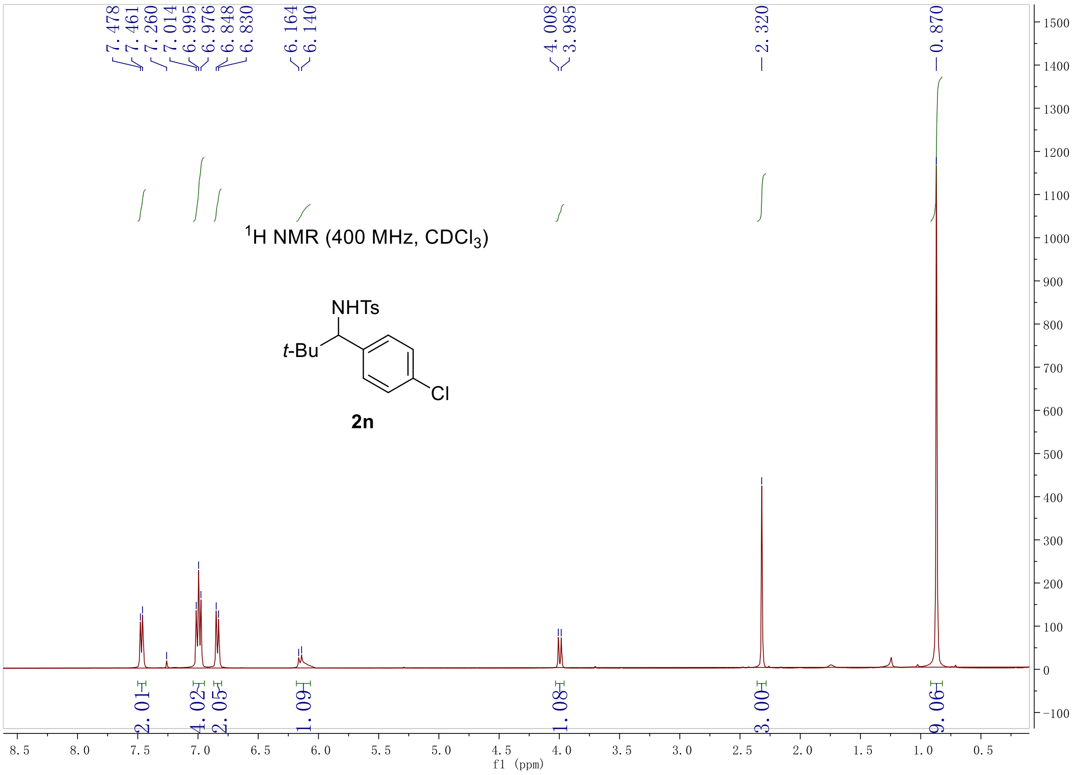

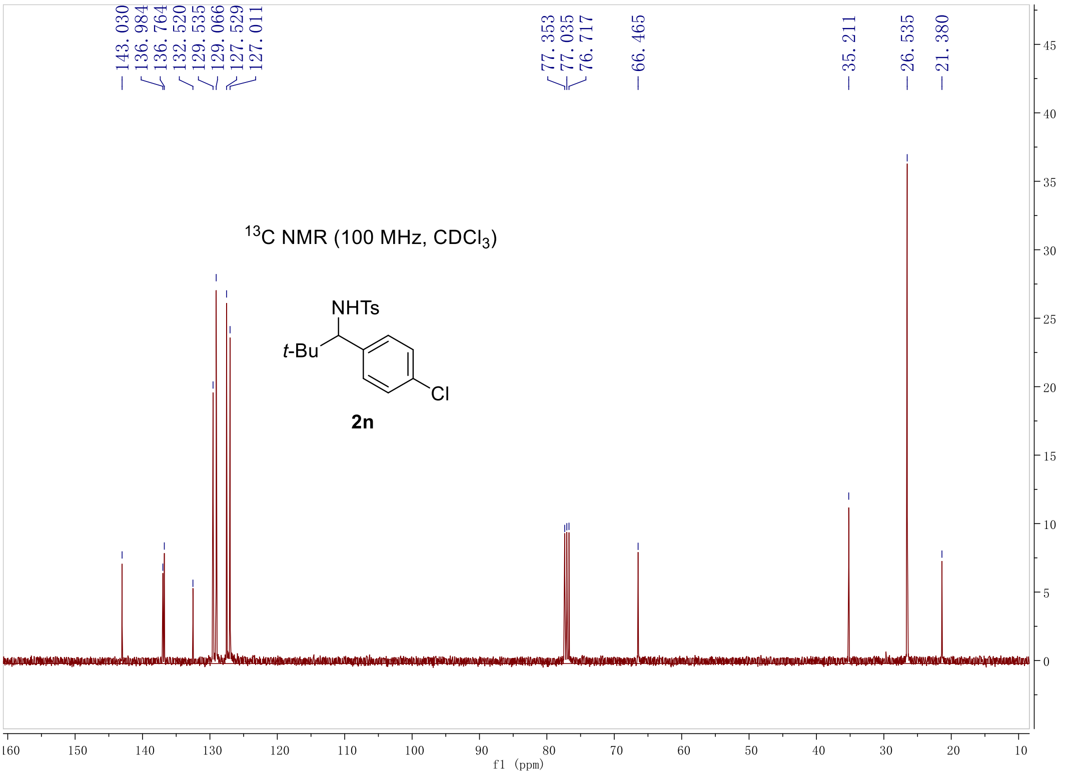

Supplementary Figure 36 ^{1}H and ^{13}C NMR spectrum of compound **2n** in CDCl3

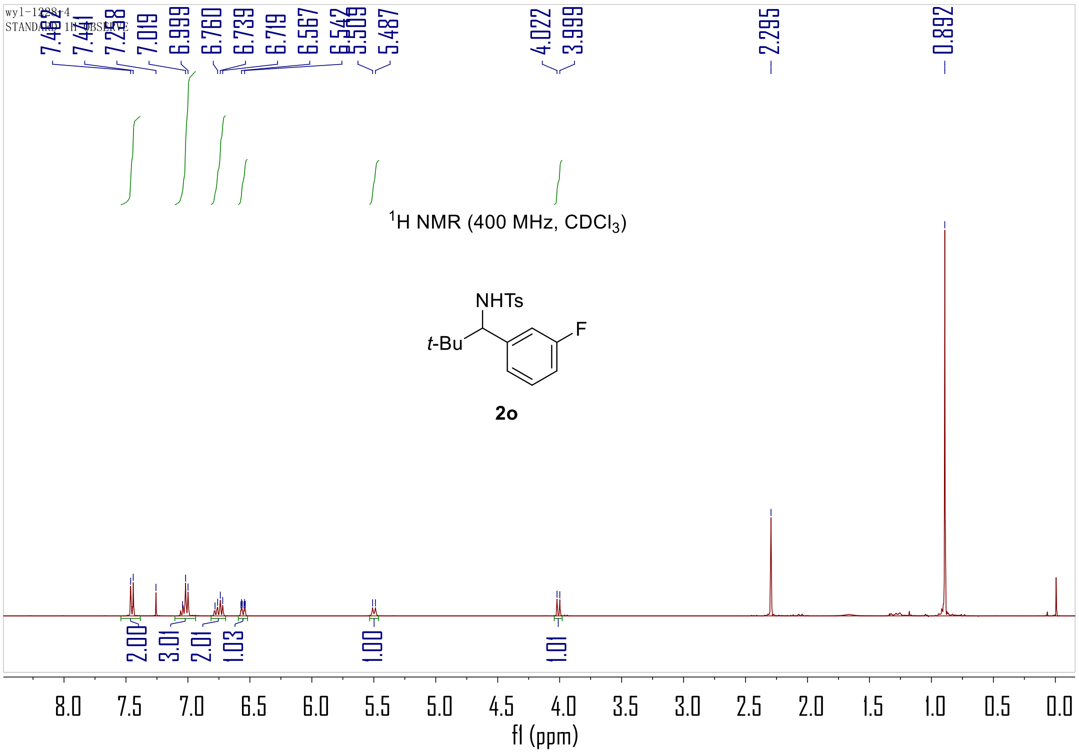

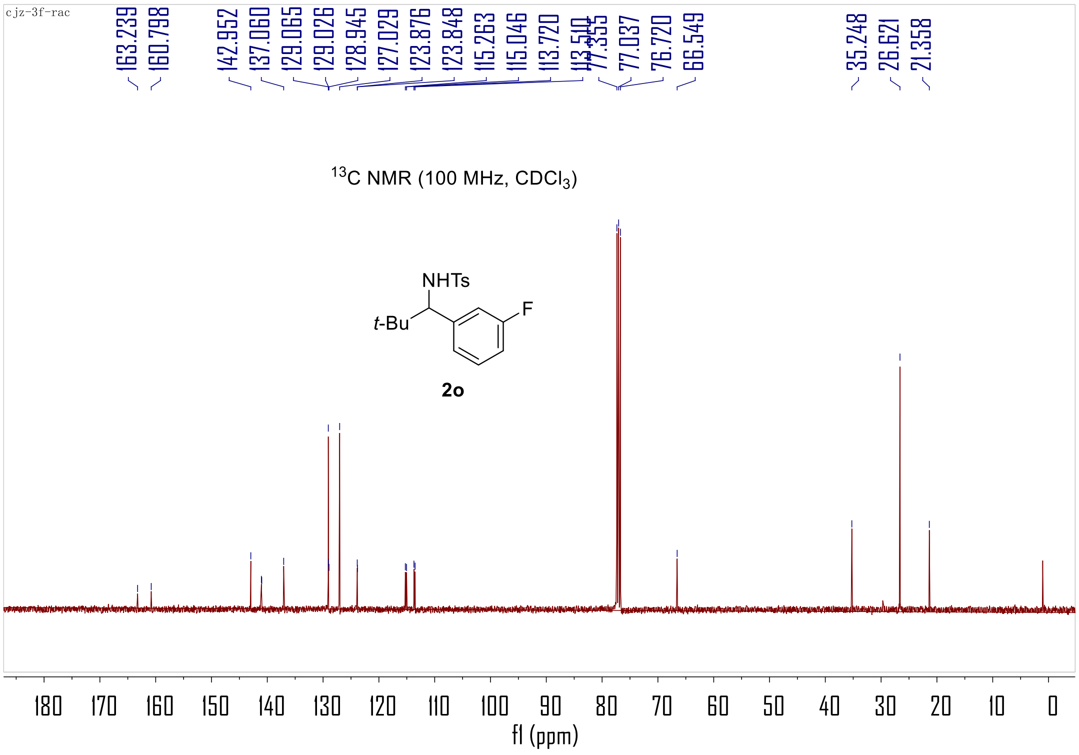

Supplementary Figure 37 ^{1}H and ^{13}C NMR spectrum of compound **2o** in CDCl3

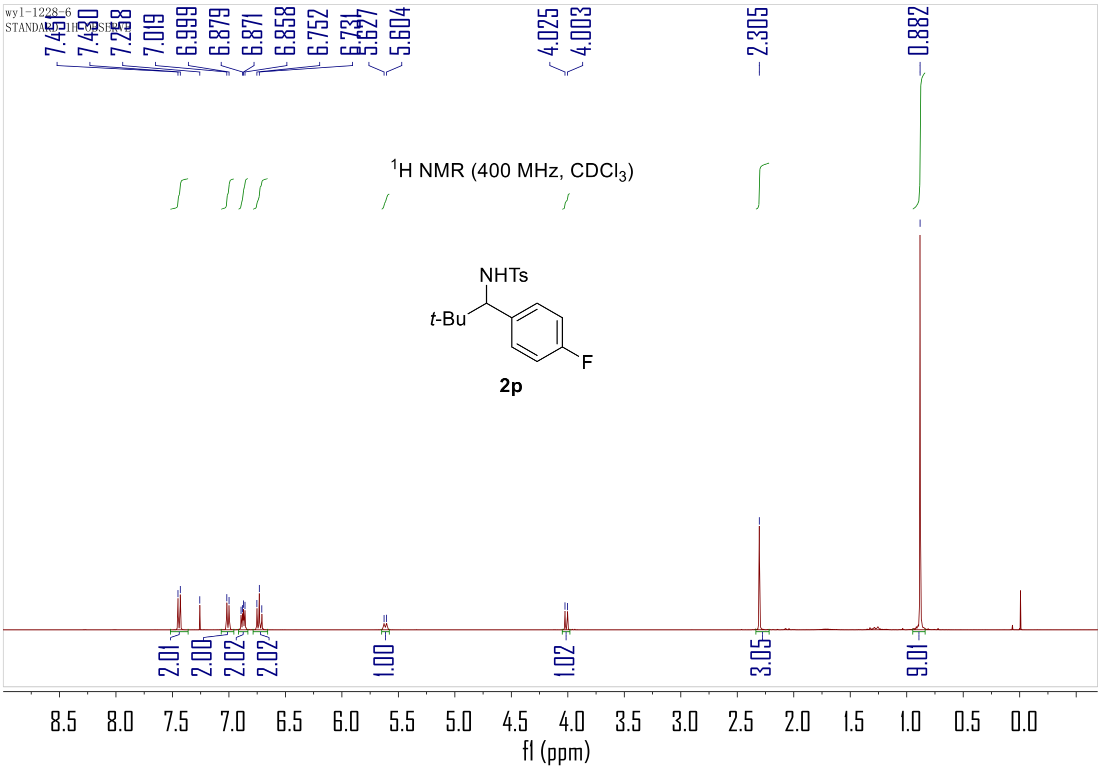

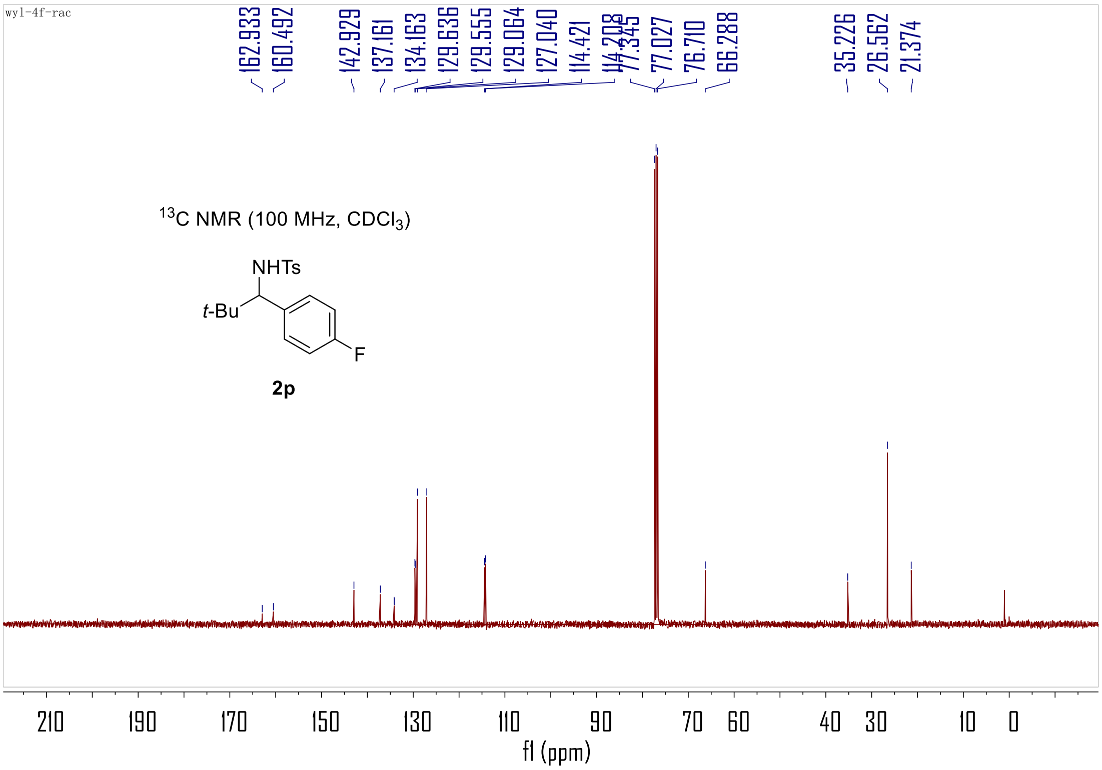

Supplementary Figure 38 ^{1}H and ^{13}C NMR spectrum of compound **2p** in CDCl3

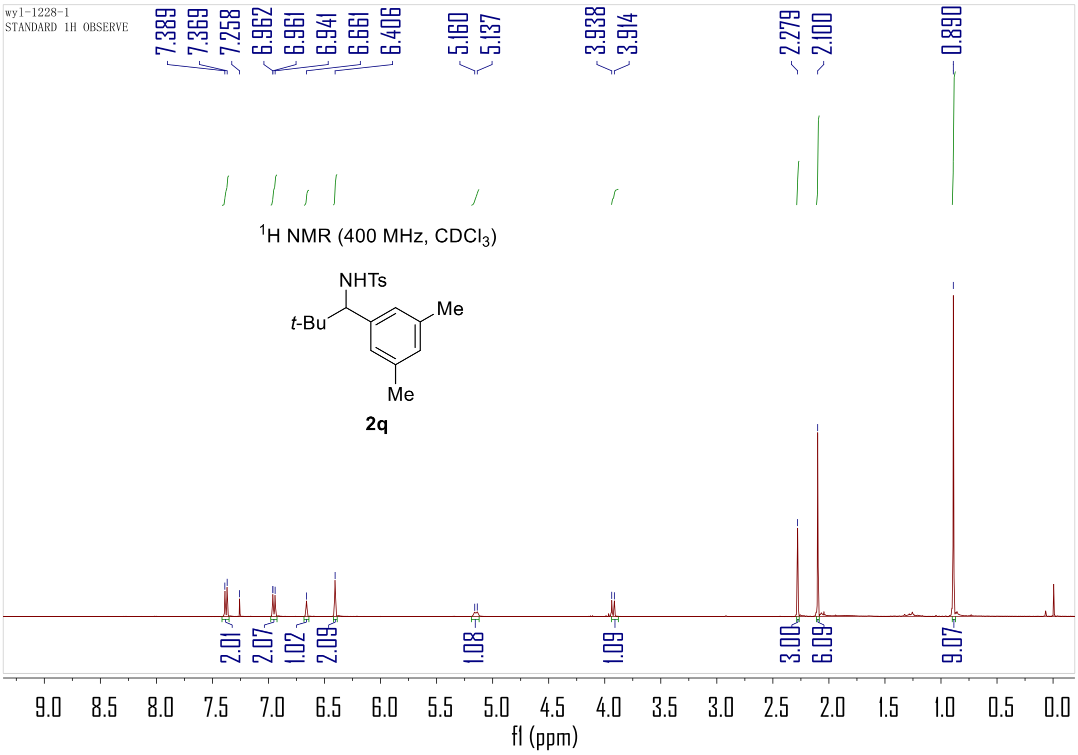

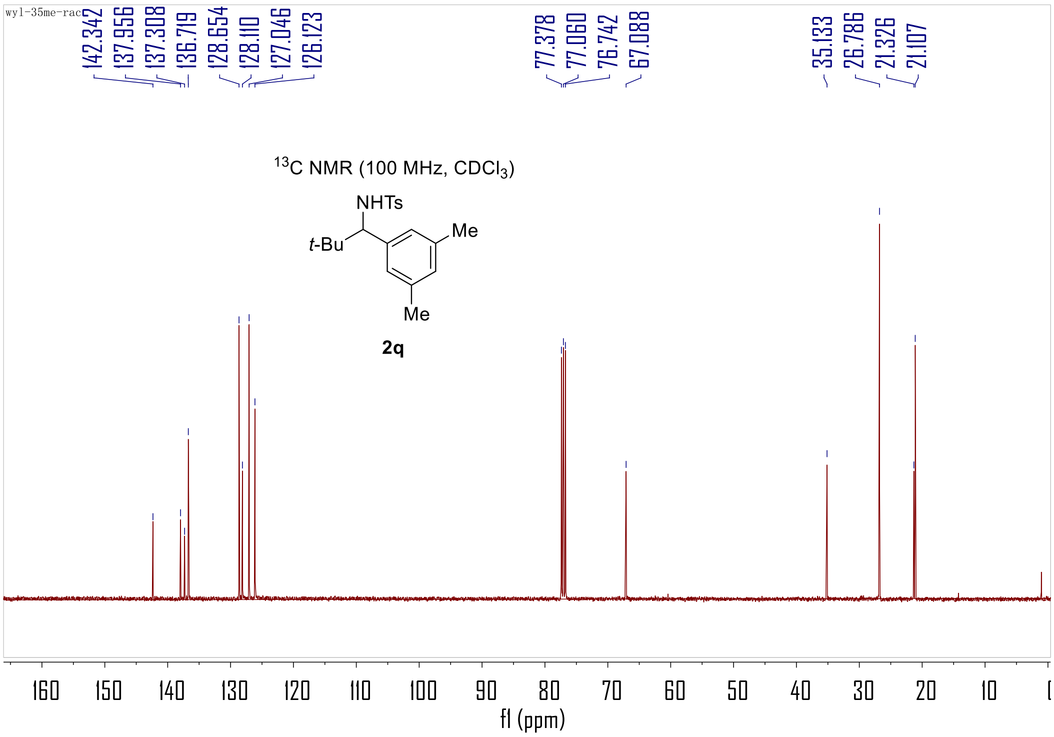

Supplementary Figure 39 ^{1}H and ^{13}C NMR spectrum of compound **2q** in $CDCl_3$

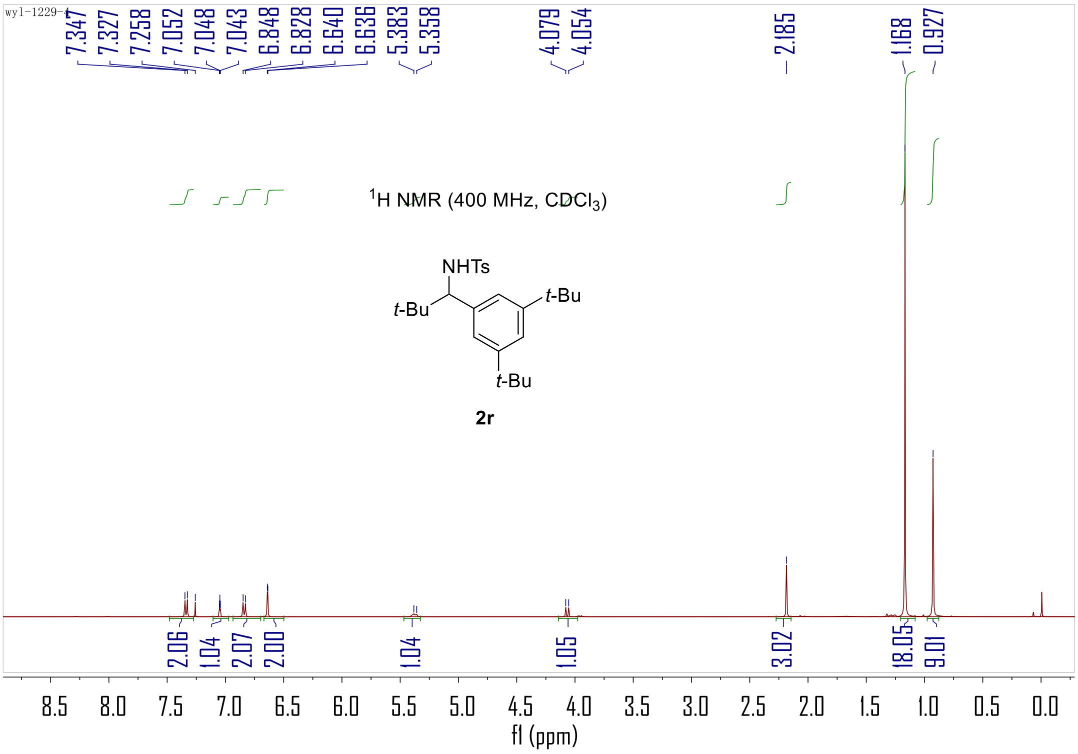

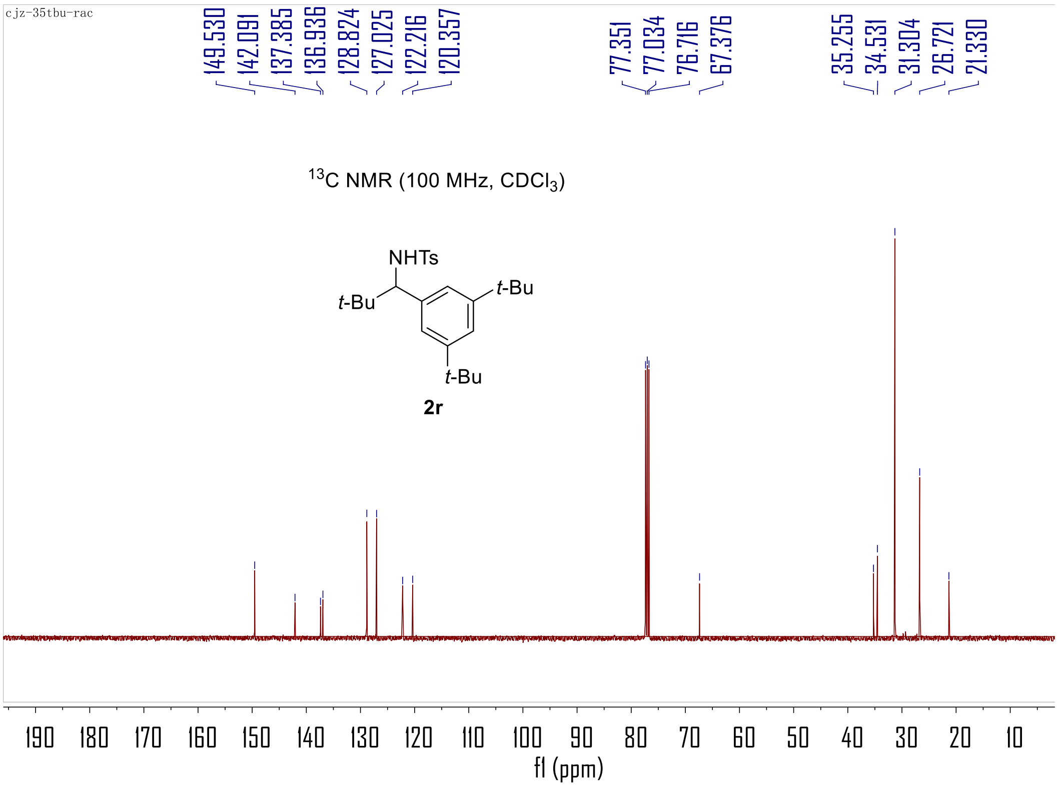

Supplementary Figure 40 ^{1}H and ^{13}C NMR spectrum of compound **2r** in CDCl3

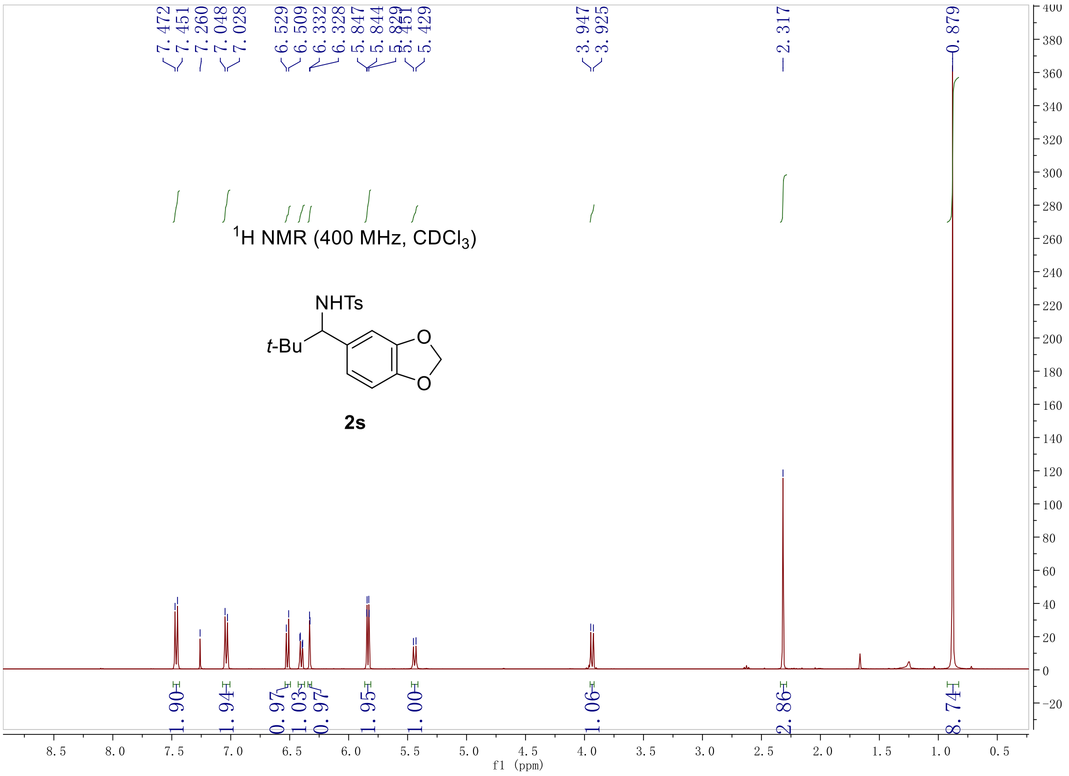

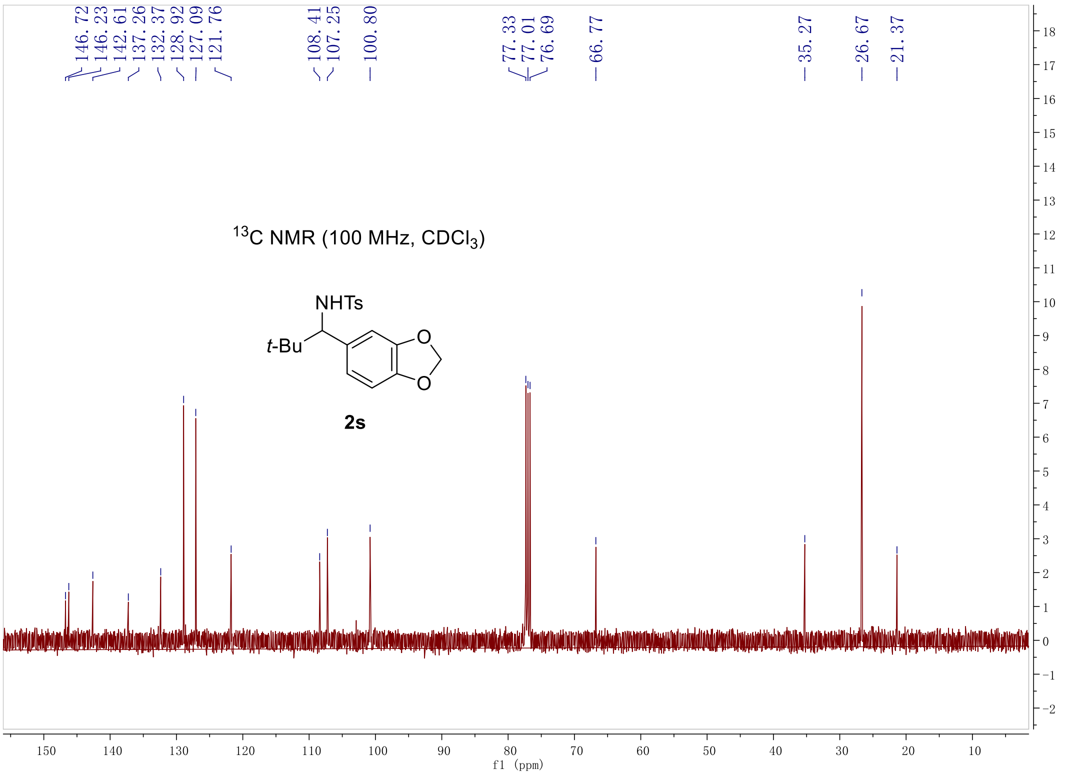

Supplementary Figure 41 ^{1}H and ^{13}C NMR spectrum of compound **2s** in CDCl$_3$

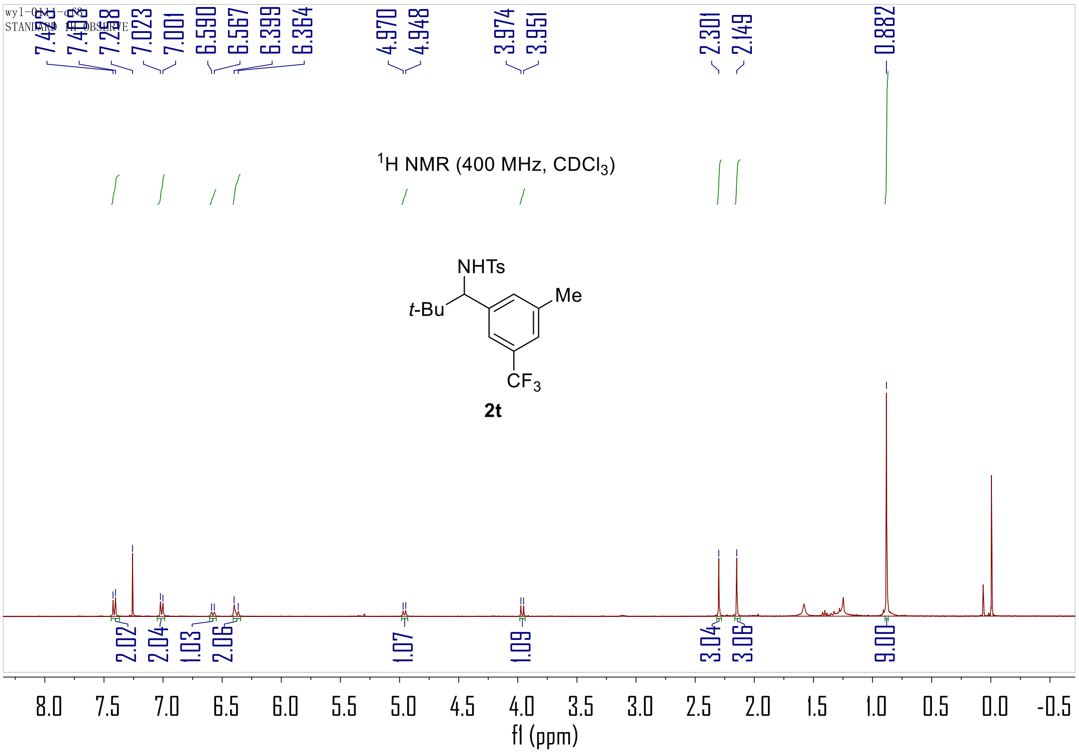

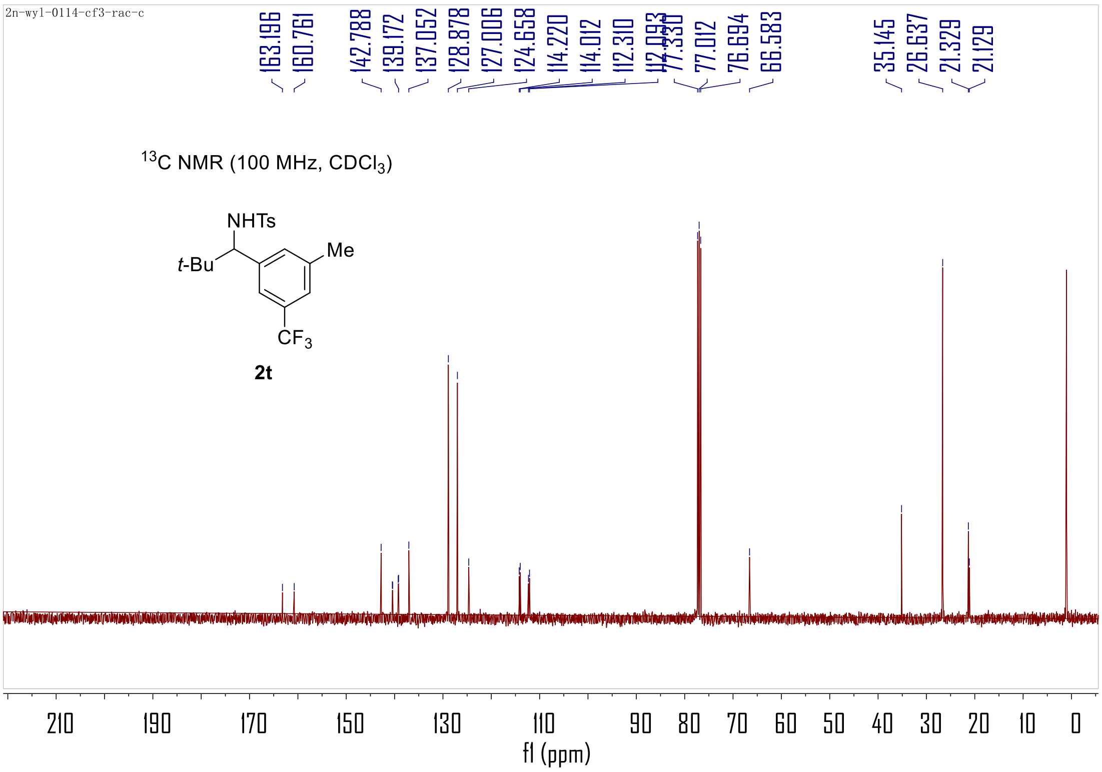

Supplementary Figure 42 ^{1}H and ^{13}C NMR spectrum of compound **2t** in $CDCl_3$

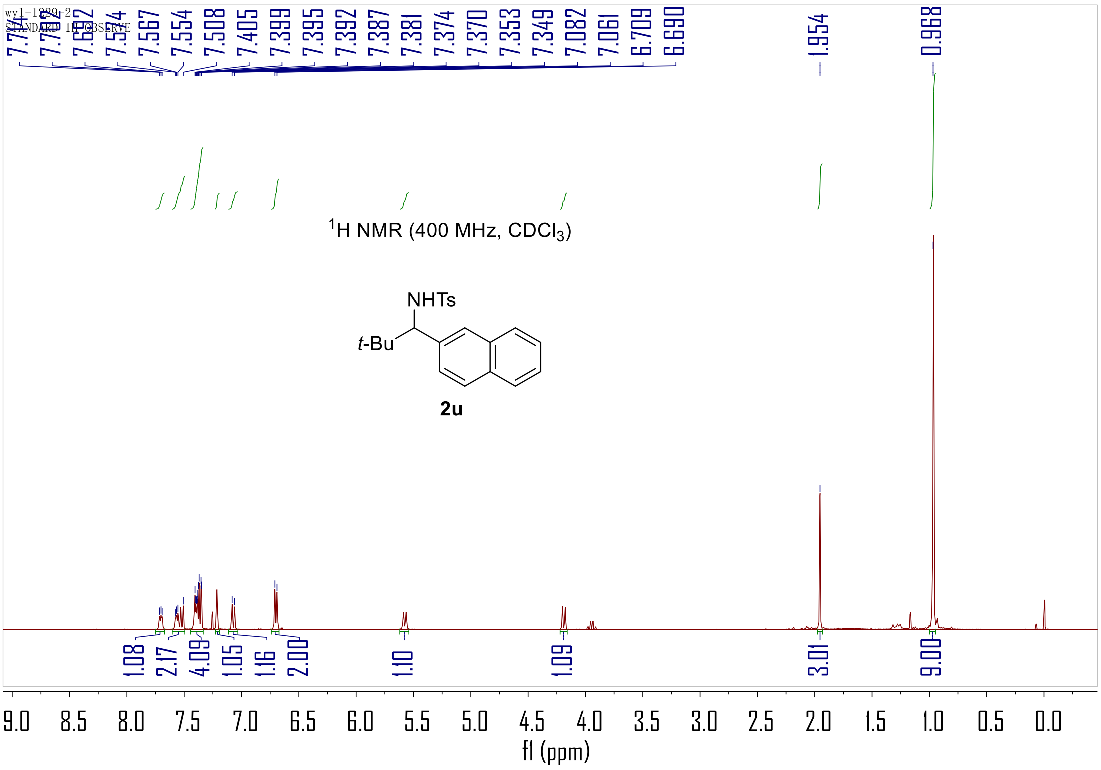

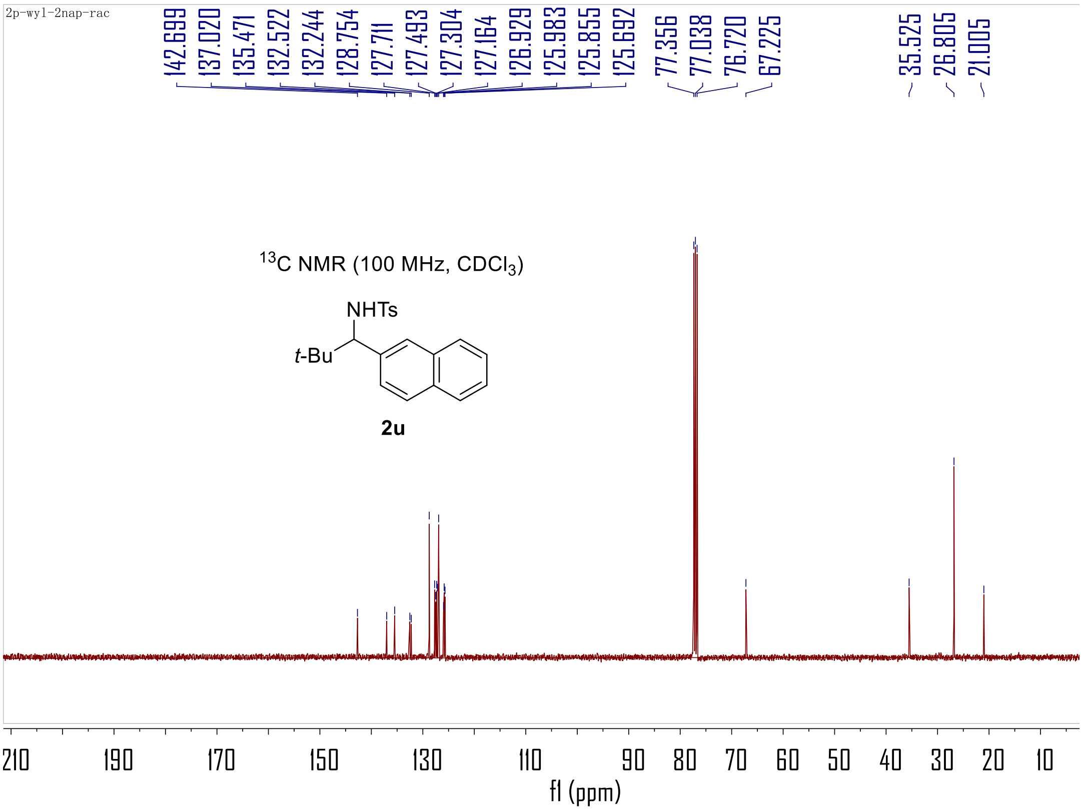

Supplementary Figure 43 ^{1}H and ^{13}C NMR spectrum of compound **2u** in CDCl3

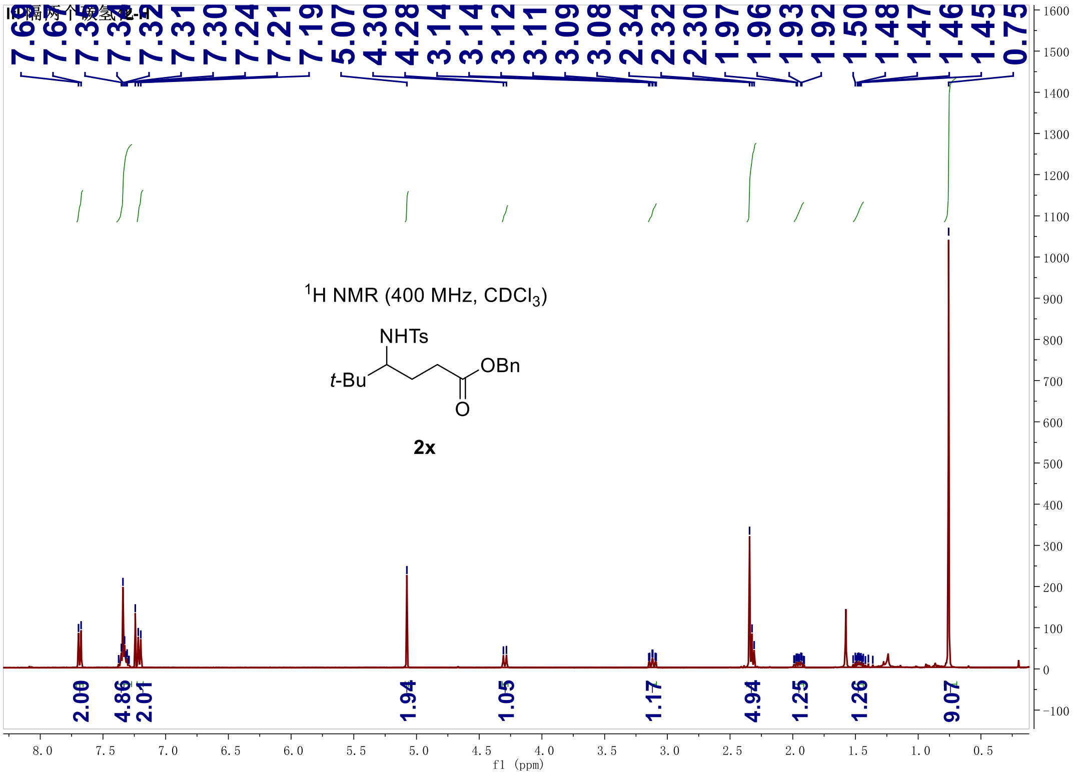

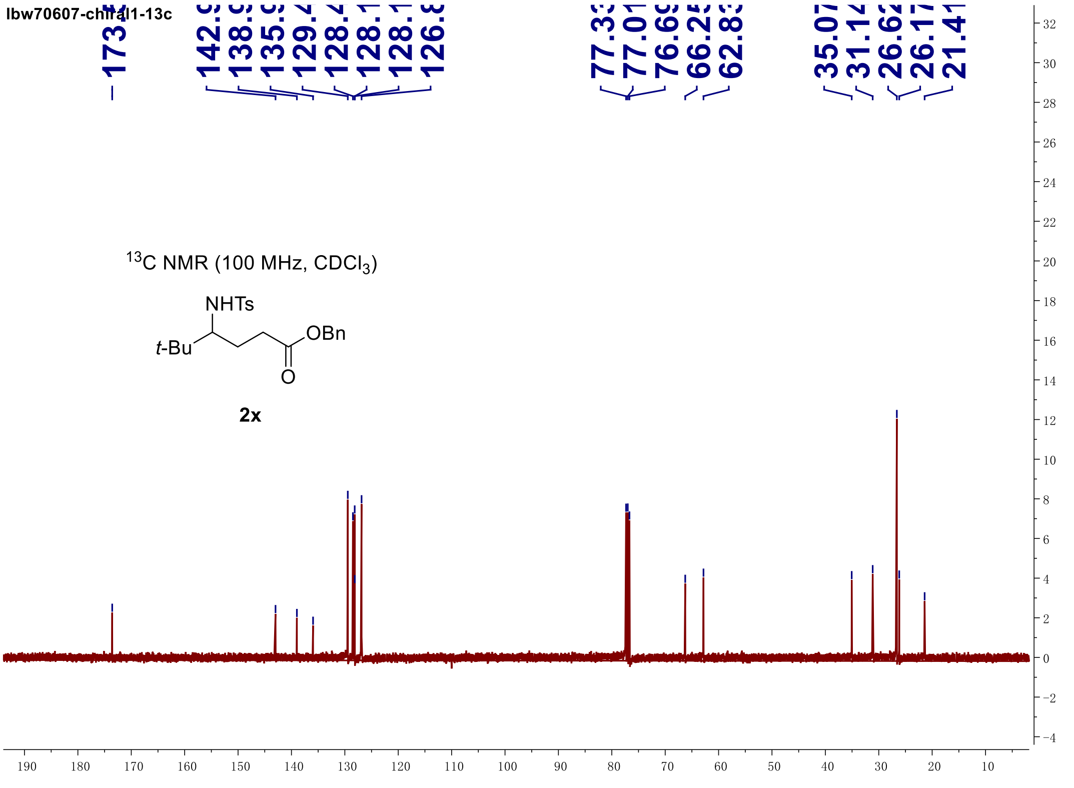

Supplementary Figure 44 ^{1}H and ^{13}C NMR spectrum of compound **2x** in $CDCl_3$

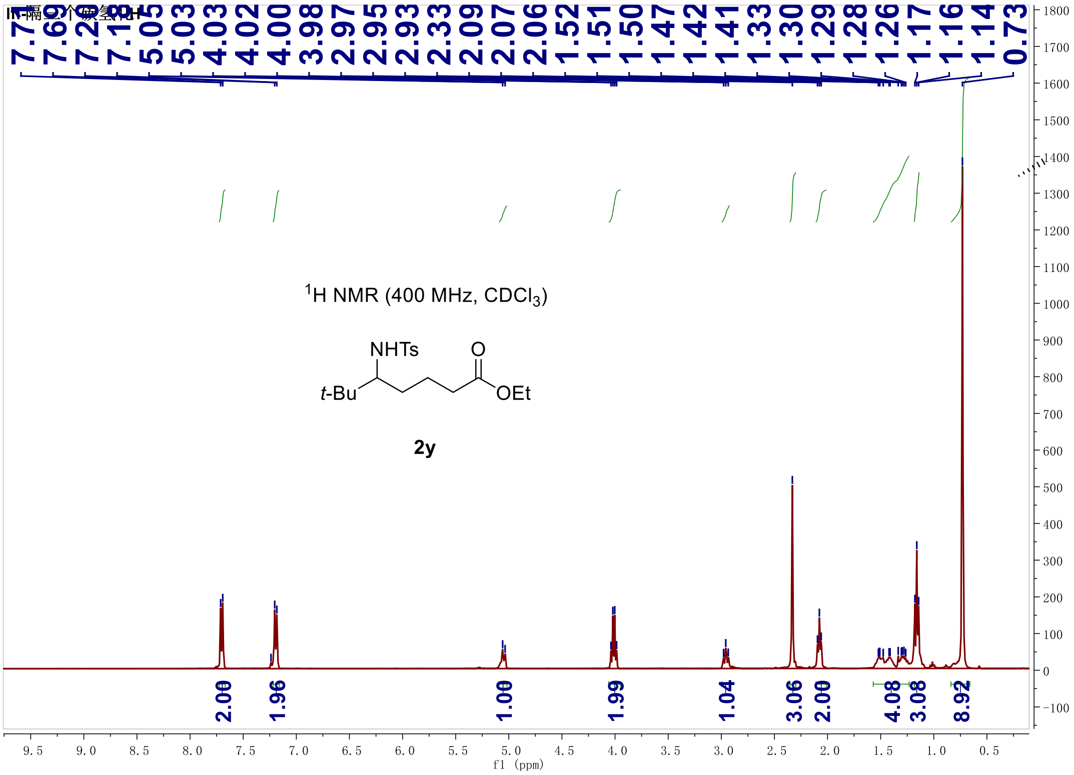

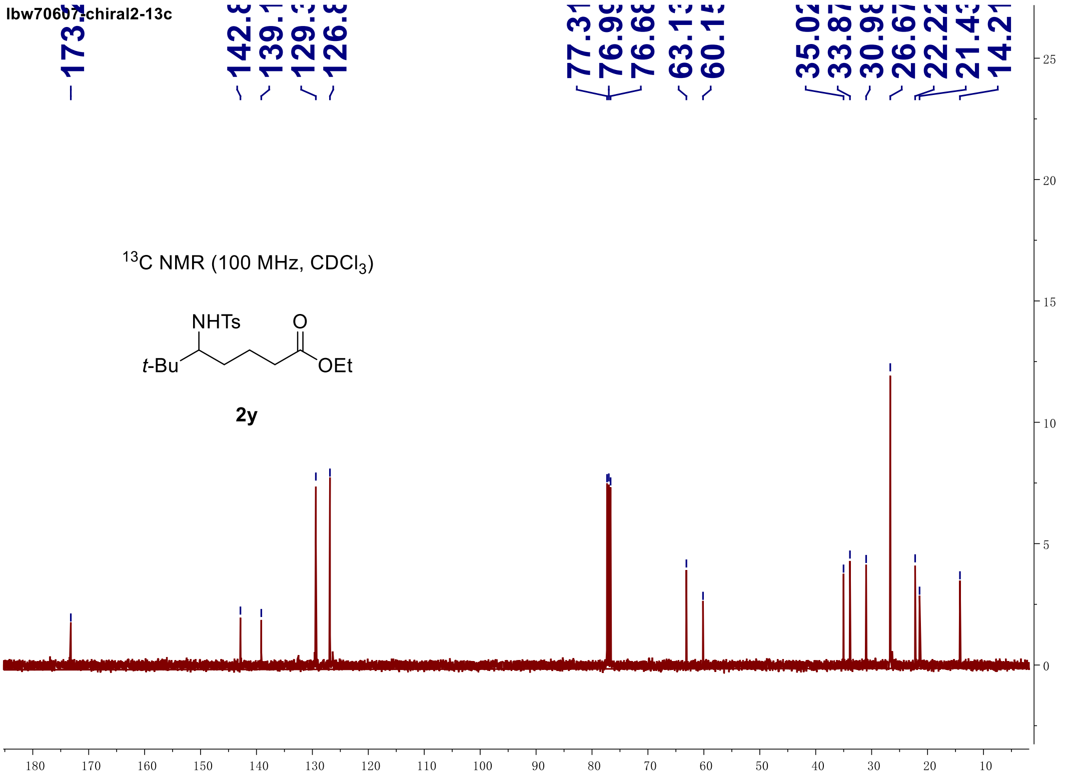

Supplementary Figure 45 ^{1}H and ^{13}C NMR spectrum of compound **2y** in $CDCl_3$

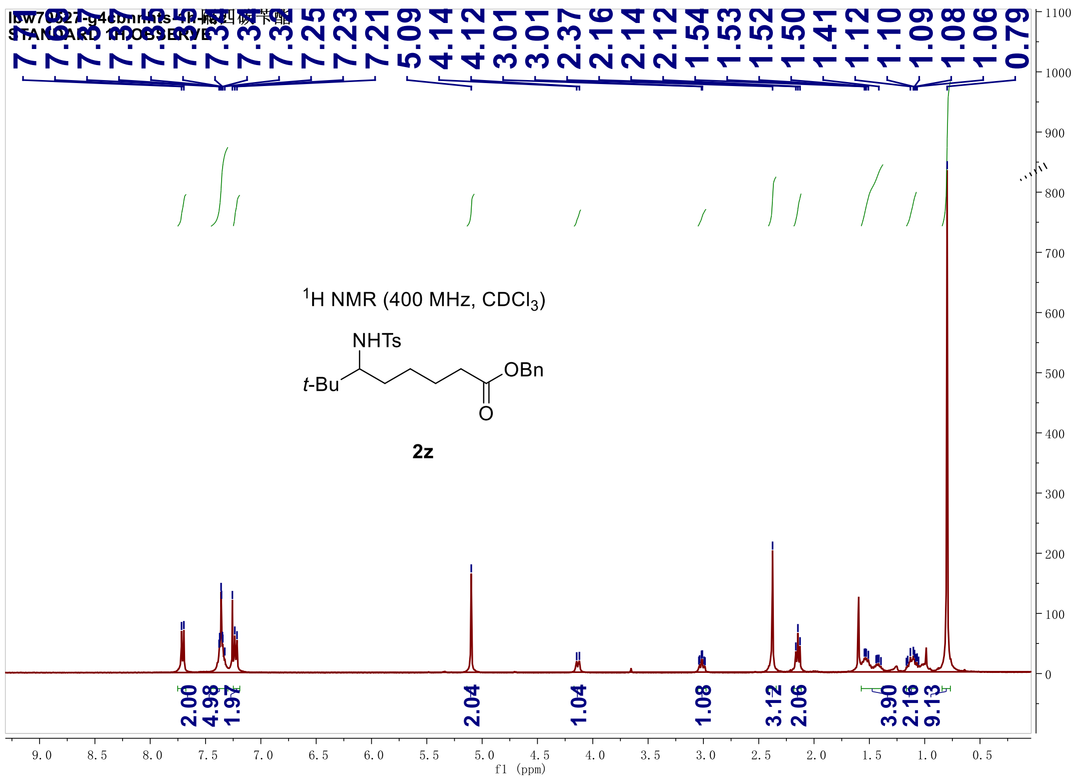

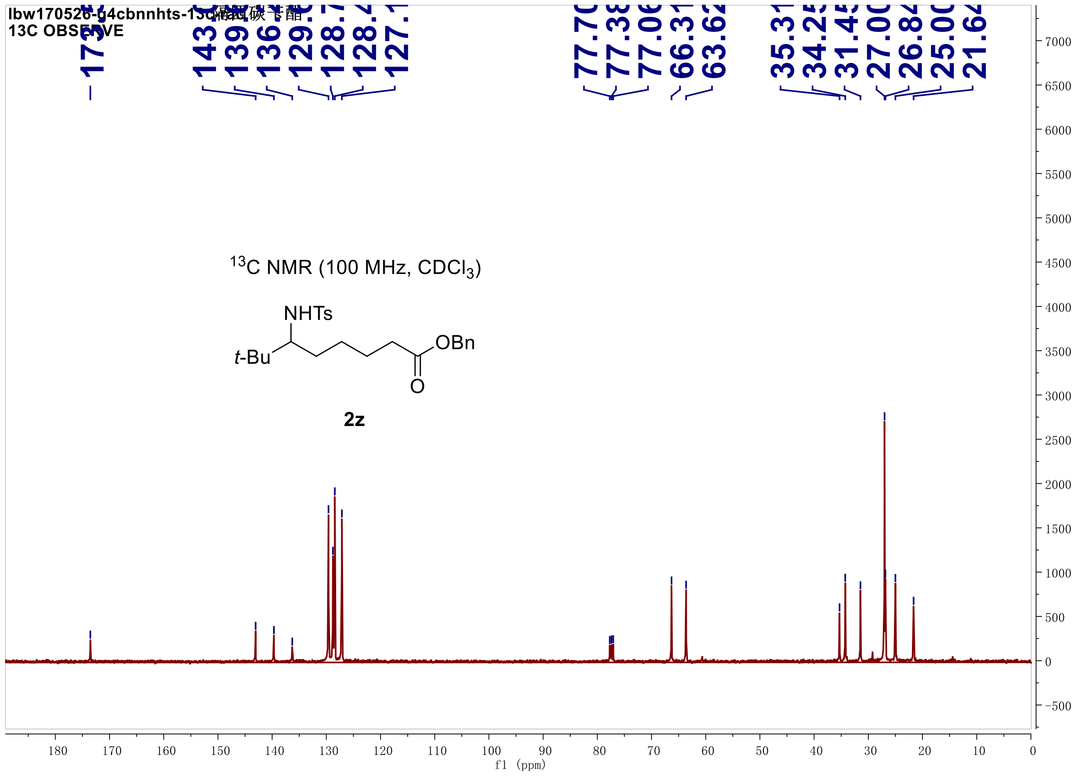

Supplementary Figure 46 ^{1}H and ^{13}C NMR spectrum of compound **2z** in CDCl3

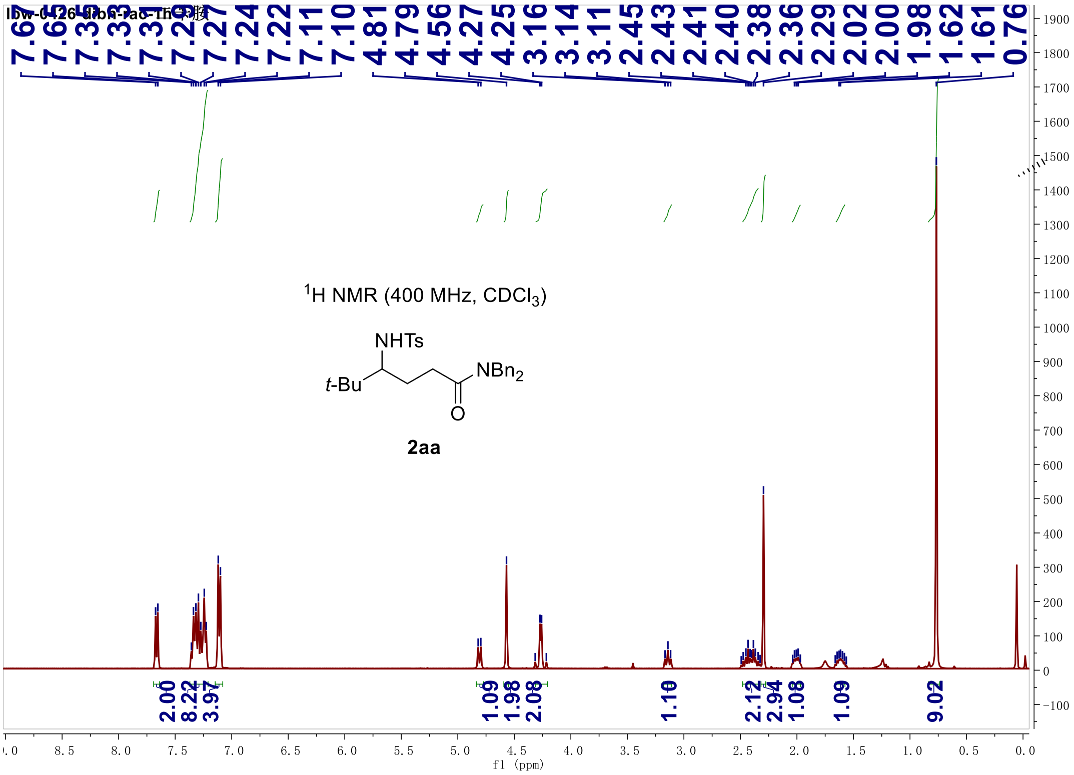

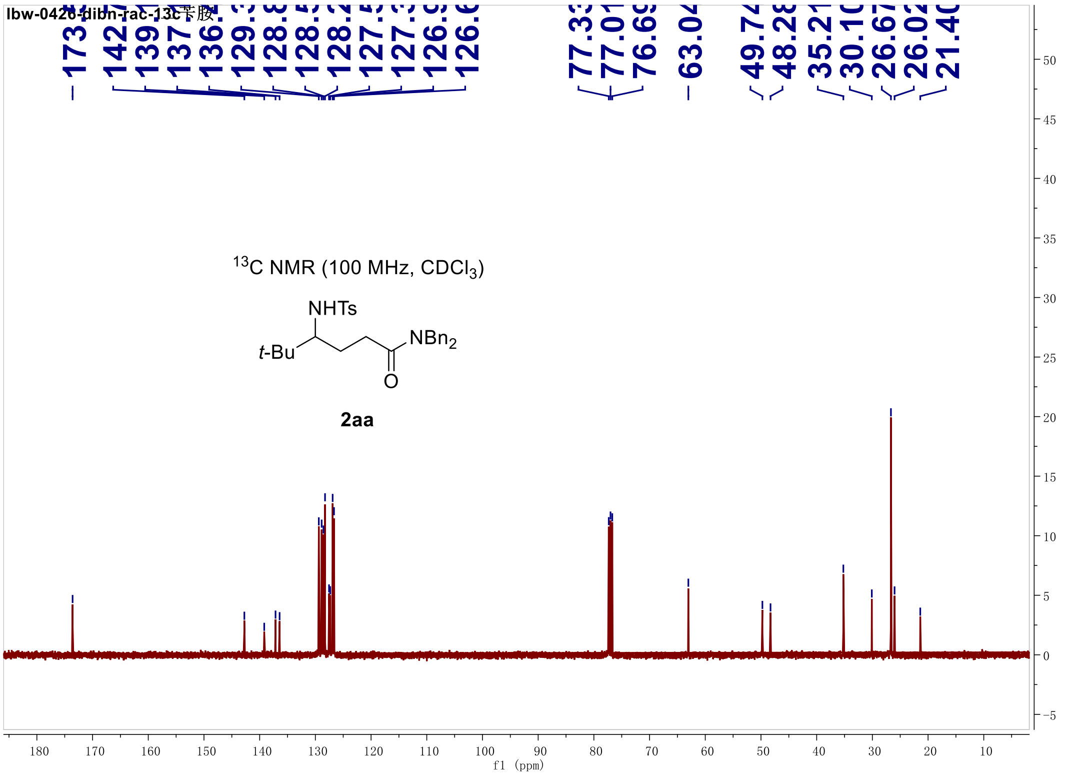

Supplementary Figure 47 ^{1}H and ^{13}C NMR spectrum of compound **2aa** in $CDCl_3$

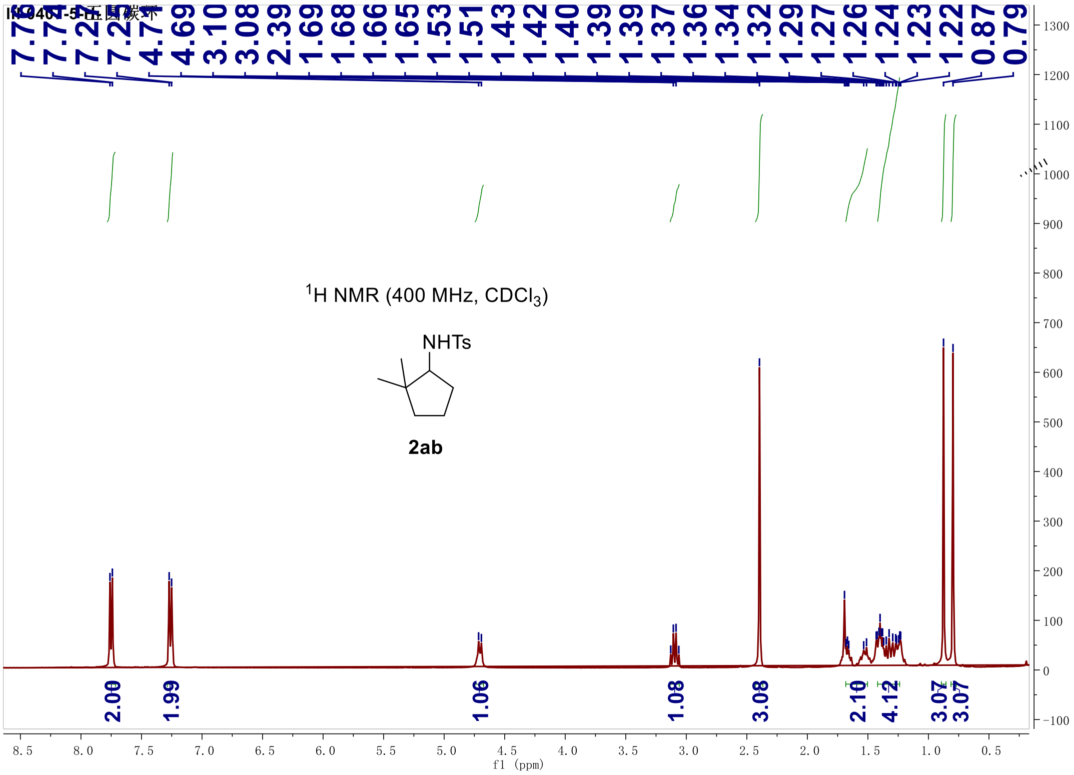

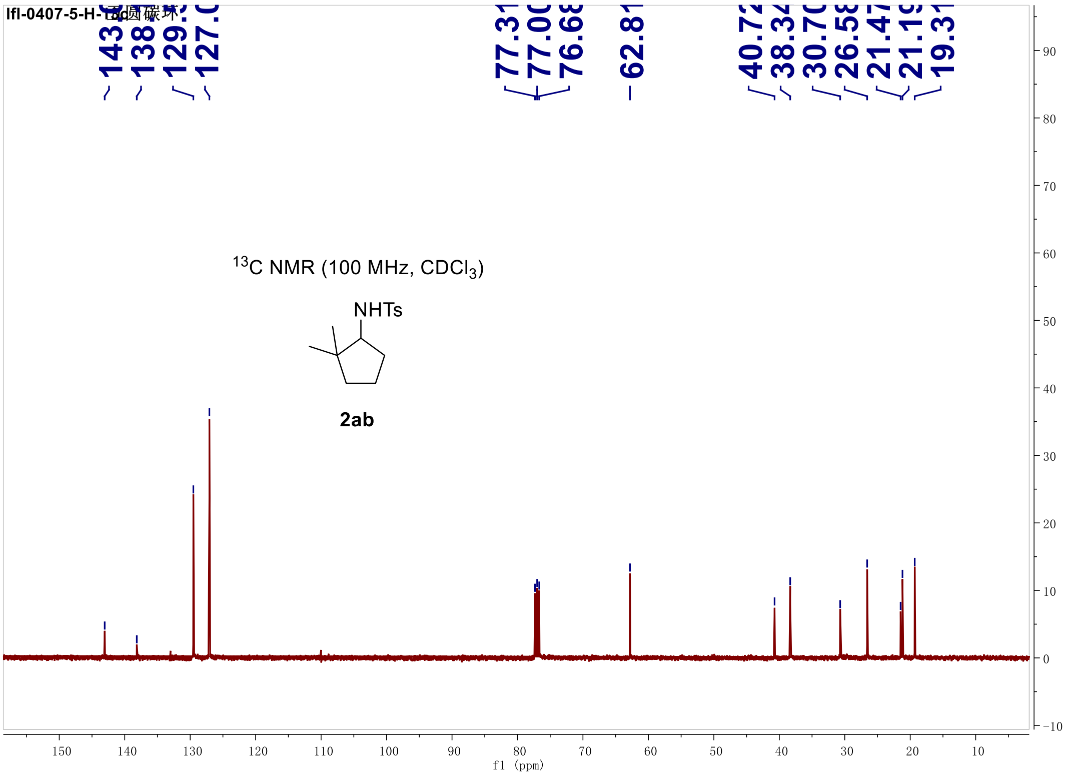

Supplementary Figure 48 ^{1}H and ^{13}C NMR spectrum of compound **2ab** in $CDCl_3$

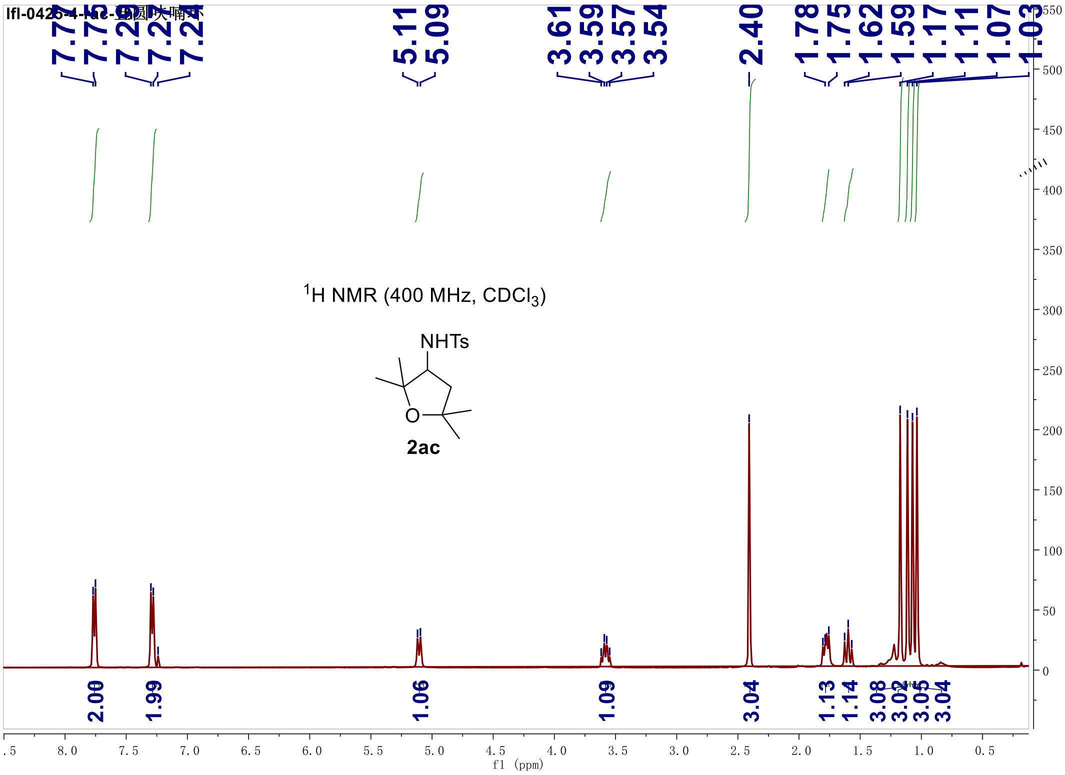

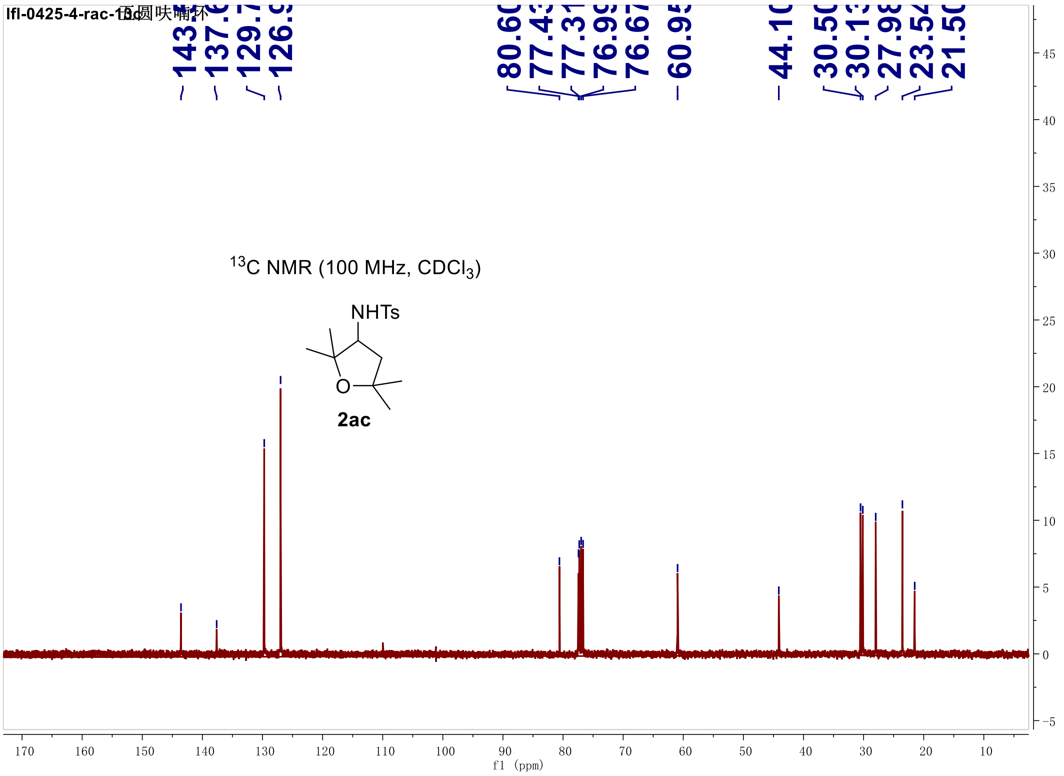

Supplementary Figure 49 ^{1}H and ^{13}C NMR spectrum of compound **2ac** in CDCl3

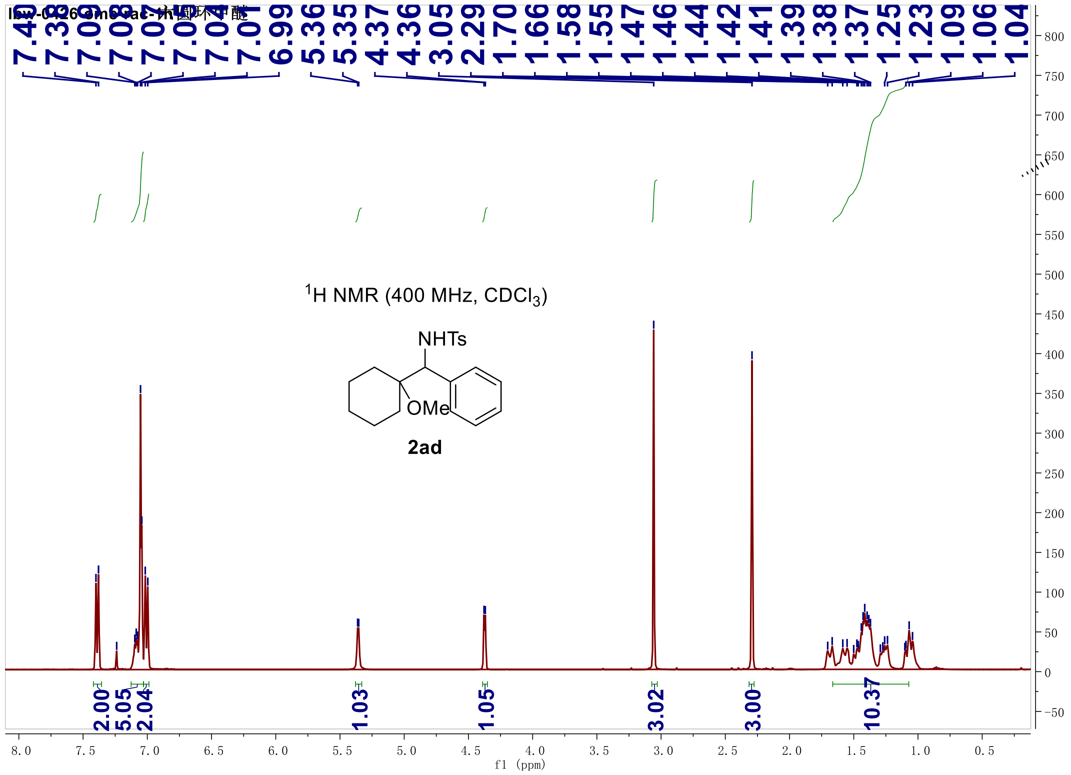

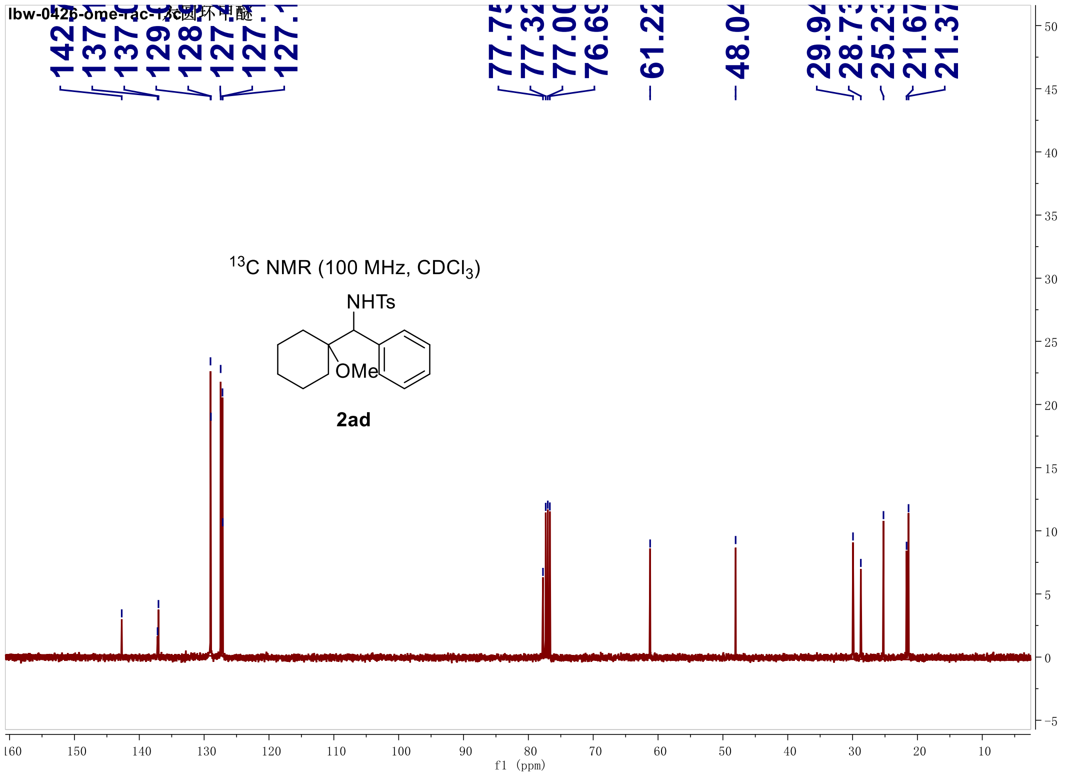

Supplementary Figure 50 ^{1}H and ^{13}C NMR spectrum of compound **2ad** in CDCl3

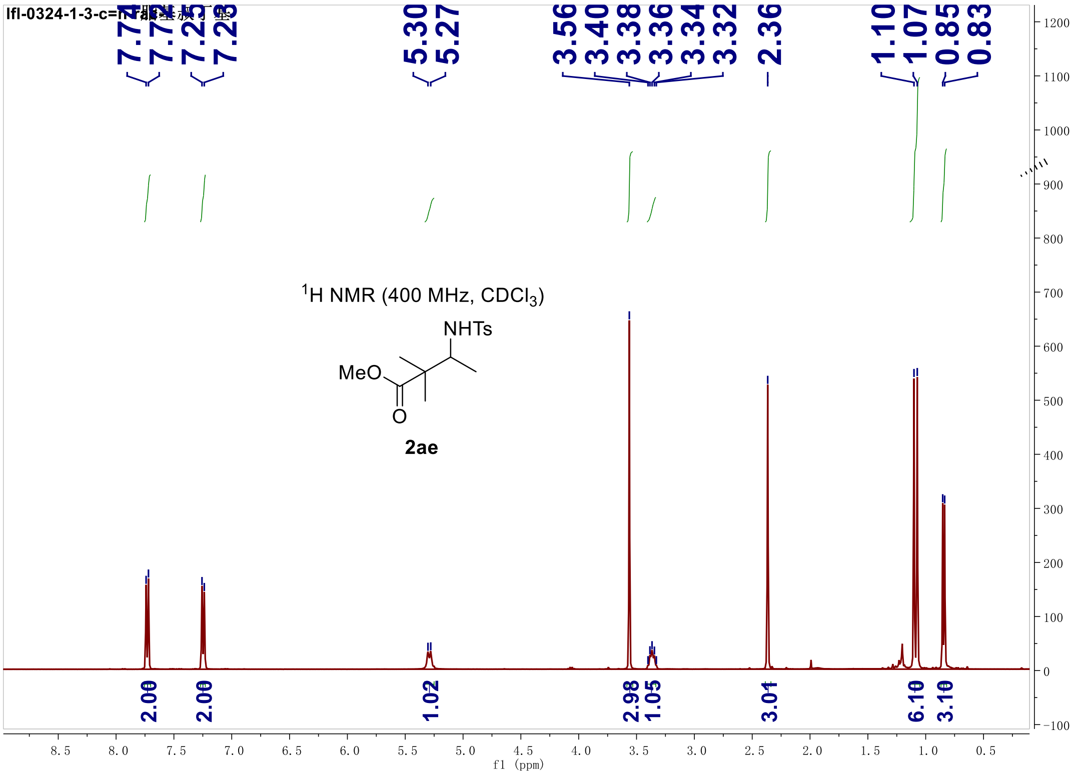

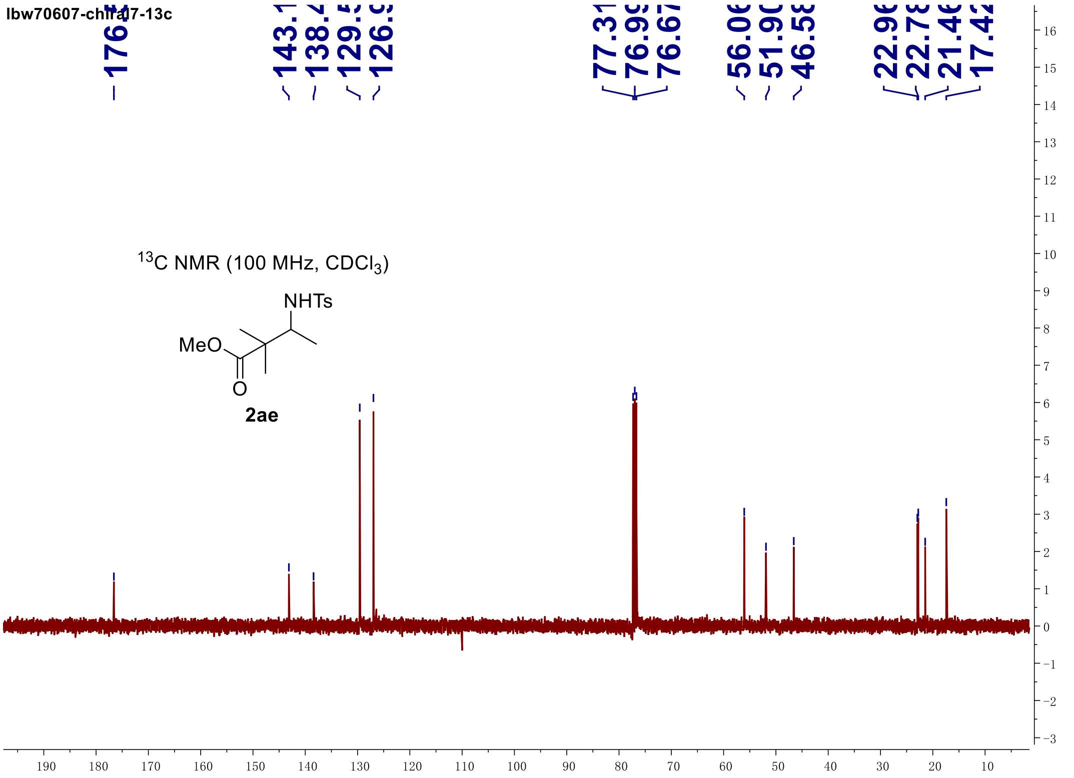

Supplementary Figure 51 ^{1}H and ^{13}C NMR spectrum of compound **2ae** in $CDCl_3$

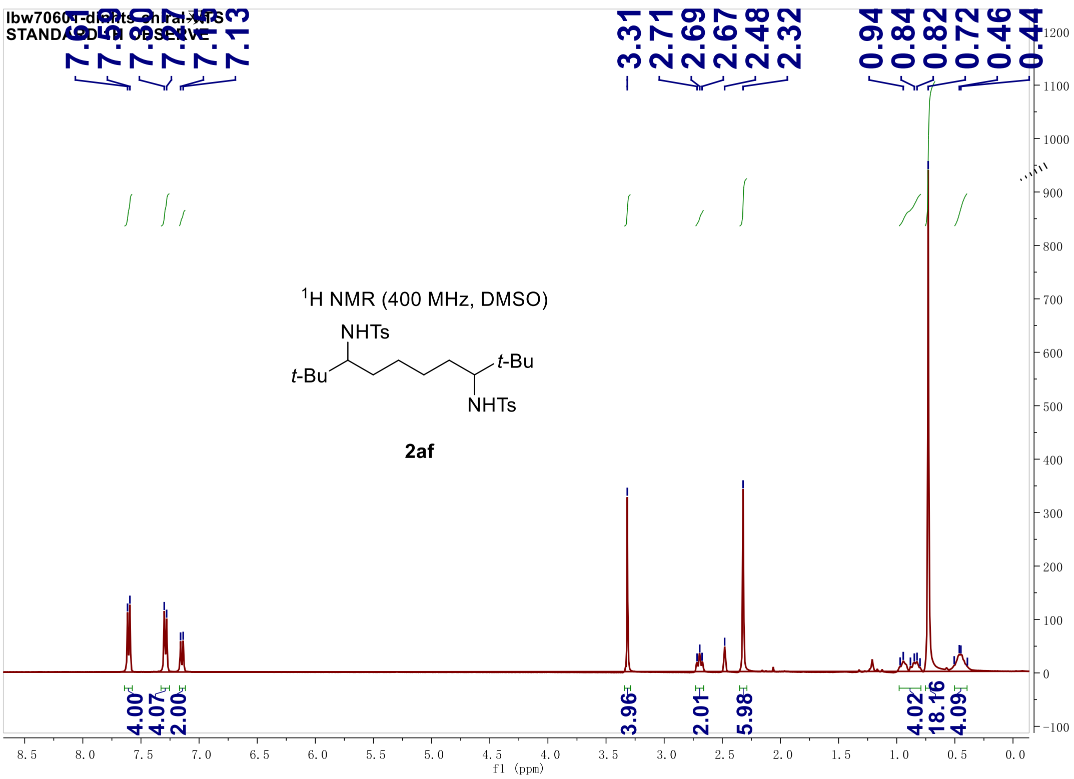

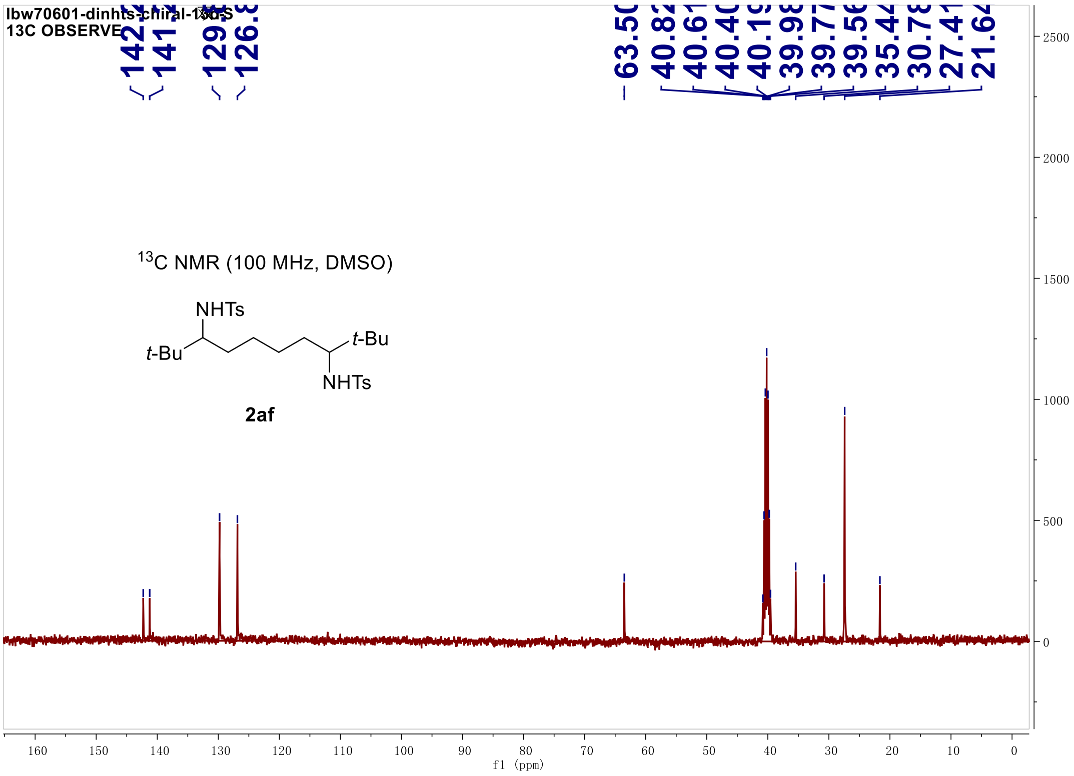

Supplementary Figure 52 ^{1}H and ^{13}C NMR spectrum of compound **2af** in DMSO

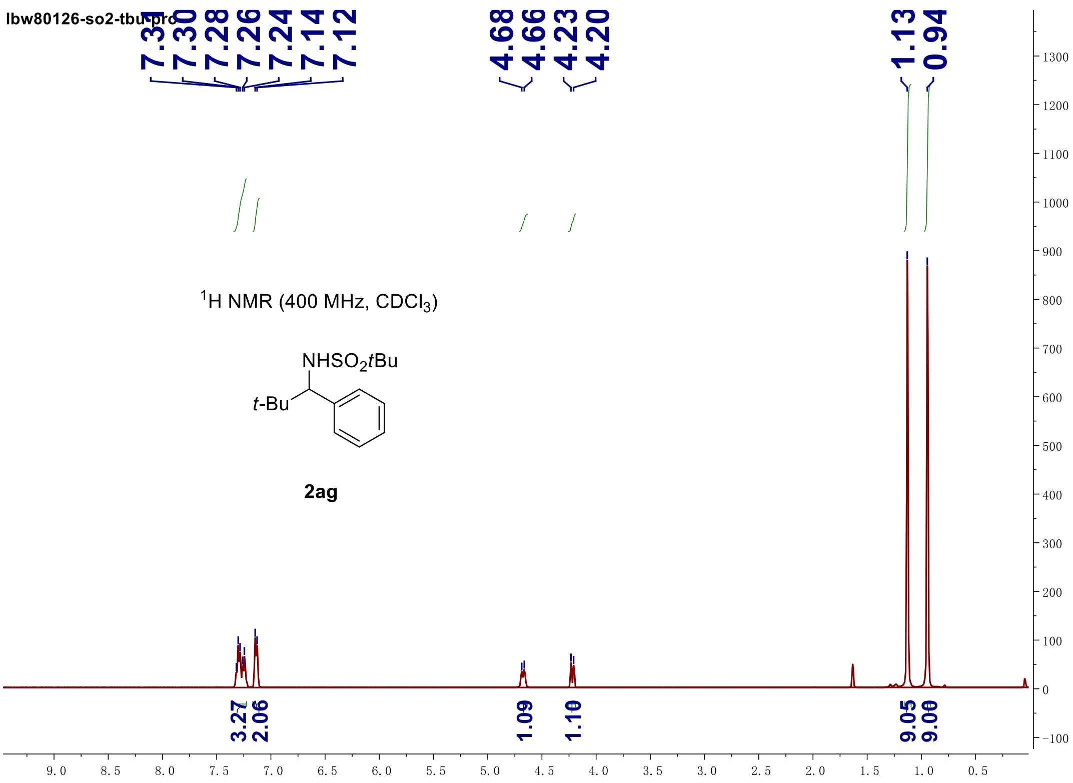

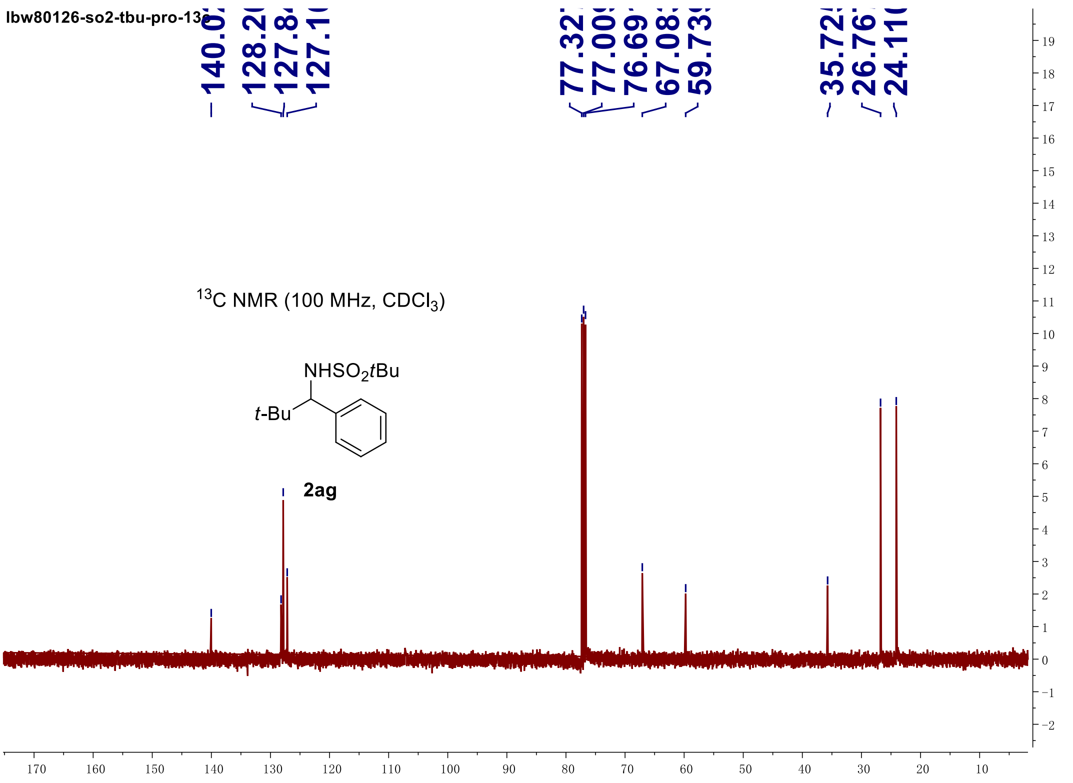

Supplementary Figure 53 ^{1}H and ^{13}C NMR spectrum of compound **2ag** in CDCl3

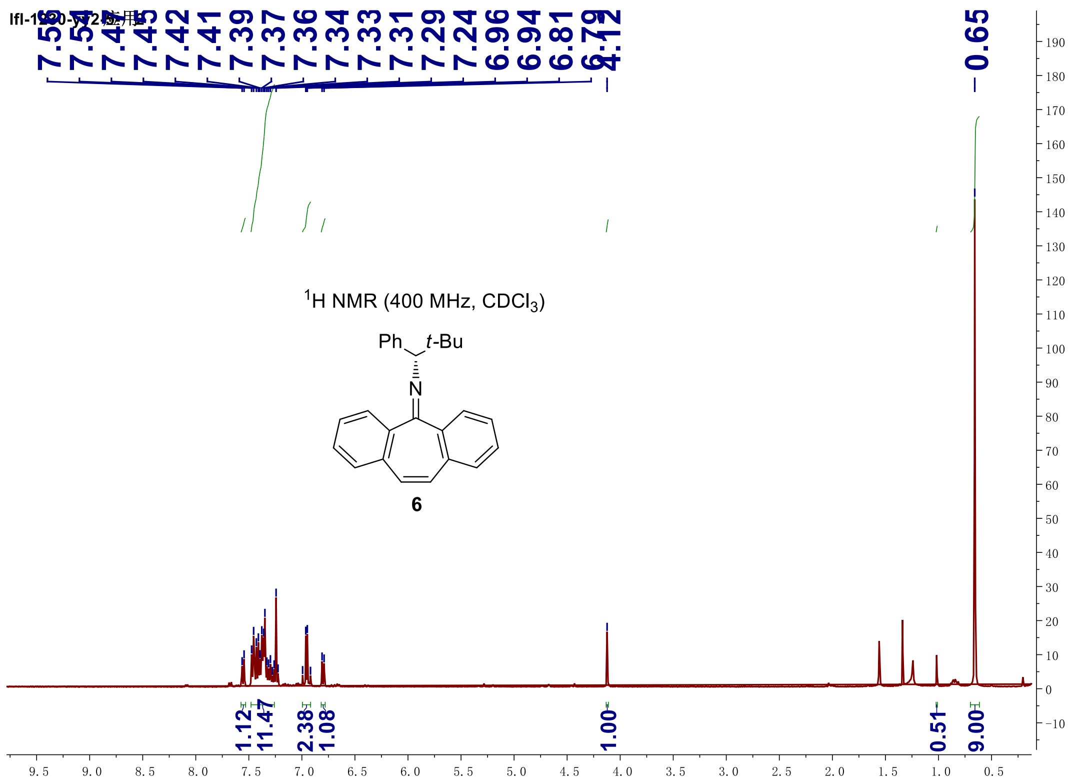

Supplementary Figure 54 ^{1}H NMR spectrum of compound **6** in CDCl3

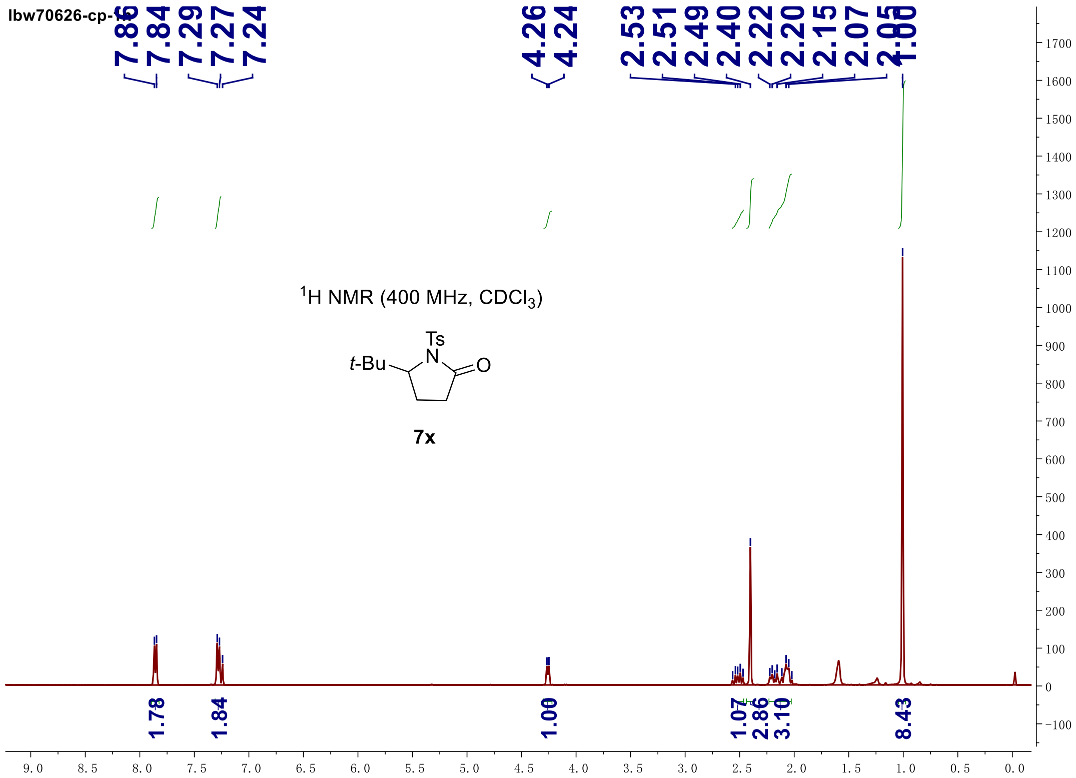

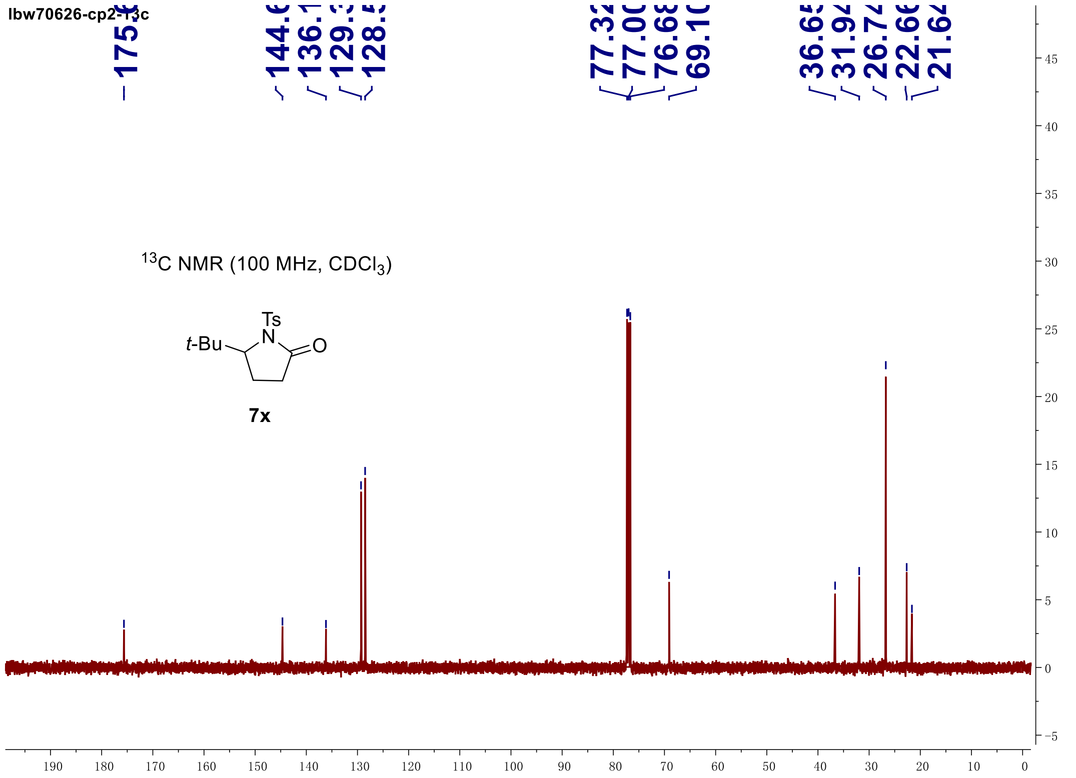

Supplementary Figure 55 ^{1}H and ^{13}C NMR spectrum of compound **7x** in $CDCl_3$

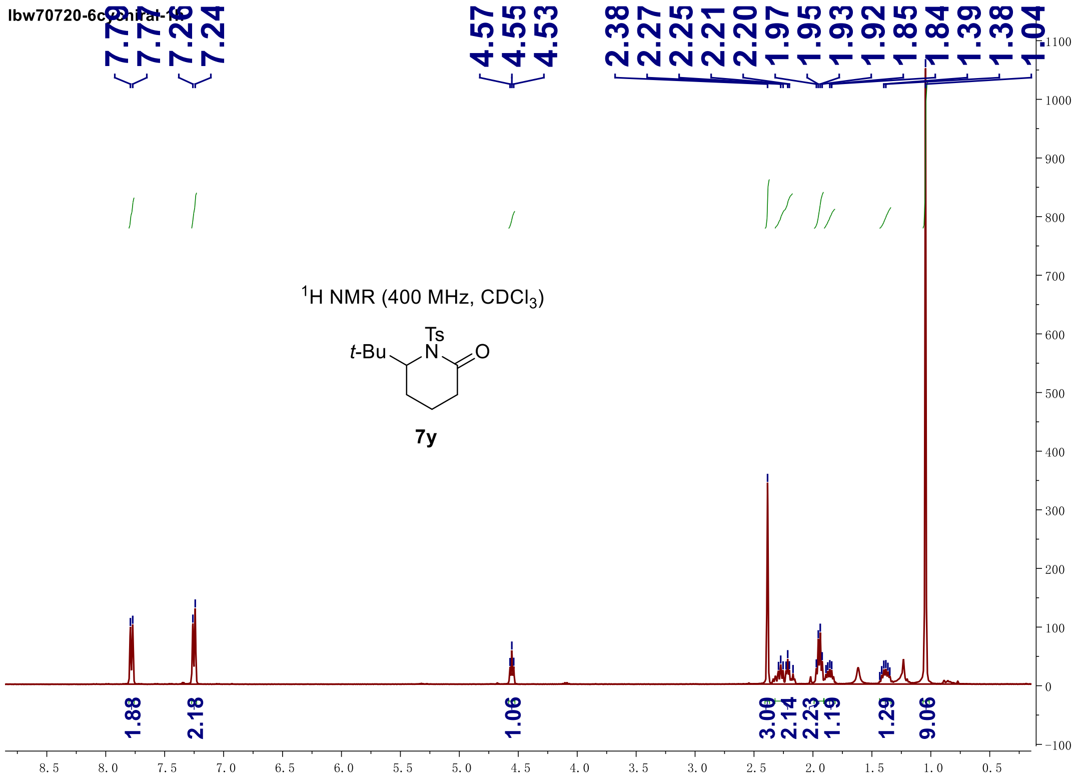

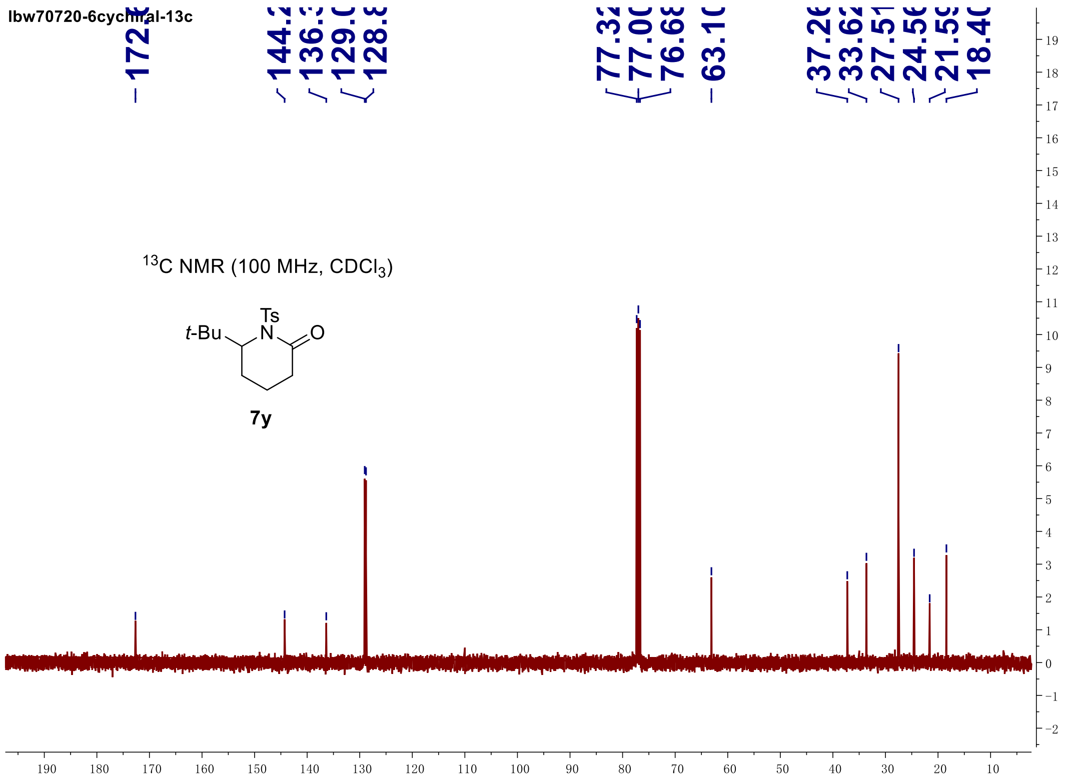

Supplementary Figure 56 ^{1}H and ^{13}C NMR spectrum of compound **7y** in $CDCl_3$

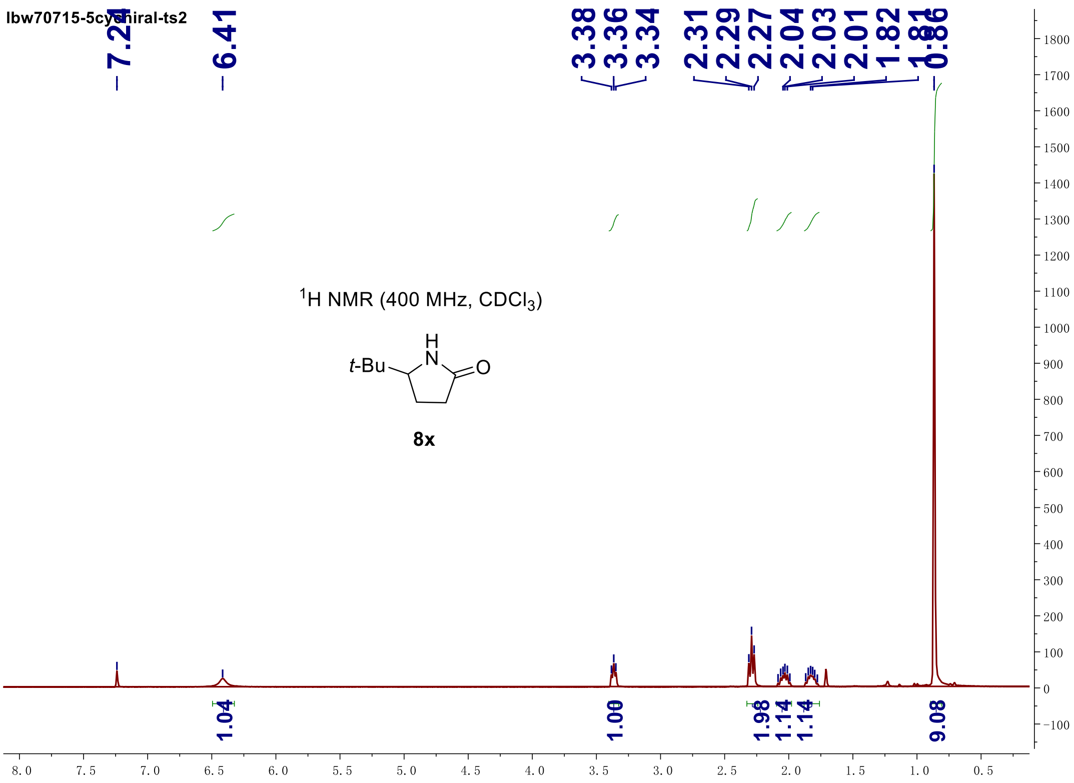

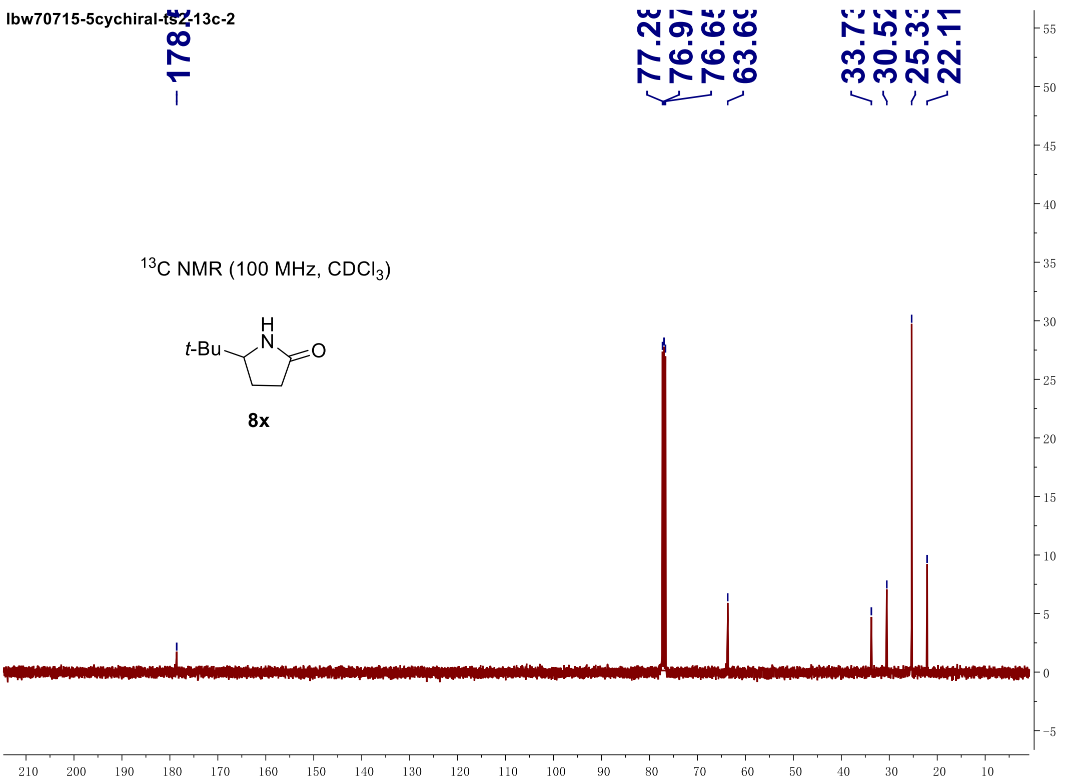

Supplementary Figure 57 ^{1}H and ^{13}C NMR spectrum of compound **8x** in CDCl3

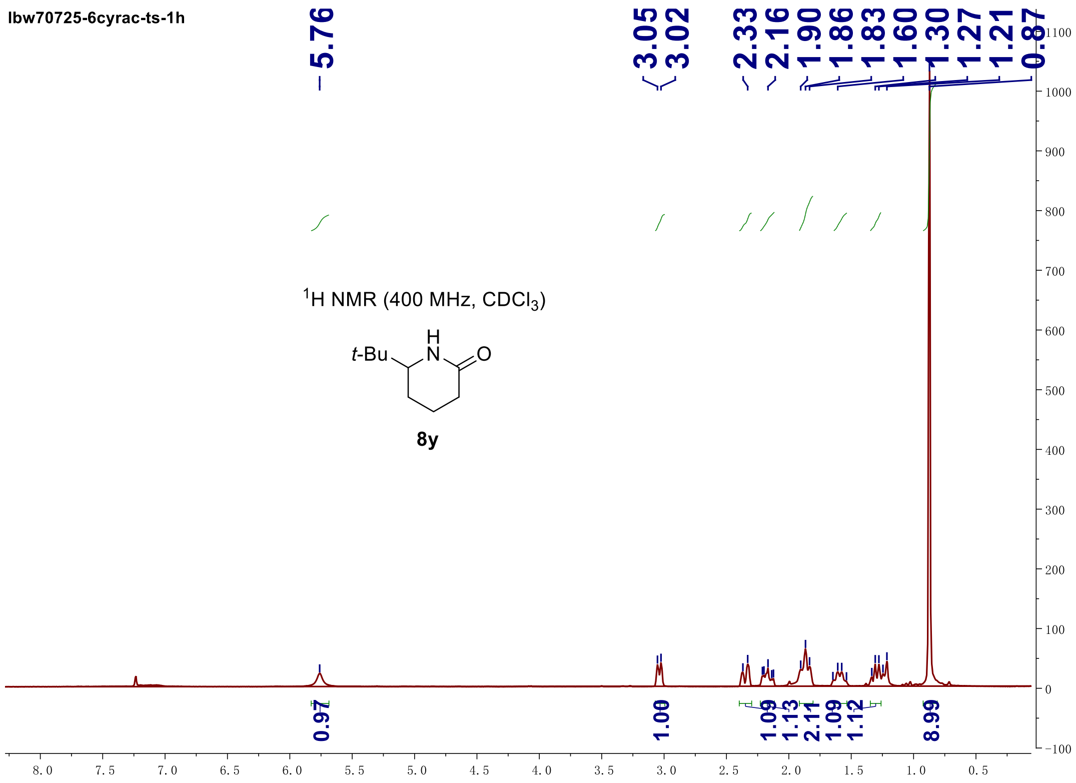

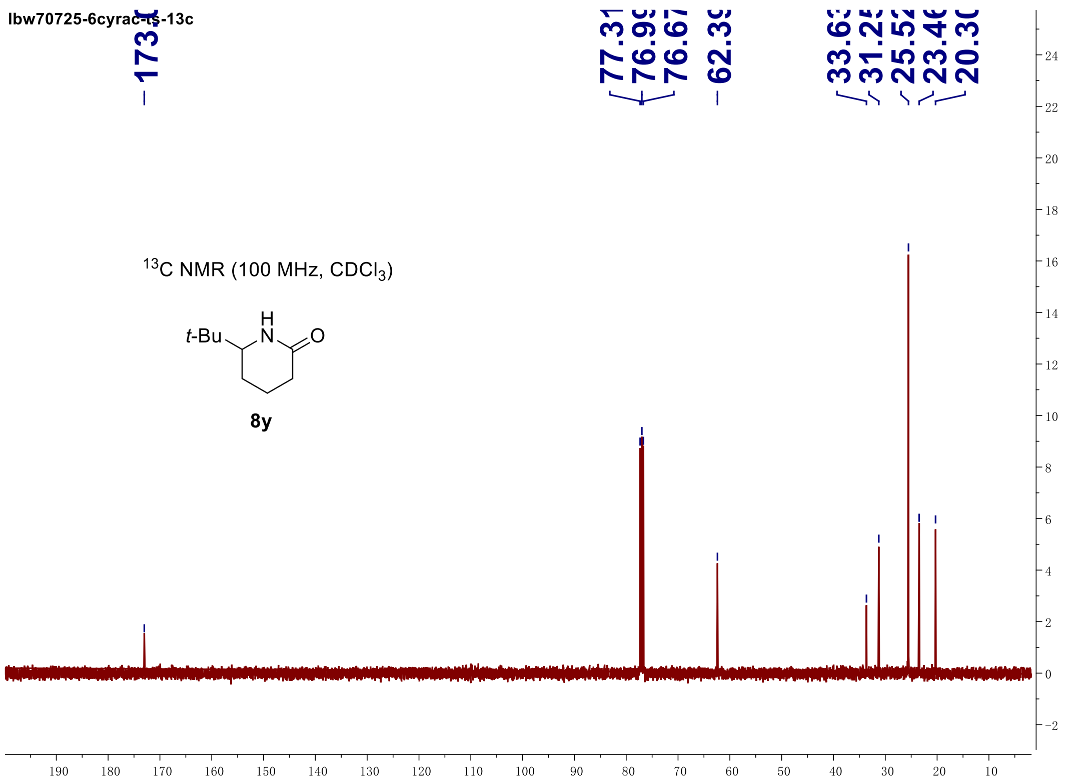

Supplementary Figure 58 ^{1}H and ^{13}C NMR spectrum of compound **8y** in CDCl3

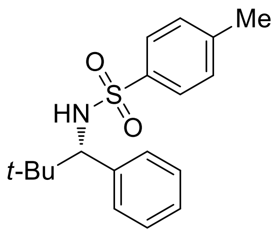

(*S*)-*N*-(2,2-Dimethyl-1-phenylpropyl)-4-methylbenzenesulfonamide (2a)

99.9% ee, enantiomeric excess was determined by HPLC, DAICEL Chiralpak OJ column, Hexane/*i*-PrOH = 70/30, 220 nm, 0.7 mL/min.

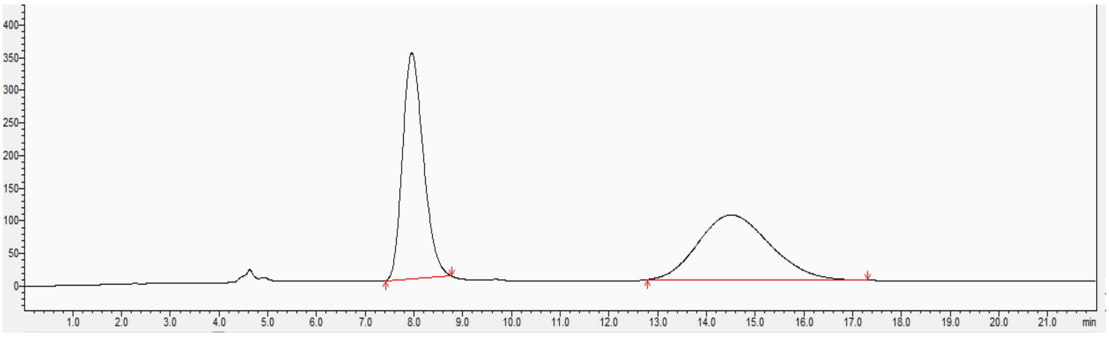

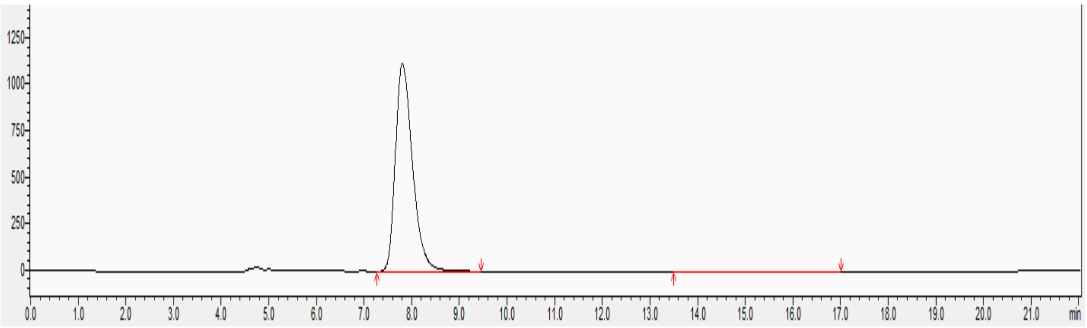

	Retention Time (min)	Area (%)
**Peak 1**	7.807	99.976
**Peak 2**	15.201	0.024

Supplementary Figure 59 HPLC spectra for product **2a**

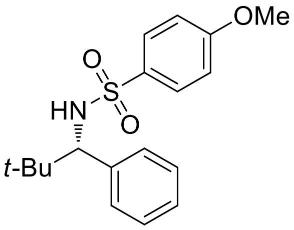

(*S*)-*N*-(2,2-Dimethyl-1-phenylpropyl)-4-methoxybenzenesulfonamide (2b)

99.0% ee, enantiomeric excess was determined by HPLC, DAICEL Chiralpak OJ column, Hexane/*i*-PrOH = 70/30, 230 nm, 1.0 mL/min.

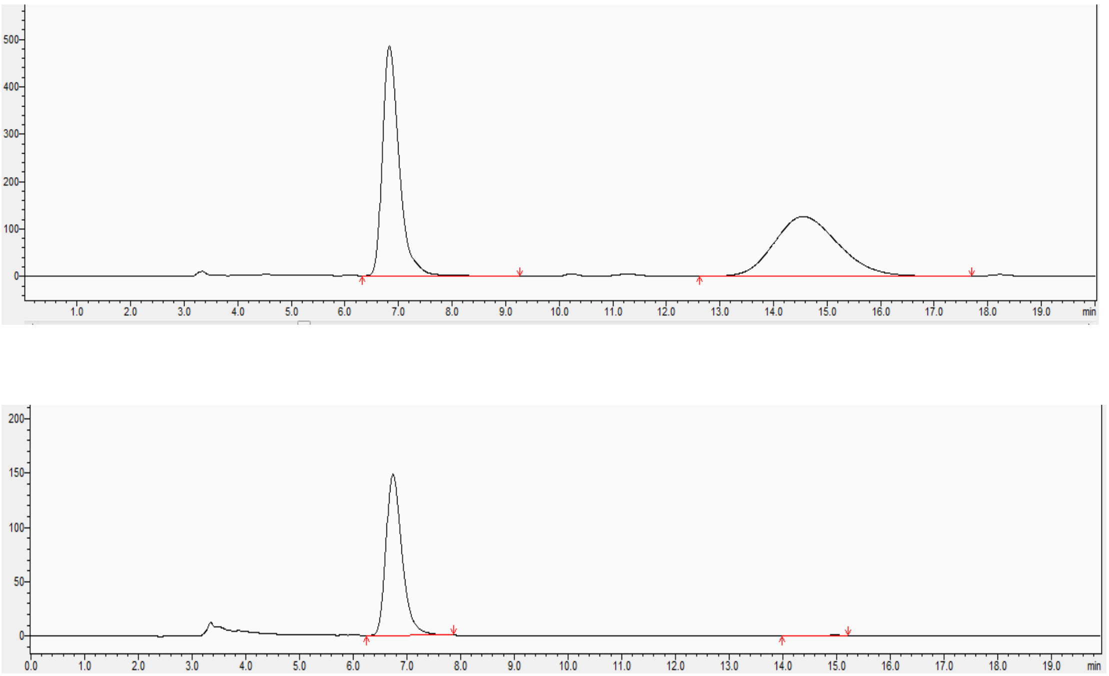

	Retention Time (min)	Area (%)
**Peak 1**	6.741	99.463
**Peak 2**	14.974	0.537

Supplementary Figure 60 HPLC spectra for product **2b**

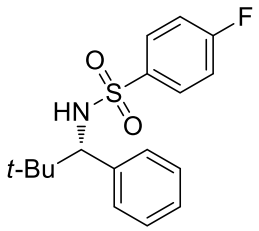

(*S*)-*N*-(2,2-Dimethyl-1-phenylpropyl)-4-fluorobenzenesulfonamide (2c)

96.0% ee, enantiomeric excess was determined by HPLC, DAICEL Chiralpak OJ column, Hexane/*i*-PrOH = 70/30, 230 nm, 1.0 mL/min.

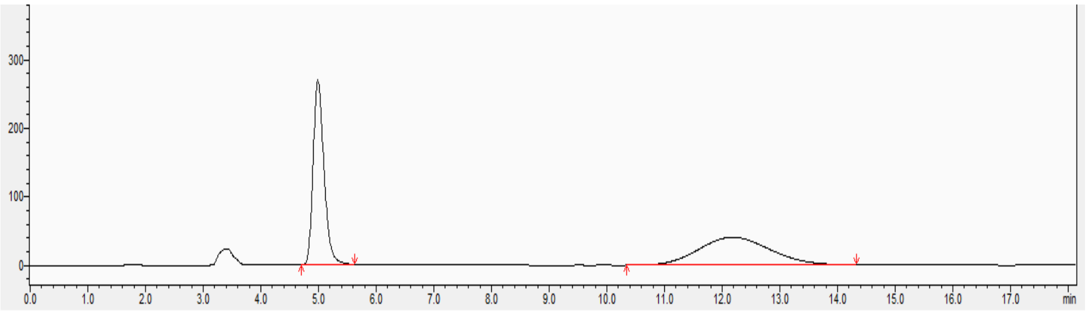

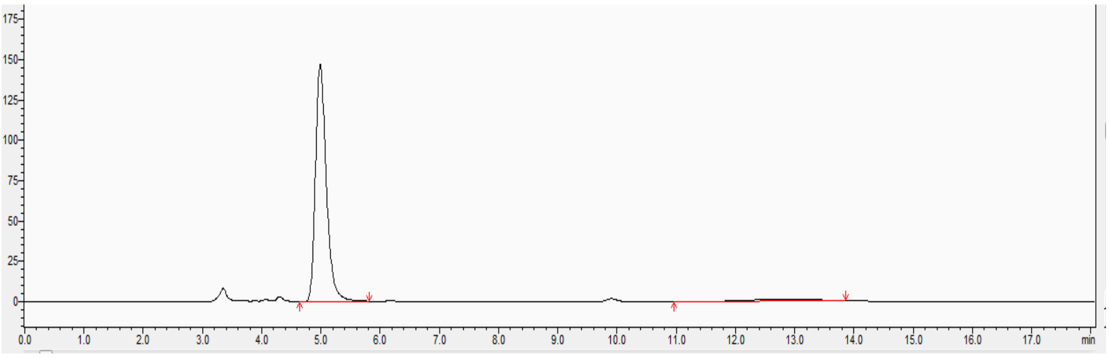

	Retention Time (min)	Area (%)
**Peak 1**	4.987	97.998
**Peak 2**	12.978	2.002

Supplementary Figure 61 HPLC spectra for product **2c**

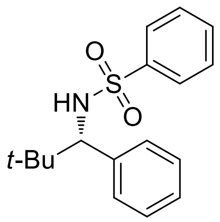

(*S*)-*N*-(2,2-Dimethyl-1-phenylpropyl)benzenesulfonamide (2d)

99.9% ee, enantiomeric excess was determined by HPLC, DAICEL Chiralpak OJ column, Hexane/*i*-PrOH = 95/5, 220 nm, 1.0 mL/min.

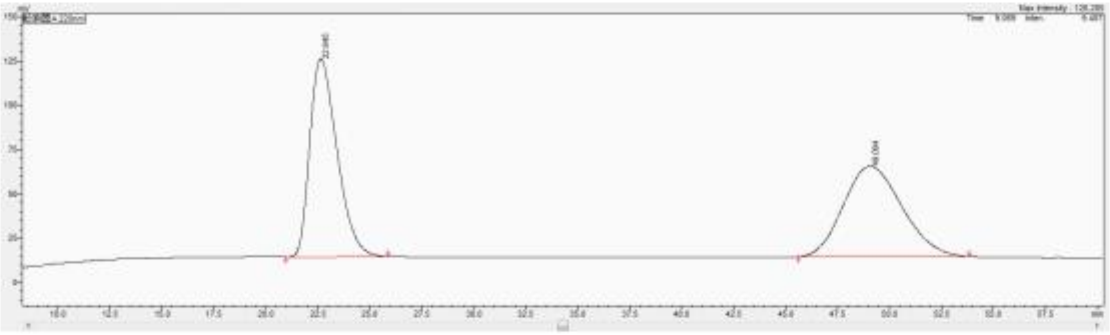

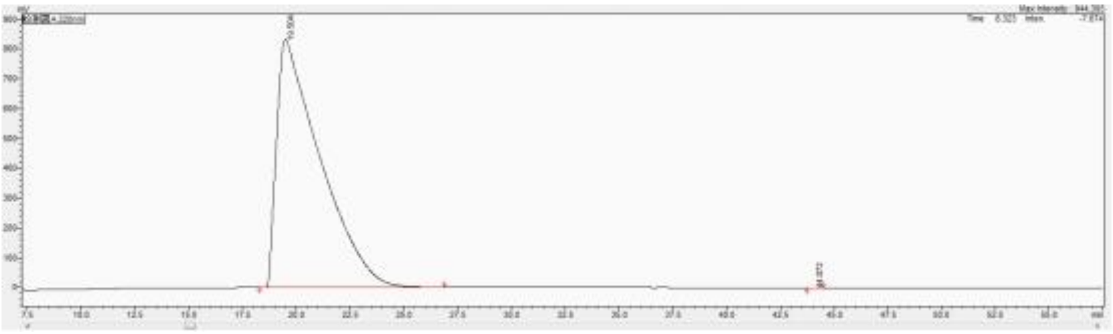

	Retention Time (min)	Area (%)
**Peak 1**	19.504	99.994
**Peak 2**	44.072	0.006

Supplementary Figure 62 HPLC spectra for product **2d**

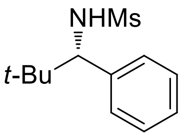

(*S*)-*N*-(2,2-Dimethyl-1-phenylpropyl)methanesulfonamide (2e)

99.1% ee, enantiomeric excess was determined by HPLC, DAICEL Chiralpak IC-3 column, Hexane/*i*-PrOH = 80/20, 210 nm, 0.6 mL/min.

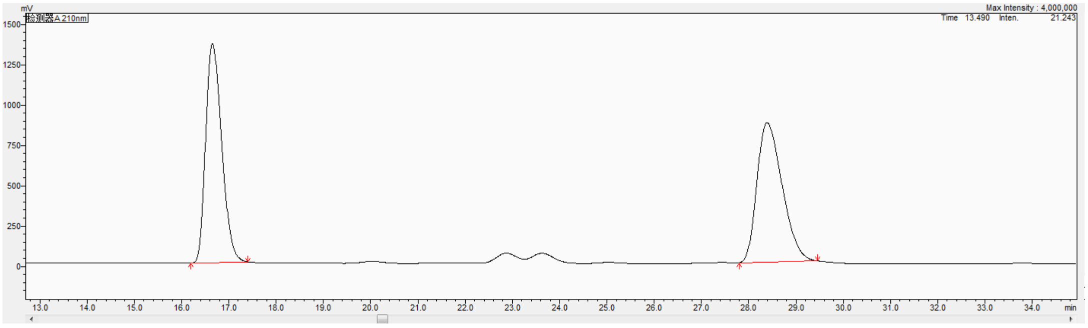

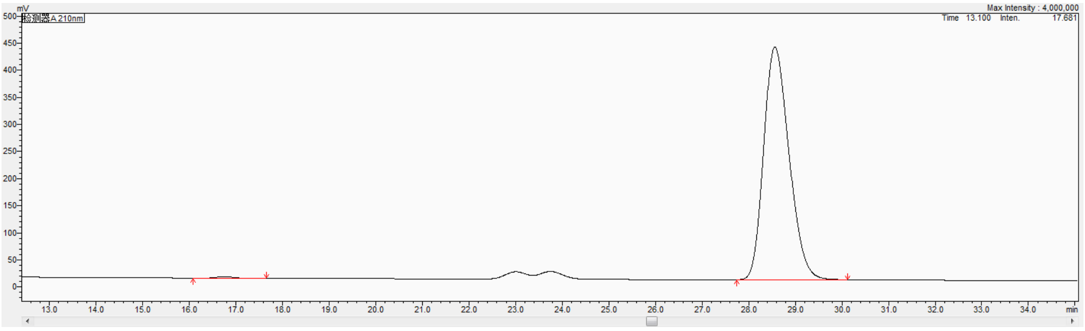

	Retention Time (min)	Area (%)
**Peak 1**	16.755	0.456
**Peak 2**	28.391	99.544

Supplementary Figure 63 HPLC spectra for product **2e**

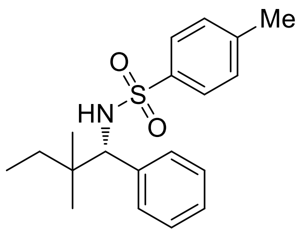

(*S*)-*N*-(2,2-Dimethyl-1-phenylbutyl)-4-methylbenzenesulfonamide (2f)

99.9% ee, enantiomeric excess was determined by HPLC, DAICEL Chiralpak OJ column, Hexane/*i*-PrOH = 90/10, 220 nm, 0.5 mL/min.

Racemate

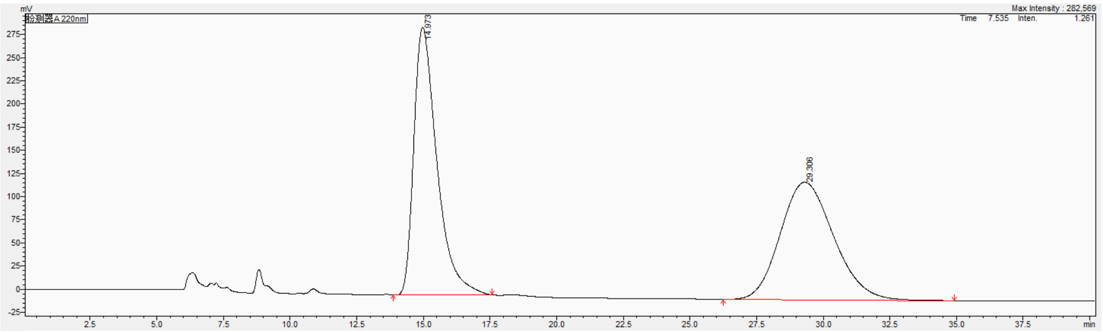

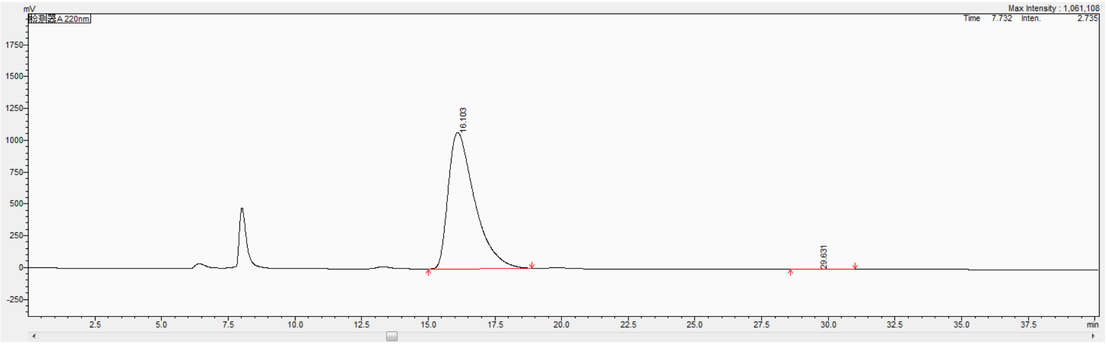

	Retention Time (min)	Area (%)
**Peak 1**	16.103	99.954
**Peak 2**	29.631	0.046

Supplementary Figure 64 HPLC spectra for product **2f**

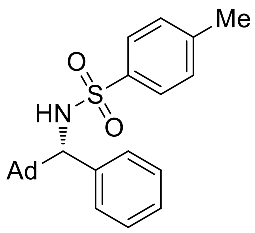

(*S*)-*N*-(Adamantan-1-yl(phenyl)methyl)-4-methylbenzenesulfonamide (2g)

99.1% ee, enantiomeric excess was determined by HPLC, DAICEL Chiralpak OD column, Hexane/*i*-PrOH = 90/10, 220 nm, 0.8 mL/min.

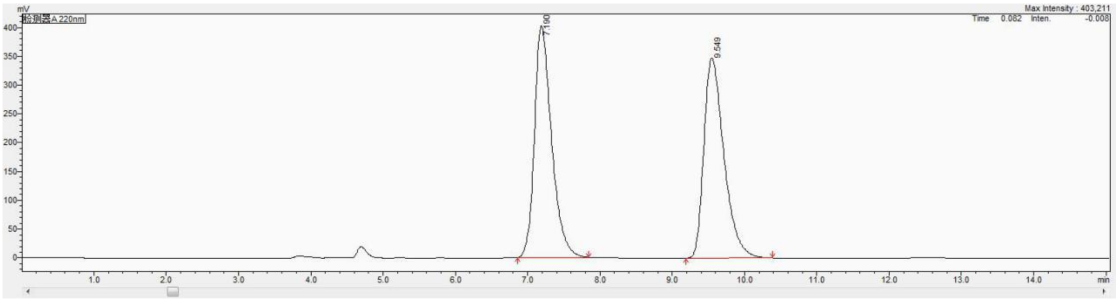

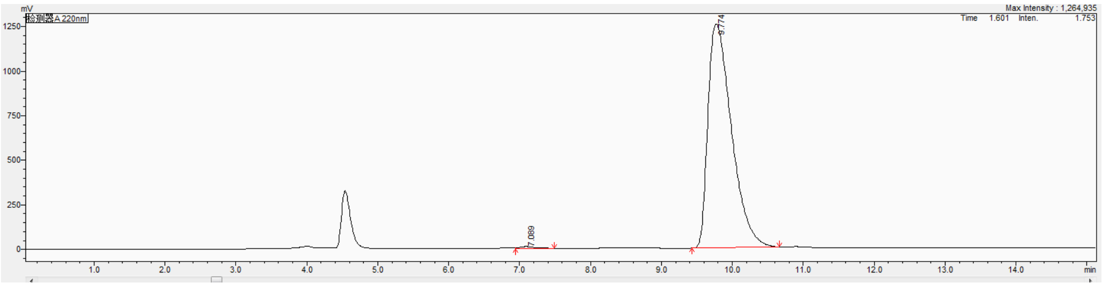

	Retention Time (min)	Area (%)
**Peak 1**	7.089	0.441
**Peak 2**	9.774	99.559

Supplementary Figure 65 HPLC spectra for product **2g**

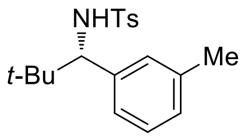

(*S*)-*N*-(2,2-Dimethyl-1-(m-tolyl)propyl)-4-methylbenzenesulfonamide (2h)

99.9% ee, enantiomeric excess was determined by HPLC, DAICEL Chiralpak OD column, Hexane/*i*-PrOH = 93/7, 220 nm, 1.0 mL/min.

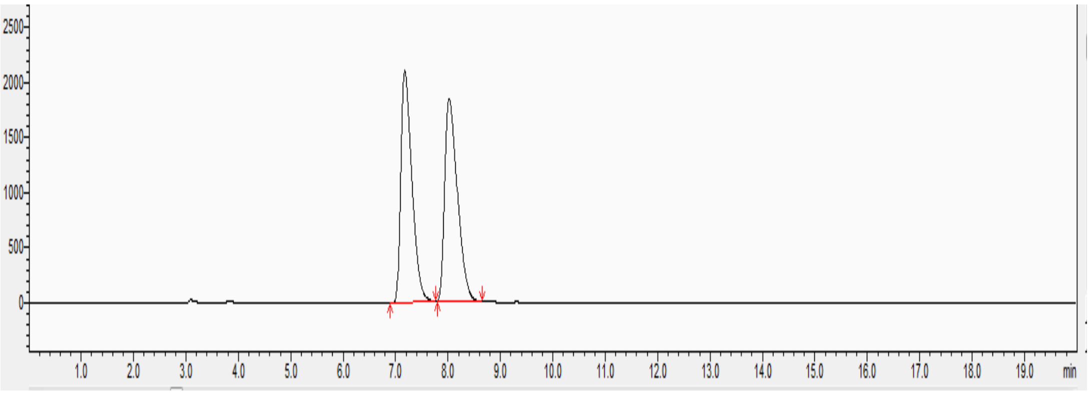

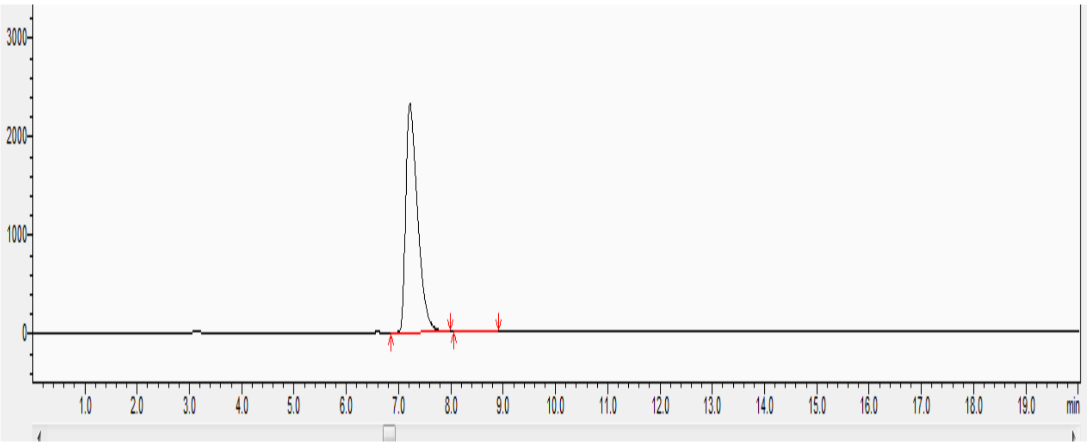

	Retention Time (min)	Area (%)
**Peak 1**	7.213	99.930
**Peak 2**	8.195	0.070

Supplementary Figure 66 HPLC spectra for product **2h**

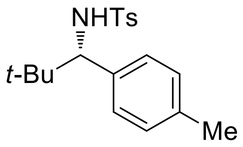

(*S*)-*N*-(2,2-Dimethyl-1-(p-tolyl)propyl)-4-methylbenzenesulfonamide (2i)

99.9% ee, enantiomeric excess was determined by HPLC, DAICEL Chiralpak OD column, Hexane/*i*-PrOH = 93/7, 220 nm, 1.0 mL/min.

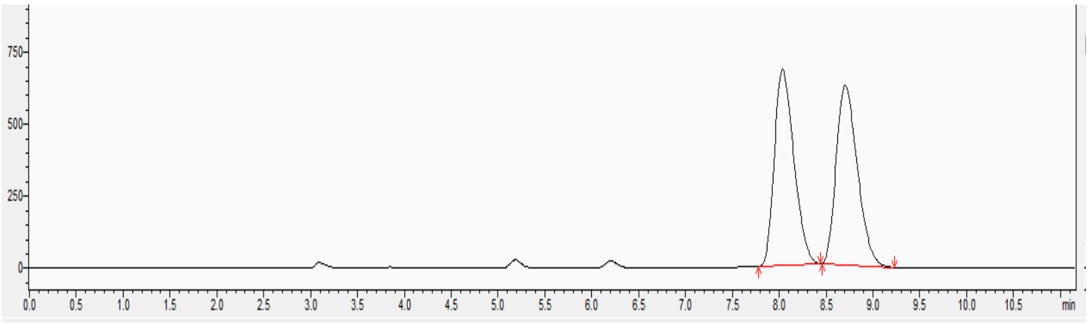

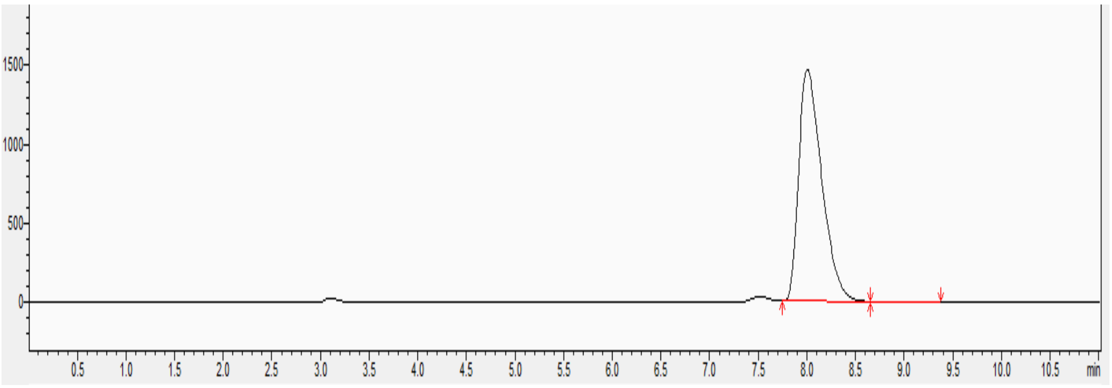

	Retention Time (min)	Area (%)
**Peak 1**	8.005	99.971
**Peak 2**	8.668	0.029

Supplementary Figure 67 HPLC spectra for product **2i**

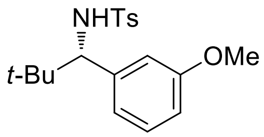

(*S*)-*N*-(1-(3-Methoxyphenyl)-2,2-dimethylpropylidene)-4-methylbenzenesulfonamide (2j)

99.9% ee, enantiomeric excess was determined by HPLC, DAICEL Chiralpak OJ column, Hexane/*i*-PrOH = 93/7, 220 nm, 1.0 mL/min.

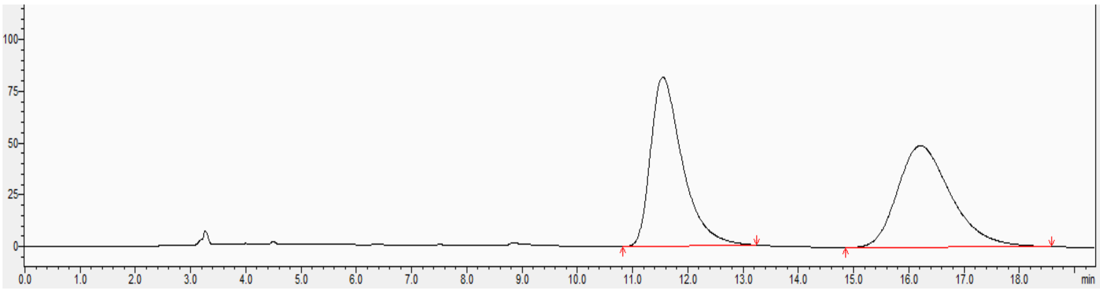

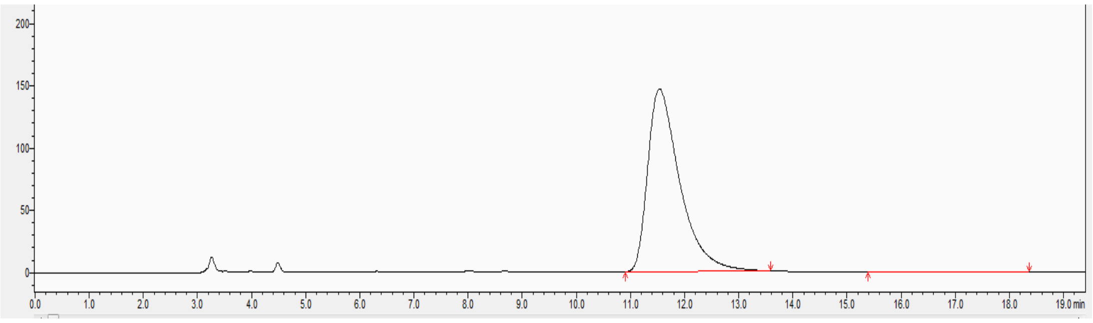

	Retention Time (min)	Area (%)
**Peak 1**	11.536	99.927
**Peak 2**	16.254	0.073

Supplementary Figure 68 HPLC spectra for product **2j**

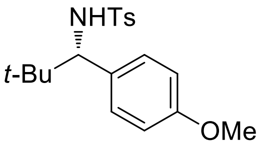

(*S*)-*N*-(1-(4-Methoxyphenyl)-2,2-dimethylpropyl)-4-methylbenzenesulfonamide (2k)

99.9% ee, enantiomeric excess was determined by HPLC, DAICEL Chiralpak OD column, Hexane/*i*-PrOH = 93/7, 220 nm, 1.0 mL/min.

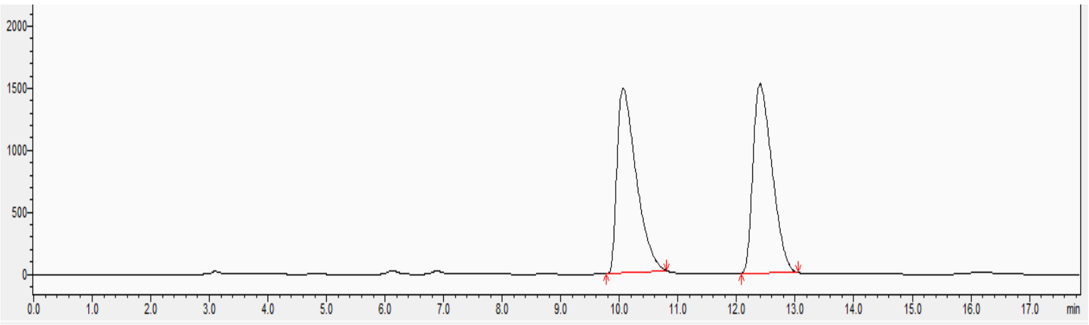

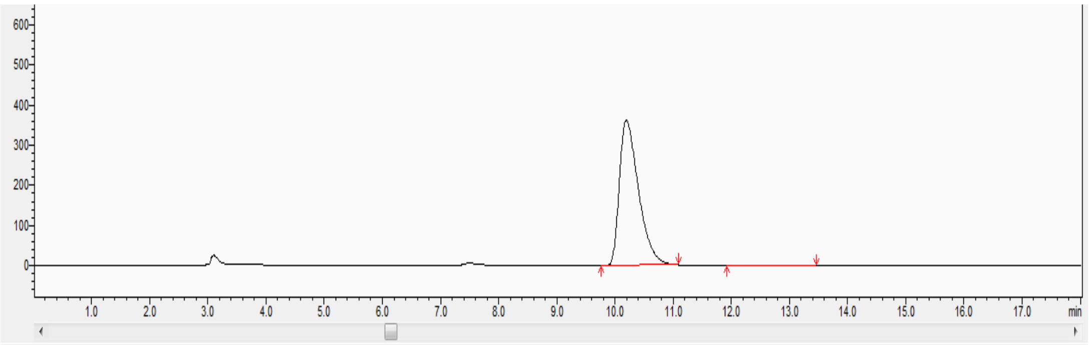

	Retention Time (min)	Area (%)
**Peak 1**	10.198	99.987
**Peak 2**	12.584	0.013

Supplementary Figure 69 HPLC spectra for product **2k**

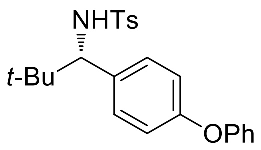

(*S*)-*N*-(2,2-Dimethyl-1-(4-phenoxyphenyl)propyl)-4-methylbenzenesulfonamide (2l)

99.9 ee%, entiomeric excess was determined by HPLC, DAICEL Chiralpak OD column, Hexane/*i*-PrOH = 96/4, 220 nm, 1.0 mL/min.

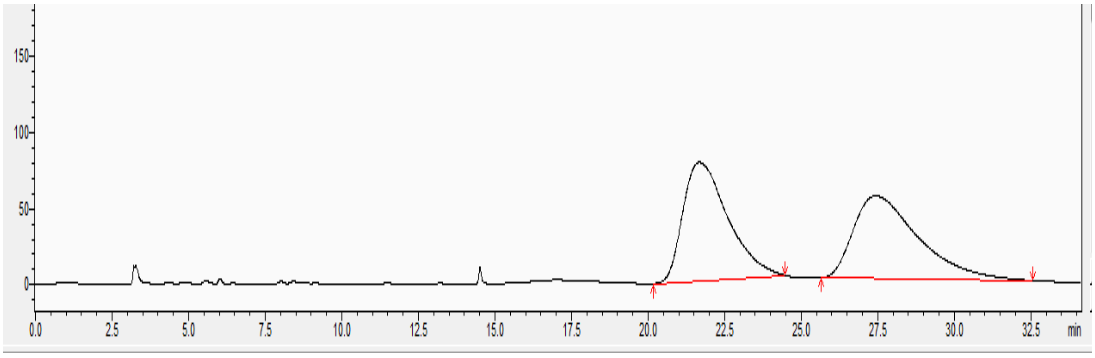

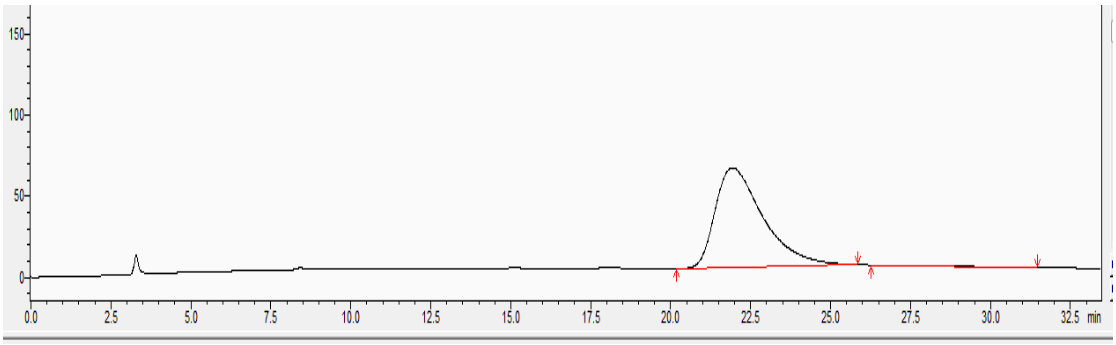

	Retention Time (min)	Area (%)
**Peak 1**	21.936	100
**Peak 2**	26.396	

Supplementary Figure 70 HPLC spectra for product **2l**

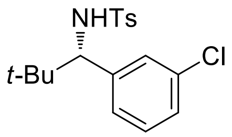

(*S*)-*N*-(1-(3-Chlorophenyl)-2,2-dimethylpropyl)-4-methylbenzenesulfonamide (2m)

99.9% ee, enantiomeric excess was determined by HPLC, DAICEL Chiralpak OJ column, Hexane/*i*-PrOH = 97/3, 220 nm, 0.5 mL/min.

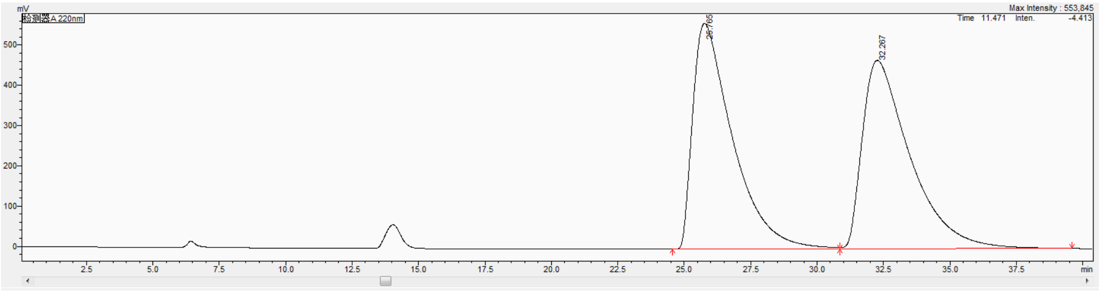

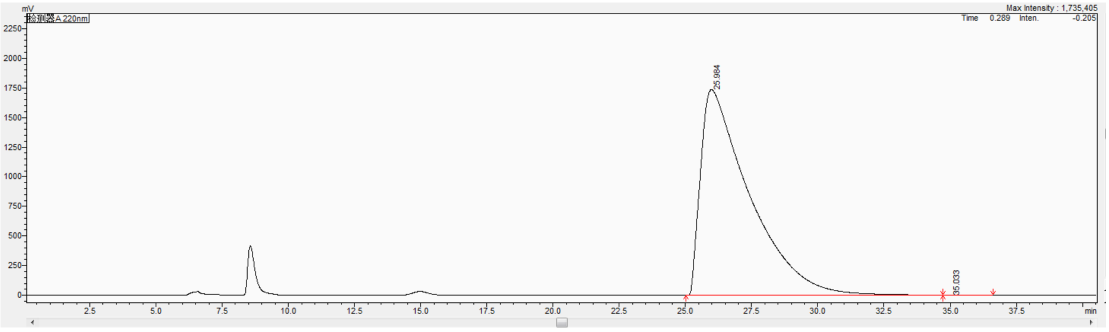

	Retention Time (min)	Area (%)
**Peak 1**	25.984	99.988
**Peak 2**	35.033	0.012

Supplementary Figure 71 HPLC spectra for product **2m**

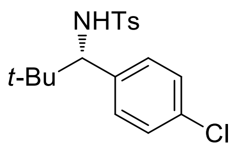

(*S*)-*N*-(1-(4-Chlorophenyl)-2,2-dimethylpropyl)-4-methylbenzenesulfonamide (2n)

99.9% ee, enantiomeric excess was determined by HPLC, DAICEL Chiralpak OJ column, Hexane/*i*-PrOH = 95/5, 220 nm, 1.0 mL/min.

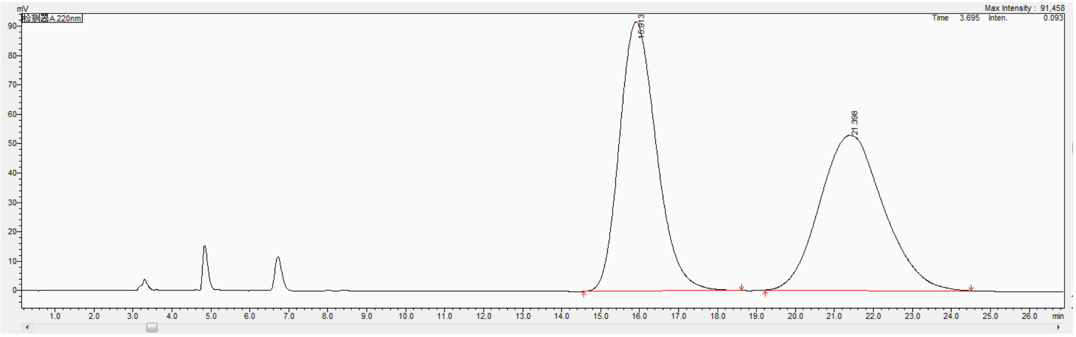

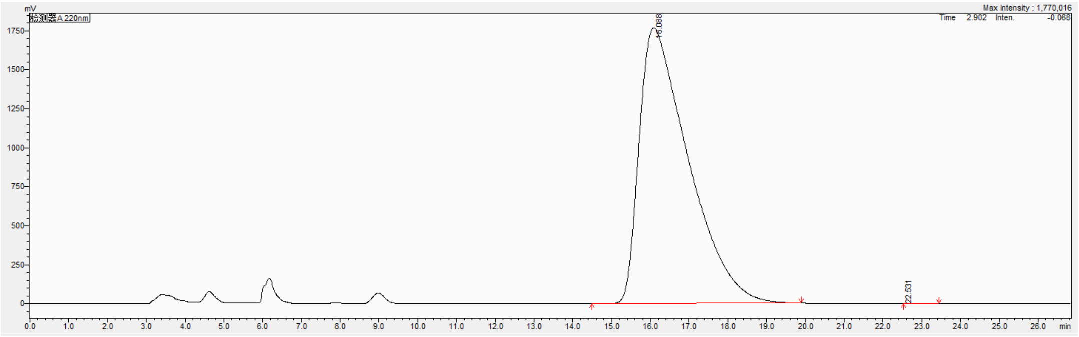

	Retention Time (min)	Area (%)
**Peak 1**	16.088	99.999
**Peak 2**	22.531	0.001

Supplementary Figure 72 HPLC spectra for product **2n**

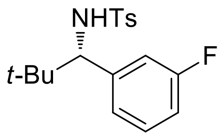

(*S*)-*N*-(1-(3-Fluorophenyl)-2,2-dimethylpropyl)-4-methylbenzenesulfonamide (2o)

99.9% ee, enantiomeric excess was determined by HPLC, DAICEL Chiralpak OJ column, Hexane/*i*-PrOH = 70/30, 220 nm, 1.0 mL/min.

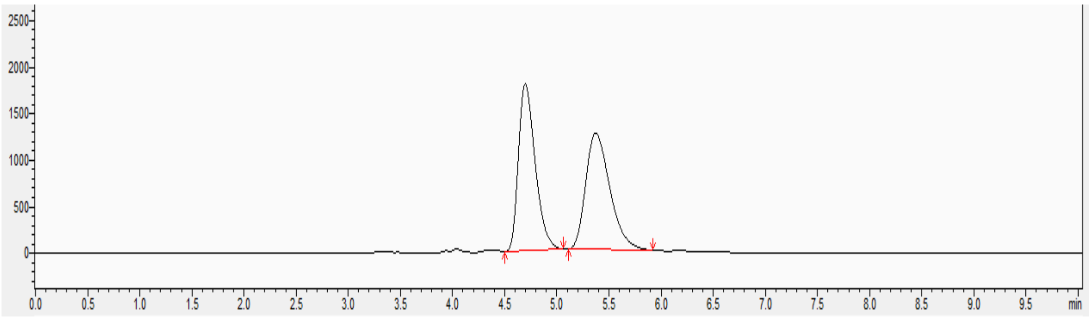

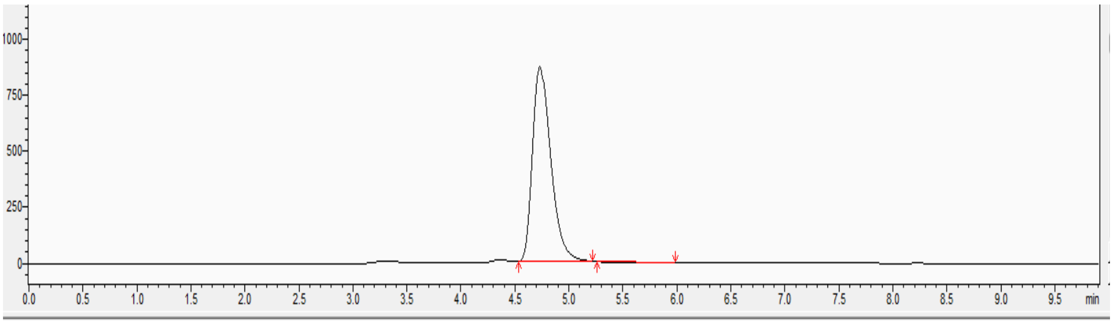

	Retention Time (min)	Area (%)
**Peak 1**	4.734	100
**Peak 2**	5.454	

Supplementary Figure 73 HPLC spectra for product **2o**

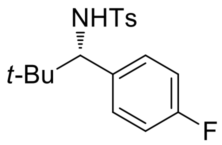

(*S*)-*N*-(2,2-Dimethyl-1-(p-tolyl)propyl)-4-methylbenzenesulfonamide (2p)

99.9% ee, entiomeric excess was determined by HPLC, DAICEL Chiralpak OJ column, Hexane/*i*-PrOH = 70/30, 220 nm, 1.0 mL/min.

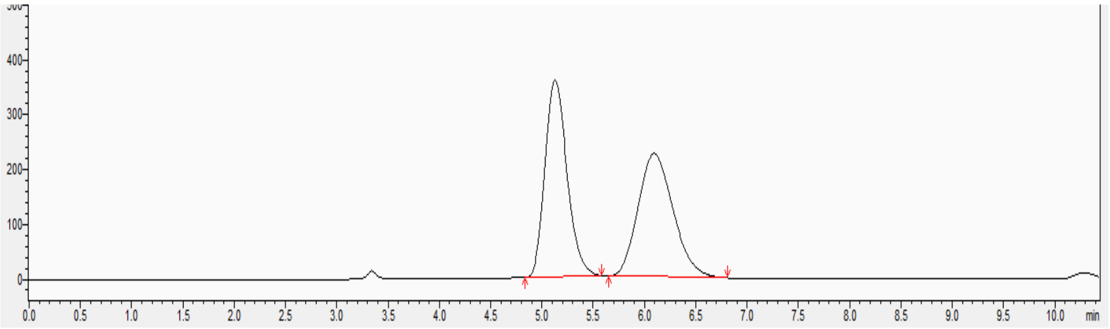

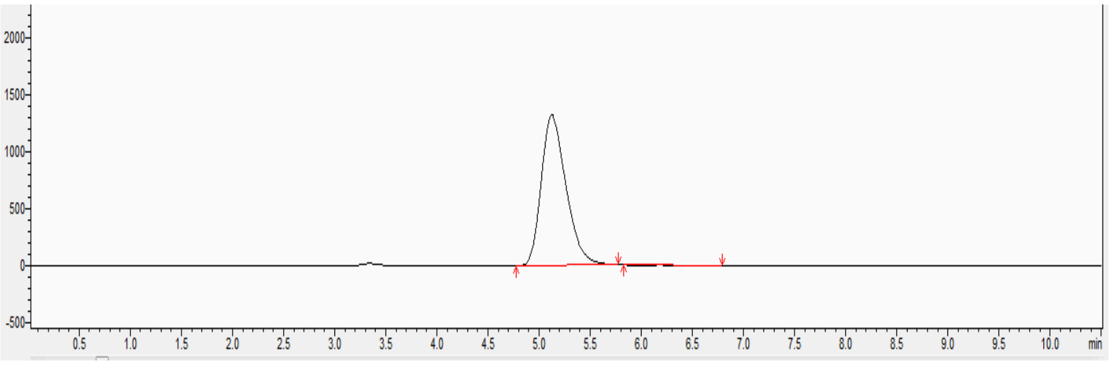

	Retention Time (min)	Area (%)
**Peak 1**	5.124	99.963
**Peak 2**	6.179	0.037

Supplementary Figure 74 HPLC spectra for product **2p**

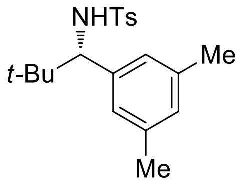

(*S*)-*N*-(1-(3,5-Dimethylphenyl)-2,2-dimethylpropyl)-4-methylbenzenesulfonamide. (2q)

99.9% ee, enantiomeric excess was determined by HPLC, DAICEL Chiralpak OD column, Hexane/*i*-PrOH = 93/7, 220 nm, 1.0 mL/min.

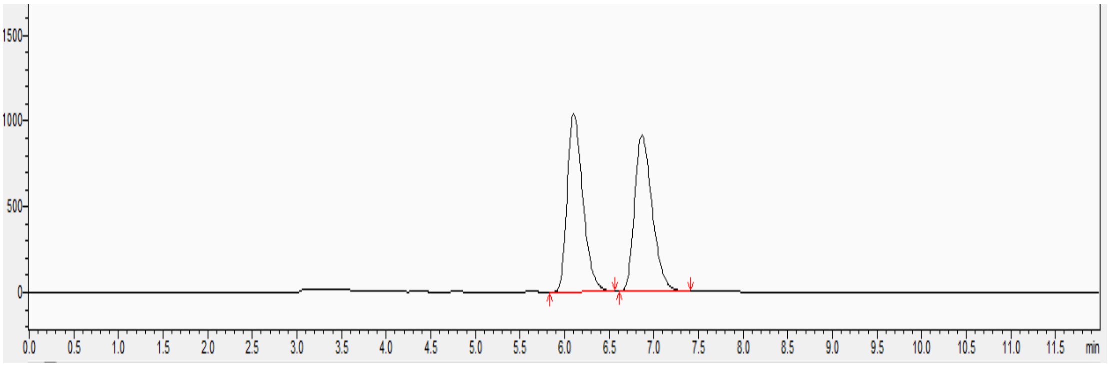

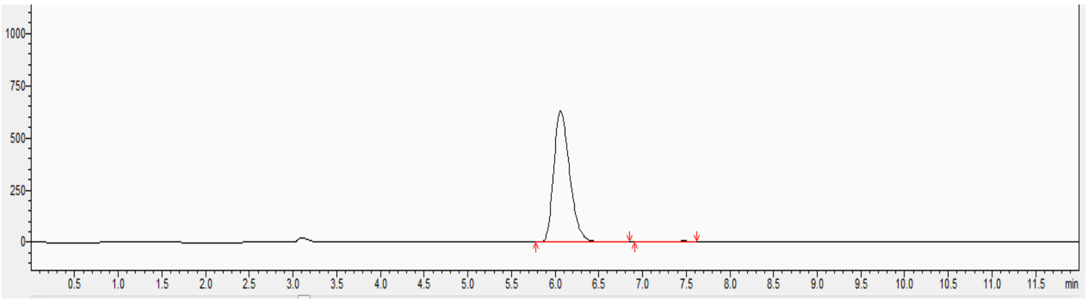

	Retention Time (min)	Area (%)
**Peak 1**	5.597	99.853
**Peak 2**	6.062	0.147

Supplementary Figure 75 HPLC spectra for product **2q**

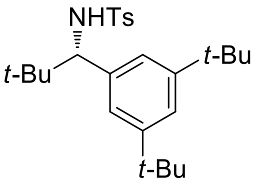

(*S*)-*N*-(1-(3,5-Di-tert-butylphenyl)-2,2-dimethylpropyl)-4-methylbenzenesulfonamide (2r)

99.9% ee, enantiomeric excess was determined by HPLC, DAICEL Chiralpak IC column, Hexane/*i*-PrOH = 95/5, 220 nm, 1.0 mL/min.

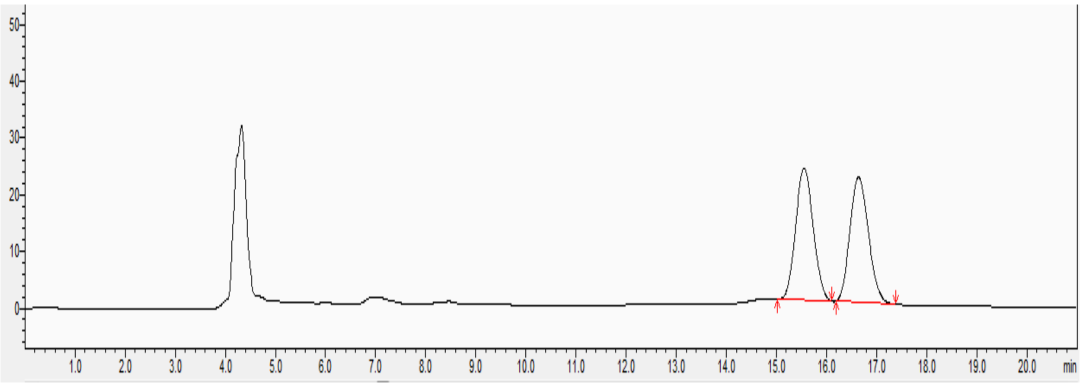

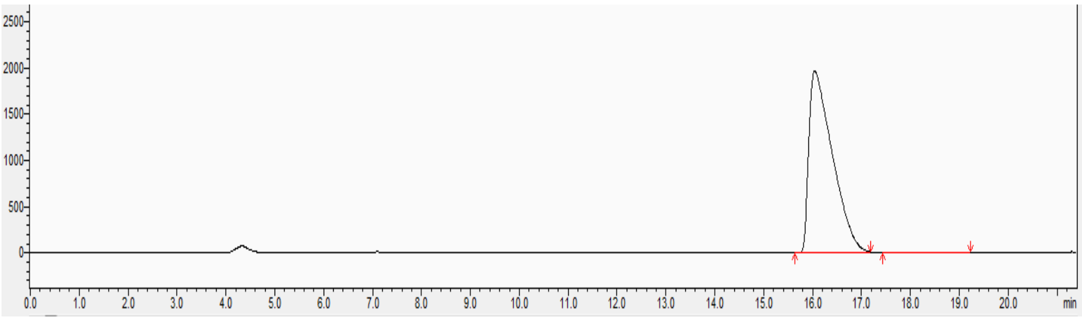

	Retention Time (min)	Area (%)
**Peak 1**	15.433	99.994
**Peak 2**	16.043	0.006

Supplementary Figure 76 HPLC spectra for product **2r**

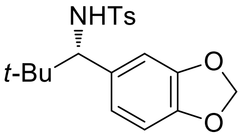

(*S*)-*N*-(1-(Benzo[d][1,3]dioxol-5-yl)-2,2-dimethylpropyl)-4-methylbenzenesulfonamide (2s)

99.9% ee, enantiomeric excess was determined by HPLC, DAICEL Chiralpak OJ column, Hexane/*i*-PrOH = 95/5, 220 nm, 1.0 mL/min.

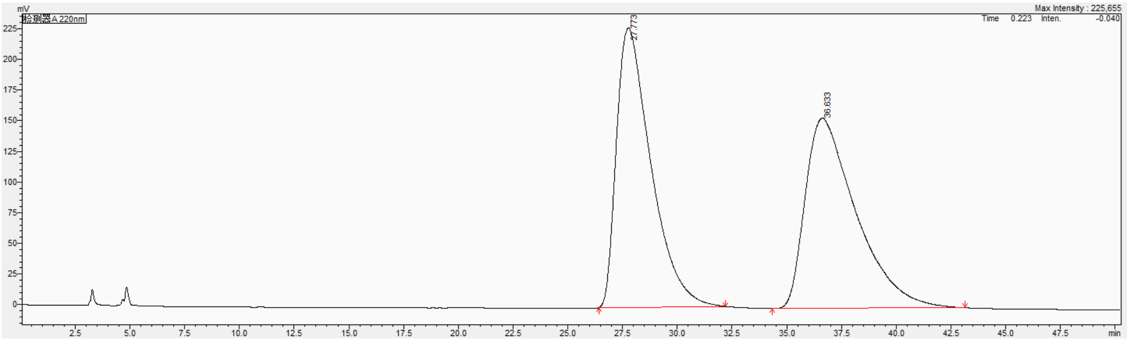

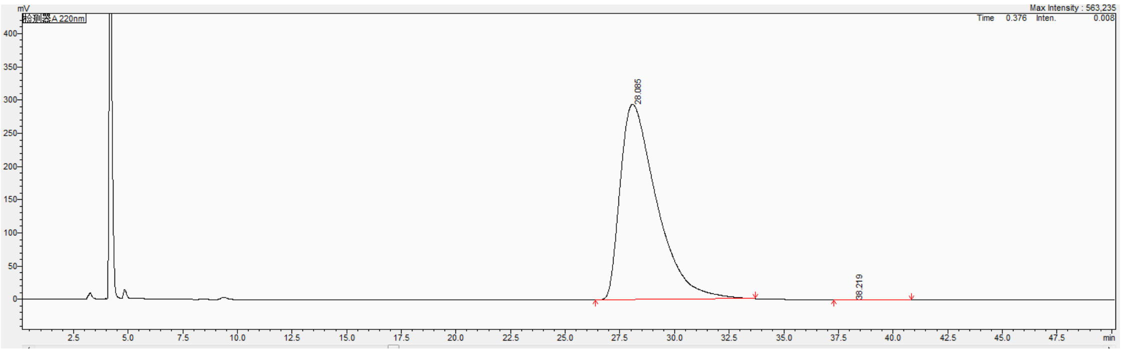

	Retention Time (min)	Area (%)
**Peak 1**	28.085	99.942
**Peak 2**	38.219	0.058

Supplementary Figure 77 HPLC spectra for product **2s**

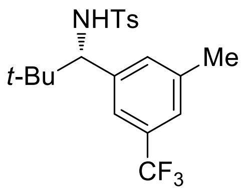

(*S*)-*N*-(2,2-Dimethyl-1-(3-methyl-5-(trifluoromethyl)phenyl)propyl)-4-methylbenzenesulfonamide (2t)

99.9% ee, enantiomeric excess was determined by HPLC, DAICEL Chiralpak IC-3 column, Hexane/*i*-PrOH = 95/5, 220 nm, 1.0 mL/min.

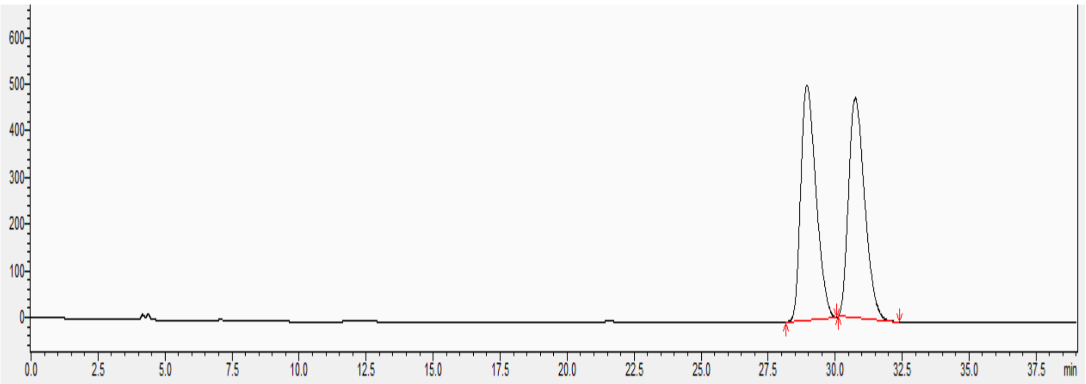

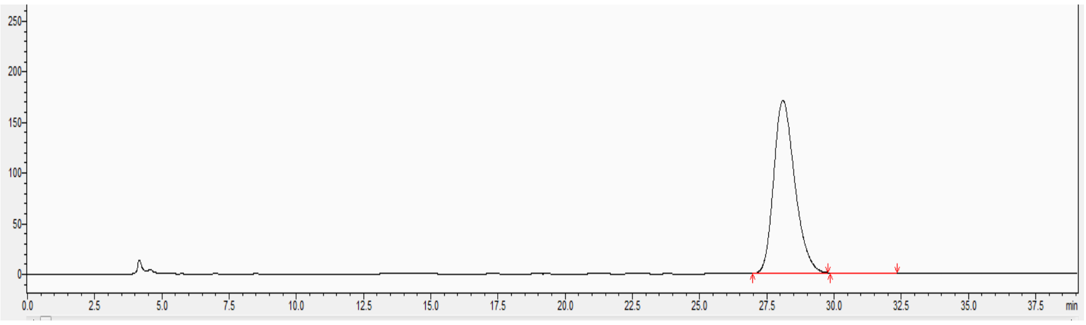

	Retention Time (min)	Area (%)
**Peak 1**	28.098	100
**Peak 2**	30.755	

Supplementary Figure 78 HPLC spectra for product **2t**

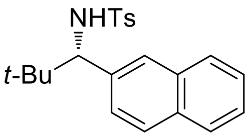

(*S*)-*N*-(2,2-Dimethyl-1-(naphthalen-2-yl)propyl)-4-methylbenzenesulfonamide (2u)

99.4% ee, enantiomeric excess was determined by HPLC, DAICEL ChiralpakOD column, Hexane/*i*-PrOH = 96/4, 220 nm, 1.0 mL/min.

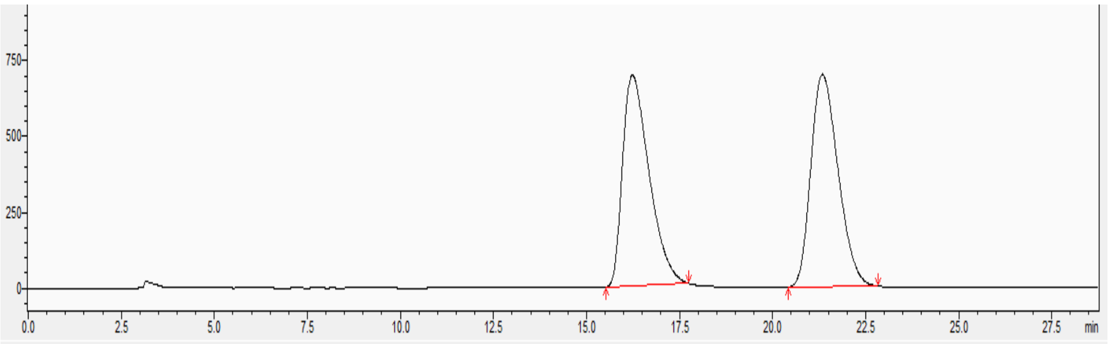

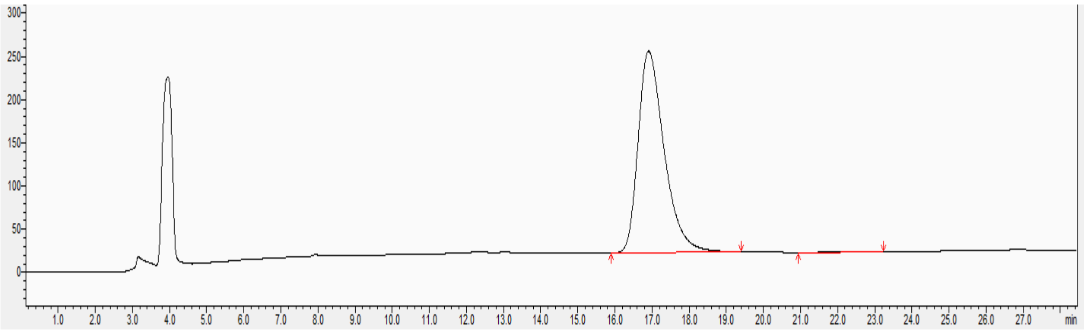

	Retention Time (min)	Area (%)
**Peak 1**	16.910	99.706
**Peak 2**	22.082	0.294

Supplementary Figure 79 HPLC spectra for product **2u**

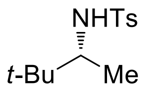

(*R*)-*N*-(3,3-Dimethylbutan-2-yl)-4-methylbenzenesulfonamide (2v)

99.9% ee, enantiomeric excess was determined by HPLC, DAICEL Chiralpak OJ column, Hexane/*i*-PrOH = 99/1, 220 nm, 1.0 mL/min.

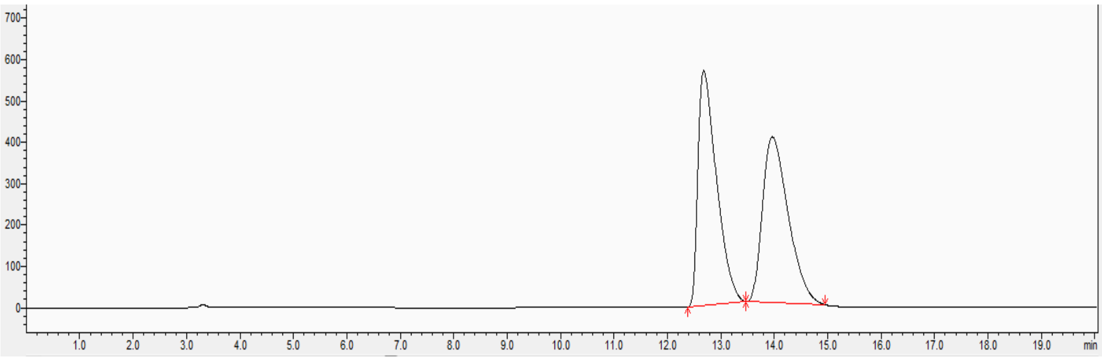

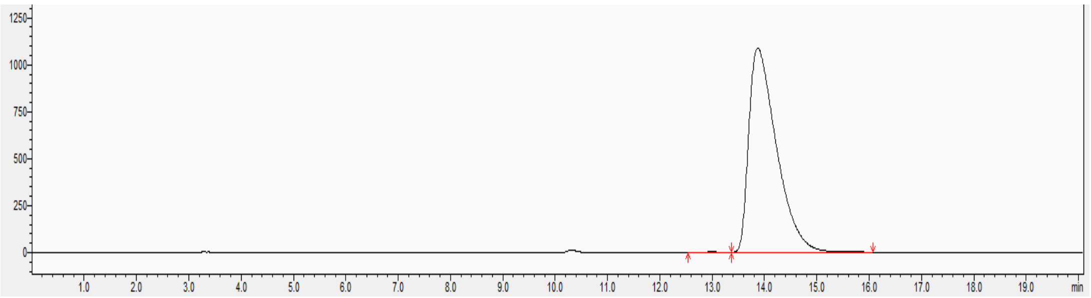

	Retention Time (min)	Area (%)
**Peak 1**	13.000	0.006
**Peak 2**	13.873	99.994

Supplementary Figure 80 HPLC spectra for product **2v**

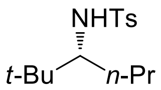

(*R*)-*N*-(2,2-Dimethylhexan-3-yl)-4-methylbenzenesulfonamide (2w)

99.8% ee, enantiomeric excess was determined by HPLC, DAICEL Chiralpak OJ column, Hexane/*i*-PrOH = 99/1, 220 nm, 1.0 mL/min.

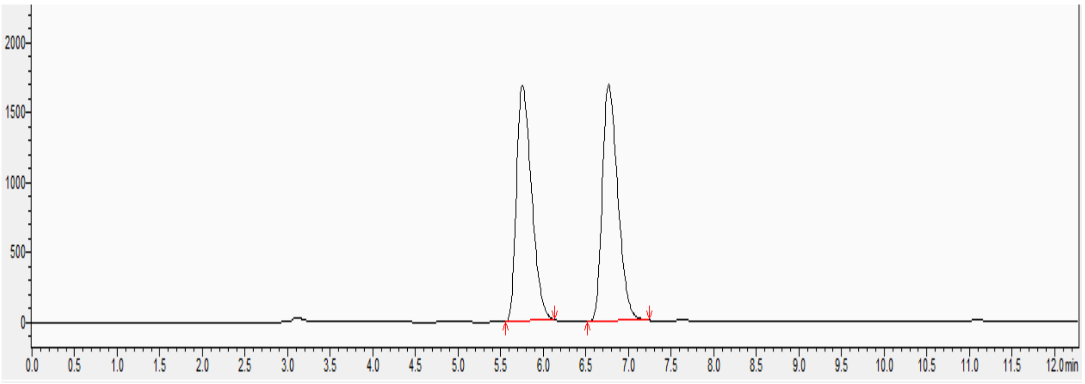

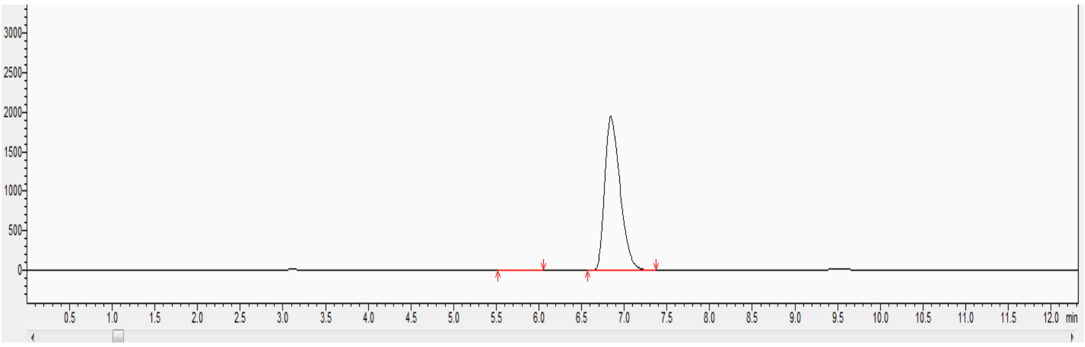

	Retention Time (min)	Area (%)
**Peak 1**	6.451	0.037
**Peak 2**	6.843	99.963

Supplementary Figure 81 HPLC spectra for product **2w**

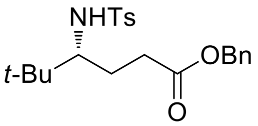

(*R*)-Benzyl 5,5-dimethyl-4-(4-methylphenylsulfonamido)hexanoate (2x)

99.7% ee, enantiomeric excess was determined by HPLC, DAICEL Chiralpak IE column, Hexane/*i*-PrOH = 90/10, 220 nm, 0.5 mL/min.

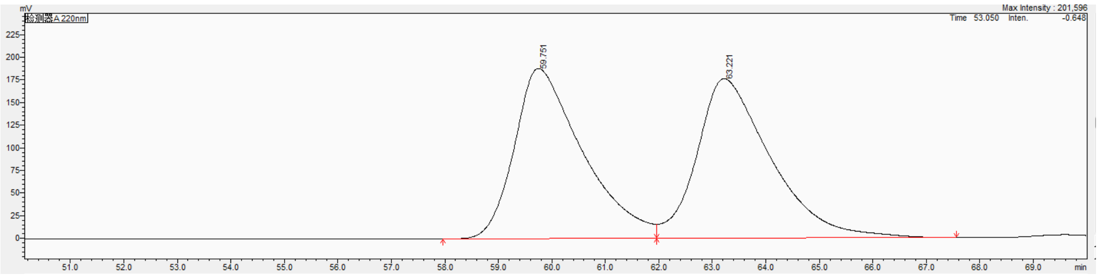

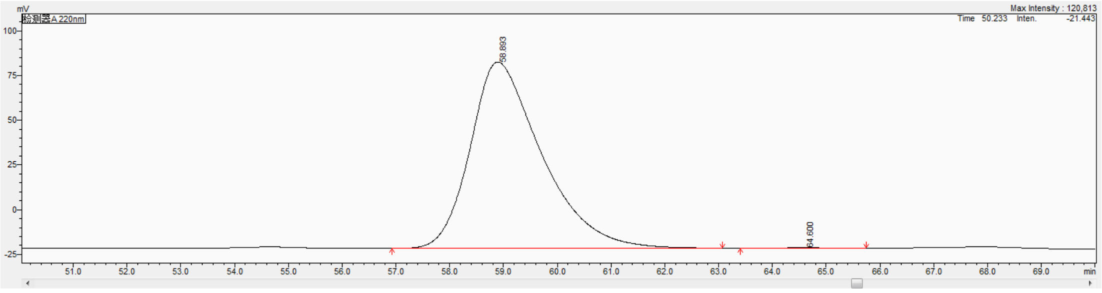

	Retention Time (min)	Area (%)
**Peak 1**	58.893	99.839
**Peak 2**	64.600	0.161

Supplementary Figure 82 HPLC spectra for product **2x**

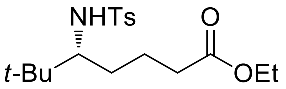

(*R*)-Ethyl 6,6-dimethyl-5-(4-methylphenylsulfonamido)heptanoate (2y)

99.4% ee, enantiomeric excess was determined by HPLC, DAICEL Chiralpak OJ column, Hexane/*i*-PrOH = 92/8, 220 nm, 0.5 mL/min.

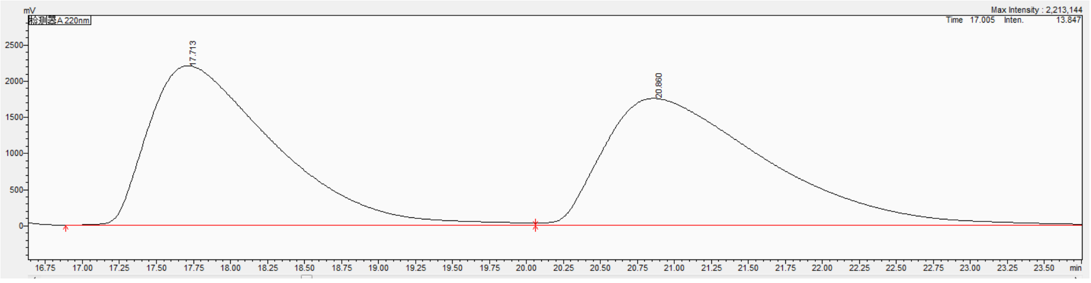

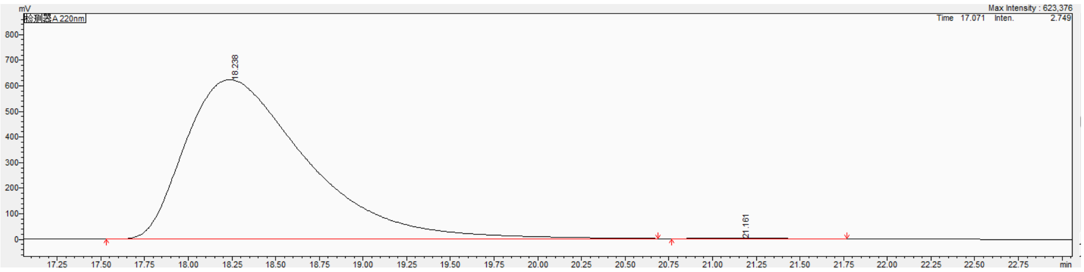

	Retention Time (min)	Area (%)
**Peak 1**	18.238	99.706
**Peak 2**	21.161	0.294

Supplementary Figure 83 HPLC spectra for product **2y**

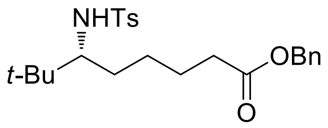

(*R*)-Benzyl 7,7-dimethyl-6-(4-methylphenylsulfonamido)octanoate (2z)

99.1% ee, enantiomeric excess was determined by HPLC, DAICEL Chiralpak IC-3 column, Hexane/*i*-PrOH = 90/10, 220 nm, 0.8 mL/min.

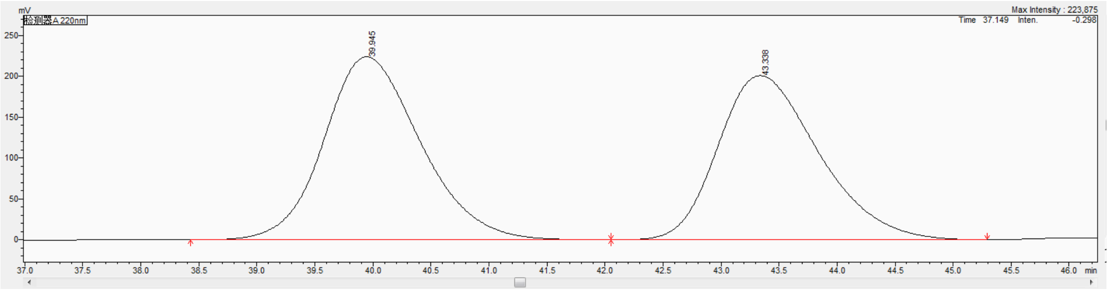

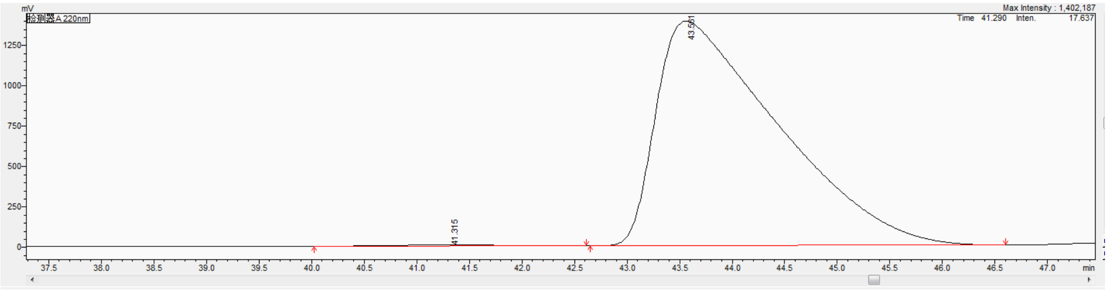

	Retention Time (min)	Area (%)
**Peak 1**	41.315	0.446
**Peak 2**	43.561	99.554

Supplementary Figure 84 HPLC spectra for product **2z**

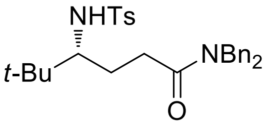

(*R*)-*N*,*N*-Dibenzyl-5,5-dimethyl-4-(4-methylphenylsulfonamido)hexanamide (2aa)

99.4% ee, enantiomeric excess was determined by HPLC, DAICEL Chiralpak IE column, Hexane/*i*-PrOH = 85/15, 230 nm, 0.8 mL/min.

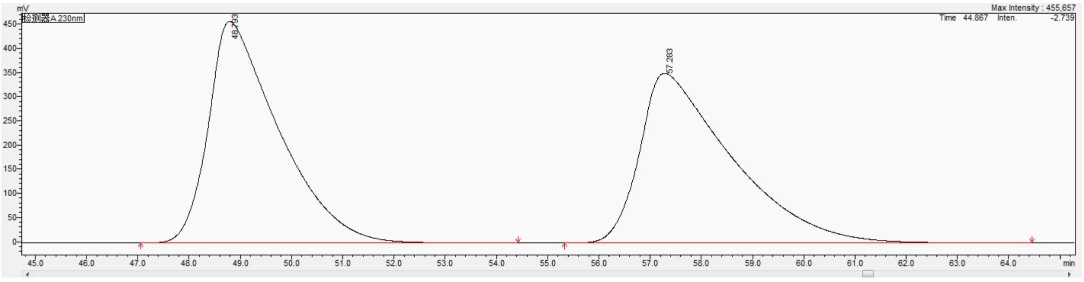

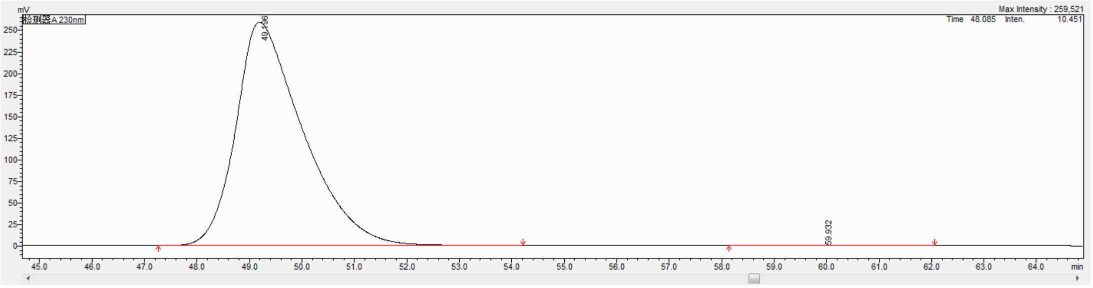

	Retention Time (min)	Area (%)
**Peak 1**	49.196	99.684
**Peak 2**	59.932	0.316

Supplementary Figure 85 HPLC spectra for product **2aa**

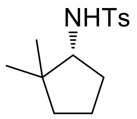

(*R*)-*N*-(2,2-Dimethylcyclopentyl)-4-methylbenzenesulfonamide (2ab)

99.3% ee, enantiomeric excess was determined by HPLC, DAICEL Chiralpak AS column, Hexane/*i*-PrOH = 80/20, 220 nm, 0.6 mL/min.

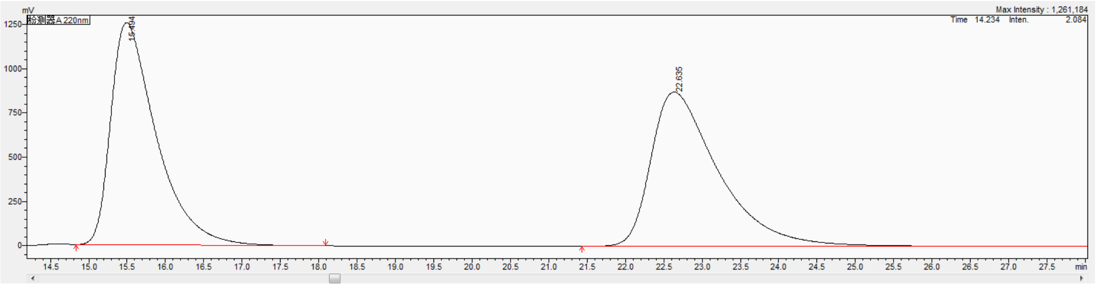

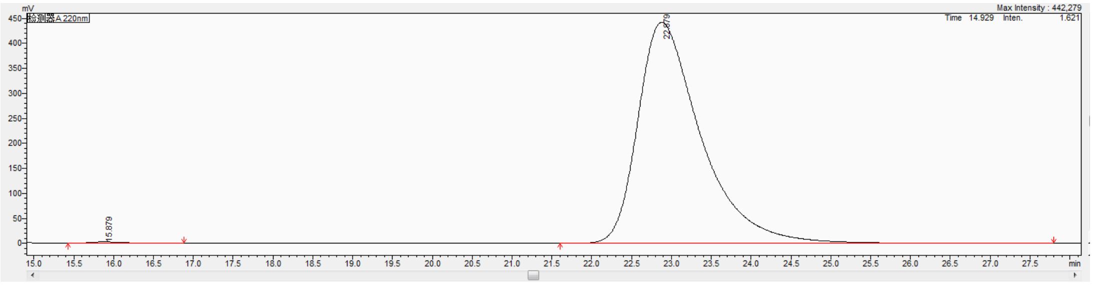

	Retention Time (min)	Area (%)
**Peak 1**	15.879	0.357
**Peak 2**	22.879	99.643

Supplementary Figure 86 HPLC spectra for product **2ab**

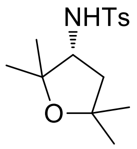

(*R*)-4-Methyl-N-(2,2,5,5-tetramethyltetrahydrofuran-3-yl)benzenesulfonamide (2ac)

99.8% ee, enantiomeric excess was determined by HPLC, DAICEL Chiralpak AS column, Hexane/*i*-PrOH = 80/20, 220 nm, 0.7 mL/min.

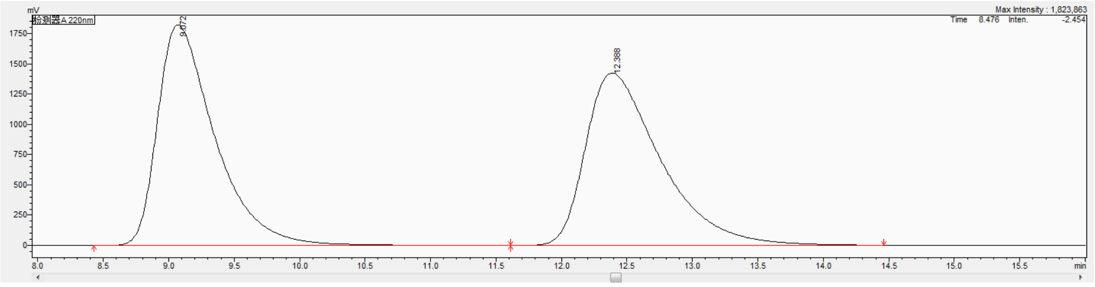

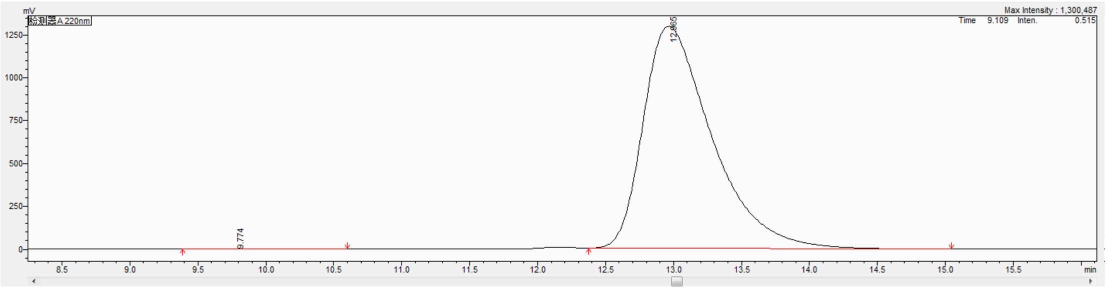

	Retention Time (min)	Area (%)
**Peak 1**	9.774	0.096
**Peak 2**	12.965	99.904

Supplementary Figure 87 HPLC spectra for product **2ac**

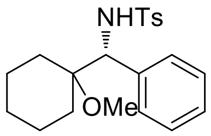

(*R*)-*N*-((1-Methoxycyclohexyl)(phenyl)methyl)-4-methylbenzenesulfonamide (2ad)

99.4% ee, enantiomeric excess was determined by HPLC, DAICEL Chiralpak AS column, Hexane/*i*-PrOH = 85/15, 220 nm, 0.8 mL/min.

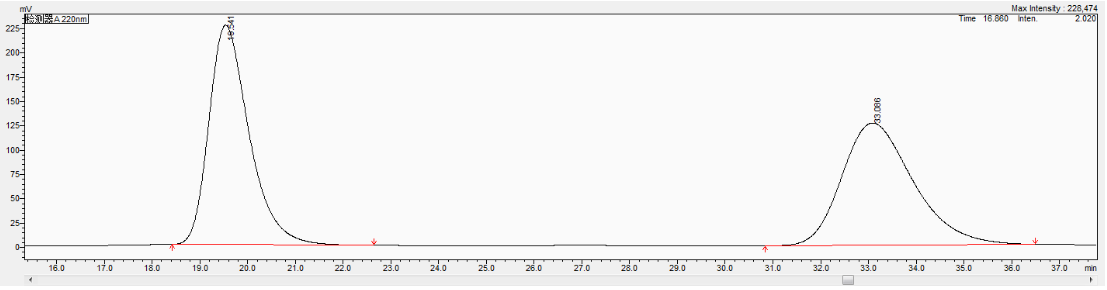

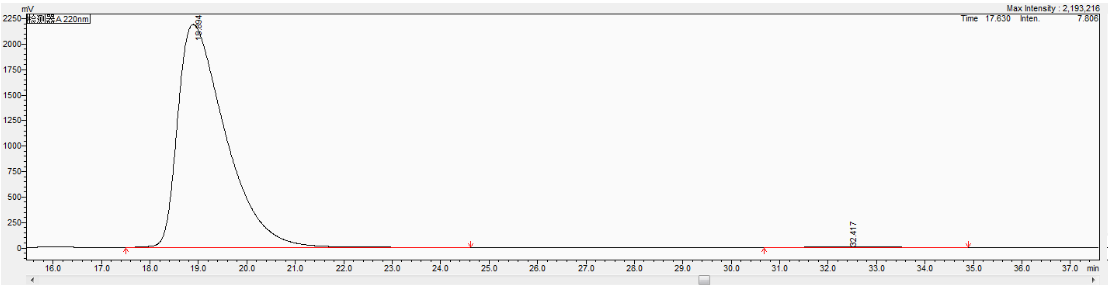

	Retention Time (min)	Area (%)
**Peak 1**	18.894	99.681
**Peak 2**	32.417	0.319

Supplementary Figure 88 HPLC spectra for product **2ad**

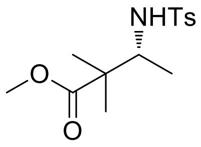

(*R*)-Methyl 2,2-dimethyl-3-(4-methylphenylsulfonamido)butanoate (2ae)

96.9% ee, enantiomeric excess was determined by HPLC, DAICEL Chiralpak OJ column, Hexane/*i*-PrOH = 85/15, 220 nm, 0.6 mL/min.

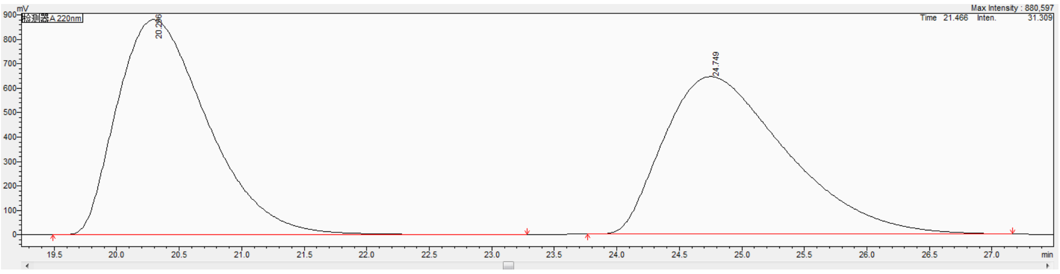

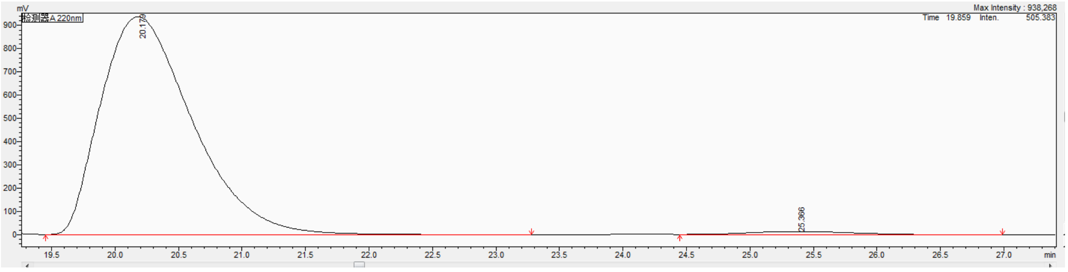

	Retention Time (min)	Area (%)
**Peak 1**	20.179	98.407
**Peak 2**	25.366	1.593

Supplementary Figure 89 HPLC spectra for product **2ae**

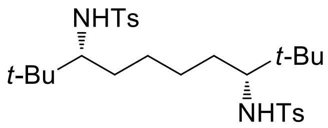

***N*,*N'*-((3*R*,8*R*)-2,2,9,9-Tetramethyldecane-3,8-diyl)bis(4-methylbenzenesulfonamide) (2af)**

99% de, 99.9% ee, enantiomeric excess was determined by HPLC, DAICEL Chiralpak IC-3 column, Hexane/*i*-PrOH = 90/10, 220 nm, 1.0 mL/min.

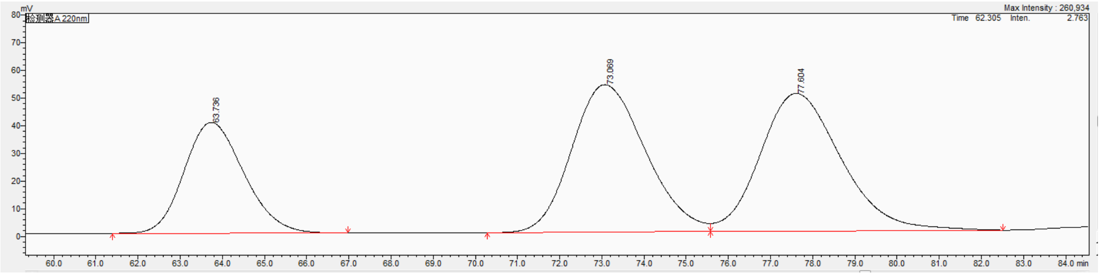

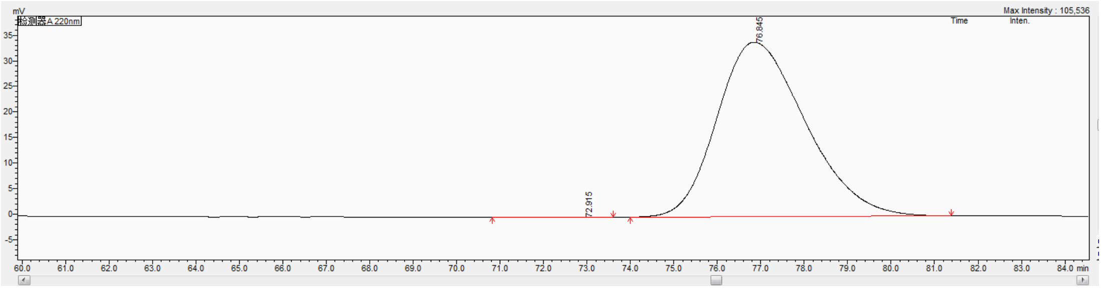

	Retention Time (min)	Area (%)
**meso**	63.738	0
**Peak 1**	72.915	0.069
**Peak 2**	76.845	99.931

Supplementary Figure 90 HPLC spectra for product **2af**

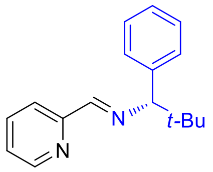

(*S*)-2,2-Dimethyl-1-phenyl-N-(pyridin-2-ylmethylene)propan-1-amine(5)

99.3% ee, enantiomeric excess was determined by HPLC, DAICEL Chiralpak OD column, Hexane/*i*-PrOH = 95/5, 230 nm, 0.5 mL/min.

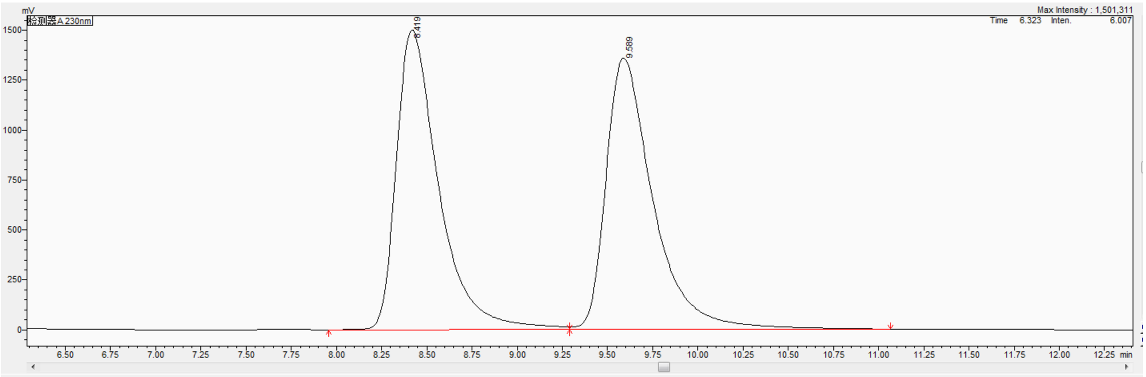

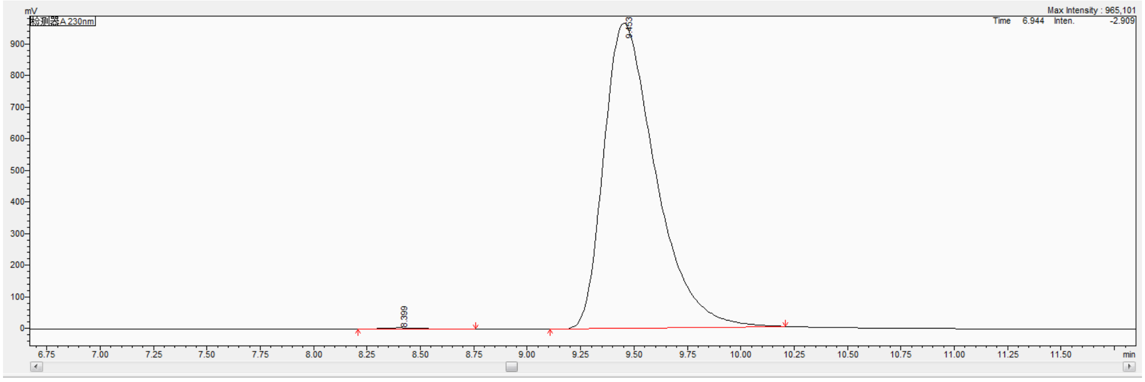

	Retention Time (min)	Area (%)
**Peak 1**	8.399	0.341
**Peak 2**	9.453	99.659

Supplementary Figure 91 HPLC spectra for product **5**

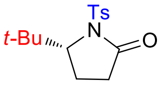

(*R*)-5-(*Tert*-butyl)-1-tosylpyrrolidin-2-one (7x)

99.9% ee, enantiomeric excess was determined by HPLC, DAICEL Chiralpak AS column, Hexane/*i*-PrOH = 85/15, 220 nm, 1.0 mL/min.

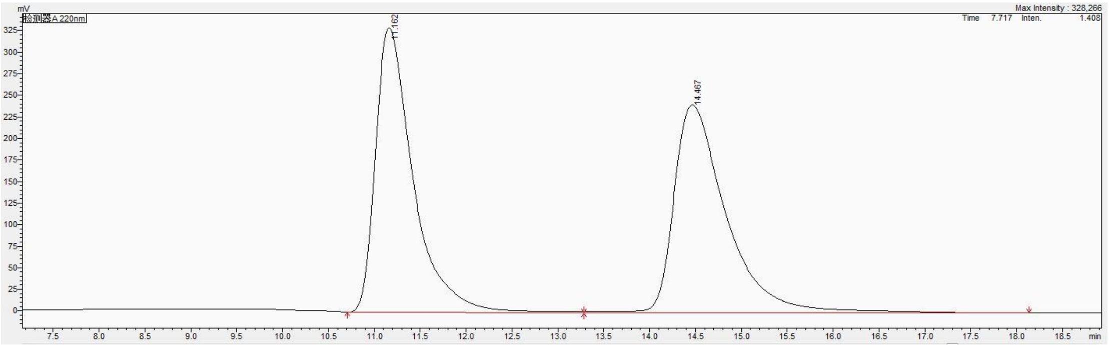

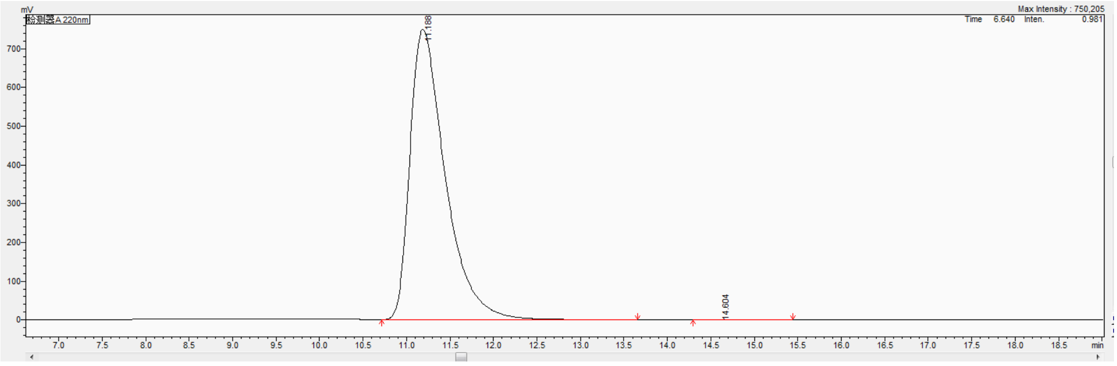

	Retention Time (min)	Area (%)
Peak 1	11.188	99.991
Peak 2	14.604	0.009

Supplementary Figure 92 HPLC spectra for product **7x**

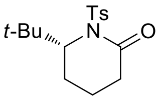

(*R*)-6-(*Tert*-butyl)-1-tosylpiperidin-2-one (7y)

99.9% ee, enantiomeric excess was determined by HPLC, DAICEL Chiralpak OJ column, Hexane/*i*-PrOH = 80/20, 220 nm, 1.0 mL/min.

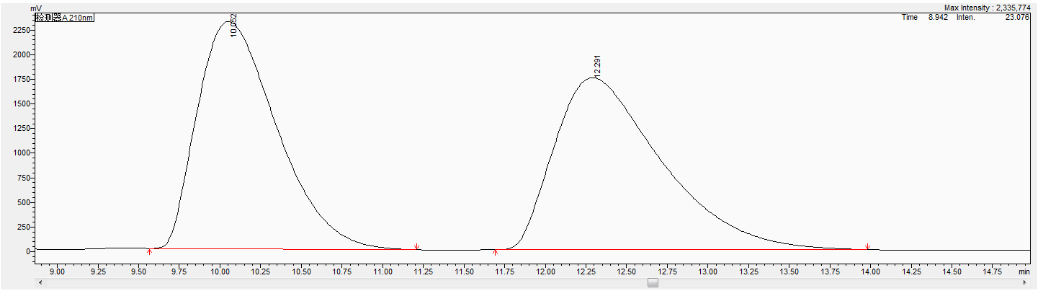

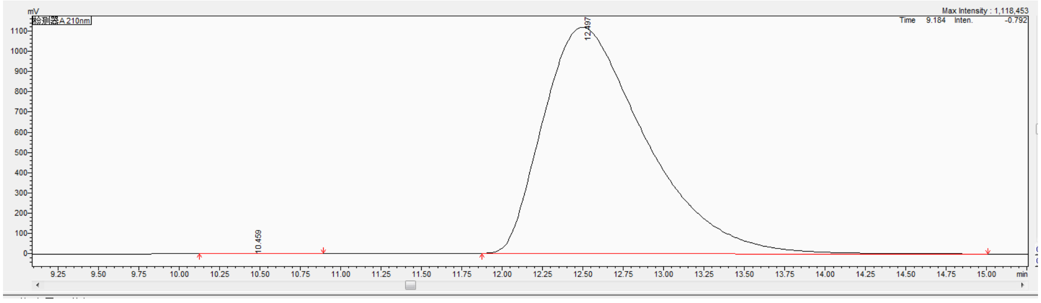

	Retention Time (min)	Area (%)
**Peak 1**	10.459	0.069
**Peak 2**	12.497	99.931

Supplementary Figure 93 HPLC spectra for product **7y**

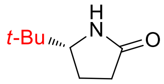

(*R*)-5-(*tert*-butyl)pyrrolidin-2-one(8x)

97% ee, enantiomeric excess was determined by HPLC, DAICEL Chiralpak IC czolumn, Hexane/*i*-PrOH = 80/20, 210 nm, 0.8 mL/min.

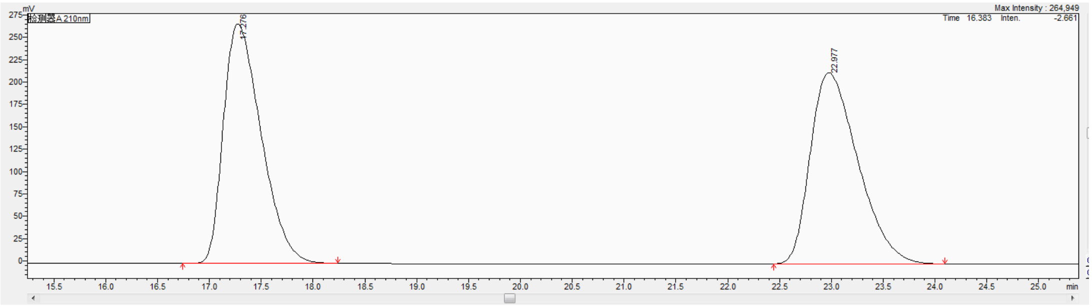

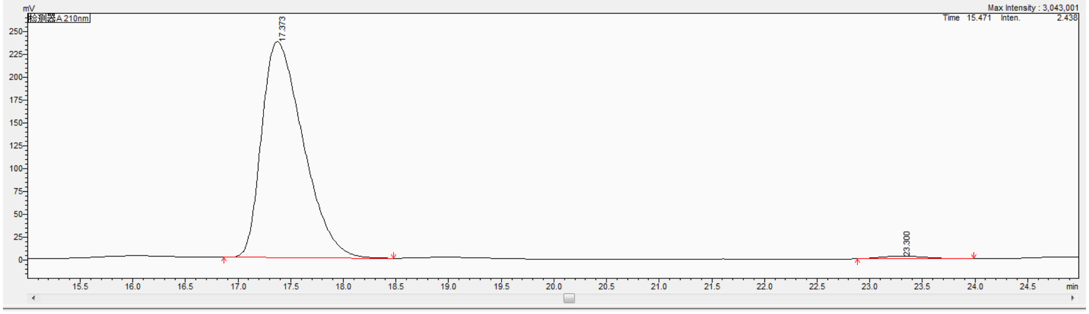

	Retention Time (min)	Area (%)
**Peak 1**	17.373	98.677
**Peak 2**	23.300	1.323

Supplementary Figure 94 HPLC spectra for product **8x**

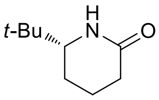

(*R*)-6-(*tert*-butyl)piperidin-2-one (8y)

99% ee, entiomeric excess was determined by HPLC, DAICEL Chiralpak AS column, Hexane/*i*-PrOH = 85/15, 210 nm, 0.8 mL/min.

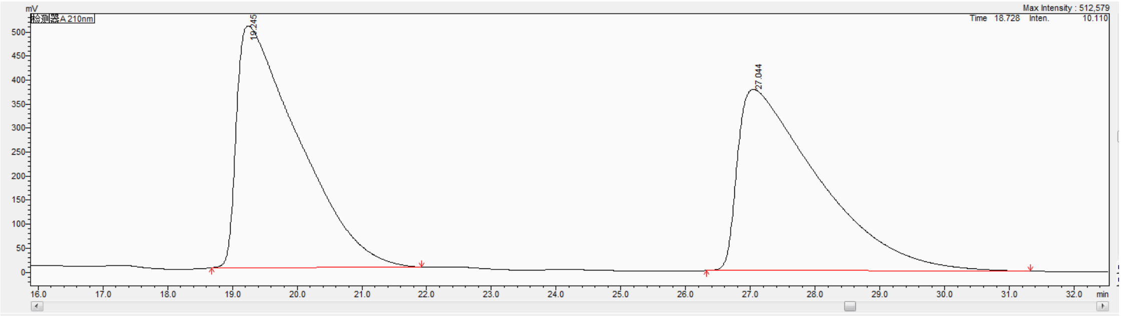

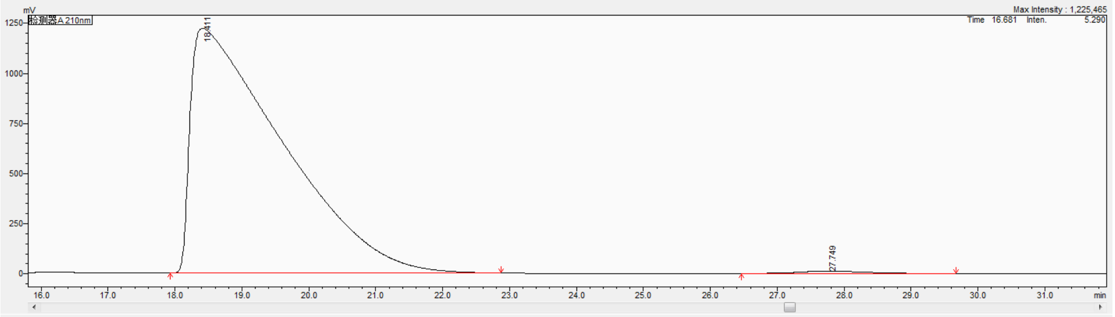

	Retention Time (min)	Area (%)
**Peak 1**	18.411	99.133
**Peak 2**	27.749	0.867

Supplementary Figure 95 HPLC spectra for product **8y**

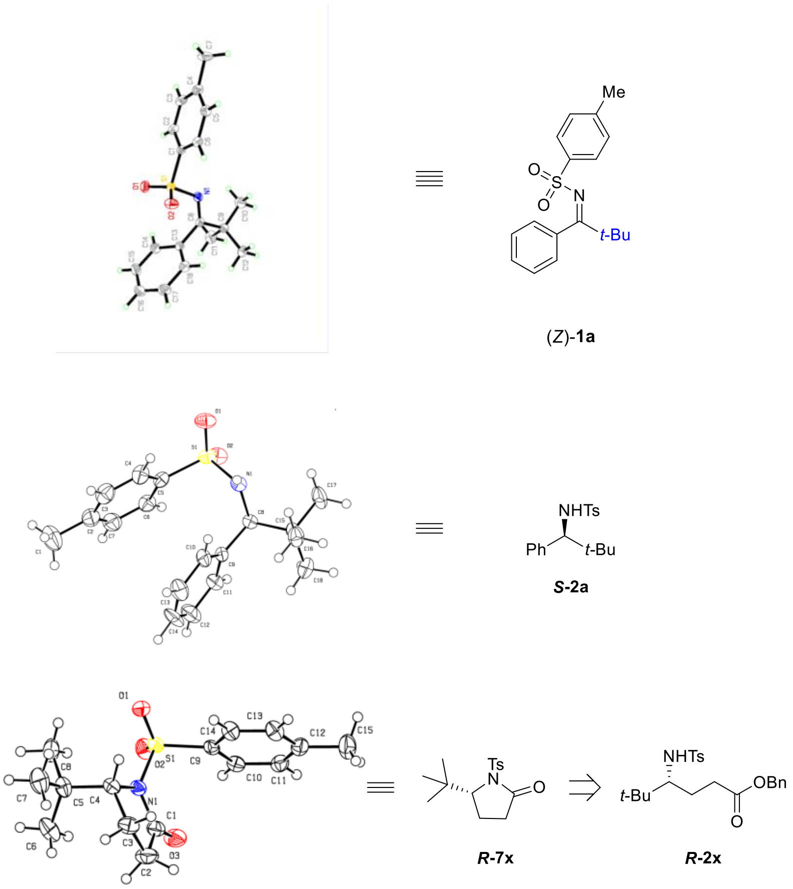

Supplementary Figure 96 ORTEP representation of **1a**, **2a** and **7x**

The absolute configurations of the substrate **1a**, the products **2a** and **7x** were determined to be *S* and *R* by X-ray crystallographic analysis (**1a:** 1875390, **2a**: CCDC 1585399, **7x**: CCDC 1585398). Therefore, substrates with aryl or alkyl groups (including functionalized compounds) are attacked by the hydride on the same favored side.

Supplementary Table 2 Bond lengths [A] and angles [deg] for **1a**.

S(1)-O(2)	1.429(2)
S(1)-O(1)	1.434(2)
S(1)-N(1)	1.678(2)
S(1)-C(1)	1.767(3)
N(1)-C(8)	1.272(4)
C(8)-C(13)	1.507(4)
C(8)-C(9)	1.539(4)
C(6)-C(1)	1.383(4)
C(6)-C(5)	1.384(4)
C(6)-H(6A)	0.9500
C(13)-C(18)	1.390(4)

C(13)-C(14)	1.392(4)
C(1)-C(2)	1.386(4)
C(2)-C(3)	1.384(5)
C(2)-H(2B)	0.9500
C(9)-C(10)	1.526(5)
C(9)-C(12)	1.533(5)
C(9)-C(11)	1.539(5)
C(5)-C(4)	1.388(5)
C(5)-H(5A)	0.9500
C(3)-C(4)	1.393(5)
C(3)-H(3A)	0.9500
C(4)-C(7)	1.495(5)
C(7)-H(7A)	0.9800
C(7)-H(7B)	0.9800
C(7)-H(7C)	0.9800
C(16)-C(17)	1.384(6)
C(16)-C(15)	1.384(6)
C(16)-H(16A)	0.9500
C(18)-C(17)	1.383(5)
C(18)-H(18A)	0.9500
C(14)-C(15)	1.374(5)
C(14)-H(14A)	0.9500
C(17)-H(17A)	0.9500
C(15)-H(15A)	0.9500
C(12)-H(12A)	0.9800
C(12)-H(12B)	0.9800
C(12)-H(12C)	0.9800
C(11)-H(11A)	0.9800
C(11)-H(11B)	0.9800
C(11)-H(11C)	0.9800
C(10)-H(10A)	0.9800
C(10)-H(10B)	0.9800
C(10)-H(10C)	0.9800
O(2)-S(1)-O(1)	119.29(15)
O(2)-S(1)-N(1)	110.49(13)
O(1)-S(1)-N(1)	108.60(13)
O(2)-S(1)-C(1)	108.30(13)
O(1)-S(1)-C(1)	108.18(13)
N(1)-S(1)-C(1)	100.28(12)
C(8)-N(1)-S(1)	120.9(2)
N(1)-C(8)-C(13)	124.4(3)
N(1)-C(8)-C(9)	117.3(3)
C(13)-C(8)-C(9)	118.2(2)

C(1)-C(6)-C(5)	118.6(3)
C(1)-C(6)-H(6A)	120.7
C(5)-C(6)-H(6A)	120.7
C(18)-C(13)-C(14)	119.4(3)
C(18)-C(13)-C(8)	120.7(3)
C(14)-C(13)-C(8)	119.9(3)
C(6)-C(1)-C(2)	121.8(3)
C(6)-C(1)-S(1)	118.7(2)
C(2)-C(1)-S(1)	119.4(2)
C(3)-C(2)-C(1)	118.3(3)
C(3)-C(2)-H(2B)	120.9
C(1)-C(2)-H(2B)	120.9
C(10)-C(9)-C(12)	110.0(3)
C(10)-C(9)-C(8)	110.9(3)
C(12)-C(9)-C(8)	108.8(3)
C(10)-C(9)-C(11)	109.4(3)
C(12)-C(9)-C(11)	109.4(3)
C(8)-C(9)-C(11)	108.3(3)
C(6)-C(5)-C(4)	121.3(3)
C(6)-C(5)-H(5A)	119.3
C(4)-C(5)-H(5A)	119.4
C(2)-C(3)-C(4)	121.4(3)
C(2)-C(3)-H(3A)	119.3
C(4)-C(3)-H(3A)	119.3
C(5)-C(4)-C(3)	118.5(3)
C(5)-C(4)-C(7)	122.6(3)
C(3)-C(4)-C(7)	118.9(3)
C(4)-C(7)-H(7A)	109.5
C(4)-C(7)-H(7B)	109.5
H(7A)-C(7)-H(7B)	109.5
C(4)-C(7)-H(7C)	109.5
H(7A)-C(7)-H(7C)	109.5
H(7B)-C(7)-H(7C)	109.5
C(17)-C(16)-C(15)	119.9(3)
C(17)-C(16)-H(16A)	120.0
C(15)-C(16)-H(16A)	120.0
C(17)-C(18)-C(13)	120.0(3)
C(17)-C(18)-H(18A)	120.0
C(13)-C(18)-H(18A)	120.0
C(15)-C(14)-C(13)	120.3(3)
C(15)-C(14)-H(14A)	119.9
C(13)-C(14)-H(14A)	119.9
C(18)-C(17)-C(16)	120.1(3)
C(18)-C(17)-H(17A)	119.9

C(16)-C(17)-H(17A)	119.9
C(14)-C(15)-C(16)	120.2(3)
C(14)-C(15)-H(15A)	119.9
C(16)-C(15)-H(15A)	119.9
C(9)-C(12)-H(12A)	109.5
C(9)-C(12)-H(12B)	109.5
H(12A)-C(12)-H(12B)	109.5
C(9)-C(12)-H(12C)	109.5
H(12A)-C(12)-H(12C)	109.5
H(12B)-C(12)-H(12C)	109.5
C(9)-C(11)-H(11A)	109.5
C(9)-C(11)-H(11B)	109.5
H(11A)-C(11)-H(11B)	109.5
C(9)-C(11)-H(11C)	109.5
H(11A)-C(11)-H(11C)	109.5
H(11B)-C(11)-H(11C)	109.5
C(9)-C(10)-H(10A)	109.5
C(9)-C(10)-H(10B)	109.5
H(10A)-C(10)-H(10B)	109.5
C(9)-C(10)-H(10C)	109.5
H(10A)-C(10)-H(10C)	109.5
H(10B)-C(10)-H(10C)	109.5

Supplementary Table 3 Bond lengths for **2a**.

Atom	Atom	Length/Å	Atom	Atom	Length/Å
S1	O1	1.432(3)	C11	C12	1.388(6)
S1	O2	1.445(3)	C15	C18	1.528(6)
S1	N1	1.622(3)	C15	C16	1.530(6)
S1	C5	1.768(4)	C15	C17	1.535(5)
N1	C8	1.473(5)	C2	C3	1.395(7)
C8	C9	1.525(5)	C2	C7	1.382(7)
C8	C15	1.566(5)	C2	C1	1.521(6)
C9	C11	1.381(6)	C4	C3	1.395(7)
C9	C10	1.393(6)	C13	C10	1.396(6)
C5	C6	1.378(6)	C13	C14	1.374(8)
C5	C4	1.384(6)	C12	C14	1.376(8)
C6	C7	1.380(7)			

Supplementary Table 4 Bond angles for **2a**.

Atom	Atom	Atom	Angle/˚	Atom	Atom	Atom	Angle/˚
O1	S1	O2	119.25(18)	C9	C11	C12	120.7(5)

O1	S1	N1	106.20(18)	C18	C15	C8	108.5(4)
O1	S1	C5	108.05(19)	C18	C15	C16	109.6(3)
O2	S1	N1	107.13(17)	C18	C15	C17	108.9(4)
O2	S1	C5	107.9(2)	C16	C15	C8	111.8(3)
N1	S1	C5	107.87(17)	C16	C15	C17	108.6(4)
C8	N1	S1	120.9(3)	C17	C15	C8	109.4(3)
N1	C8	C9	113.8(3)	C3	C2	C1	120.1(5)
N1	C8	C15	109.5(3)	C7	C2	C3	118.9(4)
C9	C8	C15	113.3(3)	C7	C2	C1	121.1(5)
C11	C9	C8	118.2(4)	C5	C4	C3	119.6(4)
C11	C9	C10	119.6(4)	C14	C13	C10	120.5(5)
C10	C9	C8	122.1(3)	C2	C3	C4	120.2(5)
C6	C5	S1	121.5(3)	C9	C10	C13	119.2(5)
C6	C5	C4	120.4(4)	C6	C7	C2	121.2(5)
C4	C5	S1	118.0(3)	C14	C12	C11	119.6(5)
C5	C6	C7	119.8(4)	C13	C14	C12	120.4(4)

Supplementary Table 5 Bond lengths for **7x**.

Atom	Atom	Length/Å	Atom	Atom	Length/Å
S2	O4	1.429(3)	C27	C30	1.507(6)
S2	O5	1.425(3)	C23	C20	1.518(6)
S2	N2	1.656(3)	C10	C11	1.383(6)
S2	C24	1.767(4)	C4	C5	1.554(6)
S1	O1	1.424(3)	C4	C3	1.535(6)
S1	O2	1.425(3)	C5	C8	1.523(7)
S1	N1	1.670(3)	C5	C7	1.532(6)
S1	C9	1.763(4)	C5	C6	1.534(7)
O6	C16	1.201(5)	C16	C17	1.487(7)
O3	C1	1.209(5)	C11	C12	1.392(6)
N2	C16	1.416(5)	C14	C13	1.385(6)
N2	C19	1.501(5)	C20	C19	1.555(6)
N1	C4	1.492(5)	C20	C22	1.536(6)
N1	C1	1.408(5)	C20	C21	1.535(6)
C24	C25	1.382(6)	C28	C29	1.380(6)
C24	C29	1.391(6)	C3	C2	1.530(6)
C26	C27	1.390(6)	C1	C2	1.494(6)
C26	C25	1.393(6)	C13	C12	1.388(6)
C9	C10	1.380(6)	C12	C15	1.500(6)
C9	C14	1.391(6)	C19	C18	1.534(6)

C27	C28	1.392(6)	C18	C17	1.531(7)

Supplementary Table 6 Bond angles for **7x**.

Atom	Atom	Atom	Angle/°	Atom	Atom	Atom	Angle/°
O4	S2	N2	106.06(17)	C8	C5	C4	110.8(4)
O4	S2	C24	109.54(17)	C8	C5	C7	107.9(4)
O5	S2	O4	118.7(2)	C8	C5	C6	109.3(4)
O5	S2	N2	109.95(17)	C7	C5	C4	107.8(4)
O5	S2	C24	108.3(2)	C7	C5	C6	109.1(4)
N2	S2	C24	103.16(18)	C6	C5	C4	112.0(4)
O1	S1	O2	119.3(2)	O6	C16	N2	123.8(4)
O1	S1	N1	105.61(17)	O6	C16	C17	128.9(4)
O1	S1	C9	108.55(18)	N2	C16	C17	107.3(4)
O2	S1	N1	109.07(17)	C10	C11	C12	120.9(4)
O2	S1	C9	108.4(2)	C13	C14	C9	118.6(4)
N1	S1	C9	104.93(17)	C23	C20	C19	110.4(3)
C16	N2	S2	120.1(3)	C23	C20	C22	108.6(4)
C16	N2	C19	112.5(3)	C23	C20	C21	108.6(4)
C19	N2	S2	125.0(3)	C22	C20	C19	107.3(4)
C4	N1	S1	124.8(3)	C21	C20	C19	112.5(4)
C1	N1	S1	120.7(3)	C21	C20	C22	109.3(4)
C1	N1	C4	112.7(3)	C29	C28	C27	121.2(4)
C25	C24	S2	120.9(3)	C2	C3	C4	106.3(3)
C25	C24	C29	120.8(4)	O3	C1	N1	123.7(4)
C29	C24	S2	118.1(3)	O3	C1	C2	128.3(4)
C27	C26	C25	121.6(4)	N1	C1	C2	108.0(4)
C10	C9	S1	120.9(3)	C14	C13	C12	121.6(4)
C10	C9	C14	121.0(4)	C11	C12	C15	121.0(4)
C14	C9	S1	118.1(3)	C13	C12	C11	118.5(4)
C26	C27	C28	118.2(4)	C13	C12	C15	120.5(4)
C26	C27	C30	120.9(4)	N2	C19	C20	111.3(3)
C28	C27	C30	120.8(4)	N2	C19	C18	101.5(3)
C24	C25	C26	118.7(4)	C18	C19	C20	115.3(4)
C9	C10	C11	119.5(4)	C17	C18	C19	105.8(3)
N1	C4	C5	112.3(3)	C1	C2	C3	105.2(3)
N1	C4	C3	101.6(3)	C16	C17	C18	106.4(4)
C3	C4	C5	115.1(4)	C28	C29	C24	119.4(4)

Computations were carried out using the range separated hybrid functional with damped atom—atom dispersion (WB97XD)[16] as implemented in the GAUSSIAN 09 software package.[17] For palladium atom the SDD basis set[18] with the associated effective core potential was employed. All other atoms were described with 6-31G** basis with additional diffuse function for phosphorus.[19-23] Non-specific solvation was introduced by using the SMD continuum model[24] (2,2,2-trifluoroethanol).

Supplementary Table 7 Coordinates and Geometries of the optimized structures[a]

	E	E(ZPVE)	H	G
H_2	-1.174642	-1.164498	-1.161194	-1.175987
**C1**+**1a**+H_2	-2926.016163	-2925.210021	-2925.156555	-2925.330635
**C1**	-1623.20386	-1622.764477	-1622.736839	-1622.819727
**C1**+**1a**	-2924.841521	-2924.045523	-2923.995361	-2924.154648
**S1**	-2924.866251	-2924.066018	-2924.016252	-2924.146694
**TS(S)**	-2924.859582	-2924.061037	-2924.011867	-2924.139738
**S2**	-2924.876166	-2924.07224	-2924.023083	-2924.150913
**S2**+H_2	-2926.050808	-2925.236738	-2925.184277	-2925.3269
**S3**	-2925.196516	-2925.245373	-2925.195572	-2925.323778
**TS2(S)**	-2925.189665	-2925.238252	-2925.188721	-2925.317100
**S4**	-2925.232510	-2925.281636	-2925.231566	-2925.362592
**C1**+**2a**(*S*)	-2926.062955	-2925.24264	-2925.193058	-2925.348964
**R1**	-2924.873018	-2924.073437	-2924.023425	-2924.15501
**TS(R)**	-2924.854953	-2924.056723	-2924.007758	-2924.134475
**R2**	-2924.880186	-2924.077228	-2924.028111	-2924.155793
**TS2(R)**	-2924.869297	-2924.066519	-2924.017931	-2924.144162
**R3**	-2924.90311	-2924.099161	-2924.049966	-2924.178117
**R4**	-2926.072188	-2925.250088	-2925.200407	-2925.328883
**TS3(R)**	-2926.053738	-2925.234081	-2925.184778	-2925.31256
**R5**	-2926.102538	-2925.278072	-2925.228696	-2925.358932

[a] All the structures were fully optimized at WB97XD/SDD(Pd)/6-31G(d,p)(all others)/SMD(TFE).

Supplementary Table 8

C1

Cartesian coordinates

6	-0.722680	-0.648953	-0.320542
15	0.904010	-1.491985	-0.558497
6	-0.779277	0.627945	0.315430
6	-3.061956	0.514624	0.303723
6	-3.001528	-0.721558	-0.395902
15	0.794036	1.535426	0.614967
46	2.461404	0.028328	-0.189399
6	0.682384	3.072777	-0.442667
6	0.340282	2.631175	-1.872698
1	1.038861	1.870162	-2.240569
1	-0.675615	2.228807	-1.941648
6	-0.358023	4.071715	0.072208
1	-0.395966	4.928067	-0.611140
1	-1.356799	3.629838	0.121002
1	-0.097522	4.450627	1.065333
6	2.081975	3.707185	-0.412859
1	2.839065	3.031761	-0.827877
1	2.077314	4.621406	-1.017097
1	2.382924	3.981324	0.604090
6	0.666195	2.051805	2.358711
1	-0.290739	2.541517	2.553657
1	0.757618	1.165708	2.992297
1	1.485210	2.737258	2.591739
6	0.804583	-2.223654	-2.215633
1	1.646595	-2.905250	-2.355342
1	-0.138981	-2.760593	-2.334878
1	0.867080	-1.420373	-2.953526
6	0.930913	-2.861535	0.713720
6	-0.154671	-3.902772	0.406635
1	-0.127043	-4.667268	1.190770
1	-1.156442	-3.466977	0.394839
1	0.020198	-4.401775	-0.551210

6	0.692282	-2.229053	2.091966
1	0.764639	-3.010164	2.856345
1	1.443144	-1.464167	2.318855
1	-0.301767	-1.776452	2.169918
6	2.310849	-3.532235	0.675059
1	3.104025	-2.842179	0.976171
1	2.306902	-4.376055	1.373549
1	2.548927	-3.922595	-0.319793
7	-1.804239	-1.293906	-0.688133
7	-1.926109	1.180197	0.640707
6	-4.200853	-1.376993	-0.768231
6	-4.322277	1.069734	0.635662
6	-5.407106	-0.815974	-0.438314
1	-4.133201	-2.318458	-1.303443
6	-5.468023	0.412469	0.269610
1	-4.347985	2.012390	1.172452
1	-6.330324	-1.314143	-0.716027
1	-6.436879	0.832600	0.519591
1	3.322494	-1.120858	-0.844662
1	0.403008	3.496871	-2.541397

Supplementary Table 9

S1

Cartesian Coordinates

6	2.989803	-1.206916	0.062218
15	1.360289	-2.057900	-0.057579
6	3.037771	0.220351	0.088070
6	5.314791	0.122118	-0.124849
6	5.268025	-1.295515	-0.041486
15	1.481358	1.179516	0.380438
46	-0.208851	-0.534670	0.339985
6	1.770436	1.977491	2.055742
6	2.001353	0.851614	3.073307
1	1.187403	0.119113	3.066406
1	2.940797	0.321169	2.882781

6	2.958279	2.946133	2.066468
1	3.055480	3.371705	3.072279
1	3.898909	2.450700	1.816537
1	2.811224	3.777368	1.369953
6	0.490043	2.751451	2.398165
1	-0.390152	2.104090	2.398948
1	0.590247	3.189052	3.398163
1	0.314632	3.569358	1.692666
6	1.623970	2.519829	-0.846949
1	2.632279	2.940011	-0.843414
1	1.399715	2.121630	-1.838820
1	0.900957	3.302100	-0.608562
6	1.491055	-3.458011	1.092327
1	0.661576	-4.146443	0.914759
1	2.443500	-3.975886	0.960272
1	1.419392	-3.073815	2.113023
6	1.294269	-2.721579	-1.806811
6	2.365940	-3.799143	-2.020983
1	2.312610	-4.138479	-3.061313
1	3.374875	-3.421576	-1.839273
1	2.198806	-4.669245	-1.378876
6	1.516021	-1.543549	-2.766731
1	1.417645	-1.904098	-3.796521
1	0.770968	-0.754666	-2.614549
1	2.514604	-1.106499	-2.660783
6	-0.097124	-3.327510	-2.043237
1	-0.878415	-2.563076	-2.019374
1	-0.109207	-3.796029	-3.033429
1	-0.340784	-4.100436	-1.306862
7	4.076030	-1.943167	0.031482
7	4.177555	0.861351	-0.041942
6	6.471590	-2.042060	-0.072891
6	6.565168	0.772911	-0.266921
6	7.668584	-1.387201	-0.201773
1	6.413352	-3.123315	-0.001643
6	7.715032	0.027674	-0.304070

1	6.581834	1.855753	-0.336048
1	8.595556	-1.950762	-0.230550
1	8.676282	0.519839	-0.410906
1	-0.911524	-1.936095	0.291020
6	-3.284352	-0.550836	0.967619
7	-2.446636	0.244188	0.368646
16	-2.780042	0.803365	-1.212742
8	-2.071973	-0.047643	-2.171526
8	-4.211681	1.013057	-1.413405
6	-2.010800	2.395498	-1.167591
6	-2.131389	3.211688	-0.043795
6	-1.453563	2.866564	-2.352542
6	-1.662703	4.515798	-0.114773
1	-2.574725	2.836204	0.872567
6	-1.003704	4.181435	-2.405955
1	-1.368927	2.221145	-3.220575
6	-1.097340	5.021635	-1.293375
1	-1.740284	5.155311	0.759732
1	-0.564583	4.555266	-3.325971
6	-4.388535	-1.253921	0.250001
6	-5.742786	-0.956184	0.420340
6	-4.001357	-2.284786	-0.614296
6	-6.700046	-1.685843	-0.274896
1	-6.046787	-0.134895	1.058852
6	-4.968276	-3.020351	-1.292857
1	-2.947864	-2.517371	-0.740386
6	-6.317091	-2.721661	-1.125552
1	-7.750604	-1.442729	-0.151477
1	-4.662524	-3.823582	-1.955748
1	-7.070795	-3.291448	-1.660101
6	-3.070641	-0.881237	2.443106
6	-2.839830	-2.396730	2.599848
1	-3.684844	-2.978599	2.223055
1	-1.932368	-2.714740	2.076757
1	-2.718563	-2.620009	3.664133
6	-4.350773	-0.486783	3.214099

1	-4.156005	-0.624227	4.281509
1	-4.612572	0.563215	3.048923
1	-5.204502	-1.113696	2.950180
6	-1.898914	-0.110234	3.044846
1	-2.053504	0.968603	2.964627
1	-1.804588	-0.365440	4.104264
1	-0.949178	-0.366540	2.559551
1	2.061727	1.279947	4.080133
6	-0.575237	6.431224	-1.338195
1	0.350039	6.512964	-0.756913
1	-0.358056	6.743041	-2.362521
1	-1.295380	7.132748	-0.906400

Supplementary Table 10

TS(*S*) *v* = 318.4*i*

Cartesian Coordinates

6	2.797050	-1.271340	0.008532
15	1.129555	-2.003255	-0.199472
6	2.908317	0.147483	0.067597
6	5.179453	-0.045622	-0.096458
6	5.067306	-1.462454	-0.048816
15	1.388138	1.159364	0.354058
46	-0.399824	-0.446771	0.382982
6	1.712417	1.945428	2.029452
6	2.030577	0.823842	3.027324
1	1.261341	0.046166	3.034862
1	2.993336	0.349439	2.810564
6	2.870688	2.949752	1.988823
1	3.009077	3.359513	2.996230
1	3.809490	2.486435	1.678331
1	2.658208	3.788311	1.318607
6	0.428884	2.681509	2.432213
1	-0.436654	2.015706	2.449715
1	0.556121	3.103875	3.435536
1	0.215319	3.507040	1.747631

6	1.566843	2.489514	-0.874655
1	2.602152	2.837565	-0.900942
1	1.283511	2.112278	-1.859130
1	0.908870	3.317670	-0.604831
6	1.175625	-3.580233	0.694932
1	0.278351	-4.154562	0.451957
1	2.066740	-4.149954	0.422603
1	1.190614	-3.373711	1.767753
6	1.014324	-2.351149	-2.038947
6	2.076213	-3.369353	-2.470650
1	1.976227	-3.533870	-3.549231
1	3.093071	-3.017936	-2.278128
1	1.941813	-4.335325	-1.974790
6	1.206409	-1.025113	-2.789524
1	1.079453	-1.207594	-3.862047
1	0.460457	-0.281918	-2.486237
1	2.205922	-0.602900	-2.641594
6	-0.386137	-2.902331	-2.332506
1	-1.162274	-2.171314	-2.092650
1	-0.455392	-3.128523	-3.402282
1	-0.588459	-3.828274	-1.784569
7	3.846787	-2.058860	-0.022652
7	4.075267	0.742821	-0.025170
6	6.238027	-2.260278	-0.071891
6	6.460113	0.552059	-0.195252
6	7.465094	-1.656124	-0.158618
1	6.130859	-3.339286	-0.029405
6	7.576353	-0.242669	-0.225172
1	6.526293	1.634342	-0.237116
1	8.366724	-2.259738	-0.180969
1	8.560203	0.208970	-0.299473
1	-1.263541	-1.758419	0.519726
6	-2.795936	-0.766769	0.967328
7	-2.429451	0.487956	0.637084
16	-2.913239	1.118988	-0.854963
8	-2.505888	0.262831	-1.975293

8	-4.330775	1.474386	-0.750689
6	-2.015773	2.638660	-0.951397
6	-2.047595	3.539105	0.112986
6	-1.483004	2.992535	-2.186553
6	-1.503413	4.802181	-0.067106
1	-2.485647	3.259323	1.065475
6	-0.959051	4.270833	-2.351565
1	-1.479015	2.283323	-3.007324
6	-0.957980	5.189405	-1.299837
1	-1.510476	5.507883	0.758724
1	-0.538570	4.552138	-3.312255
6	-3.693951	-1.645091	0.119086
6	-5.035715	-1.278161	-0.045793
6	-3.265316	-2.866808	-0.405980
6	-5.905943	-2.095219	-0.761439
1	-5.405589	-0.355757	0.382649
6	-4.133741	-3.676836	-1.128134
1	-2.243216	-3.191408	-0.253746
6	-5.457563	-3.290262	-1.315355
1	-6.940385	-1.789958	-0.883355
1	-3.769365	-4.612302	-1.541105
1	-6.136358	-3.919603	-1.882368
6	-2.792199	-1.095164	2.480651
6	-2.510530	-2.578590	2.745531
1	-3.231990	-3.233304	2.250384
1	-1.503514	-2.857432	2.416685
1	-2.577241	-2.762597	3.821845
6	-4.208130	-0.749285	2.996360
1	-4.206875	-0.844626	4.086259
1	-4.484214	0.279755	2.745009
1	-4.968308	-1.428013	2.603206
6	-1.784558	-0.242061	3.249553
1	-2.018530	0.821766	3.174436
1	-1.805644	-0.524341	4.306134
1	-0.766572	-0.404812	2.877990
1	2.089597	1.247432	4.036001

6	-0.366535	6.561795	-1.465882
1	-0.154714	6.781110	-2.515096
1	-1.043646	7.329722	-1.079729
1	0.572098	6.643456	-0.906474

Supplementary Table 11

S2

Cartesian coordinates

6	2.968561	-1.163853	0.081616
15	1.367857	-2.000450	-0.160507
6	2.932033	0.254157	0.064123
6	5.205000	0.306040	-0.000755
6	5.240143	-1.113406	0.122790
15	1.302482	1.084278	0.268279
46	-0.333697	-0.518974	0.210321
6	1.395092	1.834422	1.983091
6	1.618904	0.694481	2.985086
1	0.838409	-0.068203	2.921023
1	2.589637	0.209029	2.837319
6	2.541898	2.850446	2.084035
1	2.496158	3.305443	3.079788
1	3.523166	2.386326	1.968275
1	2.445884	3.654285	1.348352
6	0.068288	2.556297	2.248913
1	-0.800484	1.936036	2.017617
1	0.025342	2.833902	3.307613
1	-0.002544	3.473550	1.658700
6	1.326089	2.433696	-0.939849
1	2.324752	2.878119	-0.946329
1	1.090366	2.044872	-1.931505
1	0.591937	3.187443	-0.653781
6	1.361279	-3.467174	0.904342
1	0.487824	-4.080206	0.667787
1	2.273979	-4.049252	0.757491
1	1.297383	-3.139292	1.944875

6	1.400020	-2.523266	-1.959026
6	2.414541	-3.654643	-2.163733
1	2.449016	-3.896844	-3.231750
1	3.422744	-3.368286	-1.851304
1	2.125168	-4.561580	-1.624380
6	1.781366	-1.302080	-2.809257
1	1.695928	-1.572213	-3.866953
1	1.113277	-0.452936	-2.626182
1	2.812329	-0.980682	-2.631063
6	-0.006154	-3.000546	-2.343580
1	-0.750741	-2.205005	-2.232617
1	0.004008	-3.307526	-3.395189
1	-0.318859	-3.865633	-1.751038
7	4.091898	-1.840360	0.132535
7	4.023302	0.976560	-0.016625
6	6.488888	-1.777623	0.199298
6	6.418819	1.032595	-0.076763
6	7.647359	-1.048456	0.136685
1	6.496364	-2.857782	0.300861
6	7.612308	0.363241	-0.007885
1	6.370664	2.111622	-0.179334
1	8.607146	-1.551189	0.194656
1	8.546396	0.912891	-0.060594
1	-1.719914	-1.618758	1.080280
6	-2.664112	-0.974401	1.021917
7	-2.207221	0.357540	0.552742
16	-2.958104	0.998284	-0.750686
8	-2.725018	0.236488	-1.996200
8	-4.370559	1.263030	-0.426562
6	-2.191791	2.583538	-0.961406
6	-2.262371	3.528792	0.061178
6	-1.692740	2.918736	-2.215046
6	-1.783376	4.809777	-0.172335
1	-2.682450	3.267562	1.026859
6	-1.229326	4.212351	-2.437000
1	-1.666470	2.177746	-3.006198

6	-1.260595	5.171405	-1.422606
1	-1.823896	5.547973	0.623725
1	-0.833846	4.475472	-3.413640
6	-3.534762	-1.841274	0.107476
6	-4.902274	-1.633652	-0.105986
6	-2.935568	-2.948283	-0.497710
6	-5.629349	-2.493679	-0.923258
1	-5.404843	-0.788752	0.343462
6	-3.656692	-3.807261	-1.322114
1	-1.881755	-3.142640	-0.320257
6	-5.011341	-3.579426	-1.540460
1	-6.688109	-2.310289	-1.079753
1	-3.158679	-4.653347	-1.786446
1	-5.584749	-4.243112	-2.180469
6	-3.095665	-0.943313	2.530827
6	-3.558688	-2.342807	2.958524
1	-4.485022	-2.641009	2.460693
1	-2.796064	-3.099371	2.737338
1	-3.737800	-2.356241	4.038546
6	-4.206959	0.083117	2.776109
1	-4.412365	0.143515	3.850281
1	-3.906378	1.076565	2.431929
1	-5.142418	-0.187280	2.280888
6	-1.878945	-0.563590	3.381742
1	-1.518590	0.438698	3.143947
1	-2.143602	-0.578868	4.444148
1	-1.058708	-1.276515	3.232361
1	1.603637	1.107862	3.999061
6	-0.735745	6.562388	-1.649719
1	-0.556972	6.753165	-2.710773
1	-1.438404	7.313244	-1.275705
1	0.210723	6.707524	-1.117034

Supplementary Table 12

S3

Cartesian coordinates

6	-2.902216	-0.232724	0.826927
15	-1.449997	0.396004	1.721493
6	-2.709885	-0.544307	-0.538606
6	-4.956769	-0.830952	-0.773293
6	-5.132264	-0.609090	0.623614
15	-0.985700	-0.657826	-1.181430
46	0.467563	0.017957	0.527965
6	-0.820038	-2.473355	-1.620487
6	-1.166615	-3.276182	-0.358644
1	-0.557587	-2.972417	0.498343
1	-2.223635	-3.178640	-0.090213
6	-1.761201	-2.856102	-2.770695
1	-1.619890	-3.923501	-2.974726
1	-2.812470	-2.692152	-2.527455
1	-1.523106	-2.308664	-3.687465
6	0.623512	-2.744761	-2.048856
1	1.338631	-2.437910	-1.286953
1	0.739703	-3.821386	-2.214671
1	0.870447	-2.227986	-2.979529
6	-1.102571	0.333797	-2.696986
1	-1.999862	0.025589	-3.239155
1	-1.192121	1.386089	-2.417688
1	-0.212134	0.191106	-3.305795
6	-1.467151	-0.402989	3.348439
1	-0.687006	0.030294	3.978448
1	-2.443872	-0.267148	3.819055
1	-1.270011	-1.469273	3.210899
6	-1.715630	2.236157	1.902042
6	-2.992460	2.513940	2.706535
1	-3.109045	3.599682	2.793676
1	-3.885437	2.117212	2.217909
1	-2.933814	2.101620	3.718211
6	-1.825678	2.831805	0.490774
1	-1.926613	3.919021	0.575520
1	-0.932907	2.621484	-0.107664

1	-2.703782	2.455497	-0.044739
6	-0.506135	2.829744	2.636760
1	0.418745	2.703491	2.070761
1	-0.675543	3.904242	2.764612
1	-0.374514	2.391750	3.631324
7	-4.076416	-0.282336	1.411783
7	-3.721245	-0.800170	-1.335144
6	-6.428712	-0.693574	1.189433
6	-6.084429	-1.111995	-1.583934
6	-7.499650	-0.975361	0.382993
1	-6.541221	-0.523583	2.255142
6	-7.326954	-1.182284	-1.011147
1	-5.931422	-1.274512	-2.645742
1	-8.496006	-1.039219	0.807968
1	-8.194515	-1.401695	-1.624901
1	4.270239	-1.007274	-0.763636
6	3.623943	-0.899001	0.113739
7	2.505268	0.011090	-0.269249
16	2.666841	0.775932	-1.674557
8	4.082493	1.034804	-2.006706
8	1.930099	0.153277	-2.796199
6	1.876303	2.348137	-1.367656
6	1.131634	2.944122	-2.381418
6	2.093709	3.018951	-0.163678
6	0.580649	4.205333	-2.175283
1	0.978942	2.425756	-3.321538
6	1.545700	4.281351	0.023812
1	2.677436	2.552642	0.623469
6	0.774992	4.890346	-0.972827
1	-0.007547	4.664122	-2.965119
1	1.710953	4.798999	0.964606
6	3.121566	-2.307224	0.404552
6	2.213537	-2.613898	1.424153
6	3.600191	-3.360317	-0.381917
6	1.780349	-3.921725	1.630322
1	1.828238	-1.834992	2.072604

6	3.172345	-4.669990	-0.179698
1	4.309687	-3.148442	-1.177670
6	2.251995	-4.955645	0.825184
1	1.068111	-4.130617	2.423633
1	3.554312	-5.465194	-0.813133
1	1.909354	-5.973714	0.983776
6	4.612211	-0.356101	1.217360
6	5.598366	-1.486508	1.562778
1	5.114657	-2.303904	2.105970
1	6.057783	-1.906141	0.660236
1	6.402504	-1.094393	2.194888
6	3.950017	0.126807	2.512223
1	3.281865	-0.614590	2.957426
1	4.728992	0.347137	3.251012
1	3.396683	1.057203	2.352214
6	5.432132	0.818207	0.663794
1	6.132779	1.167585	1.430997
1	6.012221	0.524818	-0.216377
1	4.797573	1.661000	0.380227
1	-0.965810	-4.335992	-0.548366
6	0.139316	6.232285	-0.733867
1	-0.068723	6.749507	-1.674140
1	-0.812737	6.112286	-0.203214
1	0.778797	6.870726	-0.117902
1	1.557291	0.752888	1.793701
1	1.124828	0.261913	2.229591

Supplementary Table 13

TS2(S) *v* = 926.4*i*

Cartesian coorates

6	-2.894866	-0.112665	0.812293
15	-1.390544	0.495431	1.652504
6	-2.775119	-0.481592	-0.552331
6	-5.038352	-0.719768	-0.672021
6	-5.142702	-0.441172	0.721043

15	-1.085241	-0.685290	-1.252354
46	0.426509	0.014121	0.391158
6	-1.001659	-2.496791	-1.709202
6	-1.438089	-3.298005	-0.474440
1	-0.884866	-2.999458	0.421565
1	-2.508831	-3.186898	-0.276006
6	-1.906945	-2.819709	-2.904390
1	-1.848126	-3.897500	-3.094530
1	-2.951587	-2.562895	-2.715188
1	-1.576851	-2.303984	-3.811065
6	0.452573	-2.817669	-2.070056
1	1.130811	-2.601120	-1.243683
1	0.529767	-3.885264	-2.303576
1	0.784742	-2.255071	-2.947294
6	-1.133388	0.305506	-2.774388
1	-2.016171	0.034029	-3.358328
1	-1.188172	1.362149	-2.501718
1	-0.225699	0.127291	-3.352487
6	-1.357119	-0.295339	3.282637
1	-0.504972	0.086427	3.849195
1	-2.286724	-0.091001	3.819785
1	-1.243349	-1.373106	3.141280
6	-1.611172	2.340928	1.830763
6	-2.899771	2.663576	2.599507
1	-2.966869	3.751658	2.706910
1	-3.793107	2.321461	2.072548
1	-2.897375	2.228196	3.603249
6	-1.668357	2.925622	0.412883
1	-1.759088	4.015074	0.480298
1	-0.759576	2.694973	-0.151719
1	-2.532601	2.552268	-0.147417
6	-0.406720	2.906740	2.595395
1	0.537132	2.717820	2.078183
1	-0.530806	3.991938	2.677305
1	-0.341023	2.499771	3.609459
7	-4.043111	-0.114744	1.447995

7	-3.829135	-0.745370	-1.289585
6	-6.412559	-0.471517	1.348977
6	-6.209027	-1.002741	-1.418336
6	-7.526939	-0.755640	0.604324
1	-6.470585	-0.259230	2.411446
6	-7.424918	-1.019338	-0.786955
1	-6.110076	-1.209870	-2.478847
1	-8.503335	-0.777798	1.077140
1	-8.325187	-1.239672	-1.351376
1	4.309530	-1.180943	-0.677395
6	3.658873	-1.037070	0.191843
7	2.652553	-0.004454	-0.184046
16	2.845968	0.737174	-1.619865
8	4.272221	0.938841	-1.926713
8	2.093530	0.078560	-2.705062
6	2.080649	2.319857	-1.337537
6	1.263186	2.855145	-2.327922
6	2.370793	3.046941	-0.181757
6	0.709969	4.119000	-2.144393
1	1.054011	2.288392	-3.228149
6	1.808532	4.304278	-0.013739
1	3.014727	2.632350	0.587486
6	0.967746	4.857813	-0.987439
1	0.060640	4.531018	-2.911494
1	2.018586	4.863498	0.893587
6	3.004174	-2.383791	0.449794
6	1.987526	-2.566929	1.393942
6	3.444451	-3.497381	-0.270815
6	1.422886	-3.822844	1.600996
1	1.618199	-1.725340	1.972899
6	2.880766	-4.755091	-0.068731
1	4.233735	-3.376389	-1.008148
6	1.863666	-4.921910	0.867143
1	0.632120	-3.940596	2.336334
1	3.234055	-5.602844	-0.648220
1	1.418201	-5.899394	1.025205

6	4.639519	-0.594112	1.338266
6	5.561474	-1.783339	1.657274
1	5.020530	-2.604850	2.136486
1	6.037756	-2.174073	0.750684
1	6.355286	-1.461813	2.339900
6	3.945247	-0.152459	2.633768
1	3.265966	-0.910925	3.031378
1	4.706449	0.033927	3.399505
1	3.393507	0.783802	2.504889
6	5.517128	0.567348	0.853753
1	6.214738	0.855955	1.647905
1	6.102697	0.286919	-0.027028
1	4.922777	1.447247	0.595613
1	-1.237430	-4.360421	-0.649700
6	0.359132	6.217092	-0.779378
1	-0.457926	6.400405	-1.481448
1	-0.026770	6.321049	0.239310
1	1.110631	7.001438	-0.923989
1	1.967317	0.579248	1.026217
1	1.323400	0.690586	1.684422

Supplementary Table 14

S4

Cartesian coordinates

6	-2.830972	-0.274535	0.848240
15	-1.374906	0.359888	1.777325
6	-2.685850	-0.595262	-0.534254
6	-4.951093	-0.884065	-0.680369
6	-5.076475	-0.658203	0.716721
15	-0.990694	-0.736369	-1.263141
46	0.451623	-0.009011	0.549035
6	-0.880254	-2.567060	-1.673890
6	-1.212368	-3.343067	-0.391066
1	-0.638054	-2.979509	0.467083
1	-2.277688	-3.276288	-0.146034

6	-1.827075	-2.992255	-2.801168
1	-1.734622	-4.075715	-2.942076
1	-2.871386	-2.767199	-2.572281
1	-1.567891	-2.513152	-3.750245
6	0.567060	-2.835360	-2.103860
1	1.277397	-2.562936	-1.321865
1	0.686913	-3.903529	-2.317845
1	0.825740	-2.281319	-3.012118
6	-1.214351	0.131022	-2.853839
1	-2.096432	-0.250526	-3.372395
1	-1.352408	1.196304	-2.655052
1	-0.330443	-0.008832	-3.479698
6	-1.495101	-0.427732	3.409475
1	-0.797644	0.061142	4.093628
1	-2.513250	-0.356003	3.797563
1	-1.214070	-1.479162	3.307498
6	-1.715472	2.191706	1.969720
6	-2.950932	2.435222	2.846008
1	-3.132666	3.514569	2.896237
1	-3.847266	1.960570	2.439630
1	-2.800129	2.074769	3.868171
6	-1.931354	2.770731	0.564069
1	-2.028795	3.859281	0.638439
1	-1.084661	2.552150	-0.095461
1	-2.844018	2.385119	0.097260
6	-0.490483	2.847995	2.621425
1	0.393918	2.774519	1.983410
1	-0.707160	3.909920	2.782882
1	-0.255717	2.402062	3.593864
7	-3.989363	-0.331580	1.463275
7	-3.733646	-0.857070	-1.282951
6	-6.350849	-0.740841	1.330187
6	-6.106311	-1.169342	-1.449471
6	-7.451419	-1.025827	0.564556
1	-6.424572	-0.567194	2.398876
6	-7.328762	-1.238236	-0.833300

1	-5.991035	-1.336654	-2.515502
1	-8.431527	-1.088472	1.026176
1	-8.217279	-1.460888	-1.415314
1	4.446471	-0.738929	-0.653614
6	3.781416	-0.754354	0.215836
7	2.691718	0.264806	-0.046835
16	2.843132	1.001037	-1.594885
8	4.231593	1.407683	-1.805204
8	2.259498	0.083891	-2.567160
6	1.836717	2.444510	-1.410412
6	0.917965	2.729211	-2.413631
6	2.053167	3.328294	-0.349895
6	0.175776	3.902695	-2.333095
1	0.789583	2.051670	-3.249045
6	1.293872	4.486351	-0.283376
1	2.800551	3.136147	0.414742
6	0.343835	4.789908	-1.267877
1	-0.544628	4.127385	-3.113745
1	1.445041	5.171695	0.545206
6	3.231953	-2.161736	0.313915
6	2.242762	-2.534796	1.228837
6	3.784586	-3.145412	-0.510737
6	1.818139	-3.858196	1.313278
1	1.788397	-1.791856	1.878219
6	3.361547	-4.469567	-0.428405
1	4.554161	-2.871170	-1.227330
6	2.374886	-4.830108	0.485365
1	1.046208	-4.128323	2.027594
1	3.800456	-5.216838	-1.082472
1	2.039757	-5.860697	0.551463
6	4.691523	-0.326323	1.418159
6	5.737545	-1.431108	1.629183
1	5.281631	-2.363378	1.974192
1	6.287816	-1.640638	0.705127
1	6.459894	-1.109069	2.385936
6	3.918470	-0.114925	2.726935

1	3.432195	-1.030453	3.072085
1	4.619781	0.199832	3.506635
1	3.157542	0.669195	2.644161
6	5.434508	0.972703	1.066543
1	6.114815	1.232422	1.883677
1	6.025249	0.859675	0.152962
1	4.759944	1.825273	0.930439
1	-0.966468	-4.401639	-0.531822
6	-0.488024	6.036046	-1.156088
1	-1.002741	6.259141	-2.093639
1	-1.246738	5.914608	-0.374463
1	0.128382	6.896845	-0.880883
1	2.806229	1.026442	0.624397
1	0.989281	0.450568	1.947285

Supplementary Table 15

R1

Cartesian coordinates

6	2.973430	-1.168982	-0.092393
15	1.307620	-1.769206	-0.612462
6	3.093220	0.107335	0.538711
6	5.369486	-0.067567	0.423665
6	5.246047	-1.371280	-0.129095
15	1.559161	0.952840	1.111992
46	-0.203891	-0.462387	0.358796
6	1.719563	1.004070	2.975764
6	1.995658	-0.427419	3.457493
1	1.251443	-1.135605	3.073624
1	2.989749	-0.775825	3.157543
6	2.829048	1.950571	3.444768
1	2.915709	1.881630	4.535445
1	3.798262	1.693943	3.009682
1	2.602960	2.992157	3.195829
6	0.363883	1.481995	3.519045
1	-0.434483	0.768045	3.296176

1	0.433164	1.588681	4.607598
1	0.078364	2.456171	3.107413
6	1.738322	2.658518	0.496092
1	2.711268	3.077892	0.762886
1	1.632140	2.645968	-0.591535
1	0.942152	3.276272	0.919036
6	1.302929	-3.536531	-0.203444
1	0.431210	-4.005055	-0.666750
1	2.218151	-4.015618	-0.557524
1	1.228406	-3.645220	0.881303
6	1.316443	-1.600171	-2.476584
6	2.286279	-2.604034	-3.114065
1	2.317680	-2.419287	-4.193544
1	3.301985	-2.500996	-2.723794
1	1.957644	-3.636609	-2.962503
6	1.740894	-0.162618	-2.809357
1	1.591682	0.016756	-3.879509
1	1.147684	0.573043	-2.256727
1	2.798526	0.010186	-2.585159
6	-0.106063	-1.865355	-2.989540
1	-0.815584	-1.119598	-2.621759
1	-0.099471	-1.822769	-4.084413
1	-0.467912	-2.856329	-2.695482
7	4.021780	-1.899022	-0.393267
7	4.267534	0.652113	0.763000
6	6.411042	-2.115794	-0.438179
6	6.657410	0.480552	0.643052
6	7.644978	-1.563480	-0.212544
1	6.295392	-3.109101	-0.859501
6	7.768857	-0.257637	0.329047
1	6.732725	1.477579	1.064713
1	8.542448	-2.124991	-0.451380
1	8.758198	0.156449	0.494464
1	-1.141650	-1.589139	-0.217626
6	-4.033754	-0.682167	-0.563866
6	-4.338153	-1.073039	0.847440

6	-3.598949	-2.049640	1.520930
6	-5.397233	-0.430212	1.495996
6	-3.909058	-2.363837	2.840318
1	-2.764955	-2.538459	1.029905
6	-5.710532	-0.761618	2.810743
1	-5.974035	0.325581	0.972739
6	-4.965249	-1.724307	3.486195
1	-3.323108	-3.113586	3.362916
1	-6.537234	-0.262382	3.306092
1	-5.205176	-1.975126	4.514813
6	-4.495862	-1.585428	-1.701730
6	-3.778626	-2.945501	-1.585853
1	-2.690300	-2.822949	-1.586524
1	-4.072205	-3.491844	-0.686341
1	-4.052962	-3.555557	-2.452115
6	-4.178827	-0.966652	-3.065533
1	-4.660356	0.007001	-3.191379
1	-3.101672	-0.836171	-3.207269
1	-4.545601	-1.634637	-3.850708
6	-6.016538	-1.794794	-1.565987
1	-6.554361	-0.843505	-1.632137
1	-6.358763	-2.437153	-2.383103
1	-6.278107	-2.279054	-0.621321
7	-3.446692	0.418144	-0.897277
16	-2.823284	1.455090	0.249329
8	-3.854169	2.229906	0.931991
8	-1.891670	0.726351	1.157748
6	-1.862272	2.535312	-0.769246
6	-1.200654	2.056911	-1.897295
6	-1.729089	3.858604	-0.354040
6	-0.393440	2.927548	-2.618666
1	-1.324607	1.029887	-2.221812
6	-0.913607	4.710334	-1.088220
1	-2.257209	4.221876	0.521306
6	-0.233432	4.260495	-2.225292
1	0.118966	2.561988	-3.503716

1	-0.806680	5.744254	-0.773711
1	1.949930	-0.454700	4.552037
6	0.670847	5.185826	-2.990800
1	1.626628	5.300635	-2.466835
1	0.881178	4.798863	-3.990773
1	0.228938	6.181511	-3.087704

Supplementary Table 16

TS(*R*) *v* = 837.1*i*

Cartesian coordinates

6	-2.778998	0.132109	0.809933
15	-1.092408	0.532463	1.422723
6	-2.923427	-0.263426	-0.548537
6	-5.189042	-0.169123	-0.331394
6	-5.043145	0.144600	1.049817
15	-1.395964	-0.705634	-1.459343
46	0.402352	-0.239343	-0.059399
6	-1.534651	-2.543688	-1.778197
6	-1.885938	-3.224364	-0.447013
1	-1.199788	-2.931739	0.354600
1	-2.908671	-2.995308	-0.131179
6	-2.610798	-2.839431	-2.830029
1	-2.702136	-3.925869	-2.940092
1	-3.588168	-2.444654	-2.539957
1	-2.344437	-2.427921	-3.808024
6	-0.166778	-3.033308	-2.277339
1	0.611611	-2.911113	-1.519361
1	-0.242290	-4.100288	-2.514600
1	0.148494	-2.508301	-3.185087
6	-1.492255	0.178366	-3.041789
1	-2.445939	-0.018746	-3.537101
1	-1.399766	1.249983	-2.847115
1	-0.669362	-0.144380	-3.684732
6	-1.056082	-0.088948	3.125906
1	-0.250366	0.404483	3.673461

1	-2.011018	0.123601	3.612703
1	-0.884314	-1.167661	3.113007
6	-1.069287	2.405552	1.472023
6	-1.953229	2.915899	2.617872
1	-1.975186	4.010501	2.575453
1	-2.982316	2.555108	2.538282
1	-1.556358	2.628182	3.595833
6	-1.586274	2.931682	0.125829
1	-1.440106	4.016750	0.092948
1	-1.041684	2.496896	-0.719095
1	-2.654687	2.734724	-0.009035
6	0.380757	2.854825	1.689315
1	1.023215	2.561511	0.854297
1	0.403800	3.946701	1.776022
1	0.796540	2.437413	2.612352
7	-3.811376	0.310002	1.599631
7	-4.100775	-0.383616	-1.116776
6	-6.196463	0.313045	1.855404
6	-6.485894	-0.288475	-0.888875
6	-7.438810	0.184499	1.291595
1	-6.064340	0.546111	2.906861
6	-7.584410	-0.113942	-0.088320
1	-6.577712	-0.523123	-1.944238
1	-8.326897	0.314636	1.901322
1	-8.580349	-0.208247	-0.508636
1	1.624790	-0.036397	1.089566
6	3.133329	-0.726703	0.982645
6	2.732561	-2.158316	0.701365
6	1.620635	-2.779309	1.283785
6	3.566034	-2.912923	-0.131406
6	1.350061	-4.119942	1.036653
1	0.947876	-2.204617	1.911693
6	3.291197	-4.255107	-0.380270
1	4.441022	-2.450457	-0.577299
6	2.183790	-4.863228	0.203086
1	0.477555	-4.581489	1.488156

1	3.948899	-4.824961	-1.029091
1	1.969439	-5.909464	0.008533
6	3.625572	-0.396354	2.410526
6	2.701418	-0.919996	3.513411
1	1.691760	-0.514409	3.412128
1	2.640051	-2.010726	3.526892
1	3.097836	-0.601420	4.482322
6	3.794416	1.118118	2.571531
1	4.565536	1.511379	1.906944
1	2.857060	1.641042	2.357753
1	4.082786	1.344107	3.602778
6	5.001588	-1.083158	2.553172
1	5.704201	-0.725117	1.795396
1	5.412345	-0.852668	3.541178
1	4.919084	-2.170860	2.466019
7	3.773501	-0.011770	0.044414
16	3.176908	0.154095	-1.425713
8	4.188246	-0.088442	-2.455981
8	1.902378	-0.620519	-1.637589
6	2.673534	1.862298	-1.457486
6	3.387674	2.822609	-0.744211
6	1.556962	2.213430	-2.214016
6	2.954932	4.142573	-0.771925
1	4.251655	2.538968	-0.154640
6	1.139638	3.538606	-2.229317
1	1.009891	1.461219	-2.771461
6	1.824185	4.519685	-1.505230
1	3.499849	4.890912	-0.203739
1	0.260293	3.811841	-2.805666
1	-1.809615	-4.309771	-0.572640
6	1.367211	5.952199	-1.530352
1	1.580814	6.453958	-0.582700
1	1.887119	6.505014	-2.321281
1	0.294305	6.023715	-1.727977

Supplementary Table 17

R2

Standard orientation:

6	-2.862511	0.135704	0.810616
15	-1.158472	0.409444	1.404165
6	-2.998621	-0.283064	-0.535452
6	-5.254904	-0.106195	-0.370336
6	-5.121020	0.254665	1.004193
15	-1.470925	-0.796302	-1.398673
46	0.321169	-0.180747	-0.195229
6	-1.540878	-2.656373	-1.513467
6	-1.905433	-3.200845	-0.125044
1	-1.238538	-2.816288	0.653510
1	-2.938725	-2.964084	0.147727
6	-2.603006	-3.062358	-2.544715
1	-2.695191	-4.153644	-2.523566
1	-3.584776	-2.636921	-2.318698
1	-2.317372	-2.770053	-3.559241
6	-0.159497	-3.166988	-1.945622
1	0.602741	-2.962217	-1.189764
1	-0.223086	-4.251513	-2.083548
1	0.162262	-2.725502	-2.893821
6	-1.500233	-0.043252	-3.041697
1	-2.413993	-0.343537	-3.561276
1	-1.484572	1.043171	-2.929377
1	-0.624465	-0.372954	-3.604978
6	-0.996687	-0.466709	2.979890
1	-0.017148	-0.253658	3.415458
1	-1.782831	-0.140078	3.665377
1	-1.094477	-1.539185	2.797362
6	-0.980248	2.247341	1.667313
6	-1.720314	2.679828	2.937875
1	-1.642752	3.769052	3.026198
1	-2.781673	2.417982	2.902819
1	-1.278195	2.240497	3.836742

6	-1.563733	2.953729	0.435382
1	-1.316110	4.019365	0.488288
1	-1.145272	2.559143	-0.496083
1	-2.653414	2.862986	0.394574
6	0.521851	2.546957	1.793176
1	1.059738	2.299762	0.870740
1	0.653835	3.617507	1.982596
1	0.978329	2.003085	2.627209
7	-3.896756	0.381963	1.578958
7	-4.163329	-0.388284	-1.128531
6	-6.281350	0.504348	1.777810
6	-6.545303	-0.195442	-0.947629
6	-7.516576	0.404497	1.194054
1	-6.159059	0.773260	2.821698
6	-7.648987	0.056056	-0.175828
1	-6.627723	-0.466992	-1.994695
1	-8.409801	0.596389	1.779451
1	-8.640226	-0.014292	-0.611333
6	3.218597	-0.629408	0.781405
6	2.778028	-2.097195	0.731121
6	1.700679	-2.553302	1.498010
6	3.450773	-3.019795	-0.073081
6	1.310908	-3.890804	1.472896
1	1.158824	-1.856153	2.129082
6	3.063376	-4.357683	-0.104274
1	4.277438	-2.678786	-0.687033
6	1.992851	-4.800734	0.669740
1	0.468739	-4.216938	2.076265
1	3.602016	-5.055530	-0.738860
1	1.690036	-5.843118	0.641629
6	4.115038	-0.318153	2.030929
6	3.349419	-0.602204	3.330661
1	2.397292	-0.056770	3.362143
1	3.140170	-1.666035	3.468442
1	3.946855	-0.272689	4.187289
6	4.497743	1.166096	2.021352

1	5.167211	1.397621	1.190441
1	3.611460	1.807399	1.942188
1	5.015014	1.424372	2.951686
6	5.391177	-1.167820	1.994490
1	5.935714	-1.016229	1.057701
1	6.051987	-0.885176	2.821432
1	5.171206	-2.235549	2.094176
7	3.912970	-0.224204	-0.432306
16	3.076229	0.192197	-1.659768
8	3.841970	0.079364	-2.910730
8	1.728793	-0.539903	-1.775230
6	2.548522	1.904994	-1.522855
6	3.339995	2.811005	-0.820806
6	1.364599	2.327974	-2.124513
6	2.914594	4.125219	-0.675401
1	4.271406	2.484713	-0.375244
6	0.950839	3.647892	-1.973868
1	0.763185	1.631866	-2.698335
6	1.706061	4.559845	-1.232139
1	3.525754	4.822638	-0.108985
1	0.018804	3.969139	-2.430220
1	-1.803437	-4.290781	-0.141612
6	1.230802	5.970760	-1.016882
1	1.072675	6.165206	0.049487
1	1.971721	6.694634	-1.371441
1	0.289253	6.159133	-1.538617
1	2.301116	-0.005699	0.966464

Supplementary Table 18

TS2(*R*) v = 62.6i

Standard orientation:

6	-2.722184	-0.219133	0.892643
15	-1.043217	0.038972	1.567378
6	-2.824834	-0.392118	-0.509408
6	-5.089677	-0.360702	-0.356910

6	-4.987560	-0.255107	1.062109
15	-1.268807	-0.674423	-1.424933
46	0.483697	-0.204755	-0.101655
6	-1.253593	-2.493766	-1.845559
6	-1.547556	-3.280165	-0.560386
1	-0.844969	-3.029847	0.241215
1	-2.567073	-3.109498	-0.200042
6	-2.328983	-2.773132	-2.905620
1	-2.347219	-3.852594	-3.090813
1	-3.326471	-2.469356	-2.576904
1	-2.102598	-2.274985	-3.852794
6	0.131512	-2.864405	-2.392381
1	0.914341	-2.721250	-1.644290
1	0.115522	-3.922889	-2.673044
1	0.388399	-2.282205	-3.282277
6	-1.345497	0.326836	-2.927262
1	-2.284669	0.119921	-3.446901
1	-1.303085	1.382291	-2.650026
1	-0.499520	0.078544	-3.570634
6	-0.882719	-1.068549	2.990569
1	0.073768	-0.882881	3.484563
1	-1.702465	-0.896806	3.692447
1	-0.916408	-2.101065	2.635033
6	-0.979519	1.816976	2.125770
6	-1.918548	2.052202	3.313880
1	-1.856951	3.109473	3.594507
1	-2.959138	1.826180	3.067659
1	-1.627340	1.457826	4.185011
6	-1.377508	2.702394	0.935392
1	-1.250806	3.752242	1.220144
1	-0.748672	2.513977	0.058178
1	-2.425004	2.556087	0.652053
6	0.472520	2.093676	2.534631
1	1.155774	1.946370	1.693406
1	0.560558	3.134638	2.863793
1	0.788775	1.449262	3.361616

7	-3.776462	-0.173498	1.672173
7	-3.977561	-0.443116	-1.132604
6	-6.166231	-0.212316	1.846757
6	-6.368143	-0.405053	-0.965359
6	-7.389277	-0.262581	1.231448
1	-6.068168	-0.133891	2.924390
6	-7.490694	-0.356957	-0.181455
1	-6.424993	-0.482905	-2.045983
1	-8.296248	-0.228413	1.826052
1	-8.473328	-0.393059	-0.640171
6	2.976571	-0.505040	0.954224
6	2.881953	-1.950834	0.465316
6	1.974114	-2.835324	1.056524
6	3.706475	-2.426049	-0.557371
6	1.893149	-4.163173	0.641854
1	1.319573	-2.481761	1.848222
6	3.626967	-3.751541	-0.974738
1	4.409687	-1.750825	-1.034818
6	2.721067	-4.626243	-0.376548
1	1.174989	-4.830044	1.109667
1	4.274661	-4.101615	-1.773079
1	2.657179	-5.658248	-0.707827
6	3.954966	-0.360205	2.183098
6	3.423421	-1.158094	3.378978
1	2.410594	-0.837993	3.653653
1	3.401538	-2.233826	3.183853
1	4.070135	-0.993540	4.247031
6	4.054419	1.115331	2.589668
1	4.505905	1.716444	1.797796
1	3.071497	1.534969	2.823402
1	4.676837	1.207129	3.486428
6	5.352305	-0.866112	1.806435
1	5.726938	-0.355959	0.913673
1	6.048742	-0.666954	2.628127
1	5.359681	-1.943725	1.616983
7	3.374173	0.457308	-0.045008

16	2.856094	0.736277	-1.445892
8	3.865867	0.756628	-2.518374
8	1.689754	-0.183208	-1.848417
6	2.101393	2.361717	-1.417101
6	2.313876	3.205114	-0.333815
6	1.265436	2.750174	-2.466137
6	1.664866	4.436891	-0.286170
1	2.964700	2.888982	0.474003
6	0.615553	3.974565	-2.399123
1	1.107470	2.093712	-3.315940
6	0.799179	4.833820	-1.305914
1	1.825056	5.089457	0.567440
1	-0.048875	4.270999	-3.206561
1	-1.441954	-4.348648	-0.775175
6	0.058093	6.141536	-1.239623
1	-1.023998	5.972247	-1.261590
1	0.298708	6.691984	-0.326893
1	0.303889	6.774920	-2.098504
1	1.989920	-0.232846	1.419586

Supplementary Table 19

R3

Standard orientation:

6	-2.740154	0.118237	0.849290
15	-0.991296	0.329273	1.371098
6	-2.993517	-0.164120	-0.519511
6	-5.231678	-0.052369	-0.134818
6	-4.978761	0.157543	1.252080
15	-1.543536	-0.586472	-1.546859
46	0.355343	-0.160397	-0.378844
6	-1.682369	-2.411764	-1.896682
6	-1.963924	-3.125462	-0.566586
1	-1.225885	-2.864344	0.199947
1	-2.963027	-2.895658	-0.182926
6	-2.803826	-2.687648	-2.904384

1	-2.889809	-3.771434	-3.041014
1	-3.769646	-2.312131	-2.554440
1	-2.588267	-2.243475	-3.880774
6	-0.328443	-2.867911	-2.461525
1	0.474574	-2.743689	-1.728793
1	-0.393731	-3.931169	-2.716853
1	-0.057492	-2.319514	-3.369836
6	-1.676060	0.352372	-3.092051
1	-2.652437	0.191825	-3.555729
1	-1.544653	1.413518	-2.866349
1	-0.884621	0.026547	-3.771656
6	-0.827482	-0.700301	2.856191
1	0.180464	-0.602229	3.264859
1	-1.558162	-0.385377	3.605700
1	-1.010415	-1.742092	2.582922
6	-0.776018	2.125336	1.841498
6	-1.845508	2.578922	2.842694
1	-1.611085	3.606167	3.143607
1	-2.847152	2.575661	2.406965
1	-1.855410	1.961648	3.745767
6	-0.871520	2.954626	0.553532
1	-0.772412	4.015872	0.806237
1	-0.075171	2.695098	-0.150245
1	-1.836384	2.817336	0.052339
6	0.618924	2.268041	2.468862
1	1.401300	1.863500	1.821261
1	0.820036	3.333031	2.629299
1	0.672873	1.767827	3.441127
7	-3.708481	0.248687	1.725263
7	-4.208815	-0.229807	-1.011484
6	-6.067183	0.292300	2.149199
6	-6.568114	-0.107008	-0.601934
6	-7.349435	0.231032	1.670297
1	-5.854378	0.448571	3.201598
6	-7.601041	0.032791	0.287362
1	-6.740688	-0.266435	-1.661224

1	-8.187508	0.335892	2.351562
1	-8.627014	-0.010792	-0.063207
6	2.737621	-1.022562	1.305141
6	2.581049	-2.386252	0.637000
6	1.589964	-3.245578	1.125506
6	3.352680	-2.819321	-0.447706
6	1.372557	-4.498785	0.557071
1	0.978697	-2.927994	1.966785
6	3.136220	-4.070475	-1.020317
1	4.121358	-2.178016	-0.863991
6	2.147763	-4.916195	-0.520844
1	0.595457	-5.144284	0.955722
1	3.746286	-4.384606	-1.862149
1	1.982645	-5.890395	-0.971038
6	4.030464	-0.818266	2.159061
6	4.090982	-1.982914	3.162318
1	3.162020	-2.064329	3.739244
1	4.264452	-2.940913	2.662188
1	4.911304	-1.821643	3.869681
6	3.882790	0.492058	2.944732
1	3.848084	1.357135	2.277548
1	2.967469	0.492434	3.548087
1	4.732735	0.621805	3.623414
6	5.346373	-0.781712	1.376545
1	5.388414	0.062568	0.685857
1	6.179090	-0.680495	2.081852
1	5.511321	-1.701209	0.807323
7	2.336405	0.131646	0.455714
16	2.854637	0.430961	-1.028209
8	4.257096	0.188607	-1.371634
8	1.877332	-0.279434	-1.962852
6	2.475114	2.160435	-1.215578
6	2.931806	3.067620	-0.261944
6	1.711939	2.577899	-2.301536
6	2.593632	4.406929	-0.392901
1	3.525723	2.733177	0.581722

6	1.385876	3.925724	-2.417208
1	1.359639	1.860935	-3.035143
6	1.810548	4.855297	-1.464544
1	2.938125	5.117540	0.352990
1	0.784209	4.255418	-3.259284
1	-1.907507	-4.207141	-0.728821
6	1.413991	6.302856	-1.562568
1	0.593524	6.519546	-0.868810
1	2.247553	6.959514	-1.297310
1	1.076272	6.557436	-2.570232
1	1.950989	-0.995071	2.063799

Supplementary Table 20

R4

Standard orientation:

6	-2.617615	-0.057841	0.921980
15	-0.865153	0.289126	1.340545
6	-2.954521	-0.215337	-0.445709
6	-5.163694	-0.215778	0.092012
6	-4.821509	-0.164908	1.474930
15	-1.574382	-0.514813	-1.603559
46	0.418983	-0.188538	-0.509333
6	-1.809096	-2.273513	-2.194604
6	-2.021450	-3.159129	-0.958011
1	-1.220838	-3.027675	-0.222200
1	-2.981158	-2.960640	-0.470886
6	-3.019337	-2.356368	-3.135337
1	-3.177941	-3.408420	-3.396349
1	-3.932963	-1.980506	-2.668351
1	-2.846171	-1.804008	-4.063827
6	-0.543538	-2.716420	-2.942637
1	0.309789	-2.825402	-2.267856
1	-0.735521	-3.694401	-3.396656
1	-0.274308	-2.023525	-3.747682
6	-1.782571	0.635628	-2.990321

1	-2.797246	0.568069	-3.390070
1	-1.596055	1.650247	-2.629323
1	-1.058500	0.391584	-3.771681
6	-0.557367	-0.696086	2.832908
1	0.400753	-0.420511	3.277253
1	-1.358843	-0.501978	3.550490
1	-0.554248	-1.756461	2.571110
6	-0.830717	2.097429	1.804526
6	-1.914167	2.443031	2.834351
1	-1.763889	3.485963	3.135336
1	-2.922066	2.354727	2.423323
1	-1.846672	1.824810	3.734117
6	-1.065093	2.895634	0.516249
1	-1.039136	3.965204	0.749366
1	-0.295843	2.690846	-0.231802
1	-2.045169	2.672234	0.079102
6	0.551520	2.386636	2.408104
1	1.362598	1.989187	1.795113
1	0.676572	3.471116	2.495363
1	0.637142	1.957880	3.411472
7	-3.525966	-0.065635	1.869019
7	-4.198559	-0.261687	-0.862103
6	-5.848317	-0.183565	2.451175
6	-6.527187	-0.257350	-0.290870
6	-7.158070	-0.232256	2.052346
1	-5.567823	-0.148402	3.498640
6	-7.499278	-0.265065	0.674511
1	-6.767761	-0.291876	-1.348255
1	-7.950113	-0.242523	2.794074
1	-8.545536	-0.300579	0.389086
6	2.621069	-1.127556	1.245065
6	2.601788	-2.381780	0.372389
6	1.579977	-3.314564	0.584183
6	3.527960	-2.640073	-0.643496
6	1.475491	-4.467232	-0.191789
1	0.854162	-3.134227	1.373763

6	3.424728	-3.787652	-1.426249
1	4.325995	-1.934933	-0.842154
6	2.399228	-4.705943	-1.205272
1	0.669779	-5.171647	-0.006954
1	4.153560	-3.966082	-2.211611
1	2.323050	-5.598991	-1.818257
6	3.753565	-1.103327	2.325399
6	3.481253	-2.299556	3.259010
1	2.482352	-2.239243	3.707609
1	3.559973	-3.255629	2.731913
1	4.214031	-2.307629	4.072927
6	3.619965	0.176977	3.161652
1	3.845560	1.070969	2.578400
1	2.603721	0.274457	3.561775
1	4.309786	0.139086	4.012272
6	5.183737	-1.258537	1.790236
1	5.448831	-0.484474	1.069011
1	5.889204	-1.189731	2.626395
1	5.328423	-2.237690	1.323487
7	2.294017	0.122354	0.490453
16	3.357587	0.944862	-0.420274
8	4.502314	1.445509	0.362452
8	3.746171	0.258642	-1.676192
6	2.423608	2.380609	-0.924224
6	2.475956	3.527834	-0.135939
6	1.732921	2.382809	-2.135283
6	1.800567	4.669401	-0.548516
1	3.046611	3.532217	0.786243
6	1.054135	3.531340	-2.529942
1	1.735305	1.508073	-2.775722
6	1.071266	4.686599	-1.742459
1	1.841269	5.563296	0.067524
1	0.514427	3.531440	-3.472777
1	-2.016578	-4.207829	-1.273402
6	0.304046	5.913497	-2.152228
1	-0.662738	5.945503	-1.636198

1	0.845346	6.826520	-1.888878
1	0.107654	5.921564	-3.227464
1	1.727246	-1.219493	1.862889
1	1.680279	-0.651190	-1.835208
1	0.992243	-0.692966	-2.206082

Supplementary Table 21

TS3(*R*) *v* = 1220*i*

Standard orientation:

6	-3.063848	-0.323256	0.814994
15	-1.751152	0.655830	1.629849
6	-2.747774	-0.932873	-0.427113
6	-4.940548	-1.483155	-0.703358
6	-5.234445	-0.963722	0.590160
15	-0.980969	-1.020844	-0.920071
46	0.232877	0.251543	0.605191
6	-0.529430	-2.832295	-0.861836
6	-0.404017	-3.212732	0.619481
1	0.359982	-2.613828	1.125598
1	-1.355135	-3.079536	1.148289
6	-1.570917	-3.724792	-1.546092
1	-1.175325	-4.746666	-1.569015
1	-2.519597	-3.743695	-1.005228
1	-1.763897	-3.418788	-2.578528
6	0.816550	-2.976380	-1.583760
1	1.570278	-2.291959	-1.191313
1	1.182131	-3.998742	-1.443128
1	0.714023	-2.801142	-2.659014
6	-0.974299	-0.470833	-2.649855
1	-1.600277	-1.133185	-3.253307
1	-1.369632	0.546918	-2.693637
1	0.050828	-0.478207	-3.023749
6	-1.792475	0.205374	3.384982
1	-1.093743	0.839791	3.934884
1	-2.802977	0.330756	3.781242

1	-1.485818	-0.838906	3.482922
6	-2.247558	2.443391	1.418513
6	-3.612135	2.717097	2.064670
1	-3.847279	3.777867	1.924287
1	-4.412510	2.131071	1.607772
1	-3.602645	2.517182	3.140406
6	-2.299230	2.721858	-0.090134
1	-2.519501	3.783038	-0.249074
1	-1.342071	2.499570	-0.572650
1	-3.083777	2.140094	-0.586043
6	-1.180972	3.321884	2.086665
1	-0.194115	3.179305	1.640262
1	-1.463298	4.371459	1.950072
1	-1.111638	3.130842	3.162459
7	-4.270049	-0.360109	1.331756
7	-3.672734	-1.468520	-1.189954
6	-6.556419	-1.043145	1.094007
6	-5.979912	-2.051034	-1.481449
6	-7.539728	-1.605579	0.323370
1	-6.759057	-0.644742	2.082725
6	-7.250720	-2.108463	-0.972498
1	-5.737656	-2.437989	-2.465736
1	-8.555270	-1.667044	0.700691
1	-8.050563	-2.545936	-1.561042
6	3.278599	-0.564774	1.018456
6	3.567597	-1.933563	0.426422
6	3.149921	-3.068389	1.127113
6	4.257079	-2.113292	-0.777677
6	3.395026	-4.349590	0.637576
1	2.620773	-2.947697	2.069060
6	4.494554	-3.390550	-1.276113
1	4.617936	-1.255833	-1.335094
6	4.064496	-4.513952	-0.571320
1	3.057032	-5.215592	1.199016
1	5.024675	-3.508390	-2.216499
1	4.252898	-5.509286	-0.962369

6	4.523509	0.091424	1.730683
6	4.710181	-0.674074	3.053983
1	3.831824	-0.574312	3.702636
1	4.886502	-1.740971	2.881654
1	5.575585	-0.278703	3.596166
6	4.232361	1.560160	2.059884
1	4.217537	2.174888	1.156584
1	3.276798	1.679202	2.584394
1	5.017295	1.952726	2.715387
6	5.844013	0.000934	0.953459
1	5.777780	0.480923	-0.023671
1	6.630126	0.504911	1.527279
1	6.157341	-1.037658	0.811329
7	2.425652	0.316249	0.169269
16	2.797684	0.889933	-1.308282
8	4.199849	1.335543	-1.373237
8	2.394654	-0.030131	-2.385632
6	1.744542	2.322594	-1.429461
6	1.793473	3.320254	-0.455214
6	0.950966	2.473205	-2.562598
6	1.015867	4.459101	-0.612220
1	2.418566	3.209266	0.424399
6	0.179552	3.622030	-2.704778
1	0.935966	1.701666	-3.323527
6	0.195598	4.626732	-1.734082
1	1.040222	5.228259	0.154333
1	-0.449233	3.733764	-3.583436
1	-0.117669	-4.267107	0.699871
6	-0.644428	5.864067	-1.889165
1	-0.983040	6.235681	-0.918011
1	-0.064552	6.664071	-2.363713
1	-1.519850	5.672934	-2.515289
1	2.586474	-0.750006	1.847191
1	0.990524	1.265054	1.776474
1	1.687273	1.101382	1.152035

Supplementary Table 22

R5

Standard orientation:

6	3.166460	-0.683256	0.250068
15	1.804668	-1.449325	1.238411
6	2.965020	0.578420	-0.385688
6	4.995239	0.368628	-1.417589
6	5.237410	-0.834708	-0.702382
15	1.485188	1.581392	0.057103
46	0.099424	0.025206	1.272852
6	2.189764	2.949363	1.136210
6	2.808846	2.287419	2.375570
1	2.076113	1.674563	2.913088
1	3.665171	1.655415	2.115856
6	3.239740	3.794856	0.407634
1	3.614032	4.558549	1.099187
1	4.091613	3.199469	0.071060
1	2.815674	4.312203	-0.458301
6	1.020942	3.851648	1.560186
1	0.303650	3.320912	2.191502
1	1.415130	4.690129	2.145273
1	0.488545	4.270951	0.699699
6	1.031295	2.350741	-1.534267
1	1.900893	2.819300	-1.999800
1	0.641548	1.577298	-2.201436
1	0.254561	3.101893	-1.370659
6	2.560056	-1.735135	2.869481
1	1.885519	-2.344605	3.475454
1	3.522674	-2.239846	2.759058
1	2.707710	-0.770646	3.361251
6	1.485309	-3.117952	0.451612
6	2.702267	-4.047437	0.573070
1	2.450669	-4.999126	0.091810
1	3.586977	-3.638790	0.083086
1	2.948429	-4.259354	1.617939

6	1.145689	-2.876638	-1.024938
1	0.861751	-3.828585	-1.486607
1	0.308972	-2.182762	-1.146785
1	2.003949	-2.480046	-1.577584
6	0.301833	-3.769705	1.177885
1	-0.605384	-3.170435	1.101075
1	0.106343	-4.745893	0.721067
1	0.517189	-3.932257	2.239265
7	4.291508	-1.347523	0.126059
7	3.850887	1.073092	-1.220794
6	6.464530	-1.521047	-0.876645
6	5.973980	0.857381	-2.317930
6	7.397785	-1.022952	-1.748091
1	6.631850	-2.436241	-0.318014
6	7.149478	0.170329	-2.475387
1	5.769122	1.775733	-2.858542
1	8.338709	-1.544367	-1.891043
1	7.905271	0.540080	-3.160791
6	-3.372581	1.460502	-0.519388
6	-3.024267	1.079524	0.908370
6	-3.846845	0.226289	1.629169
6	-1.865921	1.593125	1.533455
6	-3.543451	-0.121488	2.952284
1	-4.742341	-0.173813	1.161934
6	-1.547906	1.226730	2.847329
1	-1.297086	2.372723	1.040819
6	-2.399709	0.364020	3.559942
1	-4.210675	-0.782530	3.496509
1	-0.701502	1.684732	3.352628
1	-2.159016	0.095502	4.583266
6	-3.893284	2.916146	-0.718992
6	-5.106327	3.120798	0.197891
1	-5.881862	2.370450	0.004882
1	-4.830935	3.060153	1.255779
1	-5.544266	4.108931	0.022744
6	-4.338144	3.065915	-2.179899

1	-3.494360	2.960683	-2.867508
1	-5.088572	2.313187	-2.446565
1	-4.781537	4.055115	-2.334572
6	-2.831552	3.979257	-0.411389
1	-1.903838	3.788999	-0.961866
1	-3.200583	4.963392	-0.718824
1	-2.603119	4.039523	0.656513
7	-2.219376	1.163912	-1.399015
16	-2.312499	-0.078966	-2.484003
8	-3.155587	0.332793	-3.602427
8	-0.918019	-0.445789	-2.751263
6	-3.140321	-1.422212	-1.673511
6	-4.357665	-1.880032	-2.167677
6	-2.577337	-1.967717	-0.521270
6	-5.012443	-2.907915	-1.497430
1	-4.789662	-1.435536	-3.057323
6	-3.246669	-2.989193	0.136095
1	-1.643681	-1.577476	-0.122287
6	-4.469968	-3.476170	-0.341397
1	-5.963878	-3.268108	-1.877403
1	-2.822397	-3.409837	1.043385
1	3.164553	3.065492	3.060346
6	-5.171032	-4.599340	0.371423
1	-6.238124	-4.617147	0.136173
1	-4.746330	-5.563707	0.069898
1	-5.052615	-4.514012	1.455304
1	-4.199731	0.811482	-0.828229
1	-0.481126	-1.182887	2.073448
1	-1.328944	1.102577	-0.912066

Supplementary References

[1] Sasano, Y., Murakami, K., Nishiyama, T., Kwon, E., Iwabuchi, Y. 3-Methyl-4-oxa-5-azahomoadamantane: Alkoxyamine-Type Organocatalyst for Alcohol Oxidation. *Angew. Chem. Int. Ed.* **52**, 12624–12627 (2013).

[2] Díaz-Valenzuela, M. B., Phillips, S. D., France, M. B., Gunn, M. E., Clarke, M. L. Enantioselective Hydrogenation and Transfer Hydrogenation of Bulky Ketones Catalysed by a Ruthenium Complex of a Chiral Tridentate Ligand. *Chem. Eur. J.* **15**, 1227–1232 (2009).

[3] Liu, C., Achtenhagen, M., Szostak, M. Chemoselective Ketone Synthesis by the Addition of Organometallics to *N*-Acylazetidines. *Org. Lett.* **18**, 2375-2378 (2016).

[4] Lo Fiego, M. J., Lockhart, M. T., Chopa, A. B. Catalyst-free alkanoylation of aromatic rings via arylstannanes. Scope and limitations. *J. Organomet. Chem.* **694**, 3674–3678 (2009).

[5] Palmer, B. D., Boyd, M., Denny, W. A. Aromatic Lithiation Directed by the Carboxylic Acid Groups. Synthesis of 9-Substituted Dibenzodioxin-1-carboxylic Acids and 6-Substituted Phenoxathiin-4-carboxylic Acids. *J. Org. Chem.* **55**, 438–441 (1990).

[6] Okuma, K., Izaki, T., Kubo, K., Shioji, K., Yokomori, Y. Reaction of Ketone Hydrazones with Diselenium Dihalides: Simple Synthesis of Δ^3-1,3,4-Selenadiazolines and 2,5-Diarylselenophenes. *Bull. Chem. Soc. Jpn.,* **78**, 1121–1126 (2005).

[7] Ogiwara, Y., Miyake, M., Kochi, T., Kakiuchi, F. Syntheses of $RuHCl(CO)(PAr_3)_3$ and $RuH_2(CO)(PAr_3)_3$ Containing Various Triarylphosphines and Their Use for Arylation of Sterically Congested Aromatic C–H Bonds. *Organometallics,* **36**, 159–164 (2017).

[8] Ceccon, A., Corvaja, C., Giacometti, G., Venzo, A. Benzyl radicals with O and O containing substituents at C(7). *J. Chem. Soc., Perkin Trans.* **2**, 283–288 (1978).

[9] Lauterbach, T., Arndt, S., Rudolph, M., Rominger, F., Hashmi, A. S. K. Gold Catalysis: β-Ketonaphthalenes via Molecular Gymnastics of 1,6-Diyne-4-en-3-ols. *Adv. Synth. Catal.* **355**, 1755–1761 (2013).

[10] Chen, J., Zhang, Z., Liu, D., Zhang, W. Palladium-Catalyzed Chemo- and Enantioselective C-O Bond Cleavage of α-Acyloxy Ketones via Hydrogenolysis. *Angew. Chem. Int. Ed.* **55**, 8444–8447 (2016).

[11] Yamada, Y. M. A., Jin, C., Uozumi, Y. H_2O_2-Oxidation of Alcohols Promoted by Polymeric Phosphotungstate Catalysts. *Org. Lett.* **12**, 4540–4543 (2010).

[12] Ortiz, P., Collados, J. F., Harutyunyan, S. R. Direct Synthesis of Enolizable *N*-Sulfonyl Ketimines Under Microwave Irradiation. *Eur. J. Org. Chem.* 1247–1250 (2016).

[13] Yang, Q., Shang, G., Gao, W., Deng, J., Zhang, X. A Highly Enantioselective, Pd-TangPhos-Catalyzed Hydrogenation of *N*-Tosylimines. *Angew. Chem. Int. Ed.* **45**, 3832–3835 (2006).

[14] Beisel, T., Manolikakes, G. Palladium-Catalyzed Enantioselective Three-Component Synthesis of -Substituted Amines. *Org. Lett.* **17**, 3162–3166 (2015).

[15] Berger, R., Rabbat, P. M. A., Leighton. G. L. Toward a Versatile Allylation Reagent: Practical, Enantioselective Allylation of Acylhydrazones Using Strained Silacycles. *J. Am. Chem. Soc.* **125**, 9596-9597 (2003).

[16] Chai, J.-D., Head-Gordon, M. Long-range corrected hybrid density functionals with damped atom–atom dispersion corrections. *Phys. Chem. Chem. Phys*., 2008, **10**, 6615–6620.

[17] Frisch, M. J., Trucks, G. W., Schlegel, H. B., Scuseria, G. E., Robb, M. A., Cheeseman, J. R., Scalmani, G., Barone, V., Mennucci, B., Petersson, G. A., Nakatsuji, H., Caricato, M., Li, X., Hratchian, H. P., Izmaylov, A. F., Bloino, J., Zheng, G., Sonnenberg, J. L., Hada, M., Ehara, M., Toyota, K., Fukuda, R., Hasegawa, J., Ishida, M., Nakajima, T., Honda, Y., Kitao, O., Nakai, H., Vreven, T., Montgomery, J. A., Peralta, Jr. J. E., Ogliaro, F., Bearpark, M., Heyd, J. J., Brothers, E., Kudin, K. N., Staroverov, V. N., Kobayashi, R., Normand, J., Raghavachari, K., Rendell, A., Burant, J. C., Iyengar, S. S., Tomasi, J., Cossi, M., Rega, N., Millam, J. M., Klene, M., Knox, J. E., Cross, J. B., Bakken, V., Adamo, C., Jaramillo, J., Gomperts, R., Stratmann, R. E., Yazyev, O., Austin, A. J., Cammi, R., Pomelli, C., Ochterski, J. W., Martin, R. L., Morokuma, K., Zakrzewski, V. G., Voth, G. A., Salvador, P., Dannenberg, J. J., Dapprich, S., Daniels, A. D., Farkas, O., Foresman, J. B., Ortiz, J. V., Cioslowski, J., Fox, D. J. *Gaussian 09*, *Revision D. 01*, Gaussian, Inc., Wallingford CT, 2009.

[18] Bergner, A., Dolg, M., Kuechle, W., Stoll, H., Preuß, H. *Ab initio* energy-adjusted pseudopotentials for elements of groups 13–17. *Mol. Phys.*, **80**, 1431–1441 (1993).

[19] Ditchfield, R., Hehre, W. J., Pople, J. A. Self-Consistent Molecular-Orbital Methods. IX. An Extended Gaussian-Type Basis for Molecular-Orbital Studies of Organic Molecules. *J. Chem. Phys.*, **54**, 724–728 (1971).

[20] Hehre, W. J., Ditchfield, R., Pople, J. A. Self-Consistent Molecular Orbital Methods. XII. Further Extensions of Gaussian-Type Basis Sets for Use in Molecular Orbital Studies of Organic Molecules. *J. Chem. Phys.*, **56**, 2257–2261 (1972).

[21] Hariharan, P. C., Pople, J. A. The influence of polarization functions on molecular orbital hydrogenation energies. *Theor. Chim. Acta*, **28**, 213–222 (1973).

[22] Hariharan, P. C., Pople, J. A. Accuracy of AH_n equilibrium geometries by single determinant molecular orbital theory. *Mol. Phys.*, **27**, 209–214 (1974).

[23] Gordon, M. S. The isomers of silacyclopropane. *Chem. Phys. Lett.*, **76**, 163–168 (1980).

[24] Marenich, A. V., Cramer, C. J., Truhlar, D. G. Universal Solvation Model Based on Solute Electron Density and on a Continuum Model of the Solvent Defined by the Bulk Dielectric Constant and Atomic Surface Tensions. *J. Phys. Chem. B*, **113**, 6378–6396 (2009).
